# SURGE complex of *Plasmodium falciparum* in the rhoptry-neck (SURFIN_4.2_-RON4-GLURP) contributes to merozoite invasion

**DOI:** 10.1371/journal.pone.0201669

**Published:** 2018-08-09

**Authors:** Maria del Pilar Quintana, Jun-Hong Ch’ng, Arash Zandian, Maryam Imam, Kjell Hultenby, Michael Theisen, Peter Nilsson, Ulrika Qundos, Kirsten Moll, Sherwin Chan, Mats Wahlgren

**Affiliations:** 1 Department of Microbiology, Tumor and Cell Biology (MTC), Biomedicum, Karolinska Institutet, Stockholm, Sweden; 2 Department of Microbiology and Immunology, National University of Singapore, Singapore, Singapore; 3 Affinity Proteomics, Science for Life Laboratory, School of Biotechnology, KTH-Royal Institutet of Technology, Stockholm, Sweden; 4 Division of Clinical Research Centre, Department of Laboratory Medicine, Karolinska Institutet, Huddinge, Sweden; 5 Department for Congenital Disorders, Statens Serum Institut, Copenhagen, Denmark; 6 Centre for Medical Parasitology, Department of International Health, Immunology and Microbiology, University of Copenhagen, Copenhagen, Denmark; Bernhard Nocht Institute for Tropical Medicine, GERMANY

## Abstract

*Plasmodium falciparum* invasion into red blood cells (RBCs) is a complex process engaging proteins on the merozoite surface and those contained and sequentially released from the apical organelles (micronemes and rhoptries). Fundamental to invasion is the formation of a moving junction (MJ), a region of close apposition of the merozoite and the RBC plasma membranes, through which the merozoite draws itself before settling into a newly formed parasitophorous vacuole (PV). SURFIN_4.2_ was identified at the surface of the parasitized RBCs (pRBCs) but was also found apically associated with the merozoite. Using antibodies against the N-terminus of the protein we show the presence of SURFIN_4.2_ in the neck of the rhoptries, its secretion into the PV and shedding into the culture supernatant upon schizont rupture. Using immunoprecipitation followed by mass spectrometry we describe here a novel protein complex we have named SURGE where SURFIN_4.2_ forms interacts with the rhoptry neck protein 4 (RON4) and the Glutamate Rich Protein (GLURP). The N-terminal cysteine-rich–domain (CRD) of SURFIN_4.2_ mediates binding to the RBC membrane and its interaction with RON4 suggests its involvement in the contact between the merozoite apex and the RBC at the MJ. Supporting this suggestion, we also found that polyclonal antibodies to the extracellular domain (including the CRD) of SURFIN_4.2_ partially inhibit merozoite invasion. We propose that the formation of the SURGE complex participates in the establishment of parasite infection within the PV and the RBCs.

## Introduction

Malaria is a vector borne disease that is still endemic in more than a hundred countries with 196 to 263 million malaria cases and an estimated of 445000 deaths during 2016, most of which are attributed to *Plasmodium falciparum* infections [1]. Malaria clinical symptoms are associated with the parasite’s asexual cycle inside the host’s RBCs initiated by the rupture of the pRBCs and the release of the parasite invasive stages, the merozoites.

The invasion process into the RBCs is a complex process and can be divided in a successive series of steps, beginning with the pRBC rupture and subsequent merozoite egress, followed by the initial contact (weak binding) with the new host cell, the merozoite re-orientation (accompanied by high-avidity binding and deformation) and a final entry phase, where the PV is formed [2]. Proteins on the surface or contained inside the rhoptries and the micronemes play a major role in the invasion process and have been considered important candidates for vaccine development [3].

Consistently, parasite derived proteins with documented roles in merozoite invasion, operate as part of a protein complex, suggesting that such complexes are critical players in this vital biological parasite process. For example, Merozoite Surface Protein 1 (MSP-1) believed to mediate the initial attachment of the merozoites to the RBCs, forms a complex with other surface proteins such as MSP-6 and MSP-7 [4–6]. In contrast, the RON complex formed by the rhoptry neck proteins 2, 4 and 5 (RON2, 4 and 5), is translocated from the merozoite-apex into the RBC membrane where it functions as a receptor for the micronemal protein Apical Membrane Antigen 1 (AMA-1). Together they form a moving junction (MJ), a site of close proximity between the parasite and the RBC membranes that moves rearwards as the parasite pushes its way into the RBC, acting as a trigger for further rhoptry secretion and parasite cytoskeleton activation [7–9]. The essential invasion protein *P*. *falciparum* reticulocyte binding-like homologue 5 (PfRh5) also has a role as a member of a merozoite membrane-tethered complex interacting with Pf113, *P*. *falciparum* Rh5 interacting protein (PfRipr), and Cysteine-rich protective antigen (CyRPA) [10–12].

*P*. *falciparum* is known for the ability to use different and sometimes redundant invasion pathways, where several invasion related antigens may be dispensable and compensated for, including members of the EBA and Rh protein families. Even though there is mounting experimental evidence understanding which proteins are essential which are not, much less is known regarding their precise function. In several cases, proteins associated with the surface or the apical end of the merozoites have unknown roles in the invasion process [13]. Such is the case of the SURFIN_4.2_ protein investigated herein, a parasite antigen displayed on the surface of the pRBCs and associated with the apical end of the merozoites [14,15].

**Fig 1:**
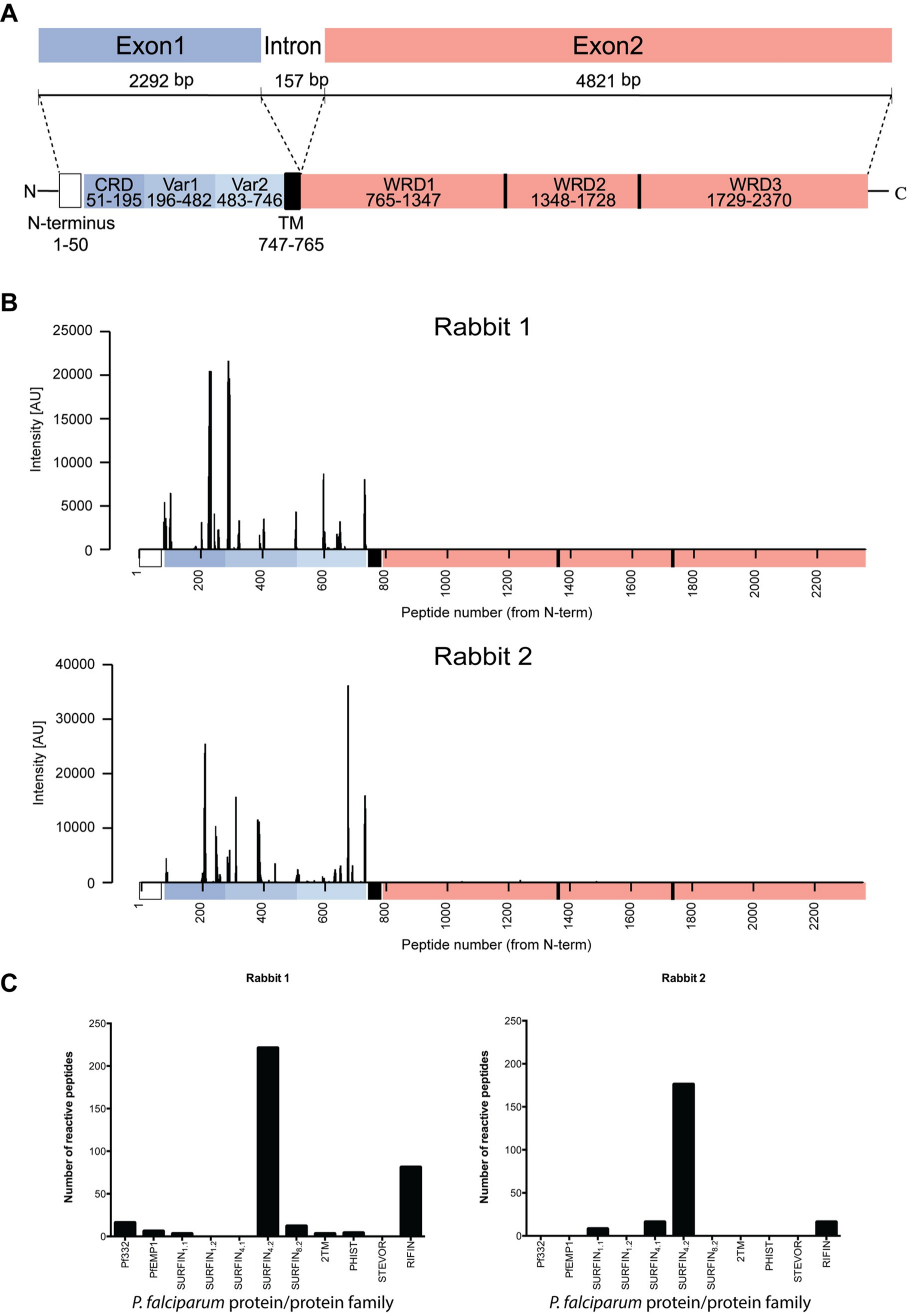
SURFIN_4.2_ gene and its encoding protein. **Polyclonal antibody specificity tested on a peptide array. (A)** Schematic representation of the SURFIN_4.2_ protein and its encoding gene (PfIT_0422600). CRD: Cysteine-rich domain; Var1 and 2: Variable region 1 and 2; TM: Transmembrane domain; WR: Tryptophan-rich domain. (**B)** Anti-SURFIN_4.2_ polyclonal antibodies reactivity on a peptide array. Reactivity profile of each anti-SURFIN_4.2_ polyclonal antibody against the whole sequence of the SURFIN_4.2_ protein. Sequence is depicted from N- to C terminus. (**C)** Number of reactive peptides for each polyclonal antibody discriminated by protein family. For the SURFIN family each member is depicted separately.

SURFIN_4.2_ is a high molecular weight protein (≈286KDa) encoded by a two-exon gene. The encoded protein is closely related to *P*. *vivax* subtelomeric transmembrane protein 1 (PvSTP1) and has an extracellular segment (≈750aa) followed by a transmembrane domain and a long intracellular domain twice as big as the extracellular domain (Fig 1A). The N-terminal segment contains a cysteine rich domain (CRD) similar to the external CRD of the *P*. *vivax* VIR protein family and the PvSTP1 protein. Further, the intracellular domain contains several tryptophan-rich domains (WRD) that are related to the WRDs of PvSTP1, antigen Pf332, SICAvar and the acidic terminal segment (ATS) of *P*. *falciparum* erythrocyte membrane protein 1 (PfEMP1) [14,16]. SURFIN_4.2_ is co-transported with PfEMP1 and RIFIN to the pRBC cytoplasm and membrane, being associated with the MCs and the knobs. The protein has also been observed associated with the merozoites, both with the parasite membrane and as a cap-liked zone in the apex of released merozoites [14].

Using specificity-validated antibodies towards the N-terminal segment of SURFIN_4.2_, including the CRD and the two variable regions (VAR1 and VAR2) just before the predicted transmembrane domain, we provide evidence that SURFIN_4.2_ is localized at the merozoite apex and specifically in the neck of the rhoptries, as well as at the surface of the nascent, free and invading merozoites. Immunoprecipitation (IP) followed by mass spectrometry (MS) identified a new protein complex we have named SURGE (**SU**RFIN_4.2_-**R**ON4-**G**LURP compl**E**x), where SURFIN_4.2_ binds GLURP and RON4. Data also indicate that the CRD of SURFIN_4.2_ expressed on the surface of CHO cells, binds RBCs. Further, since RON4 is also part of the RON complex and participates in MJ formation, we hypothesize that SURGE is present at the merozoite interaction site with the RBC.

## Results

### Expression and purification of recombinant SURFIN_4.2_

A gene fragment, encoding the full extracellular domain of SURFIN_4.2_ (PfIT_0422600) was codon optimized for expression in *Escherichia coli* and used to produce an N-terminal His-tagged recombinant protein (Panel A in S1 Fig). Protein was only expressed after induction with IPTG (Isopropyl β-D-1-thiogalactopyranoside) and was mostly expressed in the insoluble pellet (Panel A in S1 Fig). Attempts to bring the protein into the soluble fraction by expressing only the first 667 amino acids (avoiding a long stretch of hydrophobic amino acids between position 668–686) or by adding a solubilization tag (MBP: maltose binding protein) were unsuccessful (data not shown). Therefore, the original construct, covering the entire extracellular domain was extracted from inclusion bodies (IBs) and purified by immobilized metal affinity chromatography (IMAC) on a nickel-charged affinity resin. After elution with imidazole (150 mM) a dominant band above the 100KDa marker was observed (Panel B in S1 Fig, solid arrow) and under non-reducing conditions, dimer formation was observed (Panels B and C in S1 Fig, dashed arrow). After protein concentration, many different bands below the dominant band were observed, however, immunoblot with anti-His antibodies detected all these bands (Panel C in S1 Fig) suggesting they were truncated versions of the protein of interest rather than contaminants. The purified recombinant protein was used for animal immunization to generate polyclonal antibodies.

### Antibodies against SURFIN_4.2_ recognize the protein region used for the immunization

Specificity of the antibodies generated in animals (two rabbits) upon immunization with the SURFIN_4.2_ recombinant protein was tested on an ultra-dense peptide array, including 175000 peptides (12 amino acids long with 11-residues overlap), covering several reported *P*. *falciparum* surface antigens families (2TM, PHISTs, RIFINs, STEVORs, SURFINs and a selected group of PfEMP1s members). The antibodies were specific, with all reactivity observed, confined to the extracellular segment (Fig 1B) and more importantly, most of the reactive peptides belonged to the protein used for immunization (Fig 1C, S1 Table) with very limited cross-reactivity with peptides belonging to other protein families included in the array. This was particularly evident for Rabbit 2 (Fig 1C, right panel), with a specificity of more than 80%, with 176 reactive peptides (out of 216) belonging to the SURFIN_4.2_ protein. This polyclonal antibody preparation was chosen for all the subsequent experiments hereby presented, as its cross-reactivity was significantly lower as compared with Rabbit 1. Non-immune rabbit IgG was also tested, and reactivity obtained was null against all the peptides present in the array, including those belonging to the SURFIN_4.2_ sequence (S1 Table).

### SURFIN_4.2_ is mostly expressed in late stage parasites and shed into culture supernatants

In order to extract the SURFIN_4.2_ protein from the parasite preparations, a panel of different detergents was tested including, non-ionic, zwitterionic and ionic detergents, with the purpose to optimize extraction preserving the native conformation of the SURFIN_4.2_ protein. Parasites were initially treated with saponin, due to its ability to disrupt both the RBC and the PV membrane, helping to remove host cell background, releasing hemoglobin and other soluble components [17]. When parasite saponin pellets were extracted with the different detergents, and both pellet and supernatant were analyzed by Sodium Dodecyl Sulfate -Polyacrylamide Gel Electrophoresis (SDS-PAGE) after extraction, all detergents had similar efficiencies, with majority of the protein being present in the soluble supernatant, while very little was observed in the insoluble pellet (Panel A in S2 Fig). A couple of exceptions were the detergents Octyl β-D-glucopyranoside (OG) and Octyl β-D-1-thioglucopyranoside (OTG) that showed a substantial amount of protein still present in the insoluble pellet. The 3-(Decyldimethylammonio)­propane­sulfonate inner salt (SB3-10) gave the most consistent solubilization effect and more importantly, it seemed to be more efficient at extracting the SURFIN_4.2_ protein not only as monomer (band above 242KDa marker) but also as part of higher molecular weight complexes (band above 480KDa marker and a broad band around the 1MDa markers) (Panel B in S2 Fig). Consequently, SB3-10 was used for all subsequent experiments.

**Fig 2:**
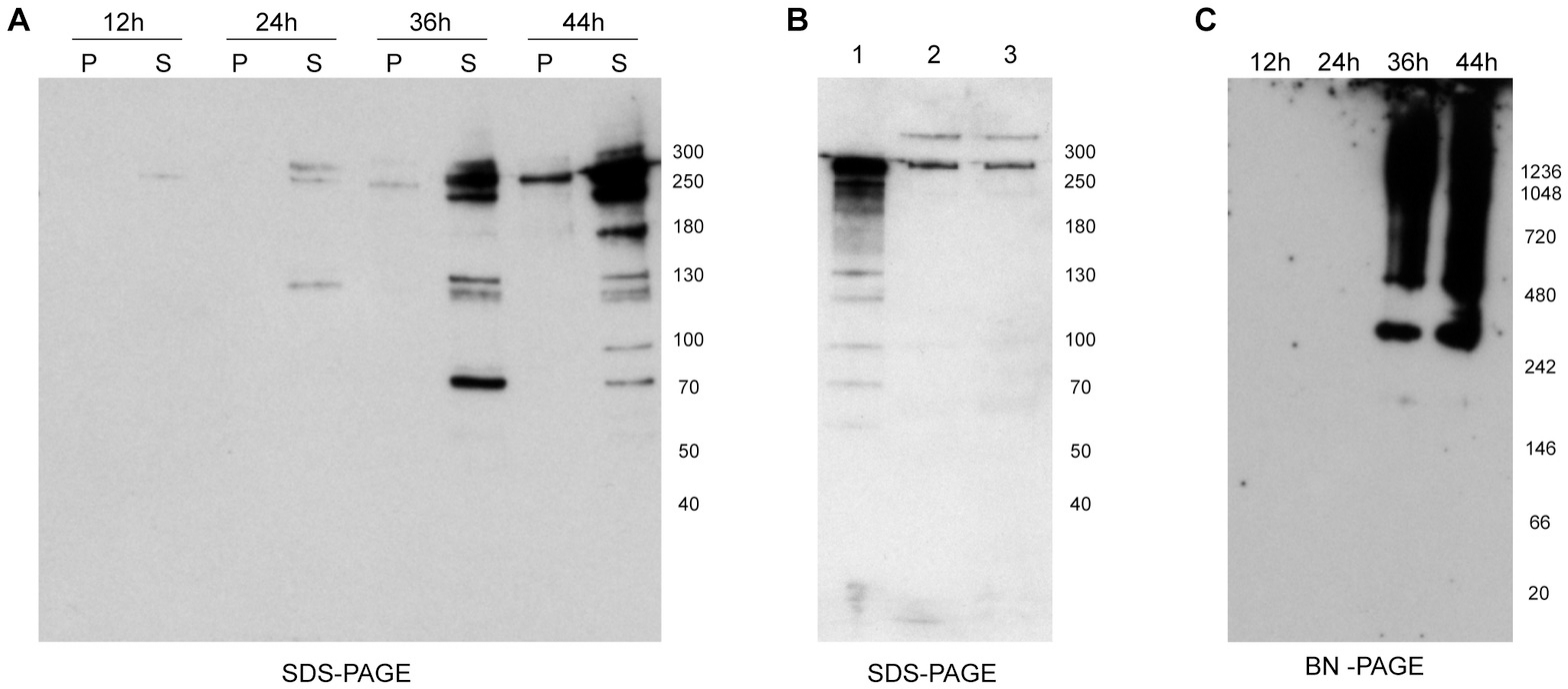
SURFIN_4.2_ protein expression during the parasite asexual cycle. All panels depict PAGE followed by immunoblot with αSURFIN_4.2_. (**A)** SDS-PAGE on a parasite time-course during the asexual cycle inside RBCs, pellet (P) and supernatant (S) after saponin pellet extraction with the detergent SB3-10. **(B)** SDS-PAGE on cellular pellet (lane 1) and culture supernatant (lanes 2 and 3, before and after ultracentrifugation respectively) upon schizont rupture. **(C)** BN-PAGE on a parasite time-course, only supernatants after extraction with SB3-10 were tested.

In order to assess the protein expression pattern during the 48-hour asexual cycle of the parasite, equal amounts of pRBCs were collected from a synchronized culture every 12 hours and analyzed by SDS and blue native polyacrylamide gels (BN-PAGE). It was observed that protein amount increased with cycle progression, with higher protein expression during the late trophozoite (36 hours) and schizont (44 hours) stages. Protein was not observed as a single band, instead several bands were detected (Fig 2A); a triplet of bands between 250-300KDa, a doublet of bands around 130 KDa and a band around 70KDa were consistently observed. The underlying cause for this observation is unclear, but could implicate the involvement of proteolytic processing of the protein or sample degradation despite the addition of protease inhibitors during preparation.

Several proteins involved in the invasion process are shed into the culture supernatant upon schizont rupture and during the invasion process itself [13]. Since SURFIN_4.2_ has been proposed to be involved in the invasion process (due to its association with the apical end of the merozoites) this possibility was tested here. Schizonts were purified and let to burst in the absence of RBCs, culture supernatants were collected followed by an ultracentrifugation step to remove cellular debris and the presence of SURFIN_4.2_ was tested by immunoblot. Bands detected by the anti-SURFIN_4.2_ antibodies were found both in culture supernatants before and after ultracentrifugation (Fig 2B, lanes 2 and 3) as well as in the cellular pellets (Fig 2B, lane 1). However, only two bands were observed in the supernatant fraction (above 250KDa and slightly below the 300KDa), whereas abundant processed proteins with lower molecular weight were detected in the cellular pellet, suggesting that the protein shed into the supernatant mostly corresponds to a partially processed protein and full length SURFIN_4.2_.

When the detergent soluble fractions for the four time points spanning the 48-hour asexual cycle were analyzed on BN-PAGE, SURFIN_4.2_ was only observed on the two last time points, corresponding to the late trophozoite and schizont stages. More importantly, the pattern observed during the initial detergent screening was consistently present, suggesting SURFIN_4.2_ is part of a protein complex formed during late stages (Fig 2C).

### SURFIN_4.2_ forms a complex with GLURP and RON4

**Fig 3:**
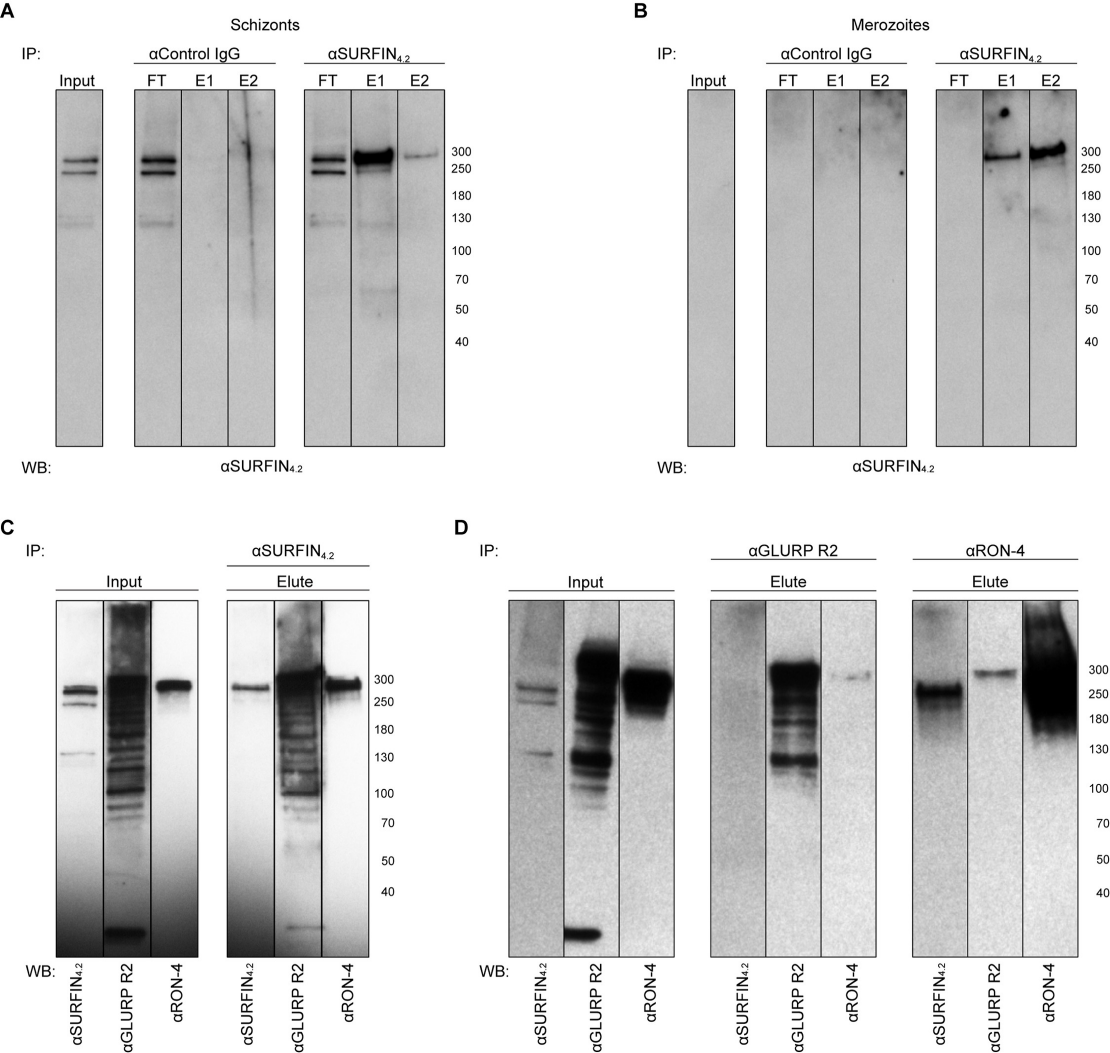
SURFIN_4.2_ protein forms a complex with GLURP and RON4. IP with αSURFIN_4.2_ specifically pulls down SURFIN_4.2_ from schizont (**A**) and merozoite (**B**) protein extract as compared with control IgGs. (**C)** IP with αSURFIN_4.2_ also pulls down GLURP and RON4. (**D)** IP with αGLURP R2 also pulls down RON4 but not SURFIN_4.2_; IP with αRON4 also pulls down GLURP and SURFIN_4.2_. Input corresponds to the supernatant fraction after SB3-10 extraction followed by detergent removal and pre-clearing on control beads; FT: Flow-through corresponds to unbound material; E1 and 2: Eluted fraction corresponds to bound material to the antibody-coupled beads.

In order to confirm that SURFIN_4.2_ forms a protein complex and to address the identity of the possible interacting partners, IP followed by MS was performed. Anti-SURFIN_4.2_ antibodies and control rabbit IgG (to rule out unspecific binding to rabbit IgG) were used to pull-down SURFIN_4.2_ together with its potential interacting partners from late schizonts (segmented and partially bursting) or purified merozoites protein extracts. Anti-SURFIN_4.2_ antibodies specifically pulled-down two bands above the 250KDa marker both from schizont and merozoite extracts (Fig 3A and 3B). For the schizont IP, faint bands of lower molecular sizes (below 130KDa and 70KDa markers) were also pulled-down (Fig 3A) and these bands partially correspond to the processed protein bands identified during the time course and observed in late stages (Fig 2A). None of the above-mentioned bands were observed when the IP was performed using control IgG (Fig 3A and 3B). Eluted fractions both from schizont and merozoite material were also analyzed by MS. Protein identification suggested that anti-SURFIN_4.2_ antibodies also pulled-down GLURP and RON4 (Table 1). MSP-1 seemed to also be present, however it was also detected with the IP performed with control IgG, indicating a nonspecific interaction with rabbit IgG and/or the matrix used for the IP (on despite of a pre-absorption step on control beads), therefore, this hit was considered a false positive.

**Table 1:** Proteins identified by Mass Spectrometry (MS) after immunoprecipitation (IP) with anti-SURFIN_4.2_ antibodies both from schizont and merozoite protein extract.

	Protein name	Molecular Weight	Score	Sequence coverage
	Merozoite Surface Protein 1 (MSP-1)	189101	664	8%
Schizonts	Glutamate Rich Protein (GLURP)	141024	223	6%
		124344	214	6%
		94874	174	5%
	Merozoite Surface Protein 1 (MSP-1)	189101	1031	17%
		75823	317	10%
Merozoites	Glutamate Rich Protein (GLURP)	124344	440	12%
		141024	406	8%
		94874	305	12%
	Rhoptry Neck Protein 4 (RON-4)	108332	231	6%

To corroborate these results, IP was repeated, and eluted fractions probed with antibodies against the two binding partners identified by MS. Anti-SURFIN_4.2_ antibodies also pulled down GLURP and RON4, as shown by immunoblots probed with specific antibodies against the two proteins. Both GLURP and RON4 migrated at anomalous sizes above 250KDa, with expected sizes for both proteins being around 135KDa (Fig 3C). This phenomenon has been previously reported and it is attributed to the skewed amino acid composition for both proteins, GLURP containing many repeats of negatively charged residues and RON4 having an extended N-terminal domain rich in proline and glutamic acid [18–20].

To reciprocate the above-mentioned results, reverse IP was also performed with anti-GLURP and anti-RON4 antibodies, and their ability to also pull-down SURFIN_4.2_ was tested by immunoblot. When IP was performed with anti-GLURP antibodies, only RON4 was also pulled-down (the detected band however, was relatively faint) but not SURFIN_4.2_. When IP was performed with anti-RON4 antibodies, both GLURP and SURFIN_4.2_ were also pulled-down (Fig 3D); the signal observed for GLURP was faint and more importantly appeared as a single band, contrasting with the multiple bands observed in the input fraction. These results corroborated the interacting partners suggested by the MS experiments, however it was puzzling to see that anti-GLURP antibodies were not able to also pull-down SURFIN_4.2_, while the opposite was observed with the initial experiments.

### SURFIN_4.2_ is localized in the neck of the rhoptries

**Fig 4:**
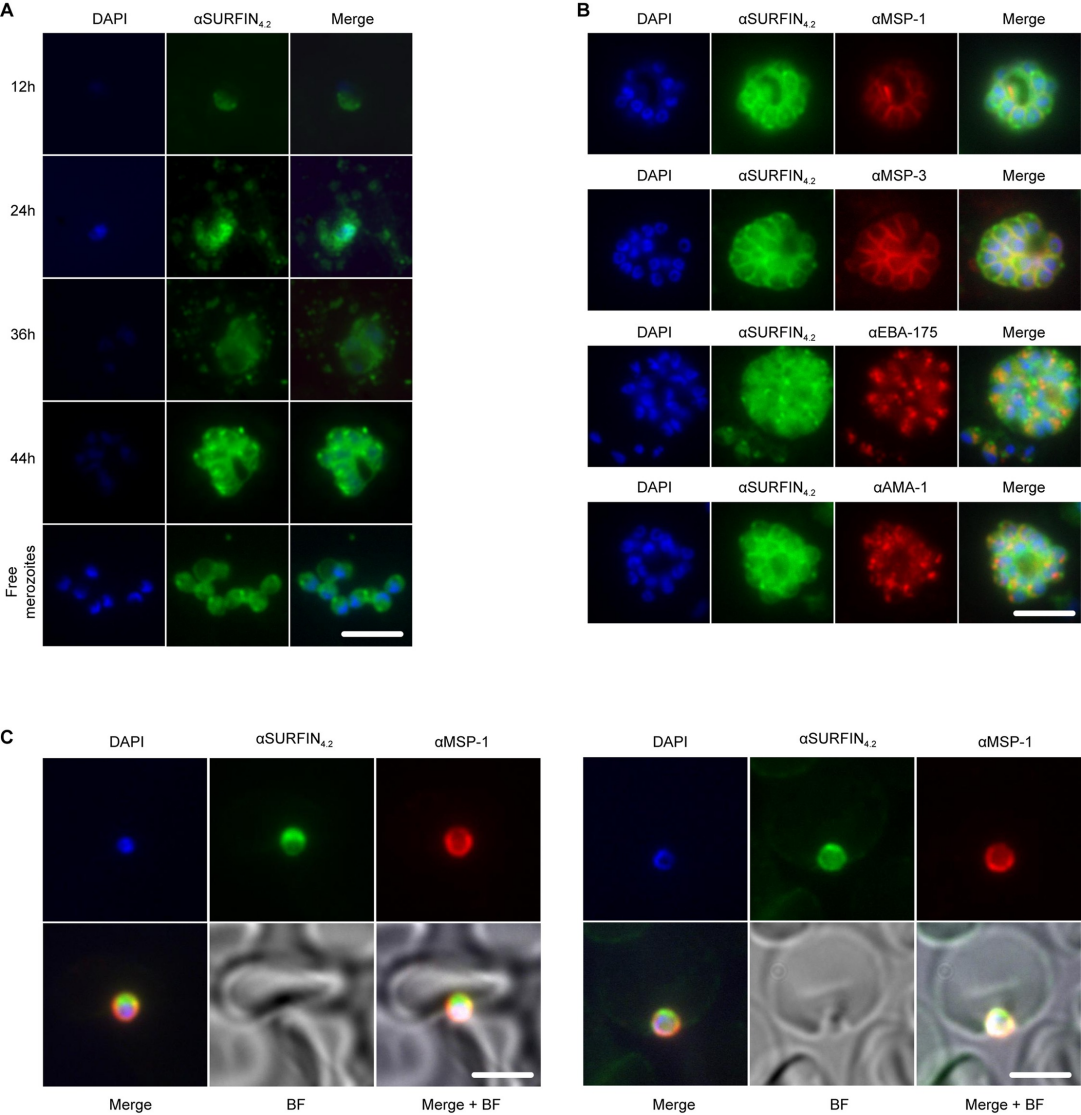
SURFIN_4.2_ localizes at the apical end and surface of the merozoites. (**A)** IFA on a time course, showing SURFIN_4.2_ labeling throughout the asexual cycle inside the RBCs and after schizont rupture. (**B)** IFA on double-labeled schizonts. (**C)** IFA on double-labeled invading merozoites upon apical attachments (upper panel) and during active invasion into the nascent PV (lower panel). SURFIN_4.2_ is shown in green, surface markers (MSP-1 and MSP-3) and microneme markers (EBA-175 and AMA-1) are shown in red. Scale bar represents 5μm.

To validate the previous reported localization of the SURFIN_4.2_ protein during the asexual cycle, parasite samples were collected every 12 hours and labeled with anti-SURFIN_4.2_ antibodies. Similarly as observed by immunoblot, the protein expression increased with cycle progression [14]. Signal was weak during ring stages (12 hours) and was mostly associated with the PV (Fig 4A) while trophozoite stages (24 and 36 hours), showed a stronger signal associated again with the PV as well as with vesicles (presumably MC) in the RBC cytoplasm (Fig 4A). Schizont stages showed a clear association between the forming merozoites and the SURFIN_4.2_ protein, staining was clearly confined to the apical end and the surface of the merozoites (Fig 4A). However, when free merozoites were studied, the merozoite surface staining was not as sharp as when they were still enclosed inside the PV at the schizont stage and this could be related with the observed protein shedding into the culture supernatant upon schizont rupture.

The specific subcellular localization of SURFIN_4.2_ at the apical end of the merozoite was confirmed using both immuno-fluorescence assays (IFA) and immuno-electron microscopy (iEM). Antibodies against SURFIN_4.2_ were used, as well as antibodies against the merozoite surface (MSP-1 and MSP-3) and the micronemes (EBA-175 and AMA-1). Co-labeling of schizonts with SURFIN_4.2_ and the merozoite surface markers MSP-1 and MSP-3 showed significant signal overlapping. Co-labeling with the microneme markers EBA-175 and AMA-1, showed the staining patterns were in close proximity, but no complete overlapping was observed (Fig 4B). Further, SURFIN_4.2_ labeling was localized more apically with respect to the microneme labeling. These observations suggested that SURFIN_4.2_ was localized both at the surface and at the apical end of the merozoite inside an organelle different from the micronemes.

The staining described above was performed on mature schizonts after fixation and permeabilization with Triton X-100, and free merozoites showed a less defined surface staining as compared with the enclosed merozoites, making the actual presence of the SURFIN_4.2_ at the surface questionable. To explore this further, accessibility of the antigen on purified merozoites was assessed in a similar fashion as above, including the fixation step but contrasting samples where a detergent (Triton X-100) permeabilization step was included or not. When co-labeling with surface and microneme markers was used, obvious differences were observed if fixed merozoites were permeabilized or not. In general, when merozoites were permeabilized, surface staining detected with SURFIN_4.2_, MSP-1 and MSP-3 antibodies was not clearly observed, indicating the treatment had shaved off the proteins exposed on the surface, while intracellular staining at the apical end detected with SURFIN_4.2_ and EBA-175 antibodies was still observed in a similar fashion as with the experiment described above (Panel A in S3 Fig). In contrast, when merozoites were not permeabilized, surface staining was clearly observed with the three antibodies used (SURFIN_4.2_, MSP-1 and MSP-3), while internal apical end staining was not observed (Panel B in S3 Fig), reassuring that SURFIN_4.2_ is exposed on the surface of free merozoites and is also inside an intracellular compartment (different from the micronemes) at the apical end.

When SURFIN_4.2_ localization was assessed during early events of the invasion process, protein was again observed at the surface and more importantly at the apical end of the merozoite. Protein was observed on the merozoite apical end during attachment to the RBC (Fig 4C, left panel) and during active invasion into the nascent PV (Fig 4C, right panel), suggesting a role of SURFIN_4.2_ during these early events.

**Fig 5:**
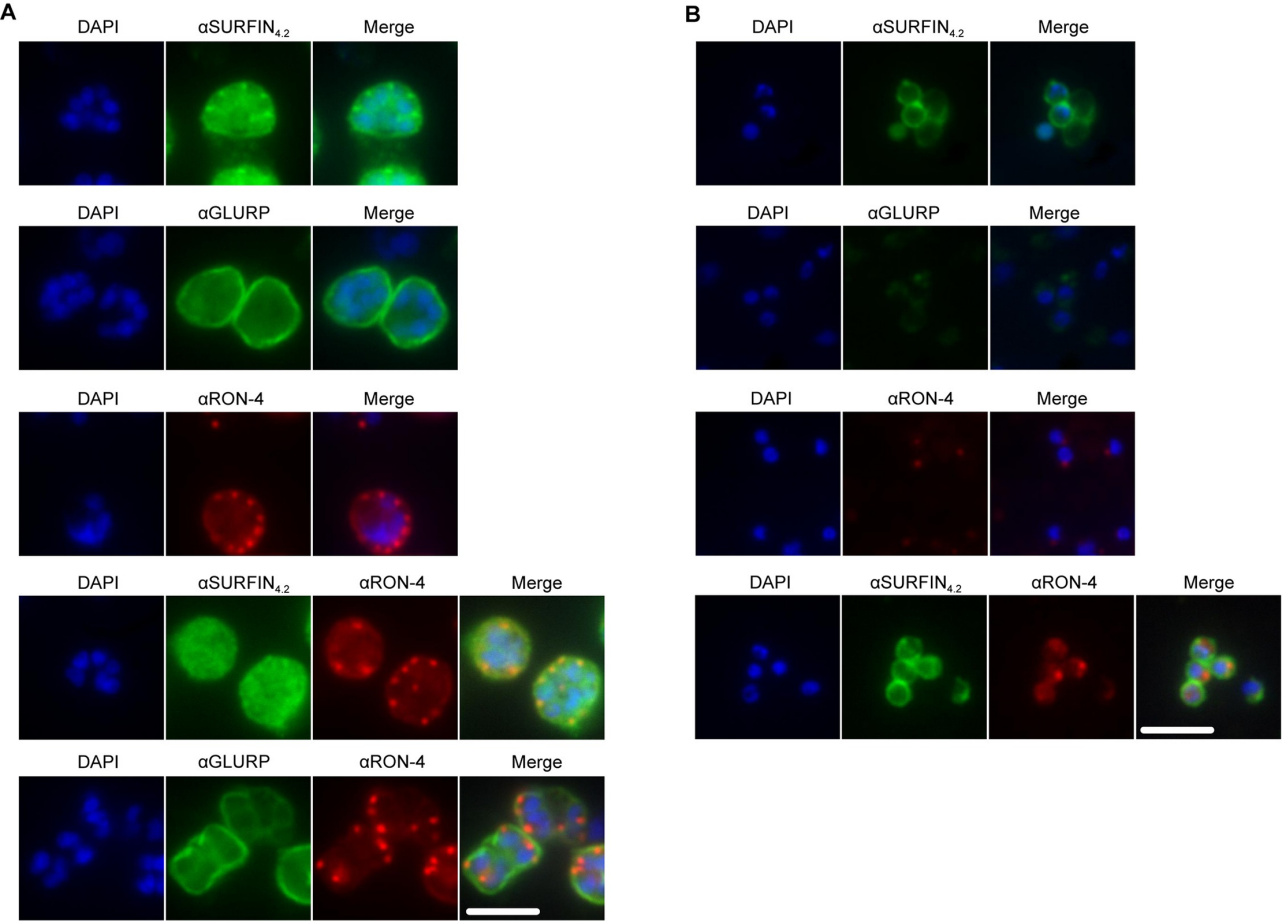
SURFIN_4.2_, GLURP and RON4 localization in schizonts and free merozoites. **(A)** IFA on single or double-labeled schizonts. (**B)** IFA on single or double-labeled free purified merozoites, SURFIN_4.2_ and GLURP are shown in green. RON4 is shown in red. Scale bar represents 5μm.

Co-localization analysis for SURFIN_4.2_ and its interacting partners RON4 and GLURP was also attempted. Available GLURP and SURFIN_4.2_ antibodies were both produced in rabbits, therefore co-localization experiments were not possible. Single staining with both antibodies however suggested that both SURFIN_4.2_ and GLURP are associated with the merozoite surface particularly in intact schizonts (Fig 5A and 5B, first two panels). Anti RON4 antibodies stained efficiently the merozoite apical end (rhoptries) if used alone (Fig 5A and 5B, third panel) but co-staining with SURFIN_4.2_ did not indicate a clear co-localization, with the apical localization for SURFIN_4.2_ not being observed when anti-RON4 antibodies were also included (Fig 5A and 5B, fourth panel). This could indicate that both proteins are in such close vicinity, that antibodies against one of them (anti-RON4) pose steric hindrance for antigen recognition by the second antibody (anti-SURFIN_4.2_).

**Fig 6:**
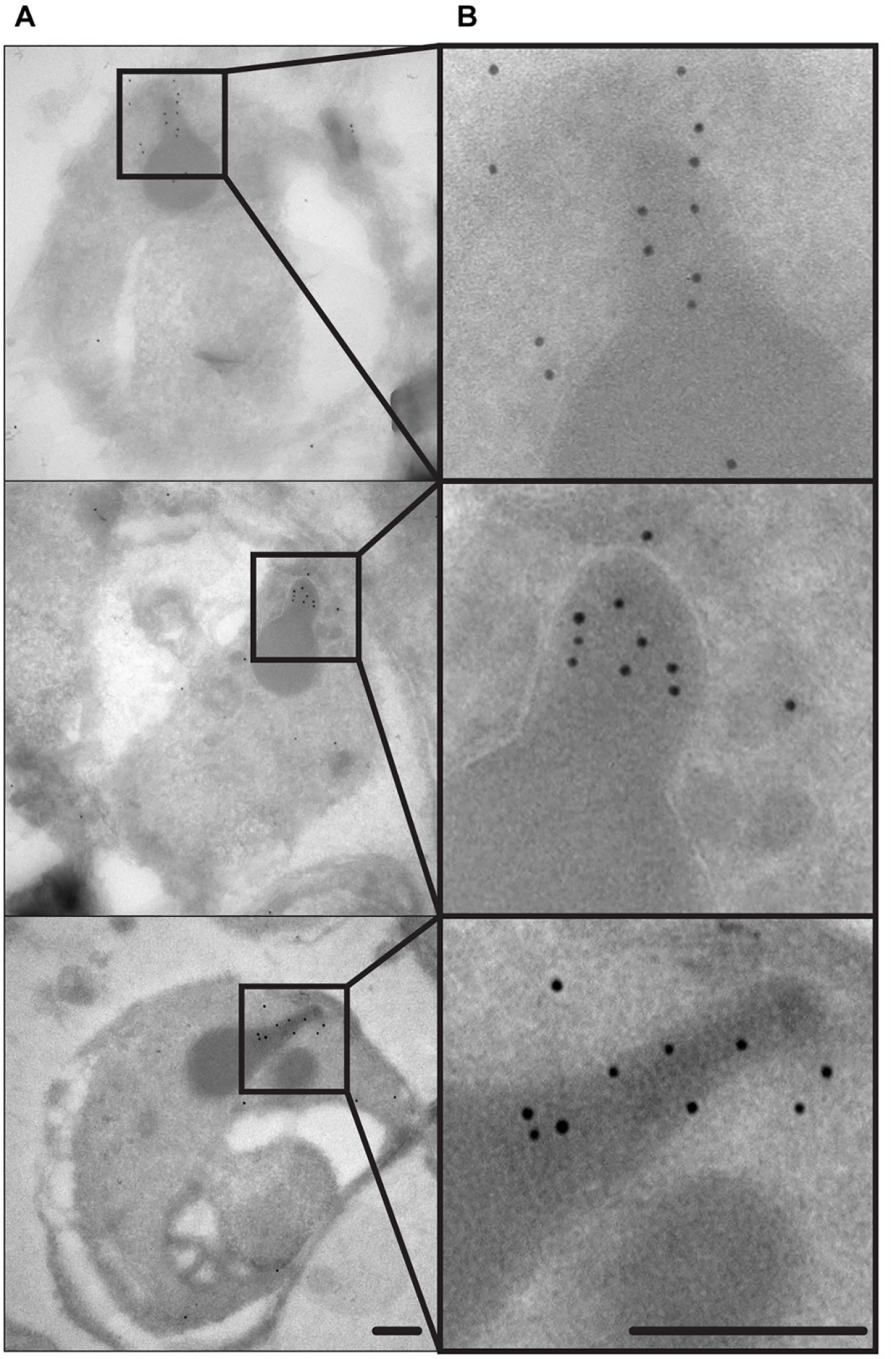
SURFIN_4.2_ localizes at the rhoptry neck. (**A)** Whole merozoites where the electrodense club-shaped rhoptries are clearly observed. (**B)** Image enlargements of the corresponding pictures shown in A. showing the rhoptries in more detail, SURFIN_4.2_ is clearly present in the most terminal part of the organelle: the neck. Scale bar represent 200nm.

Since the SURFIN_4.2_ labeling appeared to be more apical in relation to the micronemes, the most obvious candidate therefore was the rhoptries. These organelles are a pair of club-shaped apical structures with two distinct regions, the bulb (at the base of the organelle) and the neck (at the apex of the organelle), each region with a distinct protein composition [21]. iEM was chosen as an approach to determine the SURFIN_4.2_ subcellular localization, due to the clear and easy morphological identification of the rhoptries in EM. SURFIN_4.2_ was clearly observed at the more apical end of the rhoptries, the neck (Fig 6), consistent with the apical pattern observed by IFA. Interestingly, RON4 is also localized in the neck of the rhoptries [8], supporting the interaction hereby described with SURFIN_4.2_.

### SURFIN_4.2_ CRD mediates binding to RBCs

**Fig 7:**
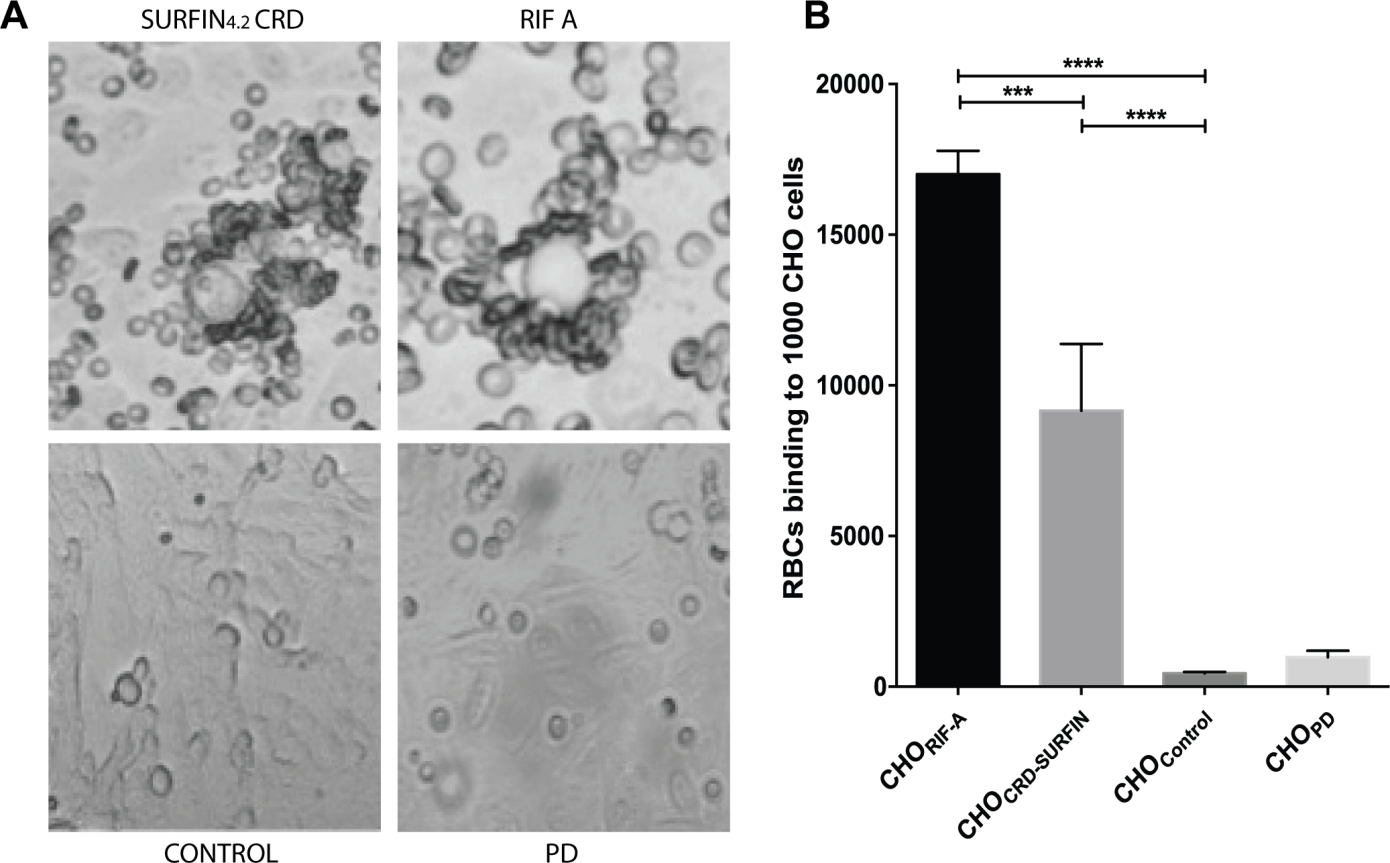
SURFIN_4.2_ CRD binds RBCs. (**A)** Bright-field images of group O RBCs binding to CHO cells expressing SURFIN_4.2_ CRD, RIFIN-A, untransfected (Control) or transfected with empty pDisplay plasmid (PD). (**B)** Number of RBCs binding to 1000 CHO cells, a positive control (RIF-A) known to bind RBCs was included. The values correspond to the mean±standard deviation (SD) of three independent experiments. Stars indicate significant difference.

The subcellular localization of SURFIN_4.2_ in the rhoptries and in the merozoite surface and its interaction with RON4, a well know player in the invasion process, strongly suggested a possible involvement of SURFIN_4.2_ in the initial binding of the merozoite to the RBC surface. To test this possibility the CRD segment of SURFIN_4.2_ was expressed on the surface of CHO cells and its ability to mediate binding to RBC was measured. Binding of RBCs was significant when the SURFIN_4.2_ CRD domain was expressed on the surface of CHO cells as compared with untransfected cells (control) or transfected with empty vector (PD). CHO cells expressing SURFIN4.2 bound abundant RBCs numbers (up to 15 RBCs per CHO cell) as observed in Fig 7A. The absolute number of RBCs bound to the CRD-transfected cells was lower than the one observed in the positive control (CHO cells transfected with a RIF-A) but was significantly higher than in the control groups (Fig 7B), indicating the CRD present in SURFIN_4.2_ is sufficient to mediate binding to the surface of the RBC.

### Antibodies against SURFIN_4.2_ partially inhibit invasion

In order to determine if SURFIN_4.2_ was required during the invasion of *P*. *falciparum*, the ability of anti-SURFIN_4.2_ antibodies to block parasite invasion was assessed. The growth inhibition assay (GIA) has been routinely used for this purpose [22–24], on despite of the fact that the assay could be measuring unspecific growth inhibitory effects not related with the invasion process.

**Fig 8:**
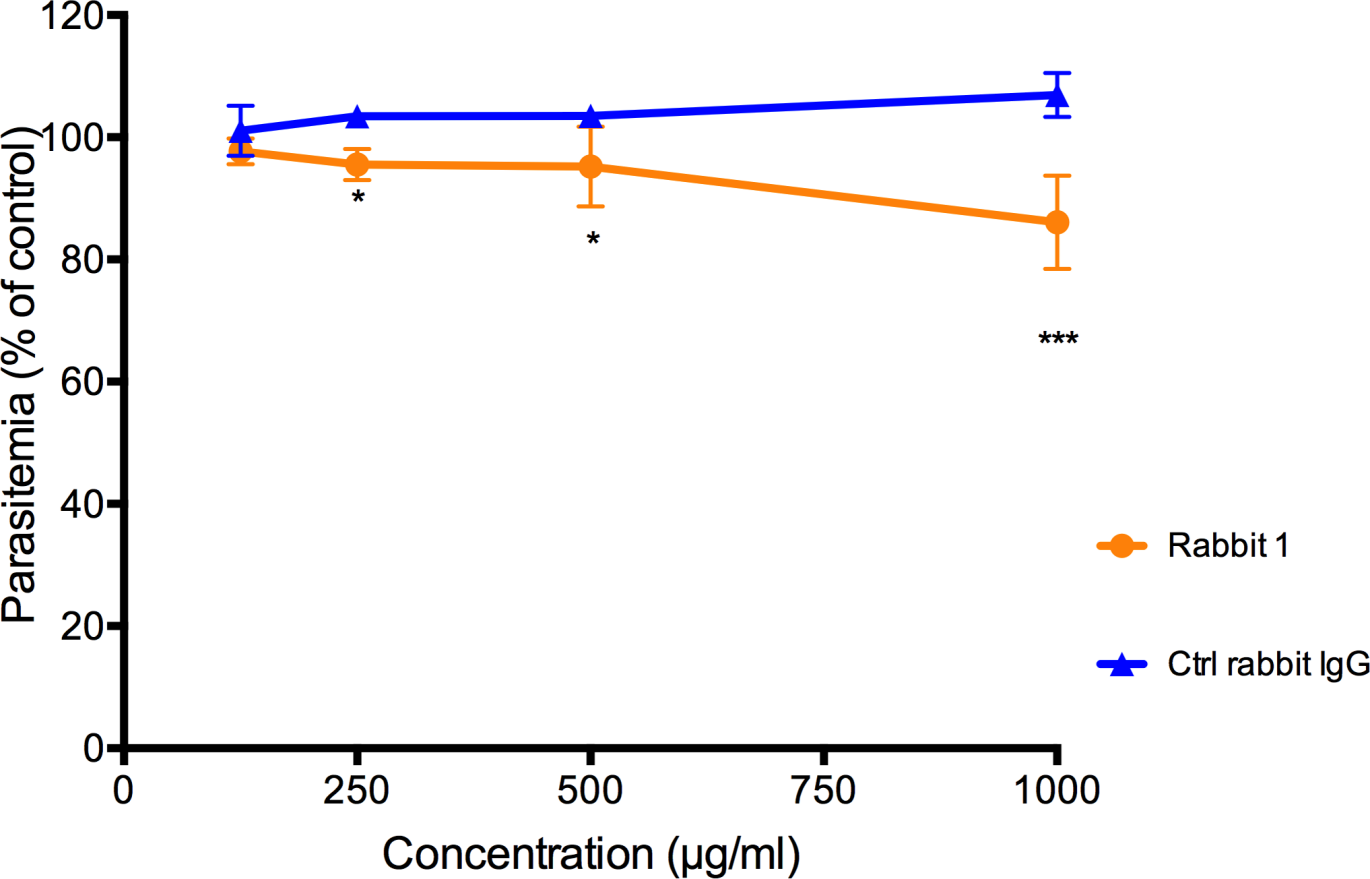
Antibodies against SURFIN_4.2_ partially inhibit invasion into RBCs by the parasite FCR3S1.2. Parasitaemia as percentage of a control (without antibody) is shown. The values correspond to the mean±standard deviation (SD) of three independent experiments. Stars indicate significant difference when compared with the rabbit IgG control.

Anti-SURFIN_4.2_ antibodies were tested at different concentrations and compared with control IgGs. Rabbit polyclonal antibodies seemed to inhibit growth in a concentration dependent manner, reaching a 20% inhibition at 1mg/ml compared with control IgGs at the same concentration (Fig 8) supporting a possible involvement of SURFIN_4.2_ during the invasion process and the early stages of parasite establishment inside the PV.

## Discussion

The invasion of *P*. *falciparum* into the RBCs is a complex process, requiring a precise succession of specific interactions between the host RBC surface protein and parasite ligands localized on the merozoite surface and/or consecutively discharged from the apical intracellular organelles (for a review see [2]). A recent video microscopy study [25] combined with the knowledge accumulated over years of research around the invasion process, has revealed the timing of particular morphological invasion steps and the correlation with particular ligand-receptor interactions.

*P*. *falciparum* is known to have the ability to use different and alternative invasion pathways, with many invasion related antigens being dispensable (for a review see [13]). Additionally, new merozoite surface proteins with RBC binding capacity or localized at the apical end and at the MJ are constantly being uncovered. Here we present yet another player at the surface and apex of the merozoite: SURFIN_4.2_. Although the protein was identified more than 10 years ago as a parasite antigen displayed on the surface of the pRBCs as well as associated with the merozoite apex [14], its function at both locations has been elusive.

To provide insights into the role of SURFIN_4.2_, particularly during the invasion process, we expressed and purified the predicted extracellular domain and raised specific antibodies against this region. The antibodies were specific and recognized the portion of the protein used for the immunization. More importantly they had minimal cross-reactivity with peptides belonging to all the other protein families included in the array. Cross-reactivity against conformational epitopes cannot be addressed using this approach and is also possible that the antibodies cross-react with other peptides not included in the array, however, considering the high-density of peptides (175000 different peptides) included and the marked specificity observed, we believe this possibility is unlikely. When tested in immunoblot these antibodies did not recognize RBC proteins (data not shown) but reacted with specific bands around the expected size (≈286KDa) for SURFIN_4.2_ when different time points during the parasite asexual cycle inside the RBCs were studied. The antibodies recognized several bands that were more apparent upon cycle progression, indicating a possible proteolytic processing of the protein to generate smaller fragments. While the role and the precise nature of the potential processing events were not assessed here, they could be important for the function of the protein. However, since multiple bands were observed early on during the parasite life cycle, it is unlikely to be related and/or essential for the invasion process. Processing of other proteins involved in the invasion process (e.g. MSP-1 and AMA-1) typically occurs right before or during active invasion [26–28]. Moreover, the fact that mostly partially processed and full-length protein is dominantly observed in culture supernatant upon schizont rupture further supports this assumption.

When protein localization was assessed during a time course, the protein was clearly observed at the surface and at the apical end of the merozoite, co-localizing with known surface markers (MSP-1 and MSP-3) while no co-localization was observed with microneme markers (EBA-175 and AMA-1). Interestingly the protein was also detected in the culture supernatant after schizont rupture and was easily cleaved from the merozoite surface with Triton X-100 (0.1%). Since SURFIN_4.2_ did not co-localize with microneme markers but was observed further along the apical end, the most obvious presumed localization was the rhoptries. Indeed SURFIN_4.2_ was localized in the neck of the rhoptries by iEM, and in some instances was observed to be in the process of being released from the merozoite into the extracellular space.

Surprisingly when protein extracts were tested by BN-PAGE, SURFIN_4.2_ was not only detected as a monomer bur also as part of protein complexes of higher molecular weight. This was more prominently observed when the detergent SB3-10 was used for the extract preparation. SB3-10 has been previously used to efficiently extract membrane proteins while retaining the integrity of membrane protein complexes [29]. To find out the identity of the proteins forming the complex, we performed IP with anti-SURFIN_4.2_ antibodies followed by MS of the pull-down (eluted) fractions. Two proteins were specifically detected both in schizont and merozoite preparations: GLURP and RON4. These results were validated detecting these proteins by immunoblot after SURFIN_4.2_ IP. We have named this new protein complex SURGE, based on the complex components (**SU**RFIN_4.2_-**R**ON4-**G**LURP compl**E**x).

When IP was performed using RON4 and GLURP antibodies, only the first was able to pull down SURFIN_4.2_, and both were able to pull down each other. These results might indicate that the antibody-binding site for the GLURP antibody is not accessible when the protein is part of SURGE. Another explanation is that the protein bulk for each protein is distributed in different states, as free protein, as a part of two-member complexes (SURFIN_4.2_-RON4, SURFIN_4.2_-GLURP, GLURP-RON4) and as part of SURGE.

GLURP was described almost 25 years ago, being observed at the PV during schizont stages and on the surface of newly released merozoites and was suggested to form part of a complex at this location [18]. The protein in a similar way as SURFIN_4.2_ and other invasion related proteins is shed during invasion, however antibodies against it do not inhibit invasion, but seem to induce antibody dependent cellular inhibition (ADCI) [30] and merozoite phagocytosis *in vitro* [31]. Moreover, naturally acquired antibodies against GLURP have been correlated with a reduced incidence of clinical malaria [32–34] attracting intense attention as a vaccine candidate, reaching phase 1 vaccine trials [35–38]. RON4, on the other hand, is a rhoptry neck protein central for MJ formation as part of the RON complex (formed by interactions with RON2, and 5). The complex is translocated to the RBC membrane and serves as interacting receptor for AMA-1 (via RON2) contributing to an essential step of the invasion process. The location of both proteins at the merozoite surface (GLURP) and at the rhoptries neck (RON4) suggest that proteins may come into contact with SURFIN_4.2_ while being translocated to the surface (SURFIN_4.2_-GLURP) and while localized at the neck of the rhoptries (SURFIN_4.2_-RON4), as well as when the three are shed and/or discharged during invasion.

Previous studies both in *Plasmodium* and *Toxoplasma* have shown that RON4 is present in considerable excess in relation to AMA-1 [20,39], therefore a significant amount of protein is likely available to interact with other proteins besides AMA-1. We speculate that the excess of RON4, free of AMA-1 interactions (through RON2) binds SURFIN_4.2_ and GLURP. Whether this interaction is cooperative to the one established with AMA-1 needs further investigation, but the fact that RON4 is consistently confined to the MJ indicates that any interacting partner is likely to be recruited to this site. Since RON4 together with RON5 (and RON8 in *Toxoplasma* MJ) are translocated by the parasite into the cytosolic side of the RBC membrane [40,41], it is possible that SURFIN_4.2_ and GLURP are similarly transported to this site. No orthologous gene for RON8 has been identified in *Plasmodium* and this protein in *Toxoplasma* is believed to mediate a strong attachment of the invading parasite to the host cell, probably anchoring it to common host cytoskeleton proteins [42]. We speculate that SURFIN_4.2_ could be performing a similar function through the WRDs in the C-terminus, which have similarities both with the PfEMP1 ATS (acidic terminal segment) as well as with a segment of Pf332 [14,16] both known to bind cytoskeleton components (indirectly and directly respectively) [43–45]. Recent evidence supports this idea, in fact showing that in fact SURFIN_4.2_ WRDs can bind RBC cytoskeleton components, including actin and spectrin [46].

Antibodies against SURFIN_4.2_ partially inhibited growth (20% growth inhibition in the presence of 1mg/ml of anti-SURFIN_4.2_ antibodies) further supporting the role of this protein during the invasion process. The inhibition observed was comparable to that observed for other antibodies against invasion related antigens, when total IgG at similar concentrations was used (e.g. AMA-1, Rh4, MSP-1 [47]). The fact that the gene encoding SURFIN_4.2_ has been successfully disrupted [48] however indicates the protein is not essential and its function is easily compensated.

In summary, here we described a novel protein complex involved in *P*. *falciparum* invasion. The complex is formed by the interactions between SURFIN_4.2_, GLURP and RON4 and is possibly localized at the MJ during the invasion process. We speculate the role of this complex mediates additional binding to receptors on the RBC surface providing stronger anchorage to the merozoite during active invasion and formation of the PV. This is supported by the fact that the CRD-domain of SURFIN_4.2_ when expressed on CHO-cell surface binds ample numbers of RBC (Fig 7).

## Materials and methods

### Parasite cultures

The *P*. *falciparum* FCR3S1.2 strain (a previously described FCR3-derived parasite [49]) was cultured according to standard methods. Red blood cells group O and 10% A^+^ non-immune Swedish serum were used, culture flasks were gassed with 90% NO_2_, 5% O_2_ and 5% CO_2_ and placed in a 37°C shaker incubator. Parasites were routinely synchronized at ring stage by sorbitol treatment [50] and the rosetting phenotype was maintained by enrichment over a Ficoll-cushion [51].

### Antibodies

Antibodies against the SURFIN_4.2_ were generated as described below (antibody production). Monoclonal antibody (mAb 24C6) against RON4 was a kind gift from Jean-Francois Dubremetz. Rabbit anti sera against GLURP region R2 was provided by Michael Theisen and produced as described before [52]. Anti EBA-175, AMA-1 antibodies were obtained through BEI Resources, MIAID, NIH. Antibodies against MSP-1 and MSP-3 were a kind gift of Virander Singh Chauhan.

For immunofluorescence detection the following secondary antibodies were used: Alexa Fluor 488 goat anti-rabbit IgG (A11008, Life Technologies), Alexa Fluor 594 goat anti-mouse IgG (A11032, Life technologies) at a 1:200 dilution.

For immunoblot detection the following secondary antibodies were used: anti-rabbit IgG HRP (NA934, GE Healthcare), anti-mouse IgG HRP (NXA931, GE Healthcare).

### Recombinant proteins

Expression of the SURFIN_4.2_ (PfIT_0422600) protein was performed as follows, the coding sequence from position 1–746 was commercially codon optimized for expression in bacteria (DNA2.0, Bioengineering Solutions) and cloned in house into the pDest527 vector (kind gift from Dominic Esposito, Addgene plasmid #11518) using the gateway cloning system (Thermo Fisher Scientific). The protein was expressed as an N-terminal 6x histidine-tagged recombinant protein in *E*. *coli* (BL21, New England Biolabs). Bacteria were grown at 37°C until they reached an OD_600_ = 0.6–0.8 and then induced with 0.25mM IPTG for 4 hours at 37°C. Pelleted bacteria were lysed by sonication and the recombinant protein was solubilized from washed IBs with denaturing solution (50mM Tris, 6M Guainidine HCl, 100 mM NaCl, 10mM DTT, 10 mM EDTA, pH 8) for 2 hours at room temperature. The protein was thereafter refolded by the rapid dilution method; 25 mg of protein were reduced with 10mM DTT for 1 hour at room temperature and the solution added drop wise into ice-cold refolding buffer (200mM Tris, 10mM EDTA, 0.6M Arginine, 6.5mM Cysteamine, 3.7 mM Cystamine, pH 8). Refolding was allowed to proceed for ≈24 hours at 4°C. The protein was then dialyzed against PBS and concentrated using Amicon Ultracel centrifugal filter units (Millipore).

Purification was performed over a Ni-NTA column (Qiagen) and eluted with increasing concentrations of imidazole. The purified protein was analyzed by sodium dodecyl sulfate polyacrylamide gel electrophoresis (SDS-PAGE) and immunoblot using an antibody against the poly-His tag (Qiagen).

### Antibody production

Polyclonal antibodies against the recombinant SURFIN_4.2_, were commercially produced by Agrisera (Vännäs, Sweden). Before immunization, a small sample of pre-immune sera was tested on parasite material–IFA on mixed staged parasite preparation and immunoblot- to rule out recognition of parasite proteins by antibodies present in the naïve animals; two rabbits were chosen, based on the absence of reactivity in both assays. Animals were immunized four times at one-month intervals with 200μg of protein emulsified in Freund’s complete for the first immunization and incomplete adjuvant for the following three immunizations. Final bleeding was carried out two weeks after the last immunization and the total IgG was purified on Protein G columns.

### Peptide array

Custom ultra-dense peptide microarrays obtained in collaboration with Roche-Nimblegen were applied for epitope mapping as described before [53]. An array containing 175,000 peptides of 12 amino acids in length and with an 11-residue overlap was designed to include several reported *P*. *falciparum* surface antigens, including the 2TM family, PHISTs, RIFINs, STEVORs, SURFINs and a handful PfEMP1s as reported in plasmodb. In all cases sequences annotated as pseudogenes were excluded. Duplicated protein sequences were deleted to reduce the total number of peptides. Anti-SURFIN_4.2_ antibodies were used and added to individual peptide arrays. Total IgG binding was detected using a DyLight 649-conjugated anti rabbit IgG (Jackson Immunoresearch) secondary antibody, and slide was scanned at 2μm resolution (MS200, Roche NimbleGen Inc., Madison, WI). Each spot on the array was subjected to pre-filtration criteria, as described previously [54], to define reactivity and also minimizing false positives: by requiring the spot MFI (mean fluorescence intensity) to be above two times the local spot background MFI and maximum 50% coefficient of variation within the spot. The number of reactive peptides per protein family was determined to assess the level of cross-reactivity.

### Merozoite purification

Merozoites were purified as described before [55]. Briefly, tightly synchronized parasites were purified on a magnetic column (Miltenyi Biotec) when they reached early segmented schizont stage. E64 protease inhibitor (E3132, Sigma) was added to the purified parasite at a final concentration of 10μM. Parasites were put back in culture for 5–8 hours till they completed segmentation and formed clear fully mature sacks of merozoites. E64-treated schizonts were centrifuged at 1900g for 5min and pellet re-suspended and filtered through a 1.2μm syringe filter (17593, Sartorius). Flow through after filtering was either used directly for imaging of the invasion process as described before [9] or re-centrifuged to obtain a merozoite pellet for protein extraction.

### Culture supernatant preparation

FCR3S1.2 parasite culture synchronized culture was grown till parasites reached early schizont stage followed by magnetic purification on a column (Miltenyi Biotec). The purified schizonts were allowed to grow and burst in the absence of human serum and RBCs and culture supernatants collected after two steps of centrifugation. First centrifugation was done at 3300g for 15 min at 4°C to remove schizonts and free merozoites. Supernatant was then centrifuged again in an Optima LE-80K ultracentrifuge at 48000rpm for 30 minutes at 4°C. Pellet, and supernatant before and after ultracentrifugation were analyzed on SDS-PAGE followed by immunoblot with anti-SURFIN_4.2_ antibodies as described below.

### Immunoblot analysis on *P*. *falciparum* material

Parasite protein extract was prepared as described before [56]. Briefly, total parasite culture was collected, culture medium removed and parasite pellet washed in PBS followed by treatment with 0.01% saponin solution in PBS. The suspension after lysis was centrifuged and the pellet further extracted in a 2% detergent solution plus protease inhibitors (Roche, Complete EDTA free tablets). The zwitterionic detergent SB3-10 (3-(Decyldimethylammonio) propanesulfonate inner salt) was routinely used after an initial detergent screening (S2 Fig) and also due to its reported efficiency to extract membrane associated proteins under non-denaturing conditions [29]. The ProteoPrep® Detergent Sample Kit was used (PROTDT, Sigma) and included, non-ionic (C13E10, DDM, OG, OTG and Triton X-100), zwitterionic (ASB-X4, CHAPS and SB3-10) and ionic (SDS) detergents, being of particular interest the first two categories, since they are considered, mild and intermediate detergents that preserve the native conformation of the proteins extracted, in contrast to ionic detergents. All the detergents were used at a concentration above the CMC (critical micelle concentration). The soluble fraction after detergent extraction was used to run both SDS-PAGE and BN-PAGE. During the initial screening the pellet was re-extracted in SDS 2% in PBS, to assess protein extraction efficiency into the soluble fraction. For the SDS-PAGE, 4–15% Tris-Glycine gels (Mini-PROTEAN®TGX, BioRad) were used; molecular weight was determined by comparison with a Multicolor High Range Protein ladder (26625, Thermo Scientific). For the BN-PAGE 3–12% Bis-Tris protein gels (NativePAGE™, Novex®) were used, molecular weight was determined by comparison with a Native standard marker (NativeMark™ Unstained Protein Standard Marker, Novex). After electrophoresis proteins were transferred to nitrocellulose or PVDF membranes (for SDS or BN respectively) using a wet transfer system. After transfer, membranes were blocked overnight at 4°C with gentle rocking in 1% blocking solution (Western Blocking Reagent, Roche) in TBS. Blocking solution was removed and primary antibodies diluted in 0.5% blocking solution in TBS were added and incubated for 1 hour at room temperature, followed by 3 washes with 0.1% Tween20 in TBS. Secondary antibodies diluted in 0.5% blocking solution in TBS were added and incubated for 1 hour at room temperature, followed by 3 washes with 0.1% Tween20 in TBS. Developing was performed using an ECL detection kit (RPN2105, GE Healthcare).

### Immunoprecipitation (IP) and mass spectrometry (MS)

For IP, parasite protein extract was prepared as described above (saponin followed by SB3-10 extraction). The Pierce Co-IP kit (26149, Thermo Fischer Scientific) was used following manufacturer’s instructions. In brief, anti-SURFIN_4.2_, anti-GLURP, anti-RON4 or control (non-immune IgG) antibodies were coupled to the kit’s agarose beads. Before adding the parasite extract to the antibody-coupled beads, detergent was removed using detergent removal spin columns (87779, Thermo Fischer Scientific) followed by a pre-clearing step of 1 hour at 4°C with control beads (antibody void agarose beads). Pre-cleared extracts were incubated with antibody-coupled beads overnight at 4°C followed by three washes and two steps of elution. Fractions after IP were ran on SDS-PAGE, transferred into nitrocellulose membrane and blotted as described above.

For MS, the elution fraction after IP with anti-SURFIN_4.2_ and control antibodies was briefly ran (to avoid protein separation and obtain small gel pieces) on SDS-PAGE, gel was stained with colloidal blue staining kit (LC6025, Invitrogen) and fragments where protein bands were observed were sent for MS protein identification to alphalyse (www.alphalyse.com). MS was performed according to alphalyse standard procedures. In brief, protein samples were reduced and alkylated with iodoacetamide and subsequently digested with trypsin. The resulting peptides were concentrated by Speed Vac lyophilization and re-dissolved for injection into a Dionex nano-LC system and MS/MS analysis on a Bruker Maxis Impact QTOF instrument. The MS/MS spectra were used for Mascot database searching against human and parasite databases downloaded from UniProt and NCBI. The protein identification was based on a probability-scoring algorithm (www.matrixscience.com). It was considered as positive identification when at least 2 peptides had an Ions score above 35 or if a protein under 20KDa had 1 peptide with an Ions score above 50. The sequence coverage was not considered for the identification and the total Mascot score provided for each identification is a total of all the individual peptide Mascot scores.

### Immunofluorescence assay (IFA)

Parasites were collected, culture medium was removed and cell pellet washed three times with PBS. Microscope slides (HTC, 30–565, 15 well, 4mm, Thermo Scientific) were treated with 0.1% Poly-L-Lysine (P8920, Sigma) and after a wash with PBS cells suspension was added and incubated for 30 minutes in a humidified chamber. Cells were fixed for 30 minutes in 3% paraformaldehyde in PBS, followed by a 10 minute permeabilization step in 0.1% Triton X-100 and 2%BSA solution in PBS. Bound cells were blocked overnight at 4°C in a 2% BSA and 0.3M Glycine solution in PBS. Blocking solution was removed and primary antibodies diluted in 2%BSA solution in PBS were added and incubated for 1 hour at room temperature, followed by 3 washes with PBS. Secondary antibodies diluted in 2%BSA solution in PBS were added and incubated for 1 hour at room temperature, followed by 3 washes with PBS and a few drops of Vectashield mounting media with DAPI (H-1200, Vector Laboratories) were added followed by sealing with a coverslip.

### Immunoelectron microscopy (iEM)

Parasites were fixed in 3% paraformaldehyde in 0.1 M phosphate buffer. After fixation cells were washed in 0.1M PBS and embedded in 10% gelatin. Samples were then infiltrated into 2.3 M of sucrose and frozen in liquid nitrogen. Sectioning was performed at -95°C and placed on carbon-reinforced formvar-coated, 50 mesh Nickel grids. Immuno-labelling procedure was performed as follows: grids were placed directly on drops of 2% BSA (Sigma fraction V) and 2% Fish gelatin (GE Healthcare, Buckinghampshire, UK) in 0.1 M phosphate buffer to block non-specific binding. Sections were then incubated with the primary antibody diluted 1:400 in 0.1M of phosphate buffer containing 0.1% BSA + 0.1% Gelatin over the night in a humidified chamber at room temperature. The sections were thoroughly washed in the same buffer and bound antibodies were detected with protein A coated with 10 nm gold (Biocell, BBInternational, Cardiff, England) at a final dilution of 1:100. Sections were rinsed in buffer and fixed in 2% glutaraldehyde contrasted with 0,05% uranyl acetate and embedded in 1% methylcellulose. Preparations were examined in a Hitachi 7700 (Tokuyo, Japan) at 80 kV. Digital images were taken with a Veleta camera (Soft Imaging System GmbH, Muenster, Germany).

### Cloning and expression of SURFIN_4.2_ in CHO cells

Sequences encoding the SURFIN_4.2_ CRD (PfIT_0422600) and an A-RIFIN (PF3D7_0100400) were PCR amplified and cloned into the pDisplay vector (Invitrogen, USA) between the BglII and SalI sites. Proteins expressed from the pDisplay vector were directed to the cellular secretory pathway by the fusion of the protein N terminus to a mouse Ig κ-chain leader sequence. More importantly, the proteins were anchored to the cell membrane by the inclusion at the C terminus of a platelet-derived growth factor receptor (PDGFR) transmembrane domain, displaying the protein of interest on the extracellular side of the membrane. The pDisplay constructs also included a hemagglutinin A tag (HA) at the N terminus and a myc tag at the C terminus (right before the PDGFR transmembrane segment). CHO cells (pgsA-745, purchased from American Type Culture Collection) were transfected with the respective pDisplay constructs using Fugene® 6 (Roche biosciences, Switzerland) following the manufacturer’s instructions. Briefly, 6μl of Fugene were added into serum free culture medium (OPTIMEM) and incubated at room temperature for 5 min. DNA (1μg) was then added to the Fugene-medium mixture and after incubation at room temperature for 30 min, the mixture was added to the CHO cells until drug selection. After 48 hours, the transfection media was removed and successfully transfected cells were selected by the addition of G418 at 1 mg/ml. The surface expression of the proteins was verified by FACS using antibodies against the HA (N terminus) and myc (C terminus) tags.

### RBC binding to CHO cells

Transfected (with SURFIN_4.2_ CRD or RIFIN-A known to bind RBCs) or control cells (untransfected controls or transfected with empty pDisplay vector–PD-) were seeded in 8-well Nunc Lab-Tek chamber slides overnight and grown until they were 40–50% confluent. Blood group O RBCs were washed twice in PBS and added to the cells at 2% hematocrit in complete media. The cells were incubated with erythrocytes at 37°C in a CO_2_ incubator for 1 hour and subsequently washed three times with RPMI to remove unbound RBCs. Binding was measured as the number of RBCs bound to 1000 CHO cells, randomly selected across a slide.

### Growth inhibition assay (GIA)

Synchronized parasite were grown till they reached trophozoite stage, then a suspension at 0.5% parasitaemia and 5% hematocrit was prepared in the presence of anti-SURFIN_4.2_ or control antibody solution at different concentrations. 100μl of the suspension were dispensed in triplicate on a 96 well U-bottom plate (Falcon, 351177) and incubated in a gassed humidified chamber at 37°C for around 30 hours; after this time parasites were expected to had reinvaded and to be at late ring stage. 100μl of acridine orange solution (1μg/ml in PBS) was added and incubated in the dark for 5 minutes, followed by one wash in PBS. Final parasitaemia was measured using a FACSVerse flow cytometer (BD Biosciences) acquiring 100000 events (doublets excluded). Parasitaemia was calculated, as a percentage of the one obtained with a control, were PBS was added instead of antibody.

### Analysis

Flow cytometry analysis was performed using the FlowJo 10.0.7 software (TreeStar, USA). Mean, standard deviations (SD) and figures were prepared using the GraphPad Prism version 6.0f for Mac OS X (La Jolla, California, USA). When relevant, an alpha level of 0.05 was used to determine statistical significance.

### Ethical statement

Animal immunizations were carried out commercially by Agrisera (Vännas, Sweden) approved by Ethical Review Board (Jordbruksverket, permits A72-11, A6-13). Animal handling adhered to the national regulations including the Animal Welfare Ordinance and the Animal Welfare Act (SJVFS 2012:26) that rule on questions regarding animal research.

## Supporting information

S1 Table Peptide array summary. (XLSX)

S1 Fig Recombinant protein expression and purification. (**A)** Coomassie stained gel of the expressed extracellular domain of SURFIN_4.2_ in *E*. *coli*. (**B)** Coomassie stained gel of the purified extracellular domain of SURFIN_4.2_ in *E*. *coli*. (**C)** Coomassie stained gel and corresponding anti-His immunoblot showing the purified and concentrated protein. Solid arrows indicate the expected size of the full fragment expressed and dashed arrows indicate dimer size bands. P: Pellet; S: Supernatant; FT: Flow-through; W: Wash; E1: Elute 1 (50mM imidazole); E2: Elute 2 (100mM imidazole); E3: Elute 3 (150 mM imidazole); β-ME: 2-Mercaptoethanol.
(TIF)

S2 Fig Detergent screening for protein extraction from parasite material. (**A)** SDS-PAGE for 10 different detergents tested, depicting both pellet (P) and supernatant (S) to check the detergent extraction efficiency into the supernatant fraction. (**B)** BN-PAGE for 9 of the detergent used, only supernatants were used here. Different colors for detergent labels indicate: ionic detergent in black, non-ionic detergents in red and zwitterionic detergent in blue. Both panel were probed with αSURFIN_4.2_.
ASB-14: 3-[N,N-Dimethyl(3-myristoylaminopropyl)ammonio]propanesulfonate, Amidosulfobetaine-14, C7
C7BzO: 3-(4-Heptyl)phenyl-3-hydroxypropyl)dimethylammoniopropanesulfona
C13E10: Polyoxyethylene (10) tridecyl ether (mixture of C11 to C14 iso-alkyl ethers with C13 iso-alkyl predominating)
CHAPS: 3-[(3-Cholamidopropyl)dimethylammonio]-1-propanesulfonate hydrate
DDM: n-Dodecyl β-D-maltoside
OG: Octyl β-D-glucopyranoside
OTG: Octyl β-D-1-thioglucopyranoside
SB3-10: 3-(Decyldimethylammonio)­propane­sulfonate inner salt
TX-100: Triton X-100
SDS: Sodium dodecyl sulfate
(TIF)

S3 Fig IFA on double-labeled free purified merozoites with **(A)** and without **(B)** a permeabilization step with Triton X-100. SURFIN_4.2_ is shown in green, surface markers (MSP-1 and MSP-3) and microneme markers (EBA-175 and AMA-1) are shown in red. Scale bar represents 5μm.
(TIF)

S4 Fig Uncropped western blot images, dashed boxes represent portions of the image used to build the figures presented in the manuscript. **(A, B and C)** Uncropped images used in Fig 2. **(D, E, F and G)** Uncropped images used in Fig 3. In panel D and E, blue boxes correspond to IP with control IgG and red boxes correspond to IP with αSURFIN_4.2_.
(PDF)

S1 Text Mass spectrometry reports. Samples 4 and 9 correspond to the eluted fractions after IP with αSURFIN_4.2_ from schizont and merozoite material respectively.
(PDF)

## References

[1] WHO (World Health Organization). World Malaria Report 2017. 2017.

[2] Weiss GE, Crabb BS, Gilson PR. Overlaying Molecular and Temporal Aspects of Malaria Parasite Invasion. Trends Parasitol. 2016 4;32(4):284–95. 10.1016/j.pt.2015.12.007 26778295

[3] Beeson JG, Drew DR, Boyle MJ, Feng G, Fowkes FJI, Richards JS. Merozoite surface proteins in red blood cell invasion, immunity and vaccines against malaria. Vol. 40, FEMS Microbiology Reviews. 2016 p. 343–72. 10.1093/femsre/fuw001 26833236 PMC4852283

[4] Kauth CW, Epp C, Bujard H, Lutz R. The merozoite surface protein 1 complex of human malaria parasite Plasmodium falciparum: Interactions and arrangements of subunits. J Biol Chem. 2003;278(25):22257–64. 10.1074/jbc.M302299200 12654909

[5] Kadekoppala M, Holder AA. Merozoite surface proteins of the malaria parasite: The MSP1 complex and the MSP7 family. Int J Parasitol. 2010;40(10):1155–61. 10.1016/j.ijpara.2010.04.008 20451527

[6] Lin CS, Uboldi AD, Epp C, Bujard H, Tsuboi T, Czabotar PE, et al Multiple plasmodium falciparum merozoite surface protein 1 complexes mediate merozoite binding to human erythrocytes. J Biol Chem. 2016;291(14):7703–15. 10.1074/jbc.M115.698282 26823464 PMC4817195

[7] Cao J, Kaneko O, Thongkukiatkul A, Tachibana M, Otsuki H, Gao Q, et al Rhoptry neck protein RON2 forms a complex with microneme protein AMA1 in Plasmodium falciparum merozoites. Parasitol Int. 2009;58(1):29–35. 10.1016/j.parint.2008.09.005 18952195

[8] Richard D, MacRaild C a, Riglar DT, Chan J-A, Foley M, Baum J, et al Interaction between Plasmodium falciparum apical membrane antigen 1 and the rhoptry neck protein complex defines a key step in the erythrocyte invasion process of malaria parasites. J Biol Chem. 2010 5 7;285(19):14815–22. 10.1074/jbc.M109.080770 20228060 PMC2863225

[9] Riglar DT, Richard D, Wilson DW, Boyle MJ, Dekiwadia C, Turnbull L, et al Super-resolution dissection of coordinated events during malaria parasite invasion of the human erythrocyte. Cell Host Microbe. 2011;9(1):9–20. 10.1016/j.chom.2010.12.003 21238943

[10] Galaway F, Drought LG, Fala M, Cross N, Kemp AC, Rayner JC, et al P113 is a merozoite surface protein that binds the N terminus of Plasmodium falciparum RH5. Nat Commun. 2017;8. 10.1038/ncomms14333 PMC5309799 28186186

[11] Reddy KS, Amlabu E, Pandey AK, Mitra P, Chauhan VS, Gaur D. Multiprotein complex between the GPI-anchored CyRPA with PfRH5 and PfRipr is crucial for Plasmodium falciparum erythrocyte invasion. Proc Natl Acad Sci. 2015 1 12;112(4):201415466. 10.1073/pnas.1415466112 PMC4313826 25583518

[12] Chen L, Lopaticki S, Riglar DT, Dekiwadia C, Uboldi AD, Tham W-H, et al An EGF-like protein forms a complex with PfRh5 and is required for invasion of human erythrocytes by Plasmodium falciparum. PLoS Pathog. 2011 9;7(9):e1002199 10.1371/journal.ppat.1002199 21909261 PMC3164636

[13] Cowman AF, Berry D, Baum J. The cellular and molecular basis for malaria parasite invasion of the human red blood cell. J Cell Biol. 2012 9 17;198(6):961–71. 10.1083/jcb.201206112 22986493 PMC3444787

[14] Winter G, Kawai S, Haeggström M, Kaneko O, von Euler A, Kawazu S, et al SURFIN is a polymorphic antigen expressed on Plasmodium falciparum merozoites and infected erythrocytes. J Exp Med. 2005;201(11):1853–63. 10.1084/jem.20041392 15939796 PMC2213267

[15] Mphande FA, Ribacke U, Kaneko O, Kironde F, Winter G, Wahlgren M. SURFIN4.1, a schizont-merozoite associated protein in the SURFIN family of Plasmodium falciparum. Malar J. 2008;7(1):116. 18593471 10.1186/1475-2875-7-116 PMC2515329

[16] Frech C, Chen N. Variant surface antigens of malaria parasites: functional and evolutionary insights from comparative gene family classification and analysis. BMC Genomics. 2013 1;14(1):427. 23805789 10.1186/1471-2164-14-427 PMC3747859

[17] Wickham ME, Culvenor JG, Cowman AF. Selective inhibition of a two-step egress of malaria parasites from the host erythrocyte. J Biol Chem. 2003 9 26;278(39):37658–63. 10.1074/jbc.M305252200 12857731

[18] Borre MB, Dziegiel M, Høgh B, Petersen E, Rieneck K, Riley E, et al Primary structure and localization of a conserved immunogenic Plasmodium falciparum glutamate rich protein (GLURP) expressed in both the preerythrocytic and erythrocytic stages of the vertebrate life cycle. Mol Biochem Parasitol. 1991;49(1):119–31. 1775153 10.1016/0166-6851(91)90135-s

[19] Theisen M, Cox G, Hogh B, Jepsen S, Vuust J. Immunogenicity of the Plasmodium falciparum glutamate-rich protein expressed by vaccinia virus. Infect Immun. 1994;62(8):3270–5. 8039897 10.1128/iai.62.8.3270-3275.1994 PMC302955

[20] Alexander DL, Arastu-Kapur S, Dubremetz JF, Boothroyd JC. Plasmodium falciparum AMA1 binds a rhoptry neck protein homologous to TgRON4, a component of the moving junction in Toxoplasma gondii. Eukaryot Cell. 2006;5(7):1169–73. 10.1128/EC.00040-06 16835460 PMC1489286

[21] Counihan NA, Kalanon M, Coppel RL, De Koning-Ward TF. Plasmodium rhoptry proteins: Why order is important. Trends Parasitol. 2013;29(5):228–36. 10.1016/j.pt.2013.03.003 23570755

[22] Persson KEM, Lee CT, Marsh K, Beeson JG. Development and optimization of high-throughput methods to measure Plasmodium falciparum-specific growth inhibitory antibodies. J Clin Microbiol. 2006;44(5):1665–73. 10.1128/JCM.44.5.1665-1673.2006 16672391 PMC1479166

[23] Bergmann-Leitner ES, Duncan EH, Mullen GE, Burge JR, Khan F, Long CA, et al Critical evaluation of different methods for measuring the functional activity of antibodies against malaria blood stage antigens. Am J Trop Med Hyg. 2006;75(3):437–42. 16968918

[24] McCallum FJ, Persson KEM, Mugyenyi CK, Fowkes FJI, Simpson JA, Richards JS, et al Acquisition of growth-inhibitory antibodies against blood-stage Plasmodium falciparum. PLoS One. 2008;3(10). 10.1371/journal.pone.0003571 PMC2570221 18958278

[25] Weiss GE, Gilson PR, Taechalertpaisarn T, Tham WH, de Jong NWM, Harvey KL, et al Revealing the Sequence and Resulting Cellular Morphology of Receptor-Ligand Interactions during Plasmodium falciparum Invasion of Erythrocytes. PLoS Pathog. 2015;11(2). 10.1371/journal.ppat.1004670 PMC4344246 25723550

[26] Fleck SL, Birdsall B, Babon J, Dluzewski AR, Martin SR, Morgan WD, et al Suramin and Suramin Analogues Inhibit Merozoite Surface Protein-1 Secondary Processing and Erythrocyte Invasion by the Malaria Parasite Plasmodium falciparum. J Biol Chem. 2003;278(48):47670–7. 10.1074/jbc.M306603200 13679371

[27] Dutta S, Haynes JD, Barbosa A, Ware LA, Snavely JD, Moch JK, et al Mode of action of invasion-inhibitory antibodies directed against apical membrane antigen 1 of Plasmodium falciparum. Infect Immun. 2005;73(4):2116–22. 10.1128/IAI.73.4.2116-2122.2005 15784553 PMC1087451

[28] Moss DK, Remarque EJ, Faber BW, Cavanagh DR, Arnot DE, Thomas AW, et al Plasmodium falciparum 19-kilodalton merozoite surface protein 1 (MSP1)-specific antibodies that interfere with parasite growth in vitro can inhibit MSP1 processing, merozoite invasion, and intracellular parasite development. Infect Immun. 2012 3;80(3):1280–7. 10.1128/IAI.05887-11 22202121 PMC3294643

[29] Everberg H, Leiding T, Schiöth A, Tjerneld F, Gustavsson N. Efficient and non-denaturing membrane solubilization combined with enrichment of membrane protein complexes by detergent/polymer aqueous two-phase partitioning for proteome analysis. J Chromatogr A. 2006 7 28;1122(1–2):35–46. 10.1016/j.chroma.2006.04.020 16682048

[30] Theisen M, Soe S, Oeuvray C, Thomas AW, Vuust J, Danielsen S, et al The glutamate-rich protein (GLURP) of Plasmodium falciparum is a target for antibody-dependent monocyte-mediated inhibition of parasite growth in vitro. Infect Immun. 1998;66(1):11–7. 9423833 10.1128/iai.66.1.11-17.1998 PMC107852

[31] Kana IH, Adu B, Tiendrebeogo RW, Singh SK, Dodoo D, Theisen M. Naturally acquired antibodies target the glutamate-rich protein on intact merozoites and predict protection against febrile malaria. J Infect Dis. 2017;215(4):623–30. 10.1093/infdis/jiw617 28329101

[32] Dodoo D, Aikins A, Kusi KA, Lamptey H, Remarque E, Milligan P, et al Cohort study of the association of antibody levels to AMA1, MSP119, MSP3 and GLURP with protection from clinical malaria in Ghanaian children. Malar J. 2008;7(1):142. 18664257 10.1186/1475-2875-7-142 PMC2529305

[33] Meraldi V, Nebié I, Tiono AB, Diallo D, Sanogo E, Theisen M, et al Natural antibody response to Plasmodium falciparum Exp-1, MSP-3 and GLURP long synthetic peptides and association with protection. Parasite Immunol. 2004;26(6–7):265–72. 10.1111/j.0141-9838.2004.00705.x 15541030

[34] Adu B, Cherif MK, Bosomprah S, Diarra A, Arthur FKN, Dickson EK, et al Antibody levels against GLURP R2, MSP1 block 2 hybrid and AS202.11 and the risk of malaria in children living in hyperendemic (Burkina Faso) and hypo-endemic (Ghana) areas. Malar J. 2016;15(1):123. 26921176 10.1186/s12936-016-1146-4 PMC4769494

[35] Hermsen CC, Verhage DF, Telgt DSC, Teelen K, Bousema JT, Roestenberg M, et al Glutamate-rich protein (GLURP) induces antibodies that inhibit in vitro growth of Plasmodium falciparum in a phase 1 malaria vaccine trial. Vaccine. 2007;25(15):2930–40. 10.1016/j.vaccine.2006.06.081 16914240

[36] Esen M, Kremsner PG, Schleucher R, Gässler M, Imoukhuede EB, Imbault N, et al Safety and immunogenicity of GMZ2—a MSP3-GLURP fusion protein malaria vaccine candidate. Vaccine. 2009;27(49):6862–8. 10.1016/j.vaccine.2009.09.011 19755144

[37] Belard S, Issifou S, Hounkpatin AB, Schaumburg F, Ngoa UA, Esen M, et al A randomized controlled phase IB trial of the malaria vaccine candidate GMZ2 in african children. PLoS One. 2011;6(7):1–8. 10.1371/journal.pone.0022525 PMC3145647 21829466

[38] Jepsen MPG, Jogdand PS, Singh SK, Esen M, Christiansen M, Issifou S, et al The malaria vaccine candidate GMZ2 elicits functional antibodies in individuals from malaria endemic and non-endemic areas. J Infect Dis. 2013;208(3):479–88. 10.1093/infdis/jit185 23624363

[39] Alexander DL, Mital J, Ward GE, Bradley P, Boothroyd JC. Identification of the moving junction complex of Toxoplasma gondii: A collaboration between distinct secretory organelles. PLoS Pathog. 2005;1(2):0137–49. 10.1371/journal.ppat.0010017 PMC1262624 16244709

[40] Besteiro S, Michelin A, Poncet J, Dubremetz JF, Lebrun M. Export of a Toxoplasma gondii rhoptry neck protein complex at the host cell membrane to form the moving junction during invasion. PLoS Pathog. 2009;5(2). 10.1371/journal.ppat.1000309 PMC2642630 19247437

[41] Srinivasan P, Beatty WL, Diouf A, Herrera R, Ambroggio X, Moch JK, et al Binding of Plasmodium merozoite proteins RON2 and AMA1 triggers commitment to invasion. Proc Natl Acad Sci U S A. 2011;108(32):13275–80. 10.1073/pnas.1110303108 21788485 PMC3156155

[42] Straub KW, Peng ED, Hajagos BE, Tyler JS, Bradley PJ. The moving junction protein RON8 facilitates firm attachment and host cell invasion in Toxoplasma gondii. PLoS Pathog. 2011;7(3). 10.1371/journal.ppat.1002007 PMC3053350 21423671

[43] Oh SS, Voigt S, Fisher D, Yi SJ, LeRoy PJ, Derick LH, et al Plasmodium falciparum erythrocyte membrane protein 1 is anchored to the actin-spectrin junction and knob-associated histidine-rich protein in the erythrocyte skeleton. Mol Biochem Parasitol. 2000 5;108(2):237–47. 10838226 10.1016/s0166-6851(00)00227-9

[44] Waller KL, Stubberfield LM, Dubljevic V, Buckingham DW, Mohandas N, Coppel RL, et al Interaction of the exported malaria protein Pf332 with the red blood cell membrane skeleton. Biochim Biophys Acta—Biomembr. 2010;1798(5):861–71. 10.1016/j.bbamem.2010.01.018 PMC4768738 20132790

[45] Mayer C, Slater L, Erat MC, Konrat R, Vakonakis I. Structural analysis of the Plasmodium falciparum erythrocyte membrane protein 1 (PfEMP1) intracellular domain reveals a conserved interaction epitope. J Biol Chem. 2012 3 2;287(10):7182–9. 10.1074/jbc.M111.330779 22249178 PMC3293552

[46] Zhu X, He Y, Liang Y, Kaneko O, Cui L, Cao Y. Tryptophan ‑ rich domains of Plasmodium falciparum SURFIN4.2 and Plasmodium vivax PvSTP2 interact with membrane skeleton of red blood cell. Malar J. 2017;1–12. 10.1186/s12936-016-1650-6 28320404 PMC5359885

[47] Williams AR, Douglas AD, Miura K, Illingworth JJ, Choudhary P, Murungi LM, et al Enhancing Blockade of Plasmodium falciparum Erythrocyte Invasion: Assessing Combinations of Antibodies against PfRH5 and Other Merozoite Antigens. PLoS Pathog. 2012;8(11). 10.1371/journal.ppat.1002991 PMC3493472 23144611

[48] Maier AG, Rug M, O’Neill MT, Brown M, Chakravorty S, Szestak T, et al Exported Proteins Required for Virulence and Rigidity of Plasmodium falciparum-Infected Human Erythrocytes. Cell. 2008;134(1):48–61. 10.1016/j.cell.2008.04.051 18614010 PMC2568870

[49] Fernandez V, Treutiger CJ, Nash GB, Wahlgren M. Multiple adhesive phenotypes linked to rosetting binding of erythrocytes in Plasmodium falciparum malaria. Infect Immun. 1998 6;66(6):2969–75. 9596774 10.1128/iai.66.6.2969-2975.1998 PMC108296

[50] Lambros C, Vanderberg JP. Synchronization of Plasmodium falciparum erythrocytic stages in culture. J Parasitol. 1979;65(3):418–20. 383936

[51] Udomsangpetch R, Wahlin B, Carlson J, Berzins K, Torii M, Aikawa M, et al Plasmodium falciparum-infected erythrocytes form spontaneous erythrocyte rosettes. J Exp Med. 1989;169(5):1835–40. 2654325 10.1084/jem.169.5.1835 PMC2189314

[52] Theisen M, Vuust J, Gottschau A, Jepsen S, Høgh B. Antigenicity and immunogenicity of recombinant glutamate-rich protein of Plasmodium falciparum expressed in Escherichia coli. Clin Diagn Lab Immunol. 1995;2(1):30–4. 7719909 10.1128/cdli.2.1.30-34.1995 PMC170096

[53] Forsström B, Axnäs BB, Stengele K, Bühler J, Albert TJ, Richmond TA, et al Proteome-wide Epitope Mapping of Antibodies Using Ultra-dense Peptide Arrays. Mol Cell Proteomics. 2014 6;13(6):1585–97. 10.1074/mcp.M113.033308 24705123 PMC4047477

[54] Zandian A, Forsström B, Häggmark-Månberg A, Schwenk JM, Uhlén M, Nilsson P, et al Whole-Proteome Peptide Microarrays for Profiling Autoantibody Repertoires within Multiple Sclerosis and Narcolepsy. J Proteome Res. 2017;16(3):1300–14. 10.1021/acs.jproteome.6b00916 28121444

[55] Boyle MJ, Wilson DW, Richards JS, Riglar DT, Tetteh KKA. Isolation of viable Plasmodium falciparum merozoites to define erythrocyte invasion events and advance vaccine and drug development. Proc Natl Acad Sci. 2010;107(32):14378–83. 10.1073/pnas.1009198107 20660744 PMC2922570

[56] Cooper RA. SDS-PAGE and Western Blotting of Plasmodium falciparum Proteins In: Doolan DL, editor. Malaria Methods and Protocols. Humana Press; 2002 p. 177–1888. 10.1385/1-59259-271-6:177 12125115

