# Supplementary figures and images for: SURGE complex of Plasmodium falciparum in the rhoptry-neck (SURFIN4.2-RON4-GLURP) contributes to merozoite invasion
Source: PLoS One. 2018 Aug 9;13(8):e0201669. doi: 10.1371/journal.pone.0201669 (PMC6084945; doi:10.1371/journal.pone.0201669)

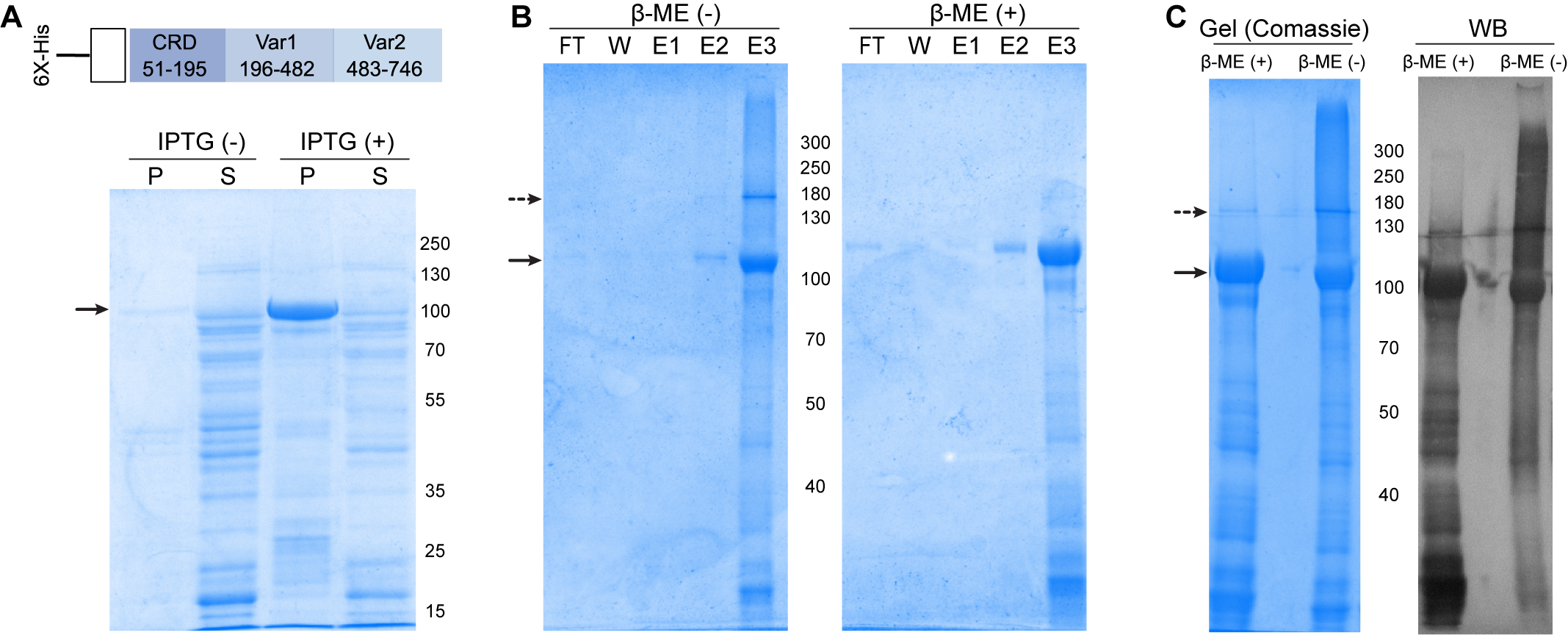

Supplement: S1 Fig — (A) Coomassie stained gel of the expressed extracellular domain of SURFIN4.2 in E. coli. (B) Coomassie stained gel of the purified extracellular domain of SURFIN4.2 in E. coli. (C) Coomassie stained gel and corresponding anti-His immunoblot showing the purified and concentrated protein. Solid arrows indicate the expected size of the full fragment expressed and dashed arrows indicate dimer size bands. P: Pellet; S: Supernatant; FT: Flow-through; W: Wash; E1: Elute 1 (50mM imidazole); E2: Elute 2 (100mM imidazole); E3: Elute 3 (150 mM imidazole); β-ME: 2-Mercaptoethanol. (TIF) [file pone.0201669.s002.tif]

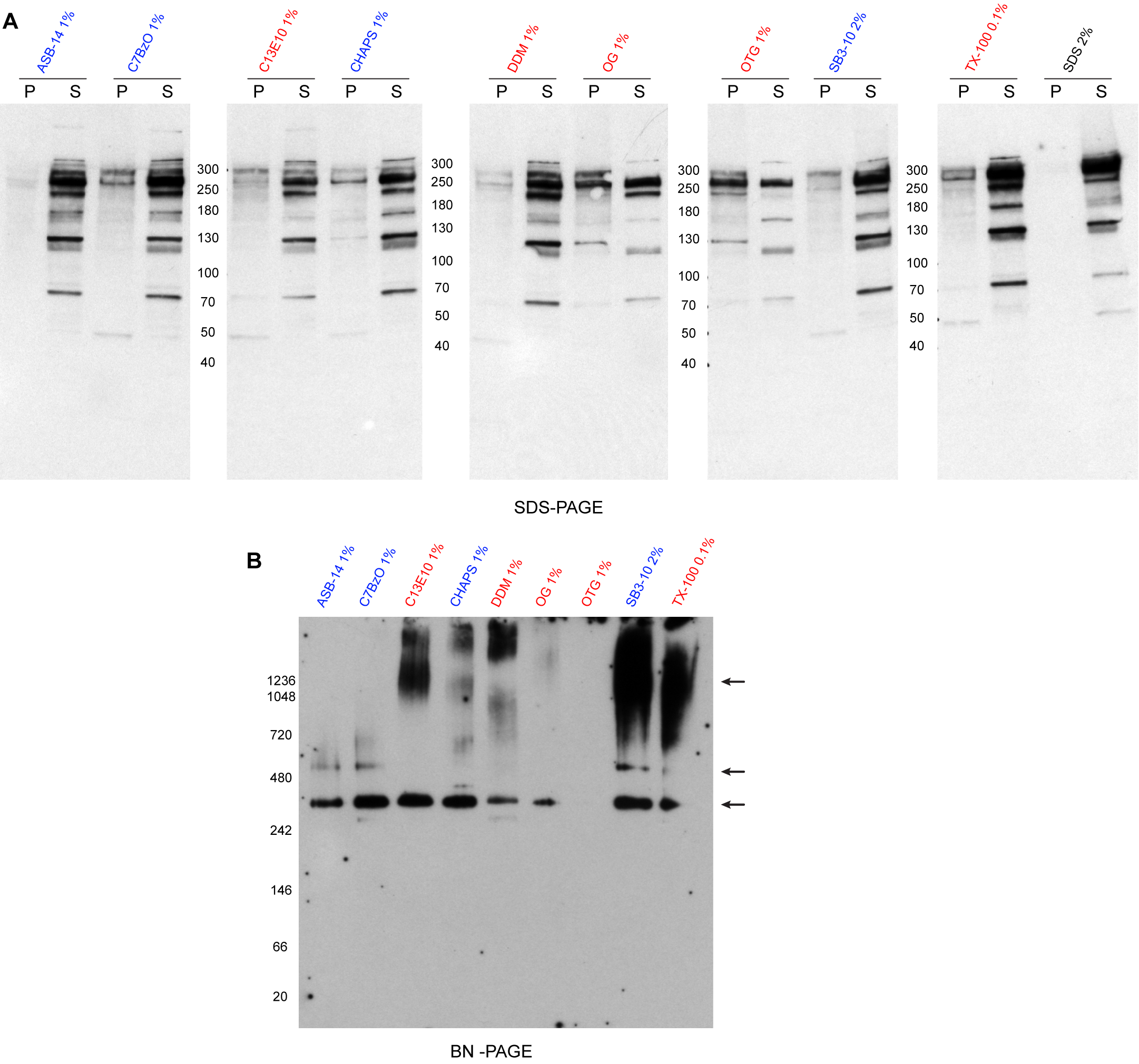

Supplement: S2 Fig — (A) SDS-PAGE for 10 different detergents tested, depicting both pellet (P) and supernatant (S) to check the detergent extraction efficiency into the supernatant fraction. (B) BN-PAGE for 9 of the detergent used, only supernatants were used here. Different colors for detergent labels indicate: ionic detergent in black, non-ionic detergents in red and zwitterionic detergent in blue. Both panel were probed with αSURFIN4.2. ASB-14: 3-[N,N-Dimethyl(3-myristoylaminopropyl)ammonio]propanesulfonate, Amidosulfobetaine-14, C7 C7BzO: 3-(4-Heptyl)phenyl-3-hydroxypropyl)dimethylammoniopropanesulfona C13E10: Polyoxyethylene (10) tridecyl ether (mixture of C11 to C14 iso-alkyl ethers with C13 iso-alkyl predominating) CHAPS: 3-[(3-Cholamidopropyl)dimethylammonio]-1-propanesulfonate hydrate DDM: n-Dodecyl β-D-maltoside OG: Octyl β-D-glucopyranoside OTG: Octyl β-D-1-thioglucopyranoside SB3-10: 3-(Decyldimethylammonio)­propane­sulfonate inner salt TX-100: Triton X-100 SDS: Sodium dodecyl sulfate (TIF) [file pone.0201669.s003.tif]

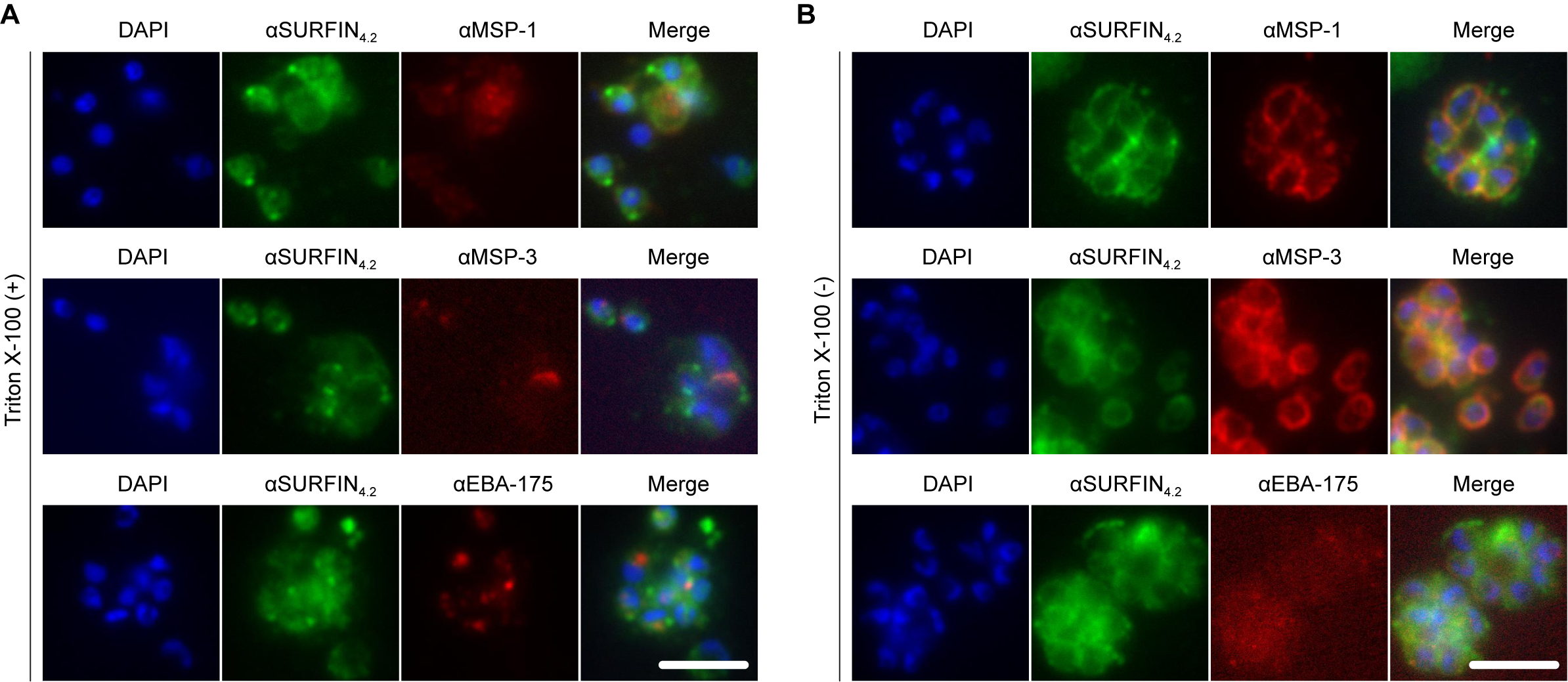

Supplement: S3 Fig — IFA on double-labeled free purified merozoites with (A) and without (B) a permeabilization step with Triton X-100. SURFIN4.2 is shown in green, surface markers (MSP-1 and MSP-3) and microneme markers (EBA-175 and AMA-1) are shown in red. Scale bar represents 5μm. (TIF) [file pone.0201669.s004.tif]
