# Supplementary material for: SURGE complex of Plasmodium falciparum in the rhoptry-neck (SURFIN4.2-RON4-GLURP) contributes to merozoite invasion
Source: PLoS One. 2018 Aug 9;13(8):e0201669. doi: 10.1371/journal.pone.0201669 (PMC6084945; doi:10.1371/journal.pone.0201669)

Uncropped images used in Fig 2.

Dashed boxes represent portions of the image used to build the figure

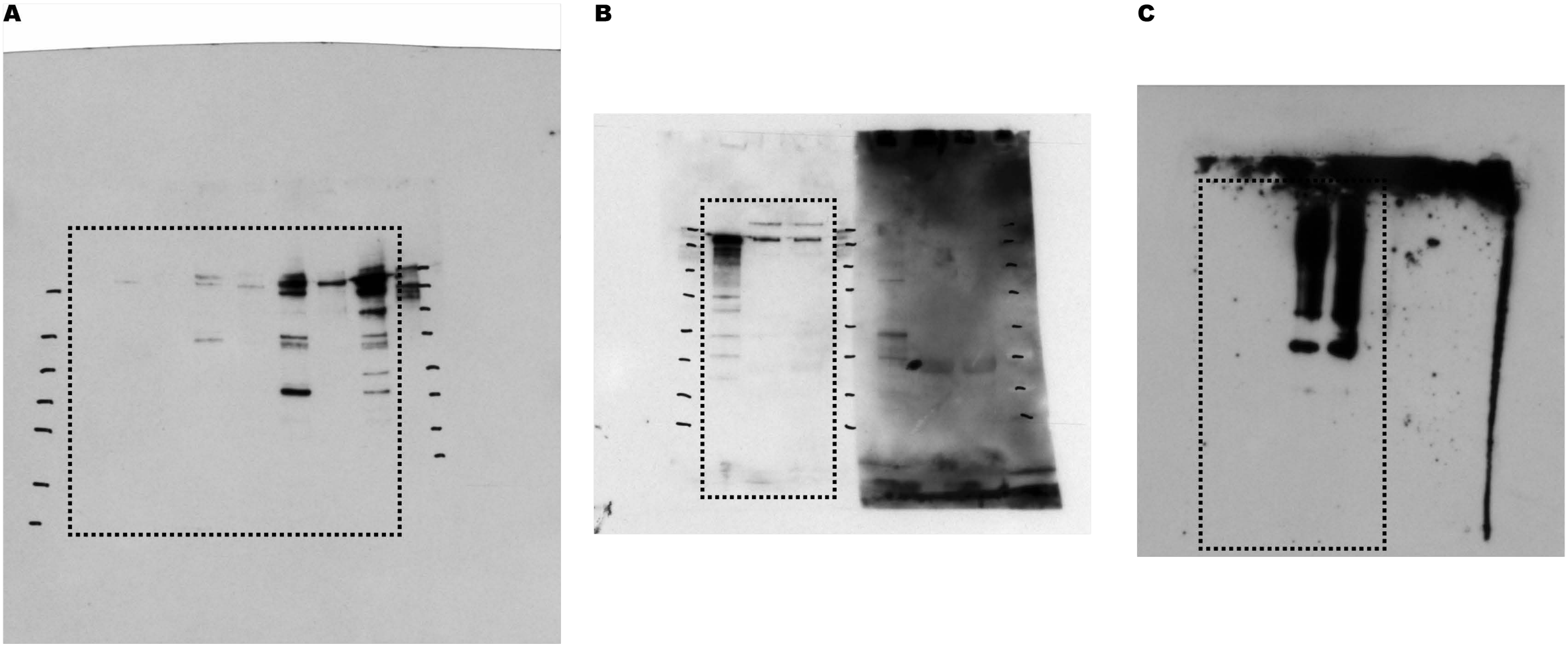

Uncropped images used in Fig 3.

A and B

Dashed boxes represent portions of the image used to build the figure. Blue correspond to IP with αControl IgG and red corresponds to IP with αSURFIN4.2

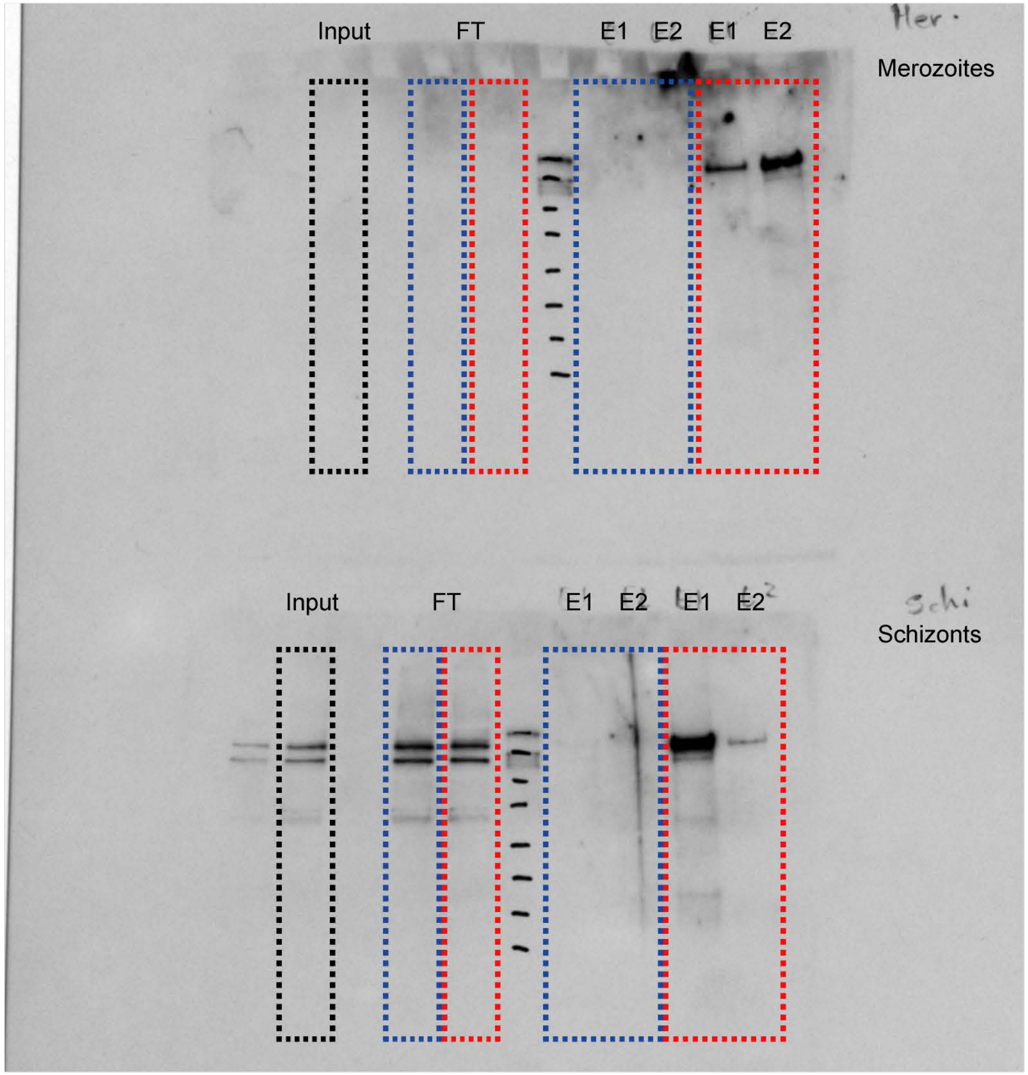

C

Dashed boxes represent portions of the image used to build the figure.

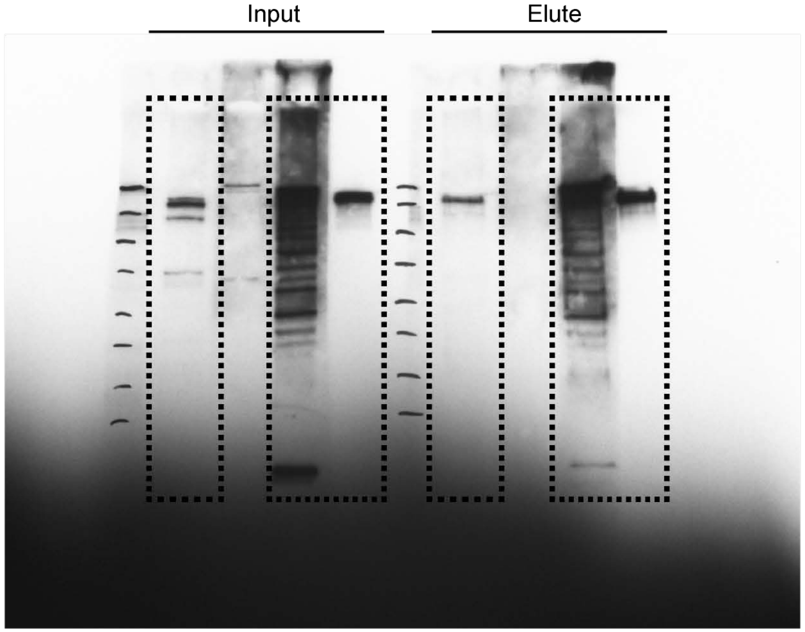

D

Dashed boxes represent portions of the image used to build the figure.

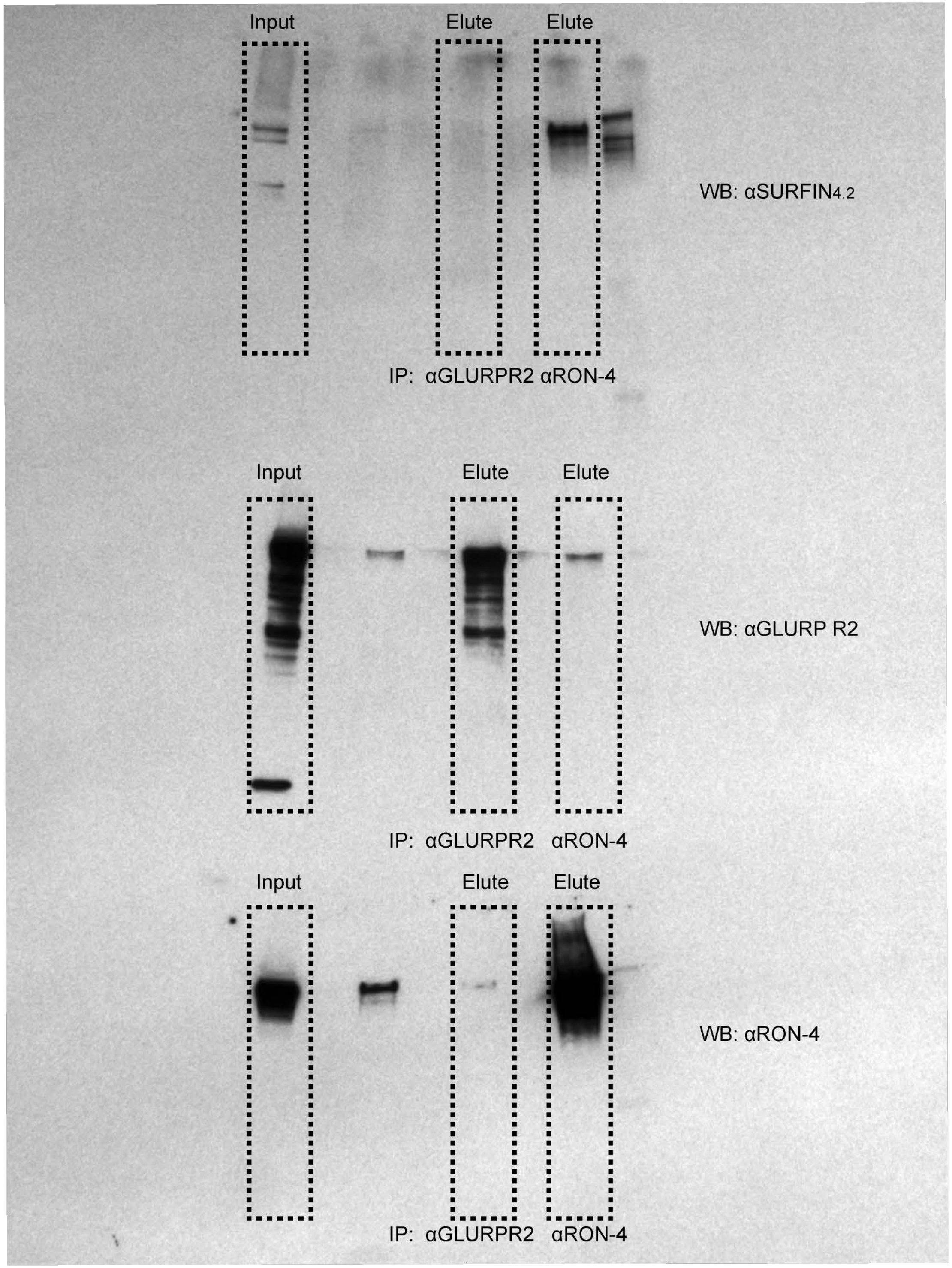

Supplement: S4 Fig — (A, B and C) Uncropped images used in Fig 2. (D, E, F and G) Uncropped images used in Fig 3. In panel D and E, blue boxes correspond to IP with control IgG and red boxes correspond to IP with αSURFIN4.2. (PDF) [file pone.0201669.s005.pdf]
