# Supplementary material for: SURGE complex of Plasmodium falciparum in the rhoptry-neck (SURFIN4.2-RON4-GLURP) contributes to merozoite invasion
Source: PLoS One. 2018 Aug 9;13(8):e0201669. doi: 10.1371/journal.pone.0201669 (PMC6084945; doi:10.1371/journal.pone.0201669)
Supplement: S1 Text — Samples 4 and 9 correspond to the eluted fractions after IP with αSURFIN4.2 from schizont and merozoite material respectively. (PDF) [file pone.0201669.s006.pdf]

Overview

LC-MS/MS

Sample name	Protein found in database	Entry name	Calculated MW	Score	Seq. cov.	Note
2	Gp195 surface antigen preprotein OS=Plasmodium falciparum GN=msp1 PE=2 SV=1	Q6LBT0_PLAFA	188643	2203	28%	
2	Glyceraldehyde-3-phosphate dehydrogenase OS=Plasmodium falciparum Vietnam Oak-Knoll (FVO) GN=PFFVO_05270 PE=3 SV=1	A0A024V013_PLAFA	37068	960	59%	
2	Enolase OS=Plasmodium falciparum Vietnam Oak-Knoll (FVO) GN=PFFVO_02844 PE=3 SV=1	A0A024V5N5_PLAFA	48989	748	28%	
2	Elongation factor 1-alpha OS=Plasmodium falciparum Vietnam Oak-Knoll (FVO) GN=PFFVO_04465 PE=3 SV=1	A0A024V296_PLAFA	49156	669	37%	
2	Phosphoglycerate kinase OS=Plasmodium falciparum Vietnam Oak-Knoll (FVO) GN=PFFVO_02527 PE=3 SV=1	A0A024V742_PLAFA	45569	606	30%	
2	Merozoite surface protein 1 (Fragment) OS=Plasmodium falciparum GN=MSP-1 PE=2 SV=1	Q8T6A9_PLAFA	75823	467	21%	
2	Hsp90-like protein OS=Plasmodium falciparum Vietnam Oak-Knoll (FVO) GN=PFFVO_01664 PE=3 SV=1	A0A024V8X4_PLAFA	86512	360	10%	
2	Hsp70-like protein OS=Plasmodium falciparum Vietnam Oak-Knoll (FVO) GN=PFFVO_02131 PE=3 SV=1	A0A024V7B6_PLAFA	74724	344	9%	
2	S-adenosylmethionine synthase OS=Plasmodium	A0A024V6I4_PLAFA	45272	333	15%	

Alphalyse A/S ■ Unsbjergvej 4 ■ 5220 Odense ■ Denmark
Tel: +45 6310 6500 ■ e-mail info@alphalyse.com
www.alphalyse.com

	falciparum Vietnam Oak-Knoll (FVO) GN=PFFVO_02524 PE=3 SV=1				
2	Uncharacterized protein OS=Plasmodium falciparum Vietnam Oak-Knoll (FVO) GN=PFFVO_03482 PE=3 SV=1	A0A024V542_PLAFA	95301	312	6%
2	Pyruvate kinase OS=Plasmodium falciparum Vietnam Oak-Knoll (FVO) GN=PFFVO_01529 PE=3 SV=1	A0A024VAV7_PLAFA	56480	294	16%
2	Ornithine aminotransferase OS=Plasmodium falciparum Vietnam Oak-Knoll (FVO) GN=PFFVO_06225 PE=3 SV=1	A0A024UX71_PLAFA	46938	275	14%
2	Hypoxanthine-guanine-xanthine phosphoribosyltransferase OS=Plasmodium falciparum Vietnam Oak-Knoll (FVO) GN=PFFVO_02805 PE=4 SV=1	A0A024V748_PLAFA	24632	272	34%
2	M1 family aminopeptidase OS=Plasmodium falciparum Vietnam Oak-Knoll (FVO) GN=PFFVO_03958 PE=4 SV=1	A0A024V3R7_PLAFA	126553	248	6%
2	L-lactate dehydrogenase OS=Plasmodium falciparum Vietnam Oak-Knoll (FVO) GN=PFFVO_04104 PE=3 SV=1	A0A024V3C8_PLAFA	34314	230	12%
2	Diphosphate-fructose-6-phosphate 1-phosphotransferase OS=Plasmodium falciparum Vietnam Oak-Knoll (FVO) GN=PFFVO_02450 PE=3 SV=1	A0A024V6T3_PLAFA	160663	191	4%
2	Chaperone DnaK OS=Plasmodium falciparum Vietnam Oak-Knoll (FVO) GN=PFFVO_02477 PE=3 SV=1	A0A024V6C5_PLAFA	72457	144	5%

Alphalyse A/S ■ Unsbjergvej 4 ■ 5220 Odense ■ Denmark
Tel: +45 6310 6500 ■ e-mail info@alphalyse.com
www.alphalyse.com

2	Uncharacterized protein OS=Plasmodium falciparum Vietnam Oak-Knoll (FVO) GN=PFFVO_01035 PE=4 SV=1	A0A024VCE7_PLAFA	27525	130	17%
2	Eukaryotic initiation factor 4A OS=Plasmodium falciparum Vietnam Oak-Knoll (FVO) GN=PFFVO_05337 PE=3 SV=1	A0A024UY98_PLAFA	46306	129	7%
2	Uncharacterized protein OS=Plasmodium falciparum Vietnam Oak-Knoll (FVO) GN=PFFVO_04306 PE=4 SV=1	A0A024V2J1_PLAFA	31297	105	8%
2	Uncharacterized protein OS=Plasmodium falciparum Vietnam Oak-Knoll (FVO) GN=PFFVO_00205 PE=3 SV=1	A0A024VCX1_PLAFA	121726	99	2%
2	Uncharacterized protein (Fragment) OS=Plasmodium falciparum Vietnam Oak-Knoll (FVO) GN=PFFVO_06138 PE=4 SV=1	A0A024UXG4_PLAFA	293738	96	1%
2	Uncharacterized protein OS=Plasmodium falciparum Vietnam Oak-Knoll (FVO) GN=PFFVO_00066 PE=4 SV=1	A0A024VEQ1_PLAFA	18479	50	8%
4	Merozoite surface protein 1 OS=Plasmodium falciparum Vietnam Oak-Knoll (FVO) GN=PFFVO_02613 PE=4 SV=1	A0A024V850_PLAFA	189101	664	8%
4	Glutamate-rich protein OS=Plasmodium falciparum (isolate 3D7) GN=PF10_0344 PE=4 SV=1	Q8IJ56_PLAF7	141024	223	6%
4	Uncharacterized protein OS=Plasmodium falciparum UGT5.1 GN=C923_03132 PE=4 SV=1	W7JXD3_PLAFA	124344	214	6%
4	Uncharacterized protein OS=Plasmodium falciparum Vietnam Oak-Knoll (FVO)	A0A024V579_PLAFA	94874	174	5%

	GN=PFFVO_03053 PE=4 SV=1				
6	Gp195 surface antigen preprotein OS=Plasmodium falciparum GN=msp1 PE=2 SV=1	Q6LBT0_PLAFA	188643	4123	43%
6	Merozoite surface protein 1 (Fragment) OS=Plasmodium falciparum GN=MSP-1 PE=2 SV=1	Q8T6A9_PLAFA	75823	1315	38%
6	Glyceraldehyde-3-phosphate dehydrogenase OS=Plasmodium falciparum Vietnam Oak-Knoll (FVO) GN=PFFVO_05270 PE=3 SV=1	A0A024V013_PLAFA	37068	826	43%
6	Elongation factor 1-alpha OS=Plasmodium falciparum Vietnam Oak-Knoll (FVO) GN=PFFVO_04465 PE=3 SV=1	A0A024V296_PLAFA	49156	654	37%
6	Phosphoglycerate kinase OS=Plasmodium falciparum Vietnam Oak-Knoll (FVO) GN=PFFVO_02527 PE=3 SV=1	A0A024V742_PLAFA	45569	648	34%
6	Enolase OS=Plasmodium falciparum Vietnam Oak-Knoll (FVO) GN=PFFVO_02844 PE=3 SV=1	A0A024V5N5_PLAFA	48989	633	24%
6	Hsp70-like protein OS=Plasmodium falciparum Vietnam Oak-Knoll (FVO) GN=PFFVO_02131 PE=3 SV=1	A0A024V7B6_PLAFA	74724	471	13%
6	Hsp90-like protein OS=Plasmodium falciparum Vietnam Oak-Knoll (FVO) GN=PFFVO_01664 PE=3 SV=1	A0A024V8X4_PLAFA	86512	468	12%
6	Uncharacterized protein (Fragment) OS=Plasmodium falciparum Vietnam Oak-Knoll (FVO) GN=PFFVO_03207 PE=4 SV=1	A0A024V5X8_PLAFA	68379	432	13%

Alphalyse A/S ■ Unsbjergvej 4 ■ 5220 Odense ■ Denmark
Tel: +45 6310 6500 ■ e-mail info@alphalyse.com
www.alphalyse.com

6	S-adenosylmethionine synthase OS=Plasmodium falciparum Vietnam Oak-Knoll (FVO) GN=PFFVO_02524 PE=3 SV=1	A0A024V6I4_PLAFA	45272	379	15%
6	Uncharacterized protein OS=Plasmodium falciparum Vietnam Oak-Knoll (FVO) GN=PFFVO_03482 PE=3 SV=1	A0A024V542_PLAFA	95301	365	9%
6	Diphosphate-fructose-6-phosphate 1-phosphotransferase OS=Plasmodium falciparum Vietnam Oak-Knoll (FVO) GN=PFFVO_02450 PE=3 SV=1	A0A024V6T3_PLAFA	160663	360	8%
6	Ornithine aminotransferase OS=Plasmodium falciparum Vietnam Oak-Knoll (FVO) GN=PFFVO_06225 PE=3 SV=1	A0A024UX71_PLAFA	46938	349	14%
6	Hypoxanthine-guanine-xanthine phosphoribosyltransferase OS=Plasmodium falciparum Vietnam Oak-Knoll (FVO) GN=PFFVO_02805 PE=4 SV=1	A0A024V748_PLAFA	24632	328	44%
6	L-lactate dehydrogenase OS=Plasmodium falciparum Vietnam Oak-Knoll (FVO) GN=PFFVO_04104 PE=3 SV=1	A0A024V3C8_PLAFA	34314	322	24%
6	Uncharacterized protein OS=Plasmodium falciparum UGT5.1 GN=C923_03132 PE=4 SV=1	W7JXD3_PLAFA	124344	254	6%
6	Uncharacterized protein OS=Plasmodium falciparum Vietnam Oak-Knoll (FVO) GN=PFFVO_01453 PE=3 SV=1	A0A024VAR9_PLAFA	93184	193	7%
6	M1 family aminopeptidase OS=Plasmodium falciparum Vietnam Oak-Knoll (FVO) GN=PFFVO_03958 PE=4 SV=1	A0A024V3R7_PLAFA	126553	179	4%

Alphalyse A/S ■ Unsbjergvej 4 ■ 5220 Odense ■ Denmark
Tel: +45 6310 6500 ■ e-mail info@alphalyse.com
www.alphalyse.com

6	Uncharacterized protein OS=Plasmodium falciparum Vietnam Oak-Knoll (FVO) GN=PFFVO_01035 PE=4 SV=1	A0A024VCE7_PLAFA	27525	167	17%
6	Elongation factor 2 OS=Plasmodium falciparum Vietnam Oak-Knoll (FVO) GN=PFFVO_05133 PE=4 SV=1	A0A024UZJ8_PLAFA	94546	160	3%
6	Uncharacterized protein OS=Plasmodium falciparum Vietnam Oak-Knoll (FVO) GN=PFFVO_05312 PE=4 SV=1	A0A024UY79_PLAFA	164287	154	2%
6	Pyruvate kinase OS=Plasmodium falciparum Vietnam Oak-Knoll (FVO) GN=PFFVO_01529 PE=3 SV=1	A0A024VAV7_PLAFA	56480	146	4%
6	Eukaryotic initiation factor 4A OS=Plasmodium falciparum Vietnam Oak-Knoll (FVO) GN=PFFVO_05337 PE=3 SV=1	A0A024UY98_PLAFA	46306	126	5%
6	Polyadenylate-binding protein OS=Plasmodium falciparum Vietnam Oak-Knoll (FVO) GN=PFFVO_03505 PE=3 SV=1	A0A024V3P9_PLAFA	97439	122	4%
6	Chaperone DnaK OS=Plasmodium falciparum Vietnam Oak-Knoll (FVO) GN=PFFVO_02477 PE=3 SV=1	A0A024V6C5_PLAFA	72457	117	5%
6	Uncharacterized protein OS=Plasmodium falciparum FCH/4 GN=PFFCH_00586 PE=4 SV=1	A0A024VT85_PLAFA	13960	88	18%
6	Uncharacterized protein OS=Plasmodium falciparum Vietnam Oak-Knoll (FVO) GN=PFFVO_02339 PE=4 SV=1	A0A024V7I0_PLAFA	99081	70	3%
6	Uncharacterized protein OS=Plasmodium falciparum Vietnam Oak-Knoll (FVO)	A0A024VEQ1_PLAFA	18479	53	8%

	GN=PFFVO_00066 PE=4 SV=1				
8	No identification		0	0	0%
9	Merozoite surface protein 1 OS=Plasmodium falciparum Vietnam Oak-Knoll (FVO) GN=PFFVO_02613 PE=4 SV=1	A0A024V850_PLAFA	189101	1031	17%
9	Uncharacterized protein OS=Plasmodium falciparum UGT5.1 GN=C923_03132 PE=4 SV=1	W7JXD3_PLAFA	124344	440	12%
9	Glutamate-rich protein OS=Plasmodium falciparum (isolate 3D7) GN=PF10_0344 PE=4 SV=1	Q8IJ56_PLAF7	141024	406	8%
9	Merozoite surface protein 1 (Fragment) OS=Plasmodium falciparum GN=MSP-1 PE=2 SV=1	Q8T6A9_PLAFA	75823	317	10%
9	Uncharacterized protein OS=Plasmodium falciparum Vietnam Oak-Knoll (FVO) GN=PFFVO_03053 PE=4 SV=1	A0A024V579_PLAFA	94874	305	12%
9	Uncharacterized protein OS=Plasmodium falciparum FCH/4 GN=PFFCH_03287 PE=4 SV=1	A0A024VN30_PLAFA	108332	231	6%
9	Glyceraldehyde-3-phosphate dehydrogenase OS=Plasmodium falciparum Vietnam Oak-Knoll (FVO) GN=PFFVO_05270 PE=3 SV=1	A0A024V013_PLAFA	37068	54	4%
9	Phosphoenolpyruvate carboxykinase (ATP) OS=Plasmodium falciparum Vietnam Oak-Knoll (FVO) GN=PFFVO_04304 PE=3 SV=1	A0A024V3K4_PLAFA	66794	37	2%

Experimental

Samples received

The following samples were received at Alphalyse for protein analysis.

2
4
6
8
9

Protein identification by nanoLC-MS/MS peptide sequencing and database search

The protein samples were reduced and alkylated with iodoacetamide, i.e. carbamidomethylated, and subsequently digested with trypsin that cleaves after lysine and arginine residues. The resulting peptides were concentrated by Speed Vac lyophilization and redissolved for injection on a Dionex nano-LC system and MS/MS analysis on a Bruker Maxis Impact QTOF instrument. The MS/MS spectra were used for Mascot database searching. The data are searched against in-house protein databases downloaded from UniProt and NCBI containing more than 70 million known non-redundant protein sequences. The data can also be searched against a custom database containing specific protein sequences provided by the client.

The Mascot software finds matching proteins in the database by their peptide masses and peptide fragment masses. The protein identification is based on a probability-scoring algorithm (www.matrixscience.com) and the significant best matching protein is shown in the Results. Homologous proteins with a lower score are not included in the report. If the protein from the correct organism is not present in the database, then a significant matching homologous protein from another organism is reported. If several proteins are identified with a significant score then several protein identifications are reported for the sample.

The identified database protein sequences are shown in the Results together with the obtained mass spectrometric peptide maps. The peptides used for the identification are highlighted in the sequence and the matching peptides are listed for comparison of the determined and calculated values.The same peptide can appear as multible identifications. It is considered a positive identification when at least 2 peptides have an Ions score above 35 or if a protein under 20kDa has 1 peptide with an Ions score above 50. The sequence coverage is not considered for the identification. The total Mascot score provided for each identification is a total of all the individual peptide Mascot scores.

Bioinformatics tools

Bioinformatics Tools are show as hyperlinks for each identified protein in the report. The identified protein can be looked up at the websites of UniProt and NCBI (National Center for Biotechnology Information) by selecting the UniProt/NCBI Entry hyperlink. A Blast homology search in the NCBI database is found by selecting Blink NCBI. Known functional domains in the protein can be found in the Conserved Domain Database (Conserved Domains in NCBI).

The Bioinformatics Guide at (www.alphalyse.com/bioinformatics-guide.html) explains and guides you through a range of important bioinformatics tools to let you investigate the function and properties of the protein. The guide contains hyperlinks to bioinformatics search forms and case examples on how the tools are used.

Alphalyse A/S ■ Unsbjergvej 4 ■ 5220 Odense ■ Denmark
Tel: +45 6310 6500 ■ e-mail info@alphalyse.com
www.alphalyse.com

Results

Sample name: 2

Protein Information

Protein name:	Gp195 surface antigen preprotein OS=Plasmodium falciparum GN=msp1 PE=2 SV=1
Entry name:	Q6LBT0_PLAFA
Calculated MW:	188643
Calculated pI:	5.98
Mascot score:	2203
Sequence coverage:	28%
Bioinformatic tools:	1: UniProt Entry 2: Conserved Domains in NCBI

Analysis Information

- Enzyme: Trypsin
- Variable modifications: Oxidation (M)
- Fixed modifications: Carbamidomethyl (C)
- Database search program: Mascot version 2.4
- Peptide Tolerance: 10 ppm
- Database: UniprotTREMBL (50011027 protein sequences)

Protein sequence

Matched peptides shown in bold underline

1 MKIIFFLCSF LFFIINTQCV THESYQELVK **KLEALEDAVL** **TGYSLFQK**EK
51 **MVLNEGTSGT** **AVTTSTPGSK** **GSVASGGSGG** **SVASGGSVAS** **GGSVASGGSV**
101 **ASGGSGNSR**R TNPSDNSSDS DAKSYADLKH RVRNYLLTIK ELKYPQLFDL
151 TNHMLTLCDN IHGFKYLIDG YEEINELLYK **LNFYFDLLR**A KLNDVCANDY
201 CQIPFNLKIR ANELDVLKKL VFGYRKPLDN IKDNVGKMED YIKKNKK**TIE**
251 **NINELIEESK** **K**TIDKNKNAT KEEEKKK**LYQ** **AQYDLSIYNK** QLEEAHNLIS
301 VLEKRIDTLK KNENIKELLD KINEIKNPPP ANSGNTPNTL LDKNKKIEEH
351 EKEIKEIAKT IKFNIDSLFT DPLELEYYLR EKNKNIDISA KVETKESTEP
401 NEYPNGVTYP LSYNDINNAL NELNSFGDLI NPFDYTKEPS KNIYTDNERK
451 KFINEIKEKI KIEKKKIESD KKSYEDRSKS LNDITKEYEK **LLNEIYDSK**F
501 NNNIDLTNFE KMMGKRYSYK VEKLTHHNTF ASYENSKHNL EKLTKALKYM
551 EDYSLRNIVV EKELKYYKNL ISKIENEIET LVENIKKDEE QLFEKKITKD
601 ENKPDEK**ILE** **VSDIVK**VQVQ KVLLMNKIDE LKKTQLILKN VELKHNIHVP
651 NSYKQENKQE PYYLIVLKKE IDKLKVFMPK **VESLINEEKK** NIKTEGQSDN
701 SEPSTEGEIT GQATTKPGQQ AGSALEGDSV QAQAQEQK**QA** **QPPVPVPVPE**
751 **AKAQVPTPPA** **PVNNK**TENVS KLDYLEKLYE FLNTSYICHK YILVSHSTMN
801 EKILKQYKIT KEEESKLSSC DPLDLLFNIQ NNIPVMYSMF DSLNNSLSQL
851 FMEIYEKEMV CNLYKLKDND KIKNLLEEAK KVSTSVK**TLS** **SSSMQPLSLT**

Alphalyse A/S ■ Unsbjergvej 4 ■ 5220 Odense ■ Denmark
Tel: +45 6310 6500 ■ e-mail info@alphalyse.com
www.alphalyse.com

901 **PQDKPEVSAN** **DDTSHSTNLN** **NSLKLFENIL** **SLGKNKNIYQ** **ELIGQK**SSEN

951 FYEK**ILKDSD** **TFYNESFTNF** **VK**SKADDINS LNDESKRKKL EEDINKLKKT

1001 LQLSFDLYNK YKLKLERLFD KKKTVGKYKM QIKKLTLLKE QLESKLNSLN

1051 NPK**HVLQNFS** **VFFNK**KK**EAE** **IAETENTLEN** **TK**ILLKHYKG LVK**YYNGESS**

1101 **PLKTLSEESI** **QTEDNYASLE** **NFK**VLSKLEG K**LKDNLNLEK** KKLSYLSSGL

1151 HHLIAELKEV IKNK**NYTGNS** **PSENNTDVNN** **ALESYKKFLP** **EGTDVATVVS**

1201 **ESGSDTLEQS** **QPK**KPASTHV GAESNTITTS QNVDDEVDDV IIVPIFGESE

1251 EDYDDLGQVV TGEAVTPSVI DNILSK**IENE** **YEVLYLKPLA** **GVYR**SLKK**QL**

1301 **ENNVMTFNVN** **VKDILNSR**FN KRENFKNVLE SDLIPYKDLT SSNYVVKDPY

1351 KFLNKEKRDK FLSSYNYIKD SIDTDINFAN DVLGYYKILS EKYKSDLDSI

1401 KKYINDKQGE NEK**YLPFLNN** **IETLYK**TVND KIDLFVIHLE AK**VLNYTYEK**

1451 SNVEVK**IKEL** **NYLK**TIQDKL ADFKK**NNNFV** **GIADLSTDYN** **HNNLLTKFLS**

1501 **TGMVFENLAK** TVLSNLLDGN LQGMLNISQH QCVKKQCPQN SGCFRHLDER

1551 EECKCLLNYK QEGDKCVENP NPTCNENNGG CDADAKCTEE DSGSNGKKIT

1601 CECTKPDSYP LFDGIFCSSS NFLGISFLLI LMLILYSFI

Peptides used for identification

Peptides shown in bold have been analysed by MS/MS sequencing

Start - End	Observed	Mr(expt)	Mr(calc)	Delta	Miss	Sequence
31 - 48	675.70	2024.08	2024.08	-1	1	K.KLEALEDAVLTGYSLFQK.E (Ions score 44)
31 - 48	675.70	2024.08	2024.08	0	1	K.KLEALEDAVLTGYSLFQK.E (Ions score 75)
31 - 48	675.70	2024.09	2024.08	2	1	K.KLEALEDAVLTGYSLFQK.E (Ions score 60)
51 - 70	977.47	1952.94	1952.94	0	0	K.MVLNEGTSGTAVTTSTPGSK.G + Oxidation (M) (Ions score 103)
51 - 70	977.48	1952.94	1952.94	0	0	K.MVLNEGTSGTAVTTSTPGSK.G + Oxidation (M) (Ions score 115)
51 - 70	977.48	1952.94	1952.94	1	0	K.MVLNEGTSGTAVTTSTPGSK.G + Oxidation (M) (Ions score 94)
71 - 109	1023.80	3068.39	3068.40	-3	0	K.GSVASGGSGGSVASGGSVASGGSVASGGSVASGGSGNSR.R (Ions score 52)
181 - 189	600.83	1199.64	1199.63	3	0	K.LNFYFDLLR.A (Ions score 40)
248 - 261	553.96	1658.87	1658.87	-2	1	K.TIENINELIEESKK.T (Ions score 40)
248 - 261	553.96	1658.87	1658.87	-1	1	K.TIENINELIEESKK.T (Ions score 38)
278 - 290	809.91	1617.80	1617.80	-2	0	K.LYQAQYDLSIYNK.Q (Ions score 76)
278 - 290	809.91	1617.80	1617.80	0	0	K.LYQAQYDLSIYNK.Q (Ions score 64)
278 - 290	809.91	1617.80	1617.80	1	0	K.LYQAQYDLSIYNK.Q (Ions score 84)
491 - 499	547.79	1093.56	1093.57	-1	0	K.LLNEIYDSK.F (Ions score 23)
608 - 616	508.30	1014.59	1014.60	-2	0	K.ILEVSDIVK.V (Ions score 13)
681 - 690	594.83	1187.64	1187.64	-2	1	K.VESLINEEKK.N (Ions score 24)
681 - 690	594.83	1187.64	1187.64	2	1	K.VESLINEEKK.N (Ions score 46)
739 - 752	728.91	1455.81	1455.81	-1	0	K.QAQPPVPVPVPEAK.A (Ions score 52)
739 - 752	728.91	1455.81	1455.81	0	0	K.QAQPPVPVPVPEAK.A (Ions score 49)
739 - 752	728.91	1455.81	1455.81	0	0	K.QAQPPVPVPVPEAK.A (Ions score 58)
753 - 765	666.87	1331.72	1331.72	-2	0	K.AQVPTPPAPVNNK.T (Ions score 55)
753 - 765	666.87	1331.72	1331.72	-1	0	K.AQVPTPPAPVNNK.T (Ions score 42)
753 - 765	666.87	1331.72	1331.72	0	0	K.AQVPTPPAPVNNK.T (Ions score 61)
888 - 924	990.72	3958.87	3958.87	-1	0	K.TLSSSSMQPLSLTPQDKPEVSANDDTSHSTNLNNSLK.L + Oxidation (M) (Ions score 64)
888 - 924	990.72	3958.87	3958.87	-1	0	K.TLSSSSMQPLSLTPQDKPEVSANDDTSHSTNLNNSLK.L + Oxidation (M) (Ions score 41)
888 - 924	990.72	3958.87	3958.87	0	0	K.TLSSSSMQPLSLTPQDKPEVSANDDTSHSTNLNNSLK.L + Oxidation (M) (Ions score 67)
925 - 934	567.33	1132.65	1132.65	-2	0	K.LFENILSLGK.N (Ions score 55)
925 - 934	567.33	1132.65	1132.65	-1	0	K.LFENILSLGK.N (Ions score 13)
925 - 934	567.33	1132.65	1132.65	0	0	K.LFENILSLGK.N (Ions score 50)
935 - 946	724.40	1446.78	1446.78	-3	1	K.NKNIYQELIGQK.S (Ions score 46)
935 - 946	724.40	1446.78	1446.78	-1	1	K.NKNIYQELIGQK.S (Ions score 46)

Alphalyse A/S ■ Unsbjergvej 4 ■ 5220 Odense ■ Denmark
Tel: +45 6310 6500 ■ e-mail info@alphalyse.com
www.alphalyse.com

937 - 946	603.33	1204.64	1204.65	0	0	K.NIYQELIGQK.S (Ions score 45)
955 - 972	723.36	2167.04	2167.05	-1	1	K.ILKDSDTFYNESFTNFVK.S (Ions score 65)
955 - 972	723.36	2167.05	2167.05	0	1	K.ILKDSDTFYNESFTNFVK.S (Ions score 54)
958 - 972	907.40	1812.79	1812.78	1	0	K.DSDTFYNESFTNFVK.S (Ions score 67)
958 - 972	907.40	1812.79	1812.78	1	0	K.DSDTFYNESFTNFVK.S (Ions score 50)
1054 - 1065	740.39	1478.76	1478.77	-2	0	K.HVLQNFSVFFNK.K (Ions score 58)
1054 - 1065	740.39	1478.77	1478.77	-1	0	K.HVLQNFSVFFNK.K (Ions score 68)
1054 - 1065	740.39	1478.77	1478.77	0	0	K.HVLQNFSVFFNK.K (Ions score 60)
1068 - 1082	846.40	1690.79	1690.79	-2	0	K.EAEIAETENTLENTK.I (Ions score 109)
1068 - 1082	846.40	1690.79	1690.79	-2	0	K.EAEIAETENTLENTK.I (Ions score 113)
1068 - 1082	846.40	1690.79	1690.79	0	0	K.EAEIAETENTLENTK.I (Ions score 109)
1094 - 1103	579.28	1156.54	1156.54	0	0	K.YYNGESSPLK.T (Ions score 47)
1094 - 1103	579.28	1156.54	1156.54	2	0	K.YYNGESSPLK.T (Ions score 29)
1104 - 1123	1159.54	2317.06	2317.06	-1	0	K.TLSEESIQTEDNYASLENFK.V (Ions score 150)
1104 - 1123	1159.54	2317.06	2317.06	0	0	K.TLSEESIQTEDNYASLENFK.V (Ions score 135)
1104 - 1123	1159.54	2317.06	2317.06	1	0	K.TLSEESIQTEDNYASLENFK.V (Ions score 134)
1132 - 1140	543.81	1085.61	1085.61	-1	1	K.LKDNLNLEK.K (Ions score 10)
1165 - 1187	853.72	2558.15	2558.15	-2	1	K.NYTGNSPSENNTDVNNALESYKK.F (Ions score 79)
1188 - 1213	907.77	2720.30	2720.30	-1	0	K.FLPEGTDVATVVSESGSDTLEQSQPK.K (Ions score 99)
1188 - 1213	907.77	2720.30	2720.30	0	0	K.FLPEGTDVATVVSESGSDTLEQSQPK.K (Ions score 101)
1188 - 1213	907.78	2720.30	2720.30	0	0	K.FLPEGTDVATVVSESGSDTLEQSQPK.K (Ions score 110)
1277 - 1294	723.72	2168.15	2168.15	-1	0	K.IENEYEVLYLKPLAGVYR.S (Ions score 109)
1277 - 1294	723.72	2168.15	2168.15	0	0	K.IENEYEVLYLKPLAGVYR.S (Ions score 112)
1277 - 1294	723.73	2168.15	2168.15	1	0	K.IENEYEVLYLKPLAGVYR.S (Ions score 104)
1299 - 1312	833.41	1664.81	1664.82	-3	0	K.QLENNVMTFNVNVK.D + Oxidation (M) (Ions score 59)
1299 - 1312	833.42	1664.82	1664.82	-2	0	K.QLENNVMTFNVNVK.D + Oxidation (M) (Ions score 110)
1299 - 1312	833.42	1664.82	1664.82	-2	0	K.QLENNVMTFNVNVK.D + Oxidation (M) (Ions score 83)
1299 - 1318	788.74	2363.19	2363.19	0	1	K.QLENNVMTFNVNVKDILNSR.F + Oxidation (M) (Ions score 129)
1414 - 1426	814.44	1626.86	1626.87	-1	0	K.YLPFLNNIETLYK.T (Ions score 67)
1414 - 1426	814.44	1626.87	1626.87	0	0	K.YLPFLNNIETLYK.T (Ions score 85)
1414 - 1426	814.44	1626.87	1626.87	0	0	K.YLPFLNNIETLYK.T (Ions score 78)
1414 - 1426	814.44	1626.87	1626.87	0	0	K.YLPFLNNIETLYK.T (Ions score 52)
1443 - 1450	515.27	1028.52	1028.52	5	0	K.VLNYTYEK.S (Ions score 11)
1457 - 1464	510.81	1019.60	1019.60	-3	1	K.IKELNYLK.T (Ions score 11)
1476 - 1497	826.40	2476.19	2476.20	-2	0	K.NNNFVGIADLSTDYNHNNLLTK.F (Ions score 125)
1476 - 1497	826.41	2476.20	2476.20	-1	0	K.NNNFVGIADLSTDYNHNNLLTK.F (Ions score 138)
1476 - 1497	826.41	2476.20	2476.20	1	0	K.NNNFVGIADLSTDYNHNNLLTK.F (Ions score 133)
1498 - 1510	728.88	1455.74	1455.74	-3	0	K.FLSTGMVFENLAK.T (Ions score 35)
1498 - 1510	728.88	1455.74	1455.74	-1	0	K.FLSTGMVFENLAK.T (Ions score 36)
1498 - 1510	728.88	1455.74	1455.74	1	0	K.FLSTGMVFENLAK.T (Ions score 46)
1498 - 1510	736.87	1471.73	1471.74	-2	0	K.FLSTGMVFENLAK.T + Oxidation (M) (Ions score 77)
1498 - 1510	736.88	1471.74	1471.74	-1	0	K.FLSTGMVFENLAK.T + Oxidation (M) (Ions score 59)
1498 - 1510	736.88	1471.74	1471.74	0	0	K.FLSTGMVFENLAK.T + Oxidation (M) (Ions score 77)
1498 - 1510	736.88	1471.74	1471.74	2	0	K.FLSTGMVFENLAK.T + Oxidation (M) (Ions score 55)

LC-MSMS Protein Identification Report

Order 16372_Plasmodium falciparum

PICK 'n POST
PROTEIN ANALYSIS

Sample name: 2

Protein Information

**Protein name:**	**Glyceraldehyde-3-phosphate dehydrogenase OS=Plasmodium falciparum Vietnam Oak-Knoll (FVO) GN=PFFVO_05270 PE=3 SV=1**
Entry name:	A0A024V013_PLAFA
Calculated MW:	37068
Calculated pI:	7.59
Mascot score:	960
Sequence coverage:	59%
Bioinformatic tools:	1: UniProt Entry 2: Conserved Domains in NCBI

Analysis Information

- Enzyme: Trypsin
- Variable modifications: Oxidation (M)
- Fixed modifications: Carbamidomethyl (C)
- Database search program: Mascot version 2.4
- Peptide Tolerance: 10 ppm
- Database: UniprotTREMBL (50011027 protein sequences)

Protein sequence

Matched peptides shown in bold underline

1 MAVTK**LGING** **FGR**IGRLVFR AAFGRKDIEV VAINDPFMDL NHLCYLLKYD
51 SVHGQFPCEV THADGFLLIG EKK**VSVFAEK** **DPSQIPWGKC** **QVDVVCESTG**
101 **VFLTKELASS** **HLK**GGAKK**VI** **MSAPPKDDTP** **IYVMGINHHQ** **YDTKQLIVSN**
151 **ASCTTNCLAP** **LAK**VINDRFG IVEGLMTTVH ASTANQLVVD GPSKGGKDWR
201 AGR**CALSNII** **PASTGAAK**AV GKVLPELNGK **LTGVAFRVPI** **GTVSVVDLVC**
251 **RLQKPAKYEE** **VALEIKK**AAE GPLK**GILGYT** **EDEVVSQDFV** **HDNR**SSIFDM
301 K**AGLALNDNF** **FKLVSWYDNE** **WGYSNRVLDL** **AVHITNN**

Peptides used for identification

Peptides shown in bold have been analysed by MS/MS sequencing

Start - End	Observed	Mr(expt)	Mr(calc)	Delta	Miss	Sequence
6 - 13	417.23	832.45	832.46	-2	0	**K.LGINGFGR.I** (Ions score 40)
6 - 13	417.23	832.45	832.46	-1	0	**K.LGINGFGR.I** (Ions score 44)
6 - 13	417.23	832.45	832.46	-1	0	**K.LGINGFGR.I** (Ions score 40)
74 - 89	596.65	1786.92	1786.93	-1	1	**K.VSVFAEKDPSQIPWGK.C** (Ions score 53)
74 - 89	596.65	1786.92	1786.93	-1	1	**K.VSVFAEKDPSQIPWGK.C** (Ions score 55)
74 - 89	596.65	1786.93	1786.93	1	1	**K.VSVFAEKDPSQIPWGK.C** (Ions score 58)
90 - 105	921.44	1840.87	1840.87	-1	0	**K.CQVDVVCESTGVFLTK.E** (Ions score 66)
90 - 105	921.44	1840.87	1840.87	-1	0	**K.CQVDVVCESTGVFLTK.E** (Ions score 75)
106 - 113	442.75	883.48	883.48	0	0	**K.ELASSHLK.G** (Ions score 20)
119 - 144	751.36	3001.42	3001.43	-4	1	**K.VIMSAPPKDDTPIYVMGINHHQYDTK.Q** + 2 Oxidation (M)

Alphalyse A/S ■ Unsbjergvej 4 ■ 5220 Odense ■ Denmark
Tel: +45 6310 6500 ■ e-mail info@alphalyse.com
www.alphalyse.com

						(Ions score 25)
145 - 163	687.69	2060.04	2060.04	-2	0	K.QLIVSNASCTTNCLAPLAK.V (Ions score 63)
145 - 163	1031.03	2060.04	2060.04	-1	0	K.QLIVSNASCTTNCLAPLAK.V (Ions score 136)
145 - 163	1031.03	2060.04	2060.04	-1	0	K.QLIVSNASCTTNCLAPLAK.V (Ions score 140)
204 - 218	737.39	1472.77	1472.77	0	0	R.CALSNIIPASTGAAK.A (Ions score 69)
204 - 218	737.39	1472.77	1472.77	0	0	R.CALSNIIPASTGAAK.A (Ions score 72)
204 - 218	737.39	1472.77	1472.77	0	0	R.CALSNIIPASTGAAK.A (Ions score 69)
231 - 251	753.43	2257.26	2257.26	1	1	K.LTGVAFRVPIGTVSVVDLVCR.L (Ions score 43)
238 - 251	757.42	1512.83	1512.83	-1	0	R.VPIGTVSVVDLVCR.L (Ions score 112)
238 - 251	757.42	1512.83	1512.83	-1	0	R.VPIGTVSVVDLVCR.L (Ions score 89)
238 - 251	757.42	1512.83	1512.83	1	0	R.VPIGTVSVVDLVCR.L (Ions score 101)
252 - 266	587.00	1757.99	1757.99	-1	1	R.LQKPAKYEEVALEIK.K (Ions score 14)
258 - 267	611.34	1220.67	1220.67	1	1	K.YEEVALEIKK.A (Ions score 36)
258 - 267	611.34	1220.67	1220.67	1	1	K.YEEVALEIKK.A (Ions score 8)
275 - 294	1147.04	2292.06	2292.07	-1	0	K.GILGYTEDEVVSQDFVHDNR.S (Ions score 134)
275 - 294	765.03	2292.07	2292.07	0	0	K.GILGYTEDEVVSQDFVHDNR.S (Ions score 148)
275 - 294	1147.04	2292.07	2292.07	0	0	K.GILGYTEDEVVSQDFVHDNR.S (Ions score 159)
275 - 294	765.03	2292.07	2292.07	1	0	K.GILGYTEDEVVSQDFVHDNR.S (Ions score 148)
275 - 294	1147.04	2292.07	2292.07	1	0	K.GILGYTEDEVVSQDFVHDNR.S (Ions score 141)
275 - 294	765.03	2292.07	2292.07	1	0	K.GILGYTEDEVVSQDFVHDNR.S (Ions score 151)
302 - 312	605.32	1208.62	1208.62	-1	0	K.AGLALNDNFFK.L (Ions score 43)
302 - 312	605.32	1208.62	1208.62	-1	0	K.AGLALNDNFFK.L (Ions score 43)
302 - 312	605.32	1208.62	1208.62	0	0	K.AGLALNDNFFK.L (Ions score 55)
313 - 326	894.90	1787.79	1787.79	-1	0	K.LVSWYDNEWGYSNR.V (Ions score 87)
313 - 326	894.90	1787.79	1787.79	-1	0	K.LVSWYDNEWGYSNR.V (Ions score 80)
313 - 326	894.90	1787.79	1787.79	-1	0	K.LVSWYDNEWGYSNR.V (Ions score 78)
327 - 337	604.83	1207.65	1207.66	-3	0	R.VLDLAVHITNN.- (Ions score 11)
327 - 337	604.83	1207.65	1207.66	-2	0	R.VLDLAVHITNN.- (Ions score 19)
327 - 337	604.83	1207.66	1207.66	-1	0	R.VLDLAVHITNN.- (Ions score 7)

Sample name: 2

Protein Information

Protein name:	Enolase OS=Plasmodium falciparum Vietnam Oak-Knoll (FVO) GN=PFFVO_02844 PE=3 SV=1
Entry name:	A0A024V5N5_PLAFA
Calculated MW:	48989
Calculated pI:	6.21
Mascot score:	748
Sequence coverage:	28%
Bioinformatic tools:	1: UniProt Entry 2: Conserved Domains in NCBI

Analysis Information

- Enzyme: Trypsin
- Variable modifications: Oxidation (M)
- Fixed modifications: Carbamidomethyl (C)
- Database search program: Mascot version 2.4
- Peptide Tolerance: 10 ppm
- Database: UniprotTREMBL (50011027 protein sequences)

Protein sequence

Matched peptides shown in bold underline

```
  1 MAHVITRINA REILDSRGNP TVEVDLETNL GIFRAAVPSG ASTGIYEALE
 51 LRDNDKSRYL GKGVQKAIKN INEIIAPKLI GMNCTEQKKI DNLMVEELDG
101 SKNEWGWSKS KLGANAILAI SMAVCRAGAA ANKVSLYKYL AQLAGKKSDQ
151 MVLPVPCLNV INGGSHAGNK LSFQEFMIVP VGAPSFKEAL RYGAEVYHTL
201 KSEIKKKYGI DATNVGDEGG FAPNILNANE ALDLLVTAIK SAGYEGKVKI
251 AMDVAASEFY NSENKTYDLD FKTPNNDKSL VKTGAQLVDL YIDLVKKYPI
301 VSIEDPFDQD DWENYAKLTA AIGKDVQIVG DDLLVTNPTR ITKALEKNAC
351 NALLLKVNQI GSITEAIEAC LLSQKNNWGV MVSHRSGETE DVFIADLVVA
401 LRTGQIKTGA PCRSERNAKY NQLLRIEESL GNNAVFAGEK FRLQLN
```

Peptides used for identification

Peptides shown in bold have been analysed by MS/MS sequencing

Start - End	Observed	Mr(expt)	Mr(calc)	Delta	Miss	Sequence
35 - 52	902.98	1803.94	1803.94	0	0	**R.AAVPSGASTGIYEALELR.D (Ions score 94)**
171 - 187	949.00	1895.98	1895.99	-1	0	**K.LSFQEFMIVPVGAPSFK.E (Ions score 37)**
171 - 187	957.00	1911.98	1911.98	0	0	**K.LSFQEFMIVPVGAPSFK.E + Oxidation (M) (Ions score 88)**
171 - 187	957.00	1911.98	1911.98	2	0	**K.LSFQEFMIVPVGAPSFK.E + Oxidation (M) (Ions score 78)**
283 - 296	774.43	1546.85	1546.86	-4	0	**K.TGAQLVDLYIDLVK.K (Ions score 17)**
283 - 296	774.44	1546.86	1546.86	-1	0	**K.TGAQLVDLYIDLVK.K (Ions score 66)**
283 - 296	774.44	1546.86	1546.86	0	0	**K.TGAQLVDLYIDLVK.K (Ions score 45)**

Alphalyse A/S ■ Unsbjergvej 4 ■ 5220 Odense ■ Denmark
Tel: +45 6310 6500 ■ e-mail info@alphalyse.com
www.alphalyse.com

297 - 317	858.07	2571.18	2571.18	0	1	K.KYPIVSIEDPFDQDDWENYAK.L (Ions score 57)
297 - 317	858.07	2571.18	2571.18	1	1	K.KYPIVSIEDPFDQDDWENYAK.L (Ions score 47)
318 - 340	803.78	2408.32	2408.33	-1	1	K.LTAAIGKDVQIVGDDLLVTNPTR.I (Ions score 96)
318 - 340	803.78	2408.33	2408.33	-1	1	K.LTAAIGKDVQIVGDDLLVTNPTR.I (Ions score 115)
318 - 340	803.78	2408.33	2408.33	0	1	K.LTAAIGKDVQIVGDDLLVTNPTR.I (Ions score 77)
325 - 340	877.97	1753.92	1753.92	-1	0	K.DVQIVGDDLLVTNPTR.I (Ions score 97)
325 - 340	877.97	1753.92	1753.92	0	0	K.DVQIVGDDLLVTNPTR.I (Ions score 95)
325 - 340	877.97	1753.92	1753.92	0	0	K.DVQIVGDDLLVTNPTR.I (Ions score 98)
357 - 375	692.03	2073.08	2073.08	0	0	K.VNQIGSITEAIEACLLSQK.N (Ions score 51)
426 - 440	789.40	1576.78	1576.77	2	0	R.IEESLGNNAVFAGEK.F (Ions score 92)
426 - 440	789.40	1576.78	1576.77	3	0	R.IEESLGNNAVFAGEK.F (Ions score 99)
426 - 442	627.65	1879.94	1879.94	-2	1	R.IEESLGNNAVFAGEKFR.L (Ions score 81)
426 - 442	627.65	1879.94	1879.94	0	1	R.IEESLGNNAVFAGEKFR.L (Ions score 73)

Sample name: 2

Protein Information

**Protein name:**	**Elongation factor 1-alpha OS=Plasmodium falciparum Vietnam Oak-Knoll (FVO) GN=PFFVO_04465 PE=3 SV=1**
Entry name:	A0A024V296_PLAFA
Calculated MW:	49156
Calculated pI:	9.12
Mascot score:	669
Sequence coverage:	37%
Bioinformatic tools:	1: UniProt Entry 2: Conserved Domains in NCBI

Analysis Information

- Enzyme: Trypsin
- Variable modifications: Oxidation (M)
- Fixed modifications: Carbamidomethyl (C)
- Database search program: Mascot version 2.4
- Peptide Tolerance: 10 ppm
- Database: UniprotTREMBL (50011027 protein sequences)

Protein sequence

Matched peptides shown in bold underline

1 MGKEKTHINL VVIGHVDSGK STTTGHIIYK LGGIDRRTIE KFEKESAEMG
51 KGSFKYAWVL DKLKAERERG ITIDIALWKF ETPR**YFFTVI** **DAPGHKDFIK**
101 NMITGTSQAD VALLVVPAEV GGFEGAFSKE GQTKEHALLA FTLGVK**QIVV**
151 **GVNKMDTVK**Y SEDRYEEIKK EVKDYLKK**VG** **YQADKVDFIP** **ISGFEGDNLI**
201 **EK**SDKTPWYK GRTLIEALDT MEPPKRPYDK PLR**IPLQGVY** **KIGGIGTVPV**
251 **GR**VETGILK**A** **GMVLNFAPSA** **VVSECKSVEM** **HKEVLEEARP** **GDNIGFNVK**N
301 VSVKEIKRGY VASDTKNEPA KGCSK**FTAQV** **IILNHPGEIK** NGYTPVLDCH
351 TSHISCKFLN IDSKIDKRSG KVVEENPKAI K**SGDSALVSL** **EPKKPMVVET**
401 **FTEYPPLGR**F AIRDMR**QTIA** **VGIIK**SVEKK EPGAVTAKAP AKK

Peptides used for identification

Peptides shown in bold have been analysed by MS/MS sequencing

Start - End	Observed	Mr(expt)	Mr(calc)	Delta	Miss	Sequence
85 - 100	633.33	1896.98	1896.98	-1	1	**R.YFFTVIDAPGHKDFIK.N (Ions score 47)**
85 - 100	633.33	1896.98	1896.98	0	1	**R.YFFTVIDAPGHKDFIK.N (Ions score 51)**
147 - 159	723.90	1445.79	1445.79	-3	1	**K.QIVVGVNKMDTVK.Y + Oxidation (M) (Ions score 13)**
147 - 159	723.90	1445.79	1445.79	1	1	**K.QIVVGVNKMDTVK.Y + Oxidation (M) (Ions score 10)**
179 - 202	885.45	2653.33	2653.33	0	1	**K.VGYQADKVDFIPISGFEGDNLIEK.S (Ions score 65)**
179 - 202	885.45	2653.33	2653.33	0	1	**K.VGYQADKVDFIPISGFEGDNLIEK.S (Ions score 118)**
179 - 202	885.45	2653.33	2653.33	2	1	**K.VGYQADKVDFIPISGFEGDNLIEK.S (Ions score 104)**

234 - 241	459.28	916.54	916.54	-2	0	R.IPLQGVYK.I (Ions score 15)
234 - 241	459.28	916.54	916.54	-2	0	R.IPLQGVYK.I (Ions score 12)
234 - 241	459.28	916.54	916.54	-1	0	R.IPLQGVYK.I (Ions score 13)
242 - 252	513.31	1024.60	1024.60	-3	0	K.IGGIGTVPVGR.V (Ions score 63)
242 - 252	513.31	1024.60	1024.60	-2	0	K.IGGIGTVPVGR.V (Ions score 63)
242 - 252	513.31	1024.60	1024.60	-1	0	K.IGGIGTVPVGR.V (Ions score 64)
260 - 276	898.44	1794.86	1794.86	-1	0	K.AGMVLNFAPSAVVSECK.S + Oxidation (M) (Ions score 107)
260 - 276	898.44	1794.86	1794.86	0	0	K.AGMVLNFAPSAVVSECK.S + Oxidation (M) (Ions score 93)
277 - 299	654.32	2613.27	2613.29	-7	1	K.SVEMHKEVLEEARPGDNIGFNVK.N + Oxidation (M) (Ions score 21)
277 - 299	872.10	2613.28	2613.29	-2	1	K.SVEMHKEVLEEARPGDNIGFNVK.N + Oxidation (M) (Ions score 29)
283 - 299	629.66	1885.95	1885.95	-3	0	K.EVLEEARPGDNIGFNVK.N (Ions score 41)
283 - 299	629.66	1885.95	1885.95	-2	0	K.EVLEEARPGDNIGFNVK.N (Ions score 21)
283 - 299	629.66	1885.95	1885.95	-1	0	K.EVLEEARPGDNIGFNVK.N (Ions score 51)
326 - 340	560.65	1678.94	1678.94	-2	0	K.FTAQVIILNHPGEIK.N (Ions score 75)
326 - 340	840.48	1678.94	1678.94	-1	0	K.FTAQVIILNHPGEIK.N (Ions score 72)
326 - 340	560.65	1678.94	1678.94	-1	0	K.FTAQVIILNHPGEIK.N (Ions score 75)
382 - 393	601.82	1201.62	1201.62	-2	0	K.SGDSALVSLEPK.K (Ions score 62)
382 - 393	601.82	1201.62	1201.62	-2	0	K.SGDSALVSLEPK.K (Ions score 74)
382 - 393	601.82	1201.62	1201.62	-1	0	K.SGDSALVSLEPK.K (Ions score 49)
394 - 409	627.32	1878.95	1878.96	-2	0	K.KPMVVETFTEYPPLGR.F + Oxidation (M) (Ions score 57)
394 - 409	627.32	1878.95	1878.96	-1	0	K.KPMVVETFTEYPPLGR.F + Oxidation (M) (Ions score 53)
417 - 425	471.80	941.59	941.59	-3	0	R.QTIAVGIIK.S (Ions score 15)
417 - 425	471.80	941.59	941.59	-2	0	R.QTIAVGIIK.S (Ions score 12)
417 - 425	471.80	941.59	941.59	-1	0	R.QTIAVGIIK.S (Ions score 8)

Sample name: 2

Protein Information

**Protein name:**	**Phosphoglycerate kinase OS=Plasmodium falciparum Vietnam Oak-Knoll (FVO) GN=PFFVO_02527 PE=3 SV=1**
Entry name:	A0A024V742_PLAFA
Calculated MW:	45569
Calculated pI:	7.63
Mascot score:	606
Sequence coverage:	30%
Bioinformatic tools:	1: UniProt Entry 2: Conserved Domains in NCBI

Analysis Information

- Enzyme: Trypsin
- Variable modifications: Oxidation (M)
- Fixed modifications: Carbamidomethyl (C)
- Database search program: Mascot version 2.4
- Peptide Tolerance: 10 ppm
- Database: UniprotTREMBL (50011027 protein sequences)

Protein sequence

Matched peptides shown in bold underline

```
  1 MLGNKLSISD LKDIKNKKVL VRVDFNVPIE NGIIKDTNRI TATLPTINHL
 51 KKEGASKIIL ISHCGRPDGL RNEKYTLKPV AETLKGLLGE EVLFLNDCVG
101 KEVEDKINAA KENSVILLEN LRFHIEEEGK GVDANGNKVK ANKEDVEKFQ
151 NDLTKLADVF INDAFGTAHR AHSSMVGVKL NVKASGFLMK KELEYFSKAL
201 ENPQRPLLAI LGGAKVSDKI QLIKNLLDKV DRMIIGGGMA YTFKKVLNNM
251 KIGTSLFDEA GSKIVGEIME KAKAKNVQIF LPVDFKIADN FDNNANTKFV
301 TDEEGIPDNW MGLDAGPKSI ENYKDVILTS KTVIWNGPQG VFEMPNFAKG
351 SIECLNLVVE VTKKGAITIV GGGDTASLVE QQNKKNEISH VSTGGGASLE
401 LLEGKELPGV LALSNK
```

Peptides used for identification

Peptides shown in bold have been analysed by MS/MS sequencing

Start - End	Observed	Mr(expt)	Mr(calc)	Delta	Miss	Sequence
6 - 15	566.33	1130.65	1130.65	-4	1	**K.LSISDLKDIK.N (Ions score 21)**
23 - 35	729.40	1456.79	1456.79	-3	0	**R.VDFNVPIENGIIK.D (Ions score 29)**
23 - 35	729.40	1456.79	1456.79	-1	0	**R.VDFNVPIENGIIK.D (Ions score 53)**
23 - 39	648.68	1943.01	1943.01	-1	1	**R.VDFNVPIENGIIKDTNR.I (Ions score 92)**
40 - 52	483.96	1448.86	1448.87	-6	1	**R.ITATLPTINHLKK.E (Ions score 31)**
40 - 52	483.96	1448.87	1448.87	0	1	**R.ITATLPTINHLKK.E (Ions score 5)**
156 - 170	549.61	1645.82	1645.82	-2	0	**K.LADVFINDAFGTAHR.A (Ions score 80)**

Alphalyse A/S ■ Unsbjergvej 4 ■ 5220 Odense ■ Denmark
Tel: +45 6310 6500 ■ e-mail info@alphalyse.com
www.alphalyse.com

199 - 215	587.68	1760.03	1760.03	-2	0	K.ALENPQRPLLAILGGAK.V (Ions score 46)
199 - 215	587.68	1760.03	1760.03	-2	0	K.ALENPQRPLLAILGGAK.V (Ions score 52)
199 - 215	587.69	1760.03	1760.03	2	0	K.ALENPQRPLLAILGGAK.V (Ions score 51)
252 - 263	612.81	1223.60	1223.60	-2	0	K.IGTSLFDEAGSK.I (Ions score 69)
252 - 263	612.81	1223.60	1223.60	-2	0	K.IGTSLFDEAGSK.I (Ions score 75)
252 - 263	612.81	1223.60	1223.60	-1	0	K.IGTSLFDEAGSK.I (Ions score 69)
276 - 286	660.37	1318.73	1318.73	-1	0	K.NVQIFLPVDFK.I (Ions score 46)
276 - 286	660.37	1318.73	1318.73	-1	0	K.NVQIFLPVDFK.I (Ions score 50)
276 - 286	660.37	1318.73	1318.73	0	0	K.NVQIFLPVDFK.I (Ions score 44)
287 - 298	668.81	1335.60	1335.61	-1	0	K.IADNFDNNANTK.F (Ions score 45)
287 - 298	668.81	1335.61	1335.61	1	0	K.IADNFDNNANTK.F (Ions score 49)
332 - 349	1026.01	2050.00	2050.00	-1	0	K.TVIWNGPQGVFEMPNFAK.G + Oxidation (M) (Ions score 104)
332 - 349	1026.01	2050.00	2050.00	0	0	K.TVIWNGPQGVFEMPNFAK.G + Oxidation (M) (Ions score 92)

Alphalyse A/S ■ Unsbjergvej 4 ■ 5220 Odense ■ Denmark
Tel: +45 6310 6500 ■ e-mail info@alphalyse.com
www.alphalyse.com

Sample name: 2

Protein Information

**Protein name:**	**Merozoite surface protein 1 (Fragment) OS=Plasmodium falciparum GN=MSP-1 PE=2 SV=1**
Entry name:	Q8T6A9_PLAFA
Calculated MW:	75823
Calculated pI:	7.26
Mascot score:	467
Sequence coverage:	21%
Bioinformatic tools:	1: UniProt Entry 2: Conserved Domains in NCBI

Analysis Information

- Enzyme: Trypsin
- Variable modifications: Oxidation (M)
- Fixed modifications: Carbamidomethyl (C)
- Database search program: Mascot version 2.4
- Peptide Tolerance: 10 ppm
- Database: UniprotTREMBL (50011027 protein sequences)

Protein sequence

Matched peptides shown in bold underline

1 **VTHESYQELV** **KKLEALEDAV** **LTGYSLFQK**E K**MVLNEGTSG** **TAVTTSTPGS**
51 **KGSVASGGSG** **GSVASGGSVA** **SGGSVASGGS** **VASGGSGNSR** RTNPSDNSSD
101 SDAKSYADLK HRVRNYLLTI KELKYPQLFD LTNHMLTLCD NIHGFKYLID
151 GYEEINELLY K**LNFYFDLLR** AKLNDVCAND YCQIPFNLKI RANELDVLKK
201 LVFGYRKPLD NIKDNVGKME DYIKKNKK**TI** **ENINELIEES** **KK**TIDKNKNA
251 TKEEEKKK**LY** **QAQYDLSIYN** **K**QLEEAHNLI SVLEKRIDTL KKNENIKELL
301 DKINEIKNPP PANSGNTPNT LLDKNKKIEE HEKEIKEIAK TIKFNIDSLF
351 TDPLELEYYL REKNKNIDIS AKVETKESTE PNEYPNGVTY PLSYNDINNA
401 LNELNSFGDL INPFDYTKEP SKNIYTDNER KKFINEIKEK IKIEKKKIES
451 DKKSYEDRSK SLNDITKEYE K**LLNEIYDSK** FNNNIDLTNF EKMMGKRYSY
501 KVEKLTHHNT FASYENSKHN LEKLTKALKY MEDYSLRNIV VEKELKYYKN
551 LISKIENEIE TLVENIKKDE EQLFEKKITK DENKPDEK**IL** **EVSDIVK**VQV
601 QKVLLMNKID ELKKTQLILK NVELKHNIHV PNSYKQENKQ EPYYLIVLKK
651 EIDKLK

Peptides used for identification

Peptides shown in bold have been analysed by MS/MS sequencing

Start - End Observed Mr(expt) Mr(calc) Delta Miss Sequence

1 - 11	666.84	1331.67	1331.67	-3	0	-.VTHESYQELVK.K (Ions score 16)
1 - 11	666.84	1331.67	1331.67	0	0	-.VTHESYQELVK.K (Ions score 25)
12 - 29	675.70	2024.08	2024.08	-1	1	K.KLEALEDAVLTGYSLFQK.E (Ions score 44)
12 - 29	675.70	2024.08	2024.08	0	1	K.KLEALEDAVLTGYSLFQK.E (Ions score 75)
12 - 29	675.70	2024.09	2024.08	2	1	K.KLEALEDAVLTGYSLFQK.E (Ions score 60)
32 - 51	977.47	1952.94	1952.94	0	0	K.MVLNEGTSGTAVTTSTPGSK.G + Oxidation (M) (Ions score 103)
32 - 51	977.48	1952.94	1952.94	0	0	K.MVLNEGTSGTAVTTSTPGSK.G + Oxidation (M) (Ions score 115)
32 - 51	977.48	1952.94	1952.94	1	0	K.MVLNEGTSGTAVTTSTPGSK.G + Oxidation (M) (Ions score 94)
52 - 90	1023.80	3068.39	3068.40	-3	0	K.GSVASGGSGGSVASGGSVASGGSVASGGSVASGGSGNSR.R (Ions score 52)
162 - 170	600.83	1199.64	1199.63	3	0	K.LNFYFDLLR.A (Ions score 40)
229 - 242	553.96	1658.87	1658.87	-2	1	K.TIENINELIEESKK.T (Ions score 40)
229 - 242	553.96	1658.87	1658.87	-1	1	K.TIENINELIEESKK.T (Ions score 38)
259 - 271	809.91	1617.80	1617.80	-2	0	K.LYQAQYDLSIYNK.Q (Ions score 76)
259 - 271	809.91	1617.80	1617.80	0	0	K.LYQAQYDLSIYNK.Q (Ions score 64)
259 - 271	809.91	1617.80	1617.80	1	0	K.LYQAQYDLSIYNK.Q (Ions score 84)
472 - 480	547.79	1093.56	1093.57	-1	0	K.LLNEIYDSK.F (Ions score 23)
589 - 597	508.30	1014.59	1014.60	-2	0	K.ILEVSDIVK.V (Ions score 13)

Sample name: 2

Protein Information

**Protein name:**	**Hsp90-like protein OS=Plasmodium falciparum Vietnam Oak-Knoll (FVO) GN=PFFVO_01664 PE=3 SV=1**
Entry name:	A0A024V8X4_PLAFA
Calculated MW:	86512
Calculated pI:	4.94
Mascot score:	360
Sequence coverage:	10%
Bioinformatic tools:	1: UniProt Entry 2: Conserved Domains in NCBI

Analysis Information

- Enzyme: Trypsin
- Variable modifications: Oxidation (M)
- Fixed modifications: Carbamidomethyl (C)
- Database search program: Mascot version 2.4
- Peptide Tolerance: 10 ppm
- Database: UniprotTREMBL (50011027 protein sequences)

Protein sequence

Matched peptides shown in bold underline

```
  1 MSTETFAFNA DIRQLMSLII NTFYSNKEIF LRELISNASD ALDKIRYESI
 51 TDTQKLSAEP EFFIRIIPDK TNNTLTIEDS GIGMTKNDLI NNLGTIARSG
101 TKAFMEAIQA SGDISMIGQF GVGFYSAYLV ADHVVVISKN NDDEQYVWES
151 AAGGSFTVTK DETNEKLGRG TKIILHLKED QLEYLEEKRI KDLVKKHSEF
201 ISFPIKLYCE RQNEKEITAS EEEEGEGEGE REGEEEEEKK KKTGEDKNAD
251 ESKEENEDEE KKEDNEEDDN KTDHPKVEDV TEELENAEKK KKEKRKKKIH
301 TVEHEWEELN KQKPLWMRKP EEVTNEEYAS FYKSLTNDWE DHLAVKHFSV
351 EGQLEFKALL FIPKRAPFDM FENRKKRNNI KLYVRRVFIM DDCEEIIPEW
401 LNFVKGVVDS EDLPLNISRE SLQQNKILKV IKKNLIKKCL DMFSELAENK
451 ENYKKFYEQF SKNLKLGIHE DNANRTKITE LLRFQTSKSG DEMIGLKEYV
501 DRMKENQKDI YYITGESINA VSNSPFLEAL TKKGFEVIYM VDPIDEYAVQ
551 QLKDFDGKKL KCCTKEGLDI DDSEEAKKDF ETLKAEYEGL CKVIKDVLHE
601 KVEKVVVGQR ITDSPCVLVT SEFGWSANME RIMKAQALRD NSMTSYMLSK
651 KIMEINARHP IISALKQKAD ADKSDKTVKD LIWLLFDTSL LTSGFALEEP
701 TTFSKRIHRM IKLGLSIDEE ENNDIDLPPL EETVDATDSK MEEVD
```

Matched (bold underlined) segments: LSAEP EFFIR (56–65), NDLI NNLGTIAR (87–98), HSEF ISFPIK (197–206), SLTNDWE DHLAVK (334–346), GVVDS EDLPLNISR (406–419), KGFEVIYM VDPIDEYAVQ QLK (533–553).

Peptides used for identification

Peptides shown in bold have been analysed by MS/MS sequencing

Alphalyse A/S ■ Unsbjergvej 4 ■ 5220 Odense ■ Denmark
Tel: +45 6310 6500 ■ e-mail info@alphalyse.com
www.alphalyse.com

Start - End	Observed	Mr(expt)	Mr(calc)	Delta	Miss	Sequence
56 - 65	604.82	1207.62	1207.62	-2	0	K.LSAEPEFFIR.I (Ions score 61)
56 - 65	604.82	1207.62	1207.62	-1	0	K.LSAEPEFFIR.I (Ions score 65)
56 - 65	604.82	1207.62	1207.62	-1	0	K.LSAEPEFFIR.I (Ions score 65)
87 - 98	657.36	1312.71	1312.71	-3	0	K.NDLINNLGTIAR.S (Ions score 42)
87 - 98	657.36	1312.71	1312.71	-1	0	K.NDLINNLGTIAR.S (Ions score 51)
197 - 206	602.82	1203.63	1203.63	-1	0	K.HSEFISFPIK.L (Ions score 38)
334 - 346	764.37	1526.73	1526.74	-1	0	K.SLTNDWEDHLAVK.H (Ions score 52)
334 - 346	764.38	1526.74	1526.74	2	0	K.SLTNDWEDHLAVK.H (Ions score 50)
406 - 419	757.40	1512.78	1512.78	-1	0	K.GVVDSEDLPLNISR.E (Ions score 85)
406 - 419	757.40	1512.78	1512.78	0	0	K.GVVDSEDLPLNISR.E (Ions score 81)
406 - 419	757.40	1512.78	1512.78	0	0	K.GVVDSEDLPLNISR.E (Ions score 80)
533 - 553	834.42	2500.25	2500.26	-1	1	K.KGFEVIYMVDPIDEYAVQQLK.D + Oxidation (M) (Ions score 69)
533 - 553	834.43	2500.26	2500.26	0	1	K.KGFEVIYMVDPIDEYAVQQLK.D + Oxidation (M) (Ions score 62)
533 - 553	834.43	2500.26	2500.26	2	1	K.KGFEVIYMVDPIDEYAVQQLK.D + Oxidation (M) (Ions score 48)

Alphalyse A/S ■ Unsbjergvej 4 ■ 5220 Odense ■ Denmark
Tel: +45 6310 6500 ■ e-mail info@alphalyse.com
www.alphalyse.com

Sample name: 2

Protein Information

**Protein name:**	**Hsp70-like protein OS=Plasmodium falciparum Vietnam Oak-Knoll (FVO) GN=PFFVO_02131 PE=3 SV=1**
Entry name:	A0A024V7B6_PLAFA
Calculated MW:	74724
Calculated pI:	5.51
Mascot score:	344
Sequence coverage:	9%
Bioinformatic tools:	1: UniProt Entry 2: Conserved Domains in NCBI

Analysis Information

- Enzyme: Trypsin
- Variable modifications: Oxidation (M)
- Fixed modifications: Carbamidomethyl (C)
- Database search program: Mascot version 2.4
- Peptide Tolerance: 10 ppm
- Database: UniprotTREMBL (50011027 protein sequences)

Protein sequence

Matched peptides shown in bold underline

```
  1 MASAKGSKPN LPESNIAIGI DLGTTYSCVG VWRNENVDII ANDQGNRTTP
 51 SYVAFTDTER LIGDAAKNQV ARNPENTVFD AKRLIGRKFT ESSVQSDMKH
101 WPFTVKSGVD EKPMIEVTYQ GEKKLFHPEE ISSMVLQKMK ENAEAFLGKS
151 IKNAVITVPA YFNDSQRQAT KDAGTIAGLN VMRIINEPTA AAIAYGLHKK
201 GKGEKNILIF DLGGGTFDVS LLTIEDGIFE VKATAGDTHL GGEDFDNRLV
251 NFCVEDFKRK NRGKDLSKNS RALRRLRTQC ERAKRTLSSS TQATIEIDSL
301 FEGIDYSVTV SRARFEELCI DYFRDTLIPV EKVLKDAMMD KKSVHEVVLV
351 GGSTRIPKIQ TLIKEFFNGK EACRSINPDE AVAYGAAVQA AILSGDQSNA
401 VQDLLLLDVC SLSLGLETAG GVMTKLIERN TTIPAKKSQI FTTYADNQPG
451 VLIQVYEGER ALTKDNNLLG KFHLDGIPPA PRKVPQIEVT FDIDANGILN
501 VTAVEKSTGK QNHITITNDK GRLSQDEIDR MVNDAEKYKA EDEENRKRIE
551 ARNSLENYCY GVKSSLEDQK IKEKLQPAEI ETCMKTITTI LEWLEKNQLA
601 GKDEYEAKQK EAESVCAPIM SKIYQDAAGA AGGMPGGMPG GMPGGMPGGM
651 PGGMNFPGGM PGAGMPGNAP AGSGPTVEEV D
```

Matched peptides (bold underline): TTP SYVAFTDTER, NPENTVFD AKR, NAVITVPA YFNDSQR, DAGTIAGLN VMRIINEPTA AAIAYGLHK

Peptides used for identification

Peptides shown in bold have been analysed by MS/MS sequencing

Start - End Observed Mr(expt) Mr(calc) Delta Miss Sequence

Alphalyse A/S ■ Unsbjergvej 4 ■ 5220 Odense ■ Denmark
Tel: +45 6310 6500 ■ e-mail info@alphalyse.com
www.alphalyse.com

48 - 60	744.35	1486.69	1486.69	0	0	R.TTPSYVAFTDTER.L (Ions score 74)
73 - 83	645.83	1289.64	1289.64	0	1	R.NPENTVFDAKR.L (Ions score 13)
73 - 83	645.83	1289.64	1289.64	1	1	R.NPENTVFDAKR.L (Ions score 71)
153 - 167	847.93	1693.84	1693.84	-1	0	K.NAVITVPAYFNDSQR.Q (Ions score 64)
153 - 167	847.93	1693.84	1693.84	-1	0	K.NAVITVPAYFNDSQR.Q (Ions score 61)
153 - 167	847.93	1693.84	1693.84	1	0	K.NAVITVPAYFNDSQR.Q (Ions score 59)
172 - 183	617.32	1232.62	1232.62	-1	0	K.DAGTIAGLNVMR.I + Oxidation (M) (Ions score 55)
172 - 183	617.32	1232.62	1232.62	0	0	K.DAGTIAGLNVMR.I + Oxidation (M) (Ions score 46)
172 - 183	617.32	1232.62	1232.62	0	0	K.DAGTIAGLNVMR.I + Oxidation (M) (Ions score 48)
184 - 199	561.31	1680.92	1680.92	-2	0	R.IINEPTAAAIAYGLHK.K (Ions score 78)
184 - 199	561.31	1680.92	1680.92	0	0	R.IINEPTAAAIAYGLHK.K (Ions score 81)

LC-MSMS Protein Identification Report

Order 16372_Plasmodium falciparum

Sample name: 2

Protein Information

**Protein name:**	**S-adenosylmethionine synthase OS=Plasmodium falciparum Vietnam Oak-Knoll (FVO) GN=PFFVO_02524 PE=3 SV=1**
Entry name:	A0A024V6I4_PLAFA
Calculated MW:	45272
Calculated pI:	6.28
Mascot score:	333
Sequence coverage:	15%
Bioinformatic tools:	1: UniProt Entry 2: Conserved Domains in NCBI

Analysis Information

- Enzyme: Trypsin
- Variable modifications: Oxidation (M)
- Fixed modifications: Carbamidomethyl (C)
- Database search program: Mascot version 2.4
- Peptide Tolerance: 10 ppm
- Database: UniprotTREMBL (50011027 protein sequences)

Protein sequence

Matched peptides shown in bold underline

```
  1 MSQLKIKRGN FLFTSESVNE GHPDKICDQI SDAILDSCLR EDPYSKVACE
 51 VCAKKNYIFI FGEITTKAKV NYDKVTRDVL KHIGYDDESK GLDYKTAEIK
101 VSIDEQSPDI AQCVHENRSP ELIGAGDQGI MFGYATDETE NYMPLTHHYA
151 TLLGKRLTEV RKLGILPYLG PDGKTQITIE YKNKGSCGGH LEPLRVHTVL
201 ISTQHAEDIK YEQLKTDLME NVIKYVIPEK LLDNETLYYL NPSGKFVLGG
251 PAADAGLTGR KIICDTYGGW GAHGGGAFSG KDASKVDRSA AYYLRFIAKS
301 LVANKFCRRV LVQASYSIGI ANPISLNVNS YGTVSTGYTD YDLEQIILRN
351 FDLRPGFIIQ ELKLTEPVFS KTSAYGHFGR EGDTFTWEKI KDLSHEKNAL
401 KN
```

Peptides used for identification

Peptides shown in bold have been analysed by MS/MS sequencing

Start - End	Observed	Mr(expt)	Mr(calc)	Delta	Miss	Sequence
163 - 174	621.86	1241.70	1241.70	-1	0	**K.LGILPYLGPDGK.T** (Ions score 48)
163 - 174	621.86	1241.70	1241.70	-1	0	**K.LGILPYLGPDGK.T** (Ions score 56)
163 - 174	621.86	1241.70	1241.70	-1	0	**K.LGILPYLGPDGK.T** (Ions score 56)
225 - 245	823.77	2468.28	2468.28	-2	1	**K.YVIPEKLLDNETLYYLNPSGK.F** (Ions score 38)
231 - 245	870.45	1738.88	1738.88	-1	0	**K.LLDNETLYYLNPSGK.F** (Ions score 96)
246 - 260	701.38	1400.74	1400.74	-2	0	**K.FVLGGPAADAGLTGR.K** (Ions score 112)
246 - 260	701.38	1400.74	1400.74	-1	0	**K.FVLGGPAADAGLTGR.K** (Ions score 115)

Alphalyse A/S ■ Unsbjergvej 4 ■ 5220 Odense ■ Denmark
Tel: +45 6310 6500 ■ e-mail info@alphalyse.com
www.alphalyse.com

350 - 363	845.47	1688.92	1688.93	0	0	R.NFDLRPGFIIQELK.L (Ions score 27)

Sample name: 2

Protein Information

**Protein name:**	**Uncharacterized protein OS=Plasmodium falciparum Vietnam Oak-Knoll (FVO) GN=PFFVO_03482 PE=3 SV=1**
Entry name:	A0A024V542_PLAFA
Calculated MW:	95301
Calculated pI:	5.28
Mascot score:	312
Sequence coverage:	6%
Bioinformatic tools:	1: UniProt Entry 2: Conserved Domains in NCBI

Analysis Information

- Enzyme: Trypsin
- Variable modifications: Oxidation (M)
- Fixed modifications: Carbamidomethyl (C)
- Database search program: Mascot version 2.4
- Peptide Tolerance: 10 ppm
- Database: UniprotTREMBL (50011027 protein sequences)

Protein sequence

Matched peptides shown in bold underline

1 MKLNNIYSFF FLFFVLCVIQ ENVRRVLCDS SVEGDKGPSD DVSDSSGEKK
51 EVKRDRDTLE EIEEGEKPTE SMESHQYQTE VTRLMDIIVN SLYTQKEVFL
101 RELISNAADA LEKIRFLSLS DESVLGEEKK LEIRISANKE KNILSITDTG
151 IGMTK**VDLIN NLGTIAK**SGT SNFLEAISKS GGDMSLIGQF GVGFYSAFLV
201 ADKVIVYTKN NDDEQYIWES TADAKFTIYK DPRGATLKRG TRISLHLKED
251 ATNLLNDKKL MDLISKYSQF IQFPIYLLHE NVYTEEVLAD IAKDMVNDPN
301 YDSVKVEETD DPNKKTRTVE KKVKKWTLMN EQRPIWLRSP KELKDEDYKQ
351 FFSVLSGYND QPLYHIHFFA EGEIEFKCLI YIPSKAPSMN DQLYSKQNSL
401 KLYVRR**VLVA DEFVEFLPR**Y MSFVK**GVVDS DDLPLNVSR**E QLQQNKILKA
451 VSKRIVRKIL DTFHKLYKEG KKNKETLRSE LENETDEEKK KEITKKLSEP
501 STYKLIYKEY RKFLKSGCYE DDINRNKIAK LLLFKTMQYP KSISLDTYIE
551 HMKPDQKFIY YASGDSYEYL AKIPQLQIFK KKNIDVLFLT ESVDESCIQR
601 VQEYEGKKFK SIQKGEISFE LTEEEKKKEQ QMQKMYK**ALI DVISDTLK**NQ
651 IFKVEISRRL VDAPCAVVST EWGLSGQMEK LMKMNVSNSD QIKAMSGQKI
701 LEINPNHPIM IDLLKRSVTN PKDLELTNSI KIMYQSAKLA SGFDLEDTAD
751 LAQIVYDHIN QKLGVDNNLK IDDLDPSIFE TKKIEDENDS SKFEEEINID
801 DEIQKKDNNV DNESNDKSDE L

Alphalyse A/S ■ Unsbjergvej 4 ■ 5220 Odense ■ Denmark
Tel: +45 6310 6500 ■ e-mail info@alphalyse.com
www.alphalyse.com

Peptides used for identification

Peptides shown in bold have been analysed by MS/MS sequencing

Start - End	Observed	Mr(expt)	Mr(calc)	Delta	Miss	Sequence
156 - 167	635.87	1269.73	1269.73	0	0	**K.VDLINNLGTIAK.S** (Ions score 67)
407 - 419	767.42	1532.82	1532.82	-1	0	**R.VLVADEFVEFLPR.Y** (Ions score 73)
407 - 419	767.42	1532.82	1532.82	0	0	**R.VLVADEFVEFLPR.Y** (Ions score 78)
407 - 419	767.42	1532.82	1532.82	0	0	**R.VLVADEFVEFLPR.Y** (Ions score 80)
407 - 419	767.42	1532.82	1532.82	0	0	**R.VLVADEFVEFLPR.Y** (Ions score 101)
426 - 439	743.38	1484.75	1484.75	-1	0	**K.GVVDSDDLPLNVSR.E** (Ions score 76)
426 - 439	743.38	1484.75	1484.75	0	0	**K.GVVDSDDLPLNVSR.E** (Ions score 72)
426 - 439	743.38	1484.75	1484.75	0	0	**K.GVVDSDDLPLNVSR.E** (Ions score 87)
638 - 648	594.35	1186.68	1186.68	-5	0	**K.ALIDVISDTLK.N** (Ions score 45)
638 - 648	594.35	1186.68	1186.68	1	0	**K.ALIDVISDTLK.N** (Ions score 58)

Alphalyse A/S ■ Unsbjergvej 4 ■ 5220 Odense ■ Denmark
Tel: +45 6310 6500 ■ e-mail info@alphalyse.com
www.alphalyse.com

LC-MSMS Protein Identification Report

Order 16372_Plasmodium falciparum

Sample name: 2

Protein Information

Protein name:	**Pyruvate kinase OS=Plasmodium falciparum Vietnam Oak-Knoll (FVO) GN=PFFVO_01529 PE=3 SV=1**
Entry name:	A0A024VAV7_PLAFA
Calculated MW:	56480
Calculated pI:	7.5
Mascot score:	294
Sequence coverage:	16%
Bioinformatic tools:	1: UniProt Entry 2: Conserved Domains in NCBI

Analysis Information

- Enzyme: Trypsin
- Variable modifications: Oxidation (M)
- Fixed modifications: Carbamidomethyl (C)
- Database search program: Mascot version 2.4
- Peptide Tolerance: 10 ppm
- Database: UniprotTREMBL (50011027 protein sequences)

Protein sequence

Matched peptides shown in bold underline

```
  1 MSSFKYKNSA AGASMQSAAN ITLRQILEPN NVNLRSKKTH IVCTLGPACK
 51 SVETLVKLID AGMDICRFNF SHGSHEDHKE MFNNVLKAQE LRPNCLLGML
101 LDTKGPEIRT GFLKNKEVHL KEGSKLKLVT DYEFLGDETC IACSYKKLPQ
151 SVKPGNIILI ADGSVSCKVL ETHEDHVITE VLNSAVIGER KNMNLPNVKV
201 DLPIISEKDK NDILNFAIPM GCNFIAASFI QSADDVRLIR NLLGPRGRHI
251 KIIPKIENIE GIIHFDKILA ESDGIMIARG DLGMEISPEK VFLAQKLMIS
301 KCNLQGKPII TATQMLESMT KNPRPTRAEV TDVANAVLDG TDCVMLSGET
351 AGGKFPVEAV TIMSKICLEA EACIDYKLLY QSLVNAIETP ISVQEAVARS
401 AVETAESIQA SLIIALTETG YTARLIAKYK PSCTILALSA SDSTVKCLNV
451 HRGVTCIKVG SFQGTDIVIR NAIEIAKQRN MAKVGDSVIA IHGIKEEVSG
501 GTNLMKVVQI E
```

Peptides used for identification

Peptides shown in bold have been analysed by MS/MS sequencing

Start - End	Observed	Mr(expt)	Mr(calc)	Delta	Miss	Sequence
25 - 35	655.36	1308.71	1308.72	-2	0	**R.QILEPNNVNLR.S (Ions score 57)**
25 - 35	655.36	1308.71	1308.72	-1	0	**R.QILEPNNVNLR.S (Ions score 48)**
25 - 35	655.37	1308.72	1308.72	1	0	**R.QILEPNNVNLR.S (Ions score 56)**
169 - 190	820.76	2459.26	2459.27	0	0	**K.VLETHEDHVITEVLNSAVIGER.K (Ions score 69)**

Alphalyse A/S ■ Unsbjergvej 4 ■ 5220 Odense ■ Denmark
Tel: +45 6310 6500 ■ e-mail info@alphalyse.com
www.alphalyse.com

302 - 321	766.05	2295.13	2295.13	-1	0	K.CNLQGKPIITATQMLESMTK.N + 2 Oxidation (M) (Ions score 52)
429 - 446	647.67	1939.99	1939.99	-1	0	K.YKPSCTILALSASDSTVK.C (Ions score 33)
459 - 470	646.35	1290.69	1290.69	-2	0	K.VGSFQGTDIVIR.N (Ions score 82)
459 - 470	646.35	1290.69	1290.69	-1	0	K.VGSFQGTDIVIR.N (Ions score 78)
459 - 470	646.35	1290.69	1290.69	0	0	K.VGSFQGTDIVIR.N (Ions score 83)

Sample name: 2

Protein Information

**Protein name:**	**Ornithine aminotransferase OS=Plasmodium falciparum Vietnam Oak-Knoll (FVO) GN=PFFVO_06225 PE=3 SV=1**
Entry name:	A0A024UX71_PLAFA
Calculated MW:	46938
Calculated pI:	6.47
Mascot score:	275
Sequence coverage:	14%
Bioinformatic tools:	1: UniProt Entry 2: Conserved Domains in NCBI

Analysis Information

- Enzyme: Trypsin
- Variable modifications: Oxidation (M)
- Fixed modifications: Carbamidomethyl (C)
- Database search program: Mascot version 2.4
- Peptide Tolerance: 10 ppm
- Database: UniprotTREMBL (50011027 protein sequences)

Protein sequence

Matched peptides shown in bold underline

1 MDFVKELKSS QDYMNNELTY GAHNYDPIPV VLKRGKGVFV YDIEDRRYYD
51 FLSAYSSVNQ GHCHPDILNA MINQAKKLTI CSR**AFFSDSL** **GVCERYLTNL**
101 **FGYDK**VLMMN TGAEASETAY KLCRKWGYEV KKIPENSAKI IVCNNNFSGR
151 **TLGCVSASTD** **KK**CK**NNFGPF** **VPNFLK**VPYD DLEALEKELQ DPNVCAFIVE
201 PVQGEAGVIV PSDSYFPGVA SLCKK**YNVLF** **VADEVQTGLG** **R**TGKLLCTHH
251 YGVKPDVILL GKALSGGHYP ISAILANDDV MLVLKPGEHG STYGGNPLAA
301 AICVEALKVL INEKLCENAD KLGAPFLQNL KEQLKDSKVV REVRGKGLLC
351 AIEFKNDLVN VWDICLKFKE NGLITRSVHD KTVRLTPPLC ITKEQLDECT
401 EIIVKTVKFF DDNL

Peptides used for identification

Peptides shown in bold have been analysed by MS/MS sequencing

Start - End	Observed	Mr(expt)	Mr(calc)	Delta	Miss	Sequence
84 - 95	694.32	1386.62	1386.62	-2	0	R.**AFFSDSLGVCER**.Y (Ions score 68)
96 - 105	617.31	1232.61	1232.61	0	0	R.**YLTNLFGYDK**.V (Ions score 54)
96 - 105	617.31	1232.61	1232.61	1	0	R.**YLTNLFGYDK**.V (Ions score 62)
151 - 162	633.82	1265.62	1265.63	-4	1	R.**TLGCVSASTDKK**.C (Ions score 13)
165 - 176	697.37	1392.72	1392.72	-1	0	K.**NNFGPFVPNFLK**.V (Ions score 37)
165 - 176	697.37	1392.72	1392.72	-1	0	K.**NNFGPFVPNFLK**.V (Ions score 42)
226 - 241	890.96	1779.91	1779.92	-1	0	K.**YNVLFVADEVQTGLGR**.T (Ions score 70)

226 - 241	890.96	1779.91	1779.92	-1	0	K.YNVLFVADEVQTGLGR.T (Ions score 90)

Alphalyse A/S ■ Unsbjergvej 4 ■ 5220 Odense ■ Denmark
Tel: +45 6310 6500 ■ e-mail info@alphalyse.com
www.alphalyse.com

Sample name: 2

Protein Information

**Protein name:**	**Hypoxanthine-guanine-xanthine phosphoribosyltransferase OS=Plasmodium falciparum Vietnam Oak-Knoll (FVO) GN=PFFVO_02805 PE=4 SV=1**
Entry name:	A0A024V748_PLAFA
Calculated MW:	24632
Calculated pI:	9.05
Mascot score:	272
Sequence coverage:	34%
Bioinformatic tools:	1: UniProt Entry 2: Conserved Domains in NCBI

Analysis Information

- Enzyme: Trypsin
- Variable modifications: Oxidation (M)
- Fixed modifications: Carbamidomethyl (C)
- Database search program: Mascot version 2.4
- Peptide Tolerance: 10 ppm
- Database: UniprotTREMBL (50011027 protein sequences)

Protein sequence

Matched peptides shown in bold underline

1 MCIKQYTYKN ISKLGHKYLT K**VLVPNGVIK** NRIEKLAYDI KKVYNNEEFH
51 ILCLLKGSRG FFTALLKHLS R**IHNYSAVET** **SKPLFGEHYV** **R**VK**SYCNDQS**
101 **TGTLEIVSED** **LSCLK**GK**HVL** **IVEDIIDTGK** TLVKFCEYLK KFEIKTVAIA
151 CLFIKR**TPLW** **NGFK**ADFVGF SIPDHFVVGY SLDYNEIFRD LDHCCLVNDE
201 GKKKYKATSL

Peptides used for identification

Peptides shown in bold have been analysed by MS/MS sequencing

Start - End	Observed	Mr(expt)	Mr(calc)	Delta	Miss	Sequence
22 - 30	469.80	937.59	937.60	-2	0	**K.VLVPNGVIK.N (Ions score 15)**
72 - 91	587.55	2346.17	2346.18	-2	0	**R.IHNYSAVETSKPLFGEHYVR.V (Ions score 31)**
72 - 91	587.55	2346.17	2346.18	-1	0	**R.IHNYSAVETSKPLFGEHYVR.V (Ions score 23)**
72 - 91	783.07	2346.17	2346.18	0	0	**R.IHNYSAVETSKPLFGEHYVR.V (Ions score 81)**
94 - 115	1260.06	2518.11	2518.12	-3	0	**K.SYCNDQSTGTLEIVSEDLSCLK.G (Ions score 56)**
118 - 130	726.41	1450.80	1450.80	0	0	**K.HVLIVEDIIDTGK.T (Ions score 80)**
118 - 130	726.41	1450.80	1450.80	1	0	**K.HVLIVEDIIDTGK.T (Ions score 71)**
157 - 164	481.76	961.50	961.50	-2	0	**R.TPLWNGFK.A (Ions score 22)**
157 - 164	481.76	961.50	961.50	-1	0	**R.TPLWNGFK.A (Ions score 39)**
157 - 164	481.76	961.50	961.50	0	0	**R.TPLWNGFK.A (Ions score 21)**

Alphalyse A/S ■ Unsbjergvej 4 ■ 5220 Odense ■ Denmark
Tel: +45 6310 6500 ■ e-mail info@alphalyse.com
www.alphalyse.com

Sample name: 2

Protein Information

**Protein name:**	**M1 family aminopeptidase OS=Plasmodium falciparum Vietnam Oak-Knoll (FVO) GN=PFFVO_03958 PE=4 SV=1**
Entry name:	A0A024V3R7_PLAFA
Calculated MW:	126553
Calculated pI:	7.3
Mascot score:	248
Sequence coverage:	6%
Bioinformatic tools:	1: UniProt Entry 2: Conserved Domains in NCBI

Analysis Information

- Enzyme: Trypsin
- Variable modifications: Oxidation (M)
- Fixed modifications: Carbamidomethyl (C)
- Database search program: Mascot version 2.4
- Peptide Tolerance: 10 ppm
- Database: UniprotTREMBL (50011027 protein sequences)

Protein sequence

Matched peptides shown in bold underline

1 MKLTKGCAYK YIIFTVLILA NILYDNKKRC MIKKNLRISS CGIISRLLKS
51 NSNYNSFNKN YNFTSAISEL QFSNFWNLDI LQKDIFSNIH NNKNKPQSYI
101 IHKRLMSEKG DNNNNNHQNN NGNDNKKRLG SVVNNEENTC SDKRMKPFEE
151 GHGITQVDKM NNNSDHLQQN GVMNLNSNNV ENNNNNNSVV VKKNEPKIHY
201 RKDYKPSGFI INNVTLNINI HDNETIVRSV LDMDISKHNV GEDLVFDGVG
251 LKINEISINN KKLVEGEEYT YDNEFLTIFS KFVPKSKFAF SSEVIIHPET
301 NYALTGLYKS KNIIVSQCEA TGFRRITFFI DRPDMMAKYD VTVTADKEKY
351 PVLLSNGDKV NEFEIPGGRH GARFNDPHLK PCYLFAVVAG DLKHLSATYI
401 TKYTKKKVEL YVFSEEKYVS KLQWALECLK KSMAFDEDYF GLEYDLSRLN
451 LVAVSDFNVG AMENK**GLNIF NANSLLASK**K NSIDFSYARI LTVVGHEYFH
501 NYTGNRVTLR DWFQLTLKEG LTVHRENLFS EEMTKTVTTR LSHVDLLRSV
551 QFLEDSSPLS HPIRPESYVS MENFYTTTVY DKGSEVMRMY LTILGEEYYK
601 KGFDIYIKKN DGNTATCEDF NYAMEQAYKM KKADNSANLN QYLLWFSQSG
651 TPHVSFKYNY DAEKKQYSIH VNQYTKPDEN QKEKKPLFIP ISVGLINPEN
701 GKEMISQTTL ELTK**ESDTFV FNNIAVKPIP SLFRGFSAPV YIEDNLTDEE**
751 **R**ILLLKYDSD AFVRYNSCTN IYMKQILMNY NEFLKAKNEK **LESFNLTPVN**
801 **AQFIDAIK**YL LEDPHADAGF KSYIVSLPQD RYIINFVSNL DTDVLADTKE
851 YIYKQIGDKL NDVYYKMFKS LEAKADDLTY FNDESHVDFD QMNMRTLRNT
901 LLSLLSKAQY PNILNEIIEH SKSPYPSNWL TSLSVSAYFD KYFELYDKTY

Alphalyse A/S ■ Unsbjergvej 4 ■ 5220 Odense ■ Denmark
Tel: +45 6310 6500 ■ e-mail info@alphalyse.com
www.alphalyse.com

951 KLSKDDELLL QEWLKTVSRS DRKDIYEILK KLENEVLKDS KNPNDIRAVY
1001 LPFTNNLRRF HDISGKGYKL IAEVITKTDK FNPMVATQLC EPFKLWNKLD
1051 TKRQELMLNE MNTMLQEPNI SNNLKEYLLR LTNKL

Peptides used for identification

Peptides shown in bold have been analysed by MS/MS sequencing

Start - End	Observed	Mr(expt)	Mr(calc)	Delta	Miss	Sequence
466 - 479	731.40	1460.79	1460.80	-3	0	**K.GLNIFNANSLLASK.K** (Ions score 34)
466 - 479	731.40	1460.80	1460.80	-3	0	**K.GLNIFNANSLLASK.K** (Ions score 44)
466 - 479	731.41	1460.80	1460.80	-1	0	**K.GLNIFNANSLLASK.K** (Ions score 33)
715 - 734	765.41	2293.21	2293.21	-1	0	**K.ESDTFVFNNIAVKPIPSLFR.G** (Ions score 97)
715 - 734	765.41	2293.21	2293.21	-1	0	**K.ESDTFVFNNIAVKPIPSLFR.G** (Ions score 81)
715 - 734	765.41	2293.21	2293.21	0	0	**K.ESDTFVFNNIAVKPIPSLFR.G** (Ions score 98)
735 - 751	977.96	1953.90	1953.90	1	0	**R.GFSAPVYIEDNLTDEER.I** (Ions score 92)
791 - 808	1010.54	2019.06	2019.07	-3	0	**K.LESFNLTPVNAQFIDAIK.Y** (Ions score 14)
791 - 808	1010.54	2019.07	2019.07	2	0	**K.LESFNLTPVNAQFIDAIK.Y** (Ions score 5)

Sample name: 2

Protein Information

Protein name:	L-lactate dehydrogenase OS=Plasmodium falciparum Vietnam Oak-Knoll (FVO) GN=PFFVO_04104 PE=3 SV=1
Entry name:	A0A024V3C8_PLAFA
Calculated MW:	34314
Calculated pI:	7.12
Mascot score:	230
Sequence coverage:	12%
Bioinformatic tools:	1: UniProt Entry 2: Conserved Domains in NCBI

Analysis Information

- Enzyme: Trypsin
- Variable modifications: Oxidation (M)
- Fixed modifications: Carbamidomethyl (C)
- Database search program: Mascot version 2.4
- Peptide Tolerance: 10 ppm
- Database: UniprotTREMBL (50011027 protein sequences)

Protein sequence

Matched peptides shown in bold underline

1 MAPKAKIVLV GSGMIGGVMA TLIVQK**NLGD VVLFDIVK**NM PHGKALDTSH
51 TNVMAYSNCK VSGSNTYDDL AGADVVIVTA GFTKAPGKSD KEWNRDDLLP
101 LNNKIMIEIG GHIKKNCPNA FIIVVTNPVD VMVQLLHQHS GVPKNK**IIGL**
151 **GGVLDTSR**LK YYISQKLNVC PRDVNAHIVG AHGNKMVLLK R**YITVGGIPL**
201 **QEFINNK**LIS DAELEAIFDR TVNTALEIVN LHASPYVAPA AAIIEMAESY
251 LKDLKKVLIC STLLEGQYGH SDIFGGTPVV LGANGVEQVI ELQLNSEEKA
301 KFDEAIAETK RMKALA

Peptides used for identification

Peptides shown in bold have been analysed by MS/MS sequencing

Start - End	Observed	Mr(expt)	Mr(calc)	Delta	Miss	Sequence
**27 - 38**	**666.38**	**1330.75**	**1330.75**	**-1**	**0**	**K.NLGDVVLFDIVK.N (Ions score 40)**
**27 - 38**	**666.38**	**1330.75**	**1330.75**	**0**	**0**	**K.NLGDVVLFDIVK.N (Ions score 42)**
**27 - 38**	**666.38**	**1330.75**	**1330.75**	**0**	**0**	**K.NLGDVVLFDIVK.N (Ions score 41)**
**147 - 158**	**600.85**	**1199.68**	**1199.69**	**-2**	**0**	**K.IIGLGGVLDTSR.L (Ions score 90)**
**147 - 158**	**600.85**	**1199.69**	**1199.69**	**-1**	**0**	**K.IIGLGGVLDTSR.L (Ions score 81)**
**192 - 207**	**903.49**	**1804.97**	**1804.97**	**-2**	**0**	**R.YITVGGIPLQEFINNK.L (Ions score 51)**
**192 - 207**	**903.49**	**1804.97**	**1804.97**	**0**	**0**	**R.YITVGGIPLQEFINNK.L (Ions score 98)**
**192 - 207**	**903.50**	**1804.98**	**1804.97**	**2**	**0**	**R.YITVGGIPLQEFINNK.L (Ions score 54)**

Alphalyse A/S ■ Unsbjergvej 4 ■ 5220 Odense ■ Denmark
Tel: +45 6310 6500 ■ e-mail info@alphalyse.com
www.alphalyse.com

Sample name: 2

Protein Information

**Protein name:**	**Diphosphate-fructose-6-phosphate 1-phosphotransferase OS=Plasmodium falciparum Vietnam Oak-Knoll (FVO) GN=PFFVO_02450 PE=3 SV=1**
Entry name:	A0A024V6T3_PLAFA
Calculated MW:	160663
Calculated pI:	6.32
Mascot score:	191
Sequence coverage:	4%
Bioinformatic tools:	1: UniProt Entry 2: Conserved Domains in NCBI

Analysis Information

- Enzyme: Trypsin
- Variable modifications: Oxidation (M)
- Fixed modifications: Carbamidomethyl (C)
- Database search program: Mascot version 2.4
- Peptide Tolerance: 10 ppm
- Database: UniprotTREMBL (50011027 protein sequences)

Protein sequence

Matched peptides shown in bold underline

```
  1 MDTKSGDKNA ANKGGADGLV KTVSVLLRDN KCQFNIDENY DHNDKEKLEC
 51 EVGKRDSGMI NCLMEKLTSK KFLEEKESKN SFYLVDNENM KIKKLKEHGH
101 SASLNDDLSP LQYERTKYIP SLPKALASEY QILDENYGDE FINKNDYEDV
151 KRFLKNLHNL PMLNVKDSNN NESFKGGNIL KIGIILSGGP APGGHNVISG
201 IYDYAKRYNE QSQVIGFLGG IDGLYSKNYV TITDSMMNRF RNLGGFNMLW
251 SGRGKVKNKD DLIAIENIVA KLKLNGLVII GGDGSNSNAA LMAEYFAERQ
301 IPISIIGVPK TIDGDLKSEA IEISFGFDTA TRTYSEIIGN LCTDVKTGHN
351 VYHVVRVMGR SASHVVLECA LQTRPNVVLI GEEVEQLNLS LKDIVKNIVN
401 IILKRKSLNK NYGVILLPEG LIEFVPEMKI LISELNVILK DGPFDASKLQ
451 KSKEVWDFLP PIIRDQLLMD RESTGYIQVG KIATERLIIV LVESELAKLN
501 DKNLNIQFMS HYLGYEGRCA IPSNFDCNYC YALGYNAALL IDHKKTGYMS
551 IIRNLEDSYT NWIPAAIPFL RIMHVIKDNT GNEFPAVKRY LVDLNSPLFN
601 VLKEVRSLWS LYDLYRSPGP IQFNGHLGNA RCYTVKTPTK DNLLCQNADD
651 LELIINLTNK NMYENNGDNN HNISDDKARD GGSSPTSSAK KTKYNLSDDN
701 NNNNNNINNV STNYNNTSDG STFGNTTLLN TAYNVNGDGM NTLNCQKSNT
751 SDVLSSEPVN QGFYEQHASS YKSLGCMSEL QTSRLYNKLE LPELCSDLKA
801 KVRAGKQYIS NDPYTQKQIL SNYPHMSYEN KFQIQEIFHD KYASPISFEI
851 RIGIVFLSRQ APGAMNVLCG LYRRLKLLKG VCIAFYGLYG LLHNKYIIID
901 DDNIAKYVNQ GGLELTGNSP EHSLFDKENR NKVCETVTKL QLNGLVMPGS
```

Matched peptides (bold underline): YNE QSQVIGFLGG IDGLYSK (208–227); Y LVDLNSPLFN VLK (590–603)

Alphalyse A/S ■ Unsbjergvej 4 ■ 5220 Odense ■ Denmark
Tel: +45 6310 6500 ■ e-mail info@alphalyse.com
www.alphalyse.com

951 NITITEAALL SEYFLEKKIP TSVVGIPLTG SNNLIHELIE TCVGFDSSTK
1001 **VYASLIGNVL TDAVSMPK**YW HFIRLMGRSP SHEVLECALQ THPNVVIISE
1051 EYGAADKTLW RVVQDIADVV CARADVGKNY GTVLIPDALL MHLPHMKILL
1101 SEISDILNEA SEKGQLLEAR NDLVNLSGVD HGHLTSEWVS KLTPWSLALL
1151 KTFPQFIIKE LLQVDLRSMR FEQLETEQLL LQMVKEELQD RKQKGKYSGS
1201 FMGLTHFFGY QGRSSLPSEF DCKLAYAYGH AASIVIESGL TGYIVSIRGL
1251 CGNVKDWK**LF AIPFISLMK**I LPKGQGSKYL KSASKGDLPV IPSAPVDLNG
1301 KAYRSLKIAL QKWQMEDRFC NPGPIQFEGN ASNYYNRILF EEQSEYFEML
1351 RYVECYANIL KDTCRFGVSA DYLKNVFVQL CGMLVLAYKP NDILSNMPYI
1401 GSIEDYYDWE NQRKRMN

Peptides used for identification

Peptides shown in bold have been analysed by MS/MS sequencing

Start - End	Observed	Mr(expt)	Mr(calc)	Delta	Miss	Sequence
208 - 227	1094.55	2187.09	2187.08	1	0	**R.YNEQSQVIGFLGGIDGLYSK.N** (Ions score 51)
208 - 227	1094.55	2187.09	2187.08	2	0	**R.YNEQSQVIGFLGGIDGLYSK.N** (Ions score 56)
590 - 603	817.96	1633.91	1633.91	0	0	**R.YLVDLNSPLFNVLK.E** (Ions score 28)
590 - 603	817.96	1633.91	1633.91	2	0	**R.YLVDLNSPLFNVLK.E** (Ions score 43)
1001 - 1018	947.50	1892.98	1892.99	-4	0	**K.VYASLIGNVLTDAVSMPK.Y** + Oxidation (M) (Ions score 40)
1001 - 1018	947.50	1892.99	1892.99	-2	0	**K.VYASLIGNVLTDAVSMPK.Y** + Oxidation (M) (Ions score 47)
1001 - 1018	947.50	1892.99	1892.99	1	0	**K.VYASLIGNVLTDAVSMPK.Y** + Oxidation (M) (Ions score 58)
1259 - 1269	648.37	1294.73	1294.74	-1	0	**K.LFAIPFISLMK.I** + Oxidation (M) (Ions score 14)
1259 - 1269	648.38	1294.74	1294.74	0	0	**K.LFAIPFISLMK.I** + Oxidation (M) (Ions score 32)
1259 - 1269	648.38	1294.74	1294.74	0	0	**K.LFAIPFISLMK.I** + Oxidation (M) (Ions score 35)

Alphalyse A/S ■ Unsbjergvej 4 ■ 5220 Odense ■ Denmark
Tel: +45 6310 6500 ■ e-mail info@alphalyse.com
www.alphalyse.com

LC-MSMS Protein Identification Report

Order 16372_Plasmodium falciparum

Sample name: 2

Protein Information

Protein name:	**Chaperone DnaK OS=Plasmodium falciparum Vietnam Oak-Knoll (FVO) GN=PFFVO_02477 PE=3 SV=1**
Entry name:	A0A024V6C5_PLAFA
Calculated MW:	72457
Calculated pI:	5.18
Mascot score:	144
Sequence coverage:	5%
Bioinformatic tools:	1: UniProt Entry 2: Conserved Domains in NCBI

Analysis Information

- Enzyme: Trypsin
- Variable modifications: Oxidation (M)
- Fixed modifications: Carbamidomethyl (C)
- Database search program: Mascot version 2.4
- Peptide Tolerance: 10 ppm
- Database: UniprotTREMBL (50011027 protein sequences)

Protein sequence

Matched peptides shown in bold underline

1 MKQIRPYILL LIVSLLKFIS AVDSNIEGPV IGIDLGTTYS CVGVFKNGRV
51 EILNNELGNR ITPSYVSFVD GERKVGEAAK LEATLHPTQT VFDVKRLIGR
101 KFDDQEVVKD RSLLPYEIVN NQGKPNIKVQ IKDKDTTFAP EQISAMVLEK
151 MKEIAQSFLG KPVKNAVVTV PAYFNDAQRQ ATK**DAGTIAG LNIVR**IINEP
201 TAAALAYGLD KKEETSILVY DLGGGTFDVS ILVIDNGVFE VYATAGNTHL
251 GGEDFDQRVM DYFIKMFKKK NNIDLRTDKR AIQKLRKEVE IAKRNLSVVH
301 STQIEIEDIV EGHNFSETLT RAKFEELNDD LFRETLEPVK KVLDDAKYEK
351 SKIDEIVLVG GSTRIPKIQQ IIKEFFNGKE PNRGINPDEA VAYGAAIQAG
401 IILGEELQDV VLLDVTPLTL GIETVGGIMT QLIKRNTVIP TKK**SQTFSTY**
451 **QDNQPAVLIQ VFEGER**ALTK DNHLLGKFEL SGIPPAQRGV PKIEVTFTVD
501 KNGILHVEAE DKGTGKSRGI TITNDKGRLS KEQIEKMIND AEKFADEDKN
551 LREKVEAKNN LDNYIQSMKA TVEDKDKLAD KIEKEDKNTI LSAVKDAEDW
601 LNNNSNADSE ALKQKLKDLE AVCQPIIVKL YGQPGGPSPQ PSGDEDVDSD
651 EL

Peptides used for identification

Peptides shown in bold have been analysed by MS/MS sequencing

Start - End Observed Mr(expt) Mr(calc) Delta Miss Sequence

Alphalyse A/S ■ Unsbjergvej 4 ■ 5220 Odense ■ Denmark
Tel: +45 6310 6500 ■ e-mail info@alphalyse.com
www.alphalyse.com

184 - 195	600.34	1198.66	1198.67	-3	0	K.DAGTIAGLNIVR.I (Ions score 56)
184 - 195	600.34	1198.67	1198.67	-1	0	K.DAGTIAGLNIVR.I (Ions score 53)
444 - 466	886.43	2656.27	2656.28	-3	0	K.SQTFSTYQDNQPAVLIQVFEGER.A (Ions score 68)
444 - 466	886.43	2656.28	2656.28	1	0	K.SQTFSTYQDNQPAVLIQVFEGER.A (Ions score 58)
444 - 466	886.43	2656.28	2656.28	1	0	K.SQTFSTYQDNQPAVLIQVFEGER.A (Ions score 86)
444 - 466	886.43	2656.28	2656.28	2	0	K.SQTFSTYQDNQPAVLIQVFEGER.A (Ions score 87)
444 - 466	886.44	2656.29	2656.28	4	0	K.SQTFSTYQDNQPAVLIQVFEGER.A (Ions score 64)

Alphalyse A/S ■ Unsbjergvej 4 ■ 5220 Odense ■ Denmark
Tel: +45 6310 6500 ■ e-mail info@alphalyse.com
www.alphalyse.com

Sample name: 2

Protein Information

**Protein name:**	**Uncharacterized protein OS=Plasmodium falciparum Vietnam Oak-Knoll (FVO) GN=PFFVO_01035 PE=4 SV=1**
Entry name:	A0A024VCE7_PLAFA
Calculated MW:	27525
Calculated pI:	6.07
Mascot score:	130
Sequence coverage:	17%
Bioinformatic tools:	1: UniProt Entry 2: Conserved Domains in NCBI

Analysis Information

- Enzyme: Trypsin
- Variable modifications: Oxidation (M)
- Fixed modifications: Carbamidomethyl (C)
- Database search program: Mascot version 2.4
- Peptide Tolerance: 10 ppm
- Database: UniprotTREMBL (50011027 protein sequences)

Protein sequence

Matched peptides shown in bold underline

```
  1 MDNLLRHLKI SKEQITPVVL VVGDPGRVDK IKVVCDSYVD LAYNREYKSV
 51 ECHYKGQKFL CVSHGVGSAG CAVCFEELCQ NGAKVIIRAG SCGSLQPDLI
101 KRGDICICNA AVREDRVSHL LIHGDFPAVG DFDVYDTLNK CAQELNVPVF
151 NGISVSSDMY YPNKIIPSRL EDYSKANAAV VEMELATLMV IGTLRKVKTG
201 GILIVDGCPF KWDEGDFDNN LVPHQLENMI KIALGACAKL ATKYA
```

Peptides used for identification

Peptides shown in bold have been analysed by MS/MS sequencing

Start - End	Observed	Mr(expt)	Mr(calc)	Delta	Miss	Sequence
13 - 30	641.03	1920.06	1920.07	-2	1	**K.EQITPVVLVVGDPGRVDK.I** (Ions score 57)
141 - 164	916.76	2747.26	2747.26	0	0	**K.CAQELNVPVFNGISVSSDMYYPNK.I** + Oxidation (M) (Ions score 73)
141 - 164	916.76	2747.26	2747.26	1	0	**K.CAQELNVPVFNGISVSSDMYYPNK.I** + Oxidation (M) (Ions score 71)
141 - 164	916.76	2747.26	2747.26	1	0	**K.CAQELNVPVFNGISVSSDMYYPNK.I** + Oxidation (M) (Ions score 60)

Alphalyse A/S ■ Unsbjergvej 4 ■ 5220 Odense ■ Denmark
Tel: +45 6310 6500 ■ e-mail info@alphalyse.com
www.alphalyse.com

Sample name: 2

Protein Information

**Protein name:**	**Eukaryotic initiation factor 4A OS=Plasmodium falciparum Vietnam Oak-Knoll (FVO) GN=PFFVO_05337 PE=3 SV=1**
Entry name:	A0A024UY98_PLAFA
Calculated MW:	46306
Calculated pI:	5.67
Mascot score:	129
Sequence coverage:	7%
Bioinformatic tools:	1: UniProt Entry 2: Conserved Domains in NCBI

Analysis Information

- Enzyme: Trypsin
- Variable modifications: Oxidation (M)
- Fixed modifications: Carbamidomethyl (C)
- Database search program: Mascot version 2.4
- Peptide Tolerance: 10 ppm
- Database: UniprotTREMBL (50011027 protein sequences)

Protein sequence

Matched peptides shown in bold underline

1 MSTKEETFNN ENDIEGNTEE IVDTFDALGL NEKLLRGIYS YGFEKPSAIQ
51 QR**GIKPILNG YDTIGQAQSG TGK**TATFVIS SLQLINYDYV ACQALILAPT
101 RELAQQIQKV VLALGDYLKV KCHACVGGTV VREDIDKLKQ GVHMVVGTPG
151 RVYDMIDKRH LGVDRLKLFI LDEADEMLSR GFKAQIYEVF KKLVPDIQVA
201 LFSATMPQEI LELTTRFMRD PKTILVKKDE LTLEGIRQFY VAVEKEEWKL
251 DTLCDLYETL TITQSIIYCN TRKKVDILTQ EMHNRLFTVS CMHGDMDQKD
301 RDLIMREFRS GSTR**VLVTTD LLAR**GIDVQQ VSLVINYDLP ASPDTYIHRI
351 GRSGRFGRKG VAINFVTNDD KEKDKLKKIE SYYSTQIEEM PLEQNKVDHV
401 LYF

Peptides used for identification

Peptides shown in bold have been analysed by MS/MS sequencing

Start - End	Observed	Mr(expt)	Mr(calc)	Delta	Miss	Sequence
53 - 73	706.71	2117.11	2117.11	-1	0	**R.GIKPILNGYDTIGQAQSGTGK.T** (Ions score 68)
53 - 73	706.71	2117.11	2117.11	0	0	**R.GIKPILNGYDTIGQAQSGTGK.T** (Ions score 70)
315 - 324	550.84	1099.66	1099.66	-1	0	**R.VLVTTDLLAR.G** (Ions score 59)

Alphalyse A/S ■ Unsbjergvej 4 ■ 5220 Odense ■ Denmark
Tel: +45 6310 6500 ■ e-mail info@alphalyse.com
www.alphalyse.com

Sample name: 2

Protein Information

**Protein name:**	**Uncharacterized protein OS=Plasmodium falciparum Vietnam Oak-Knoll (FVO) GN=PFFVO_04306 PE=4 SV=1**
Entry name:	A0A024V2J1_PLAFA
Calculated MW:	31297
Calculated pI:	5.43
Mascot score:	105
Sequence coverage:	8%
Bioinformatic tools:	1: UniProt Entry 2: Conserved Domains in NCBI

Analysis Information

- Enzyme: Trypsin
- Variable modifications: Oxidation (M)
- Fixed modifications: Carbamidomethyl (C)
- Database search program: Mascot version 2.4
- Peptide Tolerance: 10 ppm
- Database: UniprotTREMBL (50011027 protein sequences)

Protein sequence

Matched peptides shown in bold underline

```
  1 MTLIENLNSD KTFLENNQYT DEGVKVYEFI FGENYISSGG LEATKKILSD
 51 IELNENSKVL DIGSGLGGGC MYINEKYGAH THGIDICSNI VNMANERVSG
101 NNKIIFEAND ILTKEFPENN FDLIYSRDAI LHLSLENKNK LFQKCYKWLK
151 PTGTLLITDY CATEKENWDD EFKEYVKQRK YTLITVEEYA DTLTACNFKN
201 VVSKDLSDYW NQLLEVEHKY LHENKEEFLK LFSEKKFISL DDGWSRKIKD
251 SKRKMQRWGY FKATKN
```

Peptides used for identification

Peptides shown in bold have been analysed by MS/MS sequencing

Start - End	Observed	Mr(expt)	Mr(calc)	Delta	Miss	Sequence
**47 - 58**	**687.86**	**1373.70**	**1373.70**	**-2**	**0**	**K.ILSDIELNENSK.V (Ions score 62)**
**104 - 114**	**638.86**	**1275.71**	**1275.71**	**1**	**0**	**K.IIFEANDILTK.E (Ions score 42)**

Alphalyse A/S ■ Unsbjergvej 4 ■ 5220 Odense ■ Denmark
Tel: +45 6310 6500 ■ e-mail info@alphalyse.com
www.alphalyse.com

Sample name: 2

Protein Information

**Protein name:**	**Uncharacterized protein OS=Plasmodium falciparum Vietnam Oak-Knoll (FVO) GN=PFFVO_00205 PE=3 SV=1**
Entry name:	A0A024VCX1_PLAFA
Calculated MW:	121726
Calculated pI:	5.93
Mascot score:	99
Sequence coverage:	2%
Bioinformatic tools:	1: UniProt Entry 2: Conserved Domains in NCBI

Analysis Information

- Enzyme: Trypsin
- Variable modifications: Oxidation (M)
- Fixed modifications: Carbamidomethyl (C)
- Database search program: Mascot version 2.4
- Peptide Tolerance: 10 ppm
- Database: UniprotTREMBL (50011027 protein sequences)

Protein sequence

Matched peptides shown in bold underline

1 MIFFNFKLNR MICPIFFLYI INVLFTQYFI KCEGNKVTVI SHNNGHNDNL
51 DVNKNGVISQ ENVFDTSESL NLPSNKKVGS DDLNTTTISF TVPDNLENEV
101 KVVSSSESGK GATVSHTKVT SEGLSDTQPN VTQSVSSSTH TPGSLDSTMS
151 TEQHSSVSQS SLPTESSSET LNK**ATVPEIP** **IQINSGLLK**N YNGVKVTGSC
201 GSYFRVYLVP HILIYALTK**Y** **SVIQLESLFN** **DNAR**IDVEHK GELQNKCSEG
251 YHFKLVVYIT HNVLNLKWKT YKPNEESKSE DSDVRKYRIP KLERPFTSIQ
301 VYTANSKAGV IETKNYNIRT DIPDTCDAIA TDCFLNGNVN IEKCFQCTLL
351 VQKKDKSHEC FKYVSSEMKK KMNEIKVKAQ DDFNPNEYKL IESIDNILSK
401 IYKKANKPFE ISKDLINLED LDYQFKNELL EYCKLLKKVD TSGTLEEYEL
451 GNAEDIYNNL TRLLKSHSDE NIVTLQGKLR NTAICIKNVD EWILNKRGLT
501 LPSESPSESS SKSDSYLNTF NDKDKNEDKD DMSKNSKEEF KNDDKENSDD
551 QNNNDSNKKD DENNINNGDT NYVYDFDDDD YDNNSYEKDM YESPIKENKN
601 GVIDLEKYGN QIKLKSPYFK NSKYCNYEYC NRWRDKTSCI SQIEVEEQGN
651 CGLCWIFASK LHFETIRCMR GYGHFRSSAL YVANCSKRKP IDRCEEGSNP
701 LEFLRILDEK KFLPLESNYP YSYTSAGNSC PKLPNSWTNL WGDTKLLFNK
751 KVHRYIGNKG FISHETSYFK NNMDLFIDMV KREVQNKGSV IIYIKTQDVI
801 GYDFNGKGVH SMCGDRTPDH AANIIGYGNY INKKGEKRSY WLIRNSWSYY
851 WGDEGNFRVD MLGPKNCLYN FIHTVVFFKL DLGTIHVPKK KSWKKNVYFL
901 RHNPDFMYSL YYNNYEPETS QDFESENDYD NAFVHGQSDE SDETNKEGKN

Alphalyse A/S ■ Unsbjergvej 4 ■ 5220 Odense ■ Denmark
Tel: +45 6310 6500 ■ e-mail info@alphalyse.com
www.alphalyse.com

```
 951 VHNSVEKKIQ ILHILKHIKD SQIKRGLVKY DNINETKDEH TCSRVNSQDA
1001 EKYEECKKFC LTKWNECKDH YSPGYCLTDL YKGEDCNFCY V
```

Peptides used for identification

Peptides shown in bold have been analysed by MS/MS sequencing

Start - End	Observed	Mr(expt)	Mr(calc)	Delta	Miss	Sequence
174 - 189	847.00	1691.98	1691.98	-2	0	K.**ATVPEIPIQINSGLLK**.N (Ions score 52)
174 - 189	847.00	1691.98	1691.98	-2	0	K.**ATVPEIPIQINSGLLK**.N (Ions score 44)
174 - 189	847.00	1691.98	1691.98	-1	0	K.**ATVPEIPIQINSGLLK**.N (Ions score 20)
220 - 234	884.95	1767.88	1767.88	2	0	K.**YSVIQLESLFNDNAR**.I (Ions score 47)

Alphalyse A/S ■ Unsbjergvej 4 ■ 5220 Odense ■ Denmark
Tel: +45 6310 6500 ■ e-mail info@alphalyse.com
www.alphalyse.com

Sample name: 2

Protein Information

Protein name:	**Uncharacterized protein (Fragment) OS=Plasmodium falciparum Vietnam Oak-Knoll (FVO) GN=PFFVO_06138 PE=4 SV=1**
Entry name:	A0A024UXG4_PLAFA
Calculated MW:	293738
Calculated pI:	5.11
Mascot score:	96
Sequence coverage:	1%
Bioinformatic tools:	1: UniProt Entry 2: Conserved Domains in NCBI

Analysis Information

- Enzyme: Trypsin
- Variable modifications: Oxidation (M)
- Fixed modifications: Carbamidomethyl (C)
- Database search program: Mascot version 2.4
- Peptide Tolerance: 10 ppm
- Database: UniprotTREMBL (50011027 protein sequences)

Protein sequence

Matched peptides shown in bold underline

```
1   KRGGDPGVST AVSSIMQHEN FKRMLMFGLR SLSDFCNPTS KAYKENASDA
51  LDRGVVVSIK NAVINYKDDD DILFCSSRVL LSMSDYCMSE KDTNALKKLI
101 TDGGVDGIVE IVKSFPSDPD TLKNCMAFIK NMNDSNYQIE GREVGIALLN
151 VFTSKTYTNK LTNGIVLALC IISKSTSGSK GLNDEGAHHK LLDYCLNINS
201 LNDDTAEIVE SVFDIIKNMS SNGYVDPTII EKSVIILDKF KSYPRVISKG
251 SDAMKCAVGP EELSKCLNVL KKSAQGSKEQ DAALELLSSL SYISSITDKV
301 VESGGIPVLI ELINSGLQQY ESNPEKISRL VAGASRMLGR ISNNPPHAAI
351 VVEYGGIATL CTAISYFPND VECSKAICNA LTPFVSRSNY VSEINNYSLF
401 ASLLPILYAS LESVELAKAS MECIASASMI NEFHEQMVNN QAIEILSTCV
451 QYHLTEMDYL LNCFTAYFRL SDYITTVEPI NQYGGVDGIA NALLAVSSNS
501 KIVEIGLKLI NKMLTTSDSV NYLSNKQIVD SVLTVMLENE NKEVIIQEGT
551 KIMEKLATES DCQRHITNLE TIFNSSETNQ EEAYKTLAAI SGLSRIESLK
601 NILESKGADT SIFNGIKIWI ESARFIEQTK LIKAGLKTIK TLKLNASATL
651 HDVLGSIVDL MCLSQVKRIA ESDEPDENIL ITSAECINYL TEVNKINSAE
701 IVEACLENIF KLMKKYSESR LTQINLISAM NNILLSSNKI GVDILINKGY
751 IKHIITYLQK VPMYVDVQII GFTVLANLVK ISPDSVEGIK KLNALIPLQT
801 ALRTHAKNMK LKTTCAPLLS VLMPLDSLTN EIQDIIKLCN KSMNDKNLSK
851 LHEYLVALNE LLLTPEACKI ASRSNIGEFF NDLTAWLKKN PTVYDSSSKD
901 DYEISGRSLF DAVISEIAHA STNISQTRLG LVHLTKCNMV SSLVQLHDIL
```

Matched peptides (bold underline): LI (99–100), TDGGVDGIVE IVK (101–113), EVGIALLN (143–150), VFTSK (151–155)

```
 951 KLPGDNYTEE AVSNILEALS LLLKYDITNA EIGFNSGLIK KLCAGINYFS
1001 HSDSVINKTF GCLACMCTSK NRVGQLISCP EYEGLIKLIV ELIGDSEKNK
1051 LSRGSAIKAV YELLKTEDED IIKDISCKTS IVDNLYKIMG EYQADLSIVQ
1101 DCSRCLAIIV DYVNIEEIMK VDKYTPMKVL LECLNKSKND ELTVLEMLTV
1151 LVKLCNSDDK MKLKELGAID VISDITMIHS ENEEISRLGG VMYSYMGADE
1201 QVKKLMKLIL NVKKEDSDAV QKIDNFTSKL EMFLRAPLEN PSDALQYTEV
1251 TLQVLNSYLA SEVDNSSLQT NIALVTKRLV DRVKHDSEDP LGSWAVASAG
1301 TLNQYIDMIS NKIGLSNFKF VSPVYGVLAA CVMNPYTKQL VMDKLPLLID
1351 NTYEILEQNK NRPSVVQGVF EFLEQVVNDE EGSKLLYKKY NGSMGNLIDQ
1401 TITVMNLNRA NDSVYLPGIK LLSAICQTAS VSGYGNDMNT SNIIESCELL
1451 LNLGSEKDRT VEFLNLIDTM ILGNLLDEKV ASEALKKINS LVAEENLSKY
1501 TEENRVEIIK SYAGLVKDCA ATGLFAHIRG IEKVEILNNL GKCMEYEQND
1551 EVTIAVLDAL SQISESDPSI ATKILTSSLP IIMENNRDNI LNDAATAEAF
1601 LQALQNLVLH EGVGRQLINN TELQNLLKDL EQALDERKEE LGNDYVEDMK
1651 LKISNICNAI NGDKPKEKKC KDVYDVLTDY KKSNLIIDVL QEPSLEEDMD
1701 FVLERLKVYN QDNLLPTTAT GTDNSYGHMA IEMFCENEVN INELIKRNFH
1751 VSAFHALSKQ SNENVIHYSC RSICAFTKNP KGLEAVKDIK DFANIISKSV
1801 GDLSKENTLD KEIKEDFLIH RVLLIDRTAH NRNVYDKTNA VHYLIDIWNQ
1851 YDNGDYSVLL LRHVFRSMRK IVSDAHVQTL LKAGVLVRLI NIINDIETDK
1901 IIFPDVLFLI GSLSVVKEIK IQIGELKGID ACVNLLLRSI NVEKMEPTIT
1951 NCCLALANMC IDHKGNSSIF CNLKGPDVNV KILKLYRSNF DVTNGASVLL
2001 CNILFRNEEM KKQYGINGAP AELVECLKSY DGSDDKNAVR CIESLFKAIS
2051 NLSLYTVNVK YFLEAQIQVS YESWLRNLNE SFPDAQLETG LRTLSNLVME
2101 NDEENMKNFG VTLIPVLNVL KQNREDTKVI FLLLDILCSL CRLNANAKIF
2151 AENNGIETTI NVIQLYDYDI NLLSLAIHLL SNQCKIESSL PLLVNADAFS
2201 ILISCMEAET DEFEMTELVV SSLRCVRRLI QSEELAYEFC NCGGVPSMAN
2251 IICKSIKKSI VMLEVLRVLL CVLYYTQNVE GVTNEYPEEE EELYNARLGG
2301 WYNISMDKEM IDSIIQAVLT CAYDVNHQKQ LRLQKVSLGL LAYFAYHRLG
2351 IISMTASGFD SLTRELLNNF GGDAVIMQLL AICIDNIAMY SVEVYDTTIT
2401 RDIIKCFKSA LSKMNNKKED KQLWQKVELT LEAMNSADDP LEAFKNTLLI
2451 FDFNLSEFDK DPYVNGVHDL ASNIKDCLRK GGHSKIYYQS DQRLLFKWKA
2501 SQDLNTLEWT IGDDTERVFK ISVVRIKNIS KGLSHPILIS ANKREPRKVS
2551 AKVTLCIYGP PTEDFPEGLE LPIKTKTQKE RDAFVDLIVL WRDAASYNY
```

Peptides used for identification

Peptides shown in bold have been analysed by MS/MS sequencing

Start - End	Observed	Mr(expt)	Mr(calc)	Delta	Miss	Sequence
99 - 113	764.43	1526.85	1526.86	-1	0	K.**LITDGGVDGIVEIVK**.S (Ions score 58)
143 - 155	695.90	1389.79	1389.79	0	0	R.**EVGIALLNVFTSK**.T (Ions score 38)
143 - 155	695.90	1389.79	1389.79	3	0	R.**EVGIALLNVFTSK**.T (Ions score 18)

Sample name: 2

Protein Information

Protein name:	Uncharacterized protein OS=Plasmodium falciparum Vietnam Oak-Knoll (FVO) GN=PFFVO_00066 PE=4 SV=1
Entry name:	A0A024VEQ1_PLAFA
Calculated MW:	18479
Calculated pI:	5.1
Mascot score:	50
Sequence coverage:	8%
Bioinformatic tools:	1: UniProt Entry 2: Conserved Domains in NCBI

Analysis Information

- Enzyme: Trypsin
- Variable modifications: Oxidation (M)
- Fixed modifications: Carbamidomethyl (C)
- Database search program: Mascot version 2.4
- Peptide Tolerance: 10 ppm
- Database: UniprotTREMBL (50011027 protein sequences)

Protein sequence

Matched peptides shown in bold underline

```
  1 MSGSNCVAIA CDLRLGANTF TTVSTKFSKI FKMNNNVYVG LSGLATDIQT
 51 LYEILRYRVN LYEVRQDAEM DVECFANMLS SILYSNRFSP YFVNPIVVGF
101 KLKHYVDEEG EKKVNYEPYL TAYDLIGAKC ETRDFVVNGV TSEQLFGMCE
151 SLYVKDQVKE
```

Peptides used for identification

Peptides shown in bold have been analysed by MS/MS sequencing

Start - End	Observed	Mr(expt)	Mr(calc)	Delta	Miss	Sequence
88 - 101	807.44	1612.87	1612.87	1	0	**R.FSPYFVNPIVVGFK.L (Ions score 50)**

Alphalyse A/S ■ Unsbjergvej 4 ■ 5220 Odense ■ Denmark
Tel: +45 6310 6500 ■ e-mail info@alphalyse.com
www.alphalyse.com

Sample name: 4

Protein Information

**Protein name:**	**Merozoite surface protein 1 OS=Plasmodium falciparum Vietnam Oak-Knoll (FVO) GN=PFFVO_02613 PE=4 SV=1**
Entry name:	A0A024V850_PLAFA
Calculated MW:	189101
Calculated pI:	5.98
Mascot score:	664
Sequence coverage:	8%
Bioinformatic tools:	1: UniProt Entry 2: Conserved Domains in NCBI

Analysis Information

- Enzyme: Trypsin
- Variable modifications: Oxidation (M)
- Fixed modifications: Carbamidomethyl (C)
- Database search program: Mascot version 2.4
- Peptide Tolerance: 10 ppm
- Database: UniprotTREMBL (50011027 protein sequences)

Protein sequence

Matched peptides shown in bold underline

1 MKIIFFLCSF LFFIINTQCV THESYQELVK KLEALEDAVL TGYSLFQKEK
51 **MVLNEGTSGT AVTTSTPGSK** GSVASGGSGG SVASGGSVAS GGSVASGGSV
101 ASGGSVASGG SGNSRRTNPS DNSSDSDAKS YADLKHRVRN YLLTIKELKY
151 PQLFDLTNHM LTLCDNIHGF KYLIDGYEEI NELLYKLNFY FDLLRAKLND
201 VCANDYCQIP FNLKIRANEL DVLKKLVFGY RKPLDNIKDN VGKMEDYIKK
251 NKK**TIENINE LIEESKK**TID KNKNATKEEE KKK**LYQAQYD LSIYNK**QLEE
301 AHNLISVLEK RIDTLKKNEN IKELLDKINE IKNPPPANSG NTPNTLLDKN
351 KKIEEHEKEI KEIAKTIKFN IDSLFTDPLE LEYYLREKNK NIDISAKVET
401 KESTEPNEYP NGVTYPLSYN DINNALNELN SFGDLINPFD YTKEPSKNIY
451 TDNERKKFIN EIKEKIKIEK KKIESDKKSY EDRSKSLNDI TKEYEK**LLNE**
501 **IYDSKFNNNI DLTNFEK**MMG KRYSYKVEKL THHNTFASYE NSKHNLEKLT
551 KALKYMEDYS LRNIVVEKEL KYYKNLISKI ENEIETLVEN IKKDEEQLFE
601 KKITKDENKP DEKILEVSDI VKVQVQKVLL MNKIDELKKT QLILKNVELK
651 HNIHVPNSYK QENKQEPYYL IVLKKEIDKL KVFMPKVESL INEEKKNIKT
701 EGQSDNSEPS TEGEITGQAT TKPGQQAGSA LEGDSVQAQA QEQK**QAQPPV**
751 **PVPVPEAK**AQ VPTPPAPVNN KTENVSKLDY LEKLYEFLNT SYICHKYILV
801 SHSTMNEKIL KQYKITKEEE SKLSSCDPLD LLFNIQNNIP VMYSMFDSLN
851 NSLSQLFMEI YEKEMVCNLY KLKDNDKIKN LLEEAKKVST SVKTLSSSSM
901 QPLSLTPQDK PEVSANDDTS HSTNLNNSLK **LFENILSLGK NKNIYQELIG**

Alphalyse A/S ■ Unsbjergvej 4 ■ 5220 Odense ■ Denmark
Tel: +45 6310 6500 ■ e-mail info@alphalyse.com
www.alphalyse.com

951 **QK**SSENFYEK ILKDSDTFYN ESFTNFVKSK ADDINSLNDE SKRKKLEEDI
1001 NKLKKTLQLS FDLYNKYKLK LERLFDKKKT VGKYKMQIKK LTLLKEQLES
1051 KLNSLNNPKH VLQNFSVFFN KKK**EAEIAET ENTLENTK**IL LKHYKGLVKY
1101 YNGESSPLKT LSEESIQTED NYASLENFKV LSKLEGKLKD NLNLEKKKLS
1151 YLSSGLHHLI AELKEVIKNK NYTGNSPSEN NTDVNNALES YKKFLPEGTD
1201 VATVVSESGS DTLEQSQPKK PASTHVGAES NTITTSQNVD DEVDDVIIVP
1251 IFGESEEDYD DLGQVVTGEA VTPSVIDNIL SKIENEYEVL YLKPLAGVYR
1301 SLKK**QLENNV MTFNVNVK**DI LNSRFNKREN FKNVLESDLI PYKDLTSSNY
1351 VVKDPYKFLN KEKRDKFLSS YNYIKDSIDT DINFANDVLG YYKILSEKYK
1401 SDLDSIKKYI NDKQGENEKY LPFLNNIETL YKTVNDKIDL FVIHLEAKVL
1451 NYTYEKSNVE VKIKELNYLK TIQDKLADFK KNNNFVGIAD LSTDYNHNNL
1501 LTK**FLSTGMV FENLAK**TVLS NLLDGNLQGM LNISQHQCVK KQCPQNSGCF
1551 RHLDEREECK CLLNYKQEGD KCVENPNPTC NENNGGCDAD AKCTEEDSGS
1601 NGKKITCECT KPDSYPLFDG IFCSSSNFLG ISFLLILMLI LYSFI

Peptides used for identification

Peptides shown in bold have been analysed by MS/MS sequencing

Start - End	Observed	Mr(expt)	Mr(calc)	Delta	Miss	Sequence
51 - 70	977.48	1952.94	1952.94	0	0	K.MVLNEGTSGTAVTTSTPGSK.G + Oxidation (M) (Ions score 77)
51 - 70	977.48	1952.94	1952.94	0	0	K.MVLNEGTSGTAVTTSTPGSK.G + Oxidation (M) (Ions score 61)
51 - 70	977.48	1952.94	1952.94	2	0	K.MVLNEGTSGTAVTTSTPGSK.G + Oxidation (M) (Ions score 69)
254 - 267	553.96	1658.87	1658.87	-3	1	K.TIENINELIEESKK.T (Ions score 71)
254 - 267	553.96	1658.87	1658.87	-1	1	K.TIENINELIEESKK.T (Ions score 62)
254 - 267	553.96	1658.87	1658.87	-1	1	K.TIENINELIEESKK.T (Ions score 62)
254 - 267	553.96	1658.87	1658.87	-1	1	K.TIENINELIEESKK.T (Ions score 59)
254 - 267	830.44	1658.87	1658.87	0	1	K.TIENINELIEESKK.T (Ions score 37)
254 - 267	553.97	1658.87	1658.87	1	1	K.TIENINELIEESKK.T (Ions score 45)
254 - 267	553.97	1658.88	1658.87	2	1	K.TIENINELIEESKK.T (Ions score 29)
284 - 296	809.91	1617.80	1617.80	-1	0	K.LYQAQYDLSIYNK.Q (Ions score 51)
284 - 296	809.91	1617.80	1617.80	0	0	K.LYQAQYDLSIYNK.Q (Ions score 26)
284 - 296	809.91	1617.80	1617.80	0	0	K.LYQAQYDLSIYNK.Q (Ions score 69)
284 - 296	809.91	1617.80	1617.80	0	0	K.LYQAQYDLSIYNK.Q (Ions score 66)
284 - 296	809.91	1617.81	1617.80	2	0	K.LYQAQYDLSIYNK.Q (Ions score 60)
497 - 505	547.79	1093.56	1093.57	-3	0	K.LLNEIYDSK.F (Ions score 14)
497 - 505	547.79	1093.56	1093.57	-1	0	K.LLNEIYDSK.F (Ions score 24)
497 - 505	547.79	1093.56	1093.57	-1	0	K.LLNEIYDSK.F (Ions score 30)
497 - 505	547.79	1093.57	1093.57	2	0	K.LLNEIYDSK.F (Ions score 19)
506 - 517	734.86	1467.70	1467.70	-2	0	K.FNNNIDLTNFEK.M (Ions score 18)
506 - 517	734.86	1467.70	1467.70	-2	0	K.FNNNIDLTNFEK.M (Ions score 54)
506 - 517	734.86	1467.70	1467.70	0	0	K.FNNNIDLTNFEK.M (Ions score 72)
506 - 517	734.86	1467.70	1467.70	1	0	K.FNNNIDLTNFEK.M (Ions score 78)
745 - 758	728.91	1455.81	1455.81	-2	0	K.QAQPPVPVPVPEAK.A (Ions score 54)
745 - 758	728.91	1455.81	1455.81	-1	0	K.QAQPPVPVPVPEAK.A (Ions score 39)
745 - 758	728.91	1455.81	1455.81	-1	0	K.QAQPPVPVPVPEAK.A (Ions score 46)
745 - 758	728.91	1455.81	1455.81	0	0	K.QAQPPVPVPVPEAK.A (Ions score 49)
745 - 758	728.91	1455.81	1455.81	0	0	K.QAQPPVPVPVPEAK.A (Ions score 48)
745 - 758	728.91	1455.81	1455.81	1	0	K.QAQPPVPVPVPEAK.A (Ions score 45)
745 - 758	728.91	1455.81	1455.81	1	0	K.QAQPPVPVPVPEAK.A (Ions score 36)
745 - 758	728.91	1455.81	1455.81	4	0	K.QAQPPVPVPVPEAK.A (Ions score 52)
931 - 940	567.33	1132.65	1132.65	-2	0	K.LFENILSLGK.N (Ions score 18)
931 - 940	567.33	1132.65	1132.65	1	0	K.LFENILSLGK.N (Ions score 28)
931 - 940	567.33	1132.65	1132.65	1	0	K.LFENILSLGK.N (Ions score 15)
941 - 952	724.40	1446.78	1446.78	-1	1	K.NKNIYQELIGQK.S (Ions score 7)

Alphalyse A/S ■ Unsbjergvej 4 ■ 5220 Odense ■ Denmark
Tel: +45 6310 6500 ■ e-mail info@alphalyse.com
www.alphalyse.com

941 - 952	724.40	1446.78	1446.78	1	1	K.NKNIYQELIGQK.S (Ions score 29)
943 - 952	603.33	1204.64	1204.65	-3	0	K.NIYQELIGQK.S (Ions score 21)
1074 - 1088	846.40	1690.78	1690.79	-3	0	K.EAEIAETENTLENTK.I (Ions score 42)
1074 - 1088	846.40	1690.79	1690.79	-1	0	K.EAEIAETENTLENTK.I (Ions score 66)
1074 - 1088	846.40	1690.79	1690.79	-1	0	K.EAEIAETENTLENTK.I (Ions score 96)
1074 - 1088	846.40	1690.79	1690.79	3	0	K.EAEIAETENTLENTK.I (Ions score 84)
1305 - 1318	833.42	1664.82	1664.82	-1	0	K.QLENNVMTFNVNVK.D + Oxidation (M) (Ions score 31)
1305 - 1318	833.42	1664.82	1664.82	0	0	K.QLENNVMTFNVNVK.D + Oxidation (M) (Ions score 35)
1305 - 1318	833.42	1664.82	1664.82	2	0	K.QLENNVMTFNVNVK.D + Oxidation (M) (Ions score 87)
1305 - 1318	833.42	1664.82	1664.82	2	0	K.QLENNVMTFNVNVK.D + Oxidation (M) (Ions score 32)
1305 - 1318	833.42	1664.82	1664.82	2	0	K.QLENNVMTFNVNVK.D + Oxidation (M) (Ions score 44)
1504 - 1516	736.87	1471.73	1471.74	-4	0	K.FLSTGMVFENLAK.T + Oxidation (M) (Ions score 14)
1504 - 1516	736.88	1471.74	1471.74	2	0	K.FLSTGMVFENLAK.T + Oxidation (M) (Ions score 22)
1504 - 1516	736.88	1471.74	1471.74	3	0	K.FLSTGMVFENLAK.T + Oxidation (M) (Ions score 26)

Alphalyse A/S ■ Unsbjergvej 4 ■ 5220 Odense ■ Denmark
Tel: +45 6310 6500 ■ e-mail info@alphalyse.com
www.alphalyse.com

LC-MSMS Protein Identification Report

Order 16372_Plasmodium falciparum

Sample name: 4

Protein Information

**Protein name:**	**Glutamate-rich protein OS=Plasmodium falciparum (isolate 3D7) GN=PF10_0344 PE=4 SV=1**
Entry name:	Q8IJ56_PLAF7
Calculated MW:	141024
Calculated pI:	4.28
Mascot score:	223
Sequence coverage:	6%
Bioinformatic tools:	1: UniProt Entry 2: Conserved Domains in NCBI

Analysis Information

- Enzyme: Trypsin
- Variable modifications: Oxidation (M)
- Fixed modifications: Carbamidomethyl (C)
- Database search program: Mascot version 2.4
- Peptide Tolerance: 10 ppm
- Database: UniprotTREMBL (50011027 protein sequences)

Protein sequence

Matched peptides shown in bold underline

1 MRNLFHITIC LVTLNLFILE INAKTNTSEN RNKRIGGPKL RGNVTSNIKF
51 PSDNKGKIIR GSNDKLNKNS EDVLEQSEKS LVSENVPSGL DIDDIPKESI
101 FIQEDQEGQT HSELNPETSE HSKDLNNNDS KNESSDIISV NNKSNKVQNH
151 FESLSDLELL ENSSQDNLDK DTISTEPFPN QKHKDLQQDL NDEPLEPFPT
201 QIHKDYKEK**N** **LINEEDSEPF** **PR**QKHKKVDN HNEEKNVFHE NGSANGNQGS
251 LKLKSFDEHL KDEKIENEPL VHENLSIPND PIEQILNQPE QETNIQEQLY
301 NEKQNVEEKQ NSQIPSLDLK EPTNEDILPN HNPLENIKQS ESEINHVQDH
351 ALPKENIIDK LDNQKEHIDQ SQHNINVLQE NNINNHQLEP QEKPNIESFE
401 PKNIDSEIIL PENVETEEII DDVPSPKHSN HETFEEETSE SEHEEAVSEK
451 NAHETVEHEE TVSQESNPEK ADNDGNVSQN SNNELNENEF VESEKSEHEP
501 AENEESSLEE GHHEEIVPEQ NNEESGESKL VDNDEGGFEE AHHENFSSEV
551 SNSELNENEF VESDKSVTEP AEHEEVVSEE SNPEPAENEE SSIEEAHQEE
601 IVPEQNDEES GESGLVDNEE GDFEEPNHEE FEPDQNDSEL SENELVESEK
651 **SVSEPAEHVE** **IVSEK**SVSEP AEHVEIVSEK STSEPAEHVE SVSEQSNNEP
701 SEKK**DGPVPS** **KPFEEIEKVD** **VQPK**IVDLQI IEPNFVDSQP NPQEPVEPSF
751 VKIEKVPSEE NKHASVDPEV KEKENVSEVV EEKQNSQESV EEIPVNEDEF
801 EDVHTEQLDL DHKTVDPEIV EVEEIPSELH ENEVAHPEIV EIEEVFPEPN
851 QNNEFQEINE DDKSAHIQHE IVEVEEILPE DDKNEKVEHE IVEVEEILPE
901 DKNEKVQHEI VEVEEILPED KNEKVEHEIV EVEEILPEDK NEK**GQHEIVE**

951 **VEEILPEDKN EK**VQHEIVEV EEILPEDKNE KGQHEIVEVE EILPEDKNEK
1001 VEHEIVEVEE ILPEDKNEKG QHEIVEVEEI LPEDDKNEKG QHEIVEVEEI
1051 LPEIVEIEEV PSQTNNNENI ETIKPEEKKN EFSVVEEKAI PQEPVVPTLN
1101 ENENVTPKPS EGESTKPDIV QIKIVQENKP NKKETPVVDG PKHVEQNIQE
1151 DDNDEEDDDD IDFEGLSRKD DEKDSSNKNK KK**SSFITYIS TK**KFKKVSQT
1201 IVSVMINAYD GVIQVVSTIK GIAKDIVIFF QNI

Peptides used for identification

Peptides shown in bold have been analysed by MS/MS sequencing

Start - End	Observed	Mr(expt)	Mr(calc)	Delta	Miss	Sequence
210 - 222	780.37	1558.72	1558.73	-2	0	**K.NLINEEDSEPFPR.Q** (Ions score 75)
210 - 222	780.37	1558.73	1558.73	-1	0	**K.NLINEEDSEPFPR.Q** (Ions score 72)
210 - 222	780.37	1558.73	1558.73	1	0	**K.NLINEEDSEPFPR.Q** (Ions score 79)
210 - 222	780.37	1558.73	1558.73	1	0	**K.NLINEEDSEPFPR.Q** (Ions score 25)
651 - 665	547.28	1638.81	1638.81	-2	0	**K.SVSEPAEHVEIVSEK.S** (Ions score 11)
651 - 665	547.28	1638.81	1638.81	-1	0	**K.SVSEPAEHVEIVSEK.S** (Ions score 32)
651 - 665	547.28	1638.81	1638.81	-1	0	**K.SVSEPAEHVEIVSEK.S** (Ions score 7)
651 - 665	820.41	1638.81	1638.81	0	0	**K.SVSEPAEHVEIVSEK.S** (Ions score 18)
705 - 724	746.73	2237.16	2237.16	2	1	**K.DGPVPSKPFEEIEKVDVQPK.I** (Ions score 18)
944 - 962	745.71	2234.11	2234.11	-1	1	**K.GQHEIVEVEEILPEDKNEK.V** (Ions score 62)
944 - 962	745.71	2234.11	2234.11	1	1	**K.GQHEIVEVEEILPEDKNEK.V** (Ions score 47)
944 - 962	745.71	2234.11	2234.11	1	1	**K.GQHEIVEVEEILPEDKNEK.V** (Ions score 68)
1183 - 1192	573.81	1145.60	1145.60	-1	0	**K.SSFITYISTK.K** (Ions score 18)
1183 - 1192	573.81	1145.60	1145.60	1	0	**K.SSFITYISTK.K** (Ions score 29)

Alphalyse A/S ■ Unsbjergvej 4 ■ 5220 Odense ■ Denmark
Tel: +45 6310 6500 ■ e-mail info@alphalyse.com
www.alphalyse.com

Sample name: 4

Protein Information

Protein name:	Uncharacterized protein OS=Plasmodium falciparum UGT5.1 GN=C923_03132 PE=4 SV=1
Entry name:	W7JXD3_PLAFA
Calculated MW:	124344
Calculated pI:	4.28
Mascot score:	214
Sequence coverage:	6%
Bioinformatic tools:	1: UniProt Entry 2: Conserved Domains in NCBI

Analysis Information

- Enzyme: Trypsin
- Variable modifications: Oxidation (M)
- Fixed modifications: Carbamidomethyl (C)
- Database search program: Mascot version 2.4
- Peptide Tolerance: 10 ppm
- Database: UniprotTREMBL (50011027 protein sequences)

Protein sequence

Matched peptides shown in bold underline

1 MRNLFHITIC LVTLNLFILE INAKTNTSEN RNKRIGGPKL RGNVTSNIKF
51 PSDNKGKIIR GSNDQLNKNS EDVLEQSEKS LVSENVPSGL DIDDIPKESI
101 FIQEDQEGQT HSELNPETSE HSKDLNNNGS KNESSDIISE NNKSNKVQNH
151 FESLSDLELL ENSSQDNLDK DTISTEPFPN QKHKDLQQDL NDEPLEPFPT
201 QIHKDYKEK**N** **LINEEDSEPF** **PR**QKHKKVDN HNEEKNVFHE NGSANGNQGS
251 LKLKSFDEHL KDEKIENEPL VHENLSIPND PIEQILNQPE QETNIQEQLY
301 NEKQNVEEKQ NSQIPSLDLK EPTNEDILPN HNPLENIKQS ESEINHVQDH
351 ALPKENIIDK LDNQKEHIDQ SQHNINVLQE NNINNHQLEP QEKPNIESFE
401 PKNIDSEIIL PENVETEEII DDVPSPKHSN HETFEEETSE SEHEEAVSEK
451 NAHETVEHEE TVSQESNPEK ADNDGNVSQN SNNELNENEF VESEKSEHEP
501 AENEESSLEE GHHEEIVPEQ NNEESGESKL VDNDEGGFEE AHHENFSSEV
551 SNSELNENEF VESDKSVTEP AEHEEVVSEE SNPEPAENEE SSIEEGHQEE
601 IVPEQNDEES GESGLVDNEE GDFEEPNHEE FEPDQNDSEL SENELVESEK
651 **SVSEPAEHVE** **IVSEKSASEP** **AEHVEIVSEK** SVSEPAEHVE SVSEQSNNEP
701 SEKK**DGPVPS** **KPFEEIEKVD** **VQPK**IVDLQI IEPNFVDSQP NPQEPVEPSF
751 VKIEKVPSEE NKHASVDPEV KEKENVSEVV EEKQNSQESV EEIPVNEDEF
801 EDVHTEQLDL DHKTVDPEIV EVEEIPSELH ENEVAHPEIV EIEEVFPEPN
851 QNNEFQEINE DDKSAHIQHE IVEVEEILPE DDKNEKVEEI LPEEDKNEKG
901 QHEIVEVEEI LPEIVEIEEV PSQTNNNENI ETIKPEEKKN EFSVEEKAIP

Alphalyse A/S ■ Unsbjergvej 4 ■ 5220 Odense ■ Denmark
Tel: +45 6310 6500 ■ e-mail info@alphalyse.com
www.alphalyse.com

951 QEPVVPTLNE NENVPPKPSE GESTKPDIVQ IKIVQENKPN KKETPVVDGP
1001 KHVEQNIQED DNDEEDDDDI DFEGLSRKDD EKDSSNKNKK K**SSFITYIST**
1051 **K**KFKKVSQTI VSVMINAYDG VIQVVSTIKG IAKDIVIFFQ NI

Peptides used for identification

Peptides shown in bold have been analysed by MS/MS sequencing

Start - End	Observed	Mr(expt)	Mr(calc)	Delta	Miss	Sequence
210 - 222	780.37	1558.72	1558.73	-2	0	**K.NLINEEDSEPFPR.Q** (Ions score 75)
210 - 222	780.37	1558.73	1558.73	-1	0	**K.NLINEEDSEPFPR.Q** (Ions score 72)
210 - 222	780.37	1558.73	1558.73	1	0	**K.NLINEEDSEPFPR.Q** (Ions score 79)
210 - 222	780.37	1558.73	1558.73	1	0	**K.NLINEEDSEPFPR.Q** (Ions score 25)
651 - 665	547.28	1638.81	1638.81	-2	0	**K.SVSEPAEHVEIVSEK.S** (Ions score 11)
651 - 665	547.28	1638.81	1638.81	-1	0	**K.SVSEPAEHVEIVSEK.S** (Ions score 32)
651 - 665	547.28	1638.81	1638.81	-1	0	**K.SVSEPAEHVEIVSEK.S** (Ions score 7)
651 - 665	820.41	1638.81	1638.81	0	0	**K.SVSEPAEHVEIVSEK.S** (Ions score 18)
666 - 680	806.39	1610.77	1610.78	-4	0	**K.SASEPAEHVEIVSEK.S** (Ions score 57)
666 - 680	537.93	1610.77	1610.78	-4	0	**K.SASEPAEHVEIVSEK.S** (Ions score 6)
666 - 680	806.39	1610.77	1610.78	-3	0	**K.SASEPAEHVEIVSEK.S** (Ions score 36)
705 - 724	746.73	2237.16	2237.16	2	1	**K.DGPVPSKPFEEIEKVDVQPK.I** (Ions score 18)
1042 - 1051	573.81	1145.60	1145.60	-1	0	**K.SSFITYISTK.K** (Ions score 18)
1042 - 1051	573.81	1145.60	1145.60	1	0	**K.SSFITYISTK.K** (Ions score 29)

Alphalyse A/S ■ Unsbjergvej 4 ■ 5220 Odense ■ Denmark
Tel: +45 6310 6500 ■ e-mail info@alphalyse.com
www.alphalyse.com

Sample name: 4

Protein Information

**Protein name:**	**Uncharacterized protein OS=Plasmodium falciparum Vietnam Oak-Knoll (FVO) GN=PFFVO_03053 PE=4 SV=1**
Entry name:	A0A024V579_PLAFA
Calculated MW:	94874
Calculated pI:	4.34
Mascot score:	174
Sequence coverage:	5%
Bioinformatic tools:	1: UniProt Entry 2: Conserved Domains in NCBI

Analysis Information

- Enzyme: Trypsin
- Variable modifications: Oxidation (M)
- Fixed modifications: Carbamidomethyl (C)
- Database search program: Mascot version 2.4
- Peptide Tolerance: 10 ppm
- Database: UniprotTREMBL (50011027 protein sequences)

Protein sequence

Matched peptides shown in bold underline

```
  1 MRNLFHITIC LVTLNLFILE ISAKTNTSEN RNKRIGGPKL RGNVTSNIKL
 51 PSNNKGKIIR GSNDELNKNS EDVLEQSEKS LVSENVPSGL DIDDIPKESI
101 FIQEDQEGQT HSELNPETSE HSKDLNNNDS KNESSDIISE NNKSNKVQNH
151 FESLSDLELL ENSSQDNLDK DTISTEPFPN QKHKDLQQDL NDEPLEPFPT
201 QIHKDYKEKN LINEEDSEPF PRQEHKKVDN HNEEKNVFHE NGSANGNQGS
251 LKLKSFDEHL KDEKIENEPL VHENLSIPND PIEQILNQPE QETNIQEQLY
301 NEKQNVEEKQ NSQIPSLDLK EPTNEDILPN HNPLENIKQS ESEINHVQDH
351 ALPKENIIDK LDNQKEHIDQ SQHNINVLQE NNINNHQLEP QEKPNIESFE
401 PKNIDSEIIL PENVETEEII DDVPSPKHSN HETFEEETSE SEHEEAVSEK
451 NAHETVEHEE TVSQESNPEK ADNDGNVSQN SNNELNENEF VESEKSEHEA
501 AENEESSLEE GHHEEIVPEQ NNEESGESKL VDNDEGGFEE AHHENFSSEV
551 SNSELNENEF VESDKSVTEP AEHEEVVSEE SNPEPAENEE SSIEEGHQEE
601 IVPEQNDEES GESGLVDNEE GDFEEPNHEE FEPDQNDSEL SENELVESEK
651 SVSEPAEHVE IVPSQTNNNE NIETIKPEEK KNEFSVEEKA IPQEPVVPTL
701 NENENVPPKP SEGESTKPDI VQIKIVQENK PNKKETPVVD GPKHVEQNIQ
751 EDDNDEEDDD DIDFEGLSRK DDEKDSSNKN KKKSSFITYI STKKFKKVSQ
801 TIVSVMINAY DGVIQVVSTI KGIAKDIVIF FQNI
```

Matched peptides (bold underline): GSNDELNKNS EDVLEQSEK (61–79); N LINEEDSEPF PR (210–222); SSFITYI STK (784–793)

Alphalyse A/S ■ Unsbjergvej 4 ■ 5220 Odense ■ Denmark
Tel: +45 6310 6500 ■ e-mail info@alphalyse.com
www.alphalyse.com

Peptides used for identification

Peptides shown in bold have been analysed by MS/MS sequencing

Start - End	Observed	Mr(expt)	Mr(calc)	Delta	Miss	Sequence
61 - 79	712.33	2133.97	2133.97	1	1	R.GSNDELNKNSEDVLEQSEK.S (Ions score 35)
61 - 79	712.33	2133.97	2133.97	1	1	R.GSNDELNKNSEDVLEQSEK.S (Ions score 10)
61 - 79	712.33	2133.97	2133.97	3	1	R.GSNDELNKNSEDVLEQSEK.S (Ions score 67)
210 - 222	780.37	1558.72	1558.73	-2	0	K.NLINEEDSEPFPR.Q (Ions score 75)
210 - 222	780.37	1558.73	1558.73	-1	0	K.NLINEEDSEPFPR.Q (Ions score 72)
210 - 222	780.37	1558.73	1558.73	1	0	K.NLINEEDSEPFPR.Q (Ions score 79)
210 - 222	780.37	1558.73	1558.73	1	0	K.NLINEEDSEPFPR.Q (Ions score 25)
784 - 793	573.81	1145.60	1145.60	-1	0	K.SSFITYISTK.K (Ions score 18)
784 - 793	573.81	1145.60	1145.60	1	0	K.SSFITYISTK.K (Ions score 29)

Alphalyse A/S ■ Unsbjergvej 4 ■ 5220 Odense ■ Denmark
Tel: +45 6310 6500 ■ e-mail info@alphalyse.com
www.alphalyse.com

Sample name: 6

Protein Information

Protein name:	Gp195 surface antigen preprotein OS=Plasmodium falciparum GN=msp1 PE=2 SV=1
Entry name:	Q6LBT0_PLAFA
Calculated MW:	188643
Calculated pI:	5.98
Mascot score:	4123
Sequence coverage:	43%
Bioinformatic tools:	1: UniProt Entry 2: Conserved Domains in NCBI

Analysis Information

- Enzyme: Trypsin
- Variable modifications: Oxidation (M)
- Fixed modifications: Carbamidomethyl (C)
- Database search program: Mascot version 2.4
- Peptide Tolerance: 10 ppm
- Database: UniprotTREMBL (50011027 protein sequences)

Protein sequence

Matched peptides shown in bold underline

1 MKIIFFLCSF LFFIINTQCV THESYQELVK **KLEALEDAVL** **TGYSLFQK**EK

51 **MVLNEGTSGT** **AVTTSTPGSK** **GSVASGGSGG** **SVASGGSVAS** **GGSVASGGSV**

101 **ASGGSGNSRR** TNPSDNSSDS DAKSYADLKH RVR**NYLLTIK** ELKYPQLFDL

151 TNHMLTLCDN IHGFK**YLIDG** **YEEINELLYK** **LNFYFDLLR**A KLNDVCANDY

201 CQIPFNLKIR **ANELDVLKKL** **VFGYR**KPLDN IKDNVGKMED YIKKNKK**TIE**

251 **NINELIEESK** **K**TIDKNKNAT KEEEKKK**LYQ** **AQYDLSIYNK** **QLEEAHNLIS**

301 **VLEKR**IDTLK KNENIKELLD KINEIKNPPP ANSGNTPNTL LDKNKKIEEH

351 EKEIKEIAKT IKFNIDSLFT DPLELEYYLR EKNKNIDISA KVETKESTEP

401 NEYPNGVTYP LSYNDINNAL NELNSFGDLI NPFDYTKEPS K**NIYTDNER**K

451 K**FINEIKEK**I KIEKKKIESD KKSYEDRSK**S** **LNDITKEYEK** **LLNEIYDSK**F

501 NNNIDLTNFE KMMGKRYSYK VEK**LTHHNTF** **ASYENSK**HNL EKLTKALK**YM**

551 **EDYSLRNIVV** **EKELK**YYKNL ISKIENEIET LVENIKKDEE QLFEKKITKD

601 ENKPDEK**ILE** **VSDIVK**VQVQ KVLLMNKIDE LKKTQLILKN VELKHNIHVP

651 NSYKQENKQE PYYLIVLKKE IDKLKVFMPK **VESLINEEKK** NIK**TEGQSDN**

701 **SEPSTEGEIT** **GQATTKPGQQ** **AGSALEGDSV** **QAQAQEQKQA** **QPPVPVPVPE**

751 **AK**AQVPTPPA PVNNK**TENVS** **KLDYLEK**LYE FLNTSYICHK **YILVSHSTMN**

801 **EK**ILKQYKIT KEEESKLSSC DPLDLLFNIQ NNIPVMYSMF DSLNNSLSQL

851 FMEIYEKEMV CNLYKLKDND KIK**NLLEEAK** **K**VSTSVK**TLS** **SSSMQPLSLT**

901 **PQDKPEVSAN** **DDTSHSTNLN** **NSLKLFENIL** **SLGKNKNIYQ** **ELIGQK**SSEN

951 FYEK**ILKDSD** **TFYNESFTNF** **VK**SKADDINS LNDESKRKKL EEDINKLKKT

Alphalyse A/S ■ Unsbjergvej 4 ■ 5220 Odense ■ Denmark
Tel: +45 6310 6500 ■ e-mail info@alphalyse.com
www.alphalyse.com

```
1001 LQLSFDLYNK YKLKLERLFD KKKTVGKYKM QIKKLTLLKE QLESKLNSLN
1051 NPKHVLQNFS VFFNKKKEAE IAETENTLEN TKILLKHYKG LVKYYNGESS
1101 PLKTLSEESI QTEDNYASLE NFKVLSKLEG KLKDNLNLEK KKLSYLSSGL
1151 HHLIAELKEV IKNKNYTGNS PSENNTDVNN ALESYKKFLP EGTDVATVVS
1201 ESGSDTLEQS QPKKPASTHV GAESNTITTS QNVDDEVDDV IIVPIFGESE
1251 EDYDDLGQVV TGEAVTPSVI DNILSKIENE YEVLYLKPLA GVYRSLKKQL
1301 ENNVMTFNVN VKDILNSRFN KRENFKNVLE SDLIPYKDLT SSNYVVKDPY
1351 KFLNKEKRDK FLSSYNYIKD SIDTDINFAN DVLGYYKILS EKYKSDLDSI
1401 KKYINDKQGE NEKYLPFLNN IETLYKTVND KIDLFVIHLE AKVLNYTYEK
1451 SNVEVKIKEL NYLKTIQDKL ADFKKNNNFV GIADLSTDYN HNNLLTKFLS
1501 TGMVFENLAK TVLSNLLDGN LQGMLNISQH QCVKKQCPQN SGCFRHLDER
1551 EECKCLLNYK QEGDKCVENP NPTCNENNGG CDADAKCTEE DSGSNGKKIT
1601 CECTKPDSYP LFDGIFCSSS NFLGISFLLI LMLILYSFI
```

Peptides used for identification

Peptides shown in bold have been analysed by MS/MS sequencing

Start - End	Observed	Mr(expt)	Mr(calc)	Delta	Miss	Sequence
31 - 48	675.70	2024.08	2024.08	-3	1	K.KLEALEDAVLTGYSLFQK.E (Ions score 72)
31 - 48	675.70	2024.08	2024.08	-1	1	K.KLEALEDAVLTGYSLFQK.E (Ions score 76)
31 - 48	1013.05	2024.08	2024.08	0	1	K.KLEALEDAVLTGYSLFQK.E (Ions score 89)
31 - 48	675.70	2024.08	2024.08	0	1	K.KLEALEDAVLTGYSLFQK.E (Ions score 88)
31 - 48	1013.05	2024.08	2024.08	0	1	K.KLEALEDAVLTGYSLFQK.E (Ions score 71)
31 - 48	1013.05	2024.09	2024.08	1	1	K.KLEALEDAVLTGYSLFQK.E (Ions score 90)
51 - 70	977.47	1952.93	1952.94	-2	0	K.MVLNEGTSGTAVTTSTPGSK.G + Oxidation (M) (Ions score 147)
51 - 70	977.47	1952.93	1952.94	-2	0	K.MVLNEGTSGTAVTTSTPGSK.G + Oxidation (M) (Ions score 166)
51 - 70	977.47	1952.93	1952.94	-1	0	K.MVLNEGTSGTAVTTSTPGSK.G + Oxidation (M) (Ions score 167)
71 - 109	1023.81	3068.40	3068.40	-1	0	K.GSVASGGSGGSVASGGSVASGGSVASGGSVASGGSGNSR.R (Ions score 133)
71 - 109	1023.81	3068.40	3068.40	-1	0	K.GSVASGGSGGSVASGGSVASGGSVASGGSVASGGSGNSR.R (Ions score 100)
71 - 109	1023.81	3068.40	3068.40	0	0	K.GSVASGGSGGSVASGGSVASGGSVASGGSVASGGSGNSR.R (Ions score 153)
71 - 110	1075.84	3224.50	3224.50	-1	1	K.GSVASGGSGGSVASGGSVASGGSVASGGSVASGGSGNSRR.T (Ions score 47)
71 - 110	1075.84	3224.50	3224.50	1	1	K.GSVASGGSGGSVASGGSVASGGSVASGGSVASGGSGNSRR.T (Ions score 43)
134 - 140	432.76	863.51	863.51	-4	0	R.NYLLTIK.E (Ions score 19)
166 - 180	937.97	1873.93	1873.93	-1	0	K.YLIDGYEEINELLYK.L (Ions score 78)
166 - 180	937.98	1873.94	1873.93	1	0	K.YLIDGYEEINELLYK.L (Ions score 90)
166 - 180	937.98	1873.94	1873.93	1	0	K.YLIDGYEEINELLYK.L (Ions score 63)
181 - 189	600.82	1199.63	1199.63	-1	0	K.LNFYFDLLR.A (Ions score 58)
181 - 189	600.82	1199.63	1199.63	1	0	K.LNFYFDLLR.A (Ions score 58)
211 - 218	451.25	900.49	900.49	-3	0	R.ANELDVLK.K (Ions score 7)
211 - 218	451.25	900.49	900.49	-2	0	R.ANELDVLK.K (Ions score 10)
211 - 219	515.30	1028.58	1028.59	-6	1	R.ANELDVLKK.L (Ions score 22)
211 - 219	515.30	1028.58	1028.59	-5	1	R.ANELDVLKK.L (Ions score 14)
219 - 225	441.76	881.51	881.51	-2	1	K.KLVFGYR.K (Ions score 26)
219 - 225	441.76	881.51	881.51	-1	1	K.KLVFGYR.K (Ions score 31)
248 - 261	553.96	1658.87	1658.87	-3	1	K.TIENINELIEESKK.T (Ions score 63)
248 - 261	553.96	1658.87	1658.87	-2	1	K.TIENINELIEESKK.T (Ions score 61)
248 - 261	553.96	1658.87	1658.87	-1	1	K.TIENINELIEESKK.T (Ions score 63)
248 - 261	830.44	1658.87	1658.87	-1	1	K.TIENINELIEESKK.T (Ions score 67)
248 - 261	830.44	1658.87	1658.87	-1	1	K.TIENINELIEESKK.T (Ions score 76)
278 - 290	809.91	1617.80	1617.80	-2	0	K.LYQAQYDLSIYNK.Q (Ions score 73)
278 - 290	809.91	1617.80	1617.80	-1	0	K.LYQAQYDLSIYNK.Q (Ions score 83)
278 - 290	809.91	1617.80	1617.80	0	0	K.LYQAQYDLSIYNK.Q (Ions score 77)
291 - 304	541.63	1621.86	1621.87	-2	0	K.QLEEAHNLISVLEK.R (Ions score 10)
291 - 304	811.94	1621.87	1621.87	-1	0	K.QLEEAHNLISVLEK.R (Ions score 98)

Alphalyse A/S ■ Unsbjergvej 4 ■ 5220 Odense ■ Denmark
Tel: +45 6310 6500 ■ e-mail info@alphalyse.com
www.alphalyse.com

291 - 304	541.63	1621.87	1621.87	-1	0	K.QLEEAHNLISVLEK.R (Ions score 8)
291 - 304	811.94	1621.87	1621.87	-1	0	K.QLEEAHNLISVLEK.R (Ions score 93)
291 - 305	593.66	1777.96	1777.97	-2	1	K.QLEEAHNLISVLEKR.I (Ions score 64)
291 - 305	593.66	1777.97	1777.97	-2	1	K.QLEEAHNLISVLEKR.I (Ions score 61)
291 - 305	593.66	1777.97	1777.97	-1	1	K.QLEEAHNLISVLEKR.I (Ions score 59)
442 - 449	512.74	1023.46	1023.46	0	0	K.NIYTDNER.K (Ions score 40)
452 - 459	510.79	1019.56	1019.57	-4	1	K.FINEIKEK.I (Ions score 22)
452 - 459	510.79	1019.56	1019.57	-3	1	K.FINEIKEK.I (Ions score 17)
452 - 459	510.79	1019.56	1019.57	-2	1	K.FINEIKEK.I (Ions score 15)
480 - 490	670.34	1338.66	1338.67	-1	1	K.SLNDITKEYEK.L (Ions score 51)
491 - 499	547.79	1093.56	1093.57	-3	0	K.LLNEIYDSK.F (Ions score 36)
491 - 499	547.79	1093.56	1093.57	-3	0	K.LLNEIYDSK.F (Ions score 35)
524 - 537	550.26	1647.76	1647.76	-3	0	K.LTHHNTFASYENSK.H (Ions score 5)
549 - 556	538.74	1075.46	1075.46	-3	0	K.YMEDYSLR.N (Ions score 35)
549 - 556	538.74	1075.46	1075.46	-1	0	K.YMEDYSLR.N (Ions score 37)
549 - 556	546.74	1091.46	1091.46	-1	0	K.YMEDYSLR.N + Oxidation (M) (Ions score 31)
549 - 556	546.74	1091.46	1091.46	0	0	K.YMEDYSLR.N + Oxidation (M) (Ions score 46)
557 - 565	536.32	1070.63	1070.63	-3	1	R.NIVVEKELK.Y (Ions score 14)
557 - 565	536.32	1070.63	1070.63	-1	1	R.NIVVEKELK.Y (Ions score 17)
557 - 565	536.32	1070.63	1070.63	-1	1	R.NIVVEKELK.Y (Ions score 21)
608 - 616	508.30	1014.59	1014.60	-3	0	K.ILEVSDIVK.V (Ions score 30)
608 - 616	508.30	1014.60	1014.60	-1	0	K.ILEVSDIVK.V (Ions score 33)
608 - 616	508.31	1014.60	1014.60	-1	0	K.ILEVSDIVK.V (Ions score 26)
681 - 689	530.78	1059.54	1059.54	-3	0	K.VESLINEEK.K (Ions score 28)
681 - 690	594.83	1187.64	1187.64	0	1	K.VESLINEEKK.N (Ions score 46)
694 - 738	1144.53	4574.07	4574.08	-1	0	K.TEGQSDNSEPSTEGEITGQATTKPGQQAGSALEGDSVQAQAQEQK.Q (Ions score 188)
694 - 738	1144.53	4574.08	4574.08	1	0	K.TEGQSDNSEPSTEGEITGQATTKPGQQAGSALEGDSVQAQAQEQK.Q (Ions score 132)
739 - 752	728.91	1455.81	1455.81	-1	0	K.QAQPPVPVPVPEAK.A (Ions score 59)
739 - 752	728.91	1455.81	1455.81	-1	0	K.QAQPPVPVPVPEAK.A (Ions score 63)
739 - 752	728.91	1455.81	1455.81	0	0	K.QAQPPVPVPVPEAK.A (Ions score 66)
766 - 777	719.87	1437.73	1437.74	-2	1	K.TENVSKLDYLEK.L (Ions score 64)
766 - 777	719.87	1437.73	1437.74	-1	1	K.TENVSKLDYLEK.L (Ions score 39)
791 - 802	719.36	1436.70	1436.70	-1	0	K.YILVSHSTMNEK.I + Oxidation (M) (Ions score 50)
874 - 881	472.77	943.53	943.53	-2	1	K.NLLEEAKK.V (Ions score 35)
874 - 881	472.77	943.53	943.53	-2	1	K.NLLEEAKK.V (Ions score 15)
874 - 881	472.77	943.53	943.53	-1	1	K.NLLEEAKK.V (Ions score 17)
888 - 924	990.72	3958.87	3958.87	-1	0	K.TLSSSSMQPLSLTPQDKPEVSANDDTSHSTNLNNSLK.L + Oxidation (M) (Ions score 171)
888 - 924	990.72	3958.87	3958.87	0	0	K.TLSSSSMQPLSLTPQDKPEVSANDDTSHSTNLNNSLK.L + Oxidation (M) (Ions score 150)
888 - 924	990.73	3958.87	3958.87	0	0	K.TLSSSSMQPLSLTPQDKPEVSANDDTSHSTNLNNSLK.L + Oxidation (M) (Ions score 163)
925 - 934	567.33	1132.65	1132.65	0	0	K.LFENILSLGK.N (Ions score 61)
925 - 934	567.33	1132.65	1132.65	0	0	K.LFENILSLGK.N (Ions score 54)
925 - 934	567.33	1132.65	1132.65	1	0	K.LFENILSLGK.N (Ions score 50)
935 - 946	724.40	1446.78	1446.78	-2	1	K.NKNIYQELIGQK.S (Ions score 67)
935 - 946	724.40	1446.78	1446.78	-1	1	K.NKNIYQELIGQK.S (Ions score 52)
937 - 946	603.33	1204.64	1204.65	-2	0	K.NIYQELIGQK.S (Ions score 43)
937 - 946	603.33	1204.64	1204.65	-2	0	K.NIYQELIGQK.S (Ions score 44)
937 - 946	603.33	1204.65	1204.65	0	0	K.NIYQELIGQK.S (Ions score 39)
955 - 972	723.36	2167.05	2167.05	-1	1	K.ILKDSDTFYNESFTNFVK.S (Ions score 60)
955 - 972	723.36	2167.05	2167.05	1	1	K.ILKDSDTFYNESFTNFVK.S (Ions score 62)
958 - 972	907.40	1812.79	1812.78	1	0	K.DSDTFYNESFTNFVK.S (Ions score 80)
958 - 972	907.40	1812.79	1812.78	1	0	K.DSDTFYNESFTNFVK.S (Ions score 106)
958 - 972	907.40	1812.79	1812.78	1	0	K.DSDTFYNESFTNFVK.S (Ions score 59)
1035 - 1045	651.39	1300.76	1300.76	-1	1	K.LTLLKEQLESK.L (Ions score 57)
1054 - 1065	740.39	1478.77	1478.77	0	0	K.HVLQNFSVFFNK.K (Ions score 73)
1054 - 1065	740.39	1478.77	1478.77	0	0	K.HVLQNFSVFFNK.K (Ions score 68)
1054 - 1065	740.39	1478.77	1478.77	0	0	K.HVLQNFSVFFNK.K (Ions score 82)

Alphalyse A/S ■ Unsbjergvej 4 ■ 5220 Odense ■ Denmark
Tel: +45 6310 6500 ■ e-mail info@alphalyse.com
www.alphalyse.com

1068 - 1082	846.40	1690.79	1690.79	0	0	K.EAEIAETENTLENTK.I (Ions score 109)
1068 - 1082	846.40	1690.79	1690.79	0	0	K.EAEIAETENTLENTK.I (Ions score 102)
1068 - 1082	846.40	1690.79	1690.79	0	0	K.EAEIAETENTLENTK.I (Ions score 109)
1094 - 1103	579.28	1156.54	1156.54	-4	0	K.YYNGESSPLK.T (Ions score 50)
1094 - 1103	579.28	1156.54	1156.54	-2	0	K.YYNGESSPLK.T (Ions score 63)
1094 - 1103	579.28	1156.54	1156.54	-1	0	K.YYNGESSPLK.T (Ions score 63)
1104 - 1123	1159.54	2317.06	2317.06	-1	0	K.TLSEESIQTEDNYASLENFK.V (Ions score 169)
1104 - 1123	773.36	2317.06	2317.06	-1	0	K.TLSEESIQTEDNYASLENFK.V (Ions score 83)
1104 - 1123	1159.54	2317.06	2317.06	-1	0	K.TLSEESIQTEDNYASLENFK.V (Ions score 147)
1104 - 1123	773.36	2317.06	2317.06	0	0	K.TLSEESIQTEDNYASLENFK.V (Ions score 81)
1104 - 1123	1159.54	2317.06	2317.06	0	0	K.TLSEESIQTEDNYASLENFK.V (Ions score 154)
1124 - 1131	437.27	872.53	872.53	-2	1	K.VLSKLEGK.L (Ions score 28)
1124 - 1131	437.27	872.53	872.53	-1	1	K.VLSKLEGK.L (Ions score 18)
1132 - 1140	543.81	1085.60	1085.61	-3	1	K.LKDNLNLEK.K (Ions score 13)
1132 - 1140	543.81	1085.61	1085.61	-2	1	K.LKDNLNLEK.K (Ions score 16)
1132 - 1140	543.81	1085.61	1085.61	-1	1	K.LKDNLNLEK.K (Ions score 19)
1165 - 1187	853.72	2558.15	2558.15	-1	1	K.NYTGNSPSENNTDVNNALESYKK.F (Ions score 90)
1165 - 1187	853.72	2558.15	2558.15	-1	1	K.NYTGNSPSENNTDVNNALESYKK.F (Ions score 109)
1165 - 1187	853.72	2558.15	2558.15	0	1	K.NYTGNSPSENNTDVNNALESYKK.F (Ions score 142)
1188 - 1213	1361.16	2720.30	2720.30	-2	0	K.FLPEGTDVATVVSESGSDTLEQSQPK.K (Ions score 153)
1188 - 1213	907.77	2720.30	2720.30	-1	0	K.FLPEGTDVATVVSESGSDTLEQSQPK.K (Ions score 95)
1188 - 1213	907.77	2720.30	2720.30	-1	0	K.FLPEGTDVATVVSESGSDTLEQSQPK.K (Ions score 115)
1188 - 1213	1361.16	2720.30	2720.30	-1	0	K.FLPEGTDVATVVSESGSDTLEQSQPK.K (Ions score 140)
1188 - 1213	907.77	2720.30	2720.30	-1	0	K.FLPEGTDVATVVSESGSDTLEQSQPK.K (Ions score 98)
1188 - 1213	1361.16	2720.30	2720.30	0	0	K.FLPEGTDVATVVSESGSDTLEQSQPK.K (Ions score 124)
1277 - 1294	723.72	2168.15	2168.15	-3	0	K.IENEYEVLYLKPLAGVYR.S (Ions score 119)
1277 - 1294	1085.08	2168.15	2168.15	-1	0	K.IENEYEVLYLKPLAGVYR.S (Ions score 83)
1277 - 1294	1085.08	2168.15	2168.15	0	0	K.IENEYEVLYLKPLAGVYR.S (Ions score 80)
1277 - 1294	1085.08	2168.15	2168.15	0	0	K.IENEYEVLYLKPLAGVYR.S (Ions score 53)
1277 - 1294	723.72	2168.15	2168.15	0	0	K.IENEYEVLYLKPLAGVYR.S (Ions score 118)
1277 - 1294	723.72	2168.15	2168.15	0	0	K.IENEYEVLYLKPLAGVYR.S (Ions score 99)
1299 - 1312	833.42	1664.82	1664.82	-1	0	K.QLENNVMTFNVNVK.D + Oxidation (M) (Ions score 90)
1299 - 1312	833.42	1664.82	1664.82	0	0	K.QLENNVMTFNVNVK.D + Oxidation (M) (Ions score 93)
1299 - 1312	833.42	1664.82	1664.82	0	0	K.QLENNVMTFNVNVK.D + Oxidation (M) (Ions score 81)
1299 - 1318	788.74	2363.19	2363.19	-2	1	K.QLENNVMTFNVNVKDILNSR.F + Oxidation (M) (Ions score 140)
1299 - 1318	788.74	2363.19	2363.19	-1	1	K.QLENNVMTFNVNVKDILNSR.F + Oxidation (M) (Ions score 162)
1299 - 1318	788.74	2363.19	2363.19	0	1	K.QLENNVMTFNVNVKDILNSR.F + Oxidation (M) (Ions score 143)
1327 - 1337	645.85	1289.69	1289.69	-1	0	K.NVLESDLIPYK.D (Ions score 27)
1327 - 1347	799.75	2396.24	2396.25	-3	1	K.NVLESDLIPYKDLTSSNYVVK.D (Ions score 47)
1327 - 1347	799.75	2396.24	2396.25	-2	1	K.NVLESDLIPYKDLTSSNYVVK.D (Ions score 38)
1370 - 1387	1031.98	2061.95	2061.95	-3	0	K.DSIDTDINFANDVLGYYK.I (Ions score 93)
1370 - 1387	1031.98	2061.95	2061.95	0	0	K.DSIDTDINFANDVLGYYK.I (Ions score 66)
1370 - 1387	1031.98	2061.95	2061.95	0	0	K.DSIDTDINFANDVLGYYK.I (Ions score 124)
1408 - 1426	771.73	2312.17	2312.17	-1	1	K.QGENEKYLPFLNNIETLYK.T (Ions score 62)
1408 - 1426	771.73	2312.17	2312.17	-1	1	K.QGENEKYLPFLNNIETLYK.T (Ions score 79)
1414 - 1426	814.44	1626.86	1626.87	-2	0	K.YLPFLNNIETLYK.T (Ions score 79)
1414 - 1426	814.44	1626.86	1626.87	-1	0	K.YLPFLNNIETLYK.T (Ions score 79)
1414 - 1426	814.44	1626.86	1626.87	-1	0	K.YLPFLNNIETLYK.T (Ions score 77)
1427 - 1442	619.01	1854.02	1854.03	-3	1	K.TVNDKIDLFVIHLEAK.V (Ions score 88)
1427 - 1442	619.01	1854.02	1854.03	-3	1	K.TVNDKIDLFVIHLEAK.V (Ions score 70)
1443 - 1450	515.27	1028.53	1028.52	7	0	K.VLNYTYEK.S (Ions score 9)
1457 - 1464	510.81	1019.60	1019.60	-2	1	K.IKELNYLK.T (Ions score 9)
1457 - 1464	510.81	1019.60	1019.60	-1	1	K.IKELNYLK.T (Ions score 9)
1476 - 1497	826.40	2476.19	2476.20	-3	0	K.NNNFVGIADLSTDYNHNNLLTK.F (Ions score 138)
1476 - 1497	826.41	2476.19	2476.20	-1	0	K.NNNFVGIADLSTDYNHNNLLTK.F (Ions score 138)
1476 - 1497	826.41	2476.20	2476.20	0	0	K.NNNFVGIADLSTDYNHNNLLTK.F (Ions score 128)
1498 - 1510	728.88	1455.74	1455.74	-1	0	K.FLSTGMVFENLAK.T (Ions score 60)
1498 - 1510	728.88	1455.74	1455.74	0	0	K.FLSTGMVFENLAK.T (Ions score 72)
1498 - 1510	728.88	1455.74	1455.74	0	0	K.FLSTGMVFENLAK.T (Ions score 72)
1498 - 1510	736.88	1471.74	1471.74	0	0	K.FLSTGMVFENLAK.T + Oxidation (M) (Ions score 79)
1498 - 1510	736.88	1471.74	1471.74	0	0	K.FLSTGMVFENLAK.T + Oxidation (M) (Ions score 71)

Alphalyse A/S ■ Unsbjergvej 4 ■ 5220 Odense ■ Denmark
Tel: +45 6310 6500 ■ e-mail info@alphalyse.com
www.alphalyse.com

1498 - 1510	736.88	1471.74	1471.74	1	0	K.FLSTGMVFENLAK.T + Oxidation (M) (Ions score 58)

Alphalyse A/S ■ Unsbjergvej 4 ■ 5220 Odense ■ Denmark
Tel: +45 6310 6500 ■ e-mail info@alphalyse.com
www.alphalyse.com

LC-MSMS Protein Identification Report

Order 16372_Plasmodium falciparum

Sample name: 6

Protein Information

Protein name:	Merozoite surface protein 1 (Fragment) OS=Plasmodium falciparum GN=MSP-1 PE=2 SV=1
Entry name:	Q8T6A9_PLAFA
Calculated MW:	75823
Calculated pI:	7.26
Mascot score:	1315
Sequence coverage:	38%
Bioinformatic tools:	1: UniProt Entry 2: Conserved Domains in NCBI

Analysis Information

- Enzyme: Trypsin
- Variable modifications: Oxidation (M)
- Fixed modifications: Carbamidomethyl (C)
- Database search program: Mascot version 2.4
- Peptide Tolerance: 10 ppm
- Database: UniprotTREMBL (50011027 protein sequences)

Protein sequence

Matched peptides shown in bold underline

1 **VTHESYQELV** **KKLEALEDAV** **LTGYSLFQK**E K**MVLNEGTSG** **TAVTTSTPGS**
51 **KGSVASGGSG** **GSVASGGSVA** **SGGSVASGGS** **VASGGSGNSR** **R**TNPSDNSSD
101 SDAKSYADLK HRVR**NYLLTI** **K**ELKYPQLFD LTNHMLTLCD NIHGFK**YLID**
151 **GYEEINELLY** **KLNFYFDLLR** AKLNDVCAND YCQIPFNLKI R**ANELDVLKK**
201 **LVFGYR**KPLD NIKDNVGKME DYIKKNKK**TI** **ENINELIEES** **KK**TIDKNKNA
251 TKEEEKKK**LY** **QAQYDLSIYN** **KQLEEAHNLI** **SVLEKR**IDTL KKNENIKELL
301 DKINEIKNPP PANSGNTPNT LLDKNKKIEE HEKEIKEIAK TIKFNIDSLF
351 TDPLELEYYL REKNKNIDIS AKVETKESTE PNEYPNGVTY PLSYNDINNA
401 LNELNSFGDL INPFDYTKEP SK**NIYTDNER** KK**FINEIKEK** IKIEKKKIES
451 DKKSYEDRSK **SLNDITKEYE** **KLLNEIYDSK** FNNNIDLTNF EKMMGKRYSY
501 KVEK**LTHHNT** **FASYENSK**HN LEKLTKALK**Y** **MEDYSLRNIV** **VEKELK**YYKN
551 LISKIENEIE TLVENIKKDE EQLFEKKITK DENKPDEK**IL** **EVSDIVK**VQV
601 QKVLLMNKID ELKKTQLILK NVELKHNIHV PNSYKQENKQ EPYYLIVLKK
651 EIDKLK

Peptides used for identification

Peptides shown in bold have been analysed by MS/MS sequencing

Start - End Observed Mr(expt) Mr(calc) Delta Miss Sequence

Alphalyse A/S ■ Unsbjergvej 4 ■ 5220 Odense ■ Denmark
Tel: +45 6310 6500 ■ e-mail info@alphalyse.com
www.alphalyse.com

1 - 11	444.90	1331.67	1331.67	-5	0	-.VTHESYQELVK.K (Ions score 10)
1 - 11	444.90	1331.67	1331.67	-1	0	-.VTHESYQELVK.K (Ions score 7)
1 - 11	666.84	1331.67	1331.67	-1	0	-.VTHESYQELVK.K (Ions score 29)
1 - 11	666.84	1331.67	1331.67	-1	0	-.VTHESYQELVK.K (Ions score 54)
12 - 29	675.70	2024.08	2024.08	-3	1	K.KLEALEDAVLTGYSLFQK.E (Ions score 72)
12 - 29	675.70	2024.08	2024.08	-1	1	K.KLEALEDAVLTGYSLFQK.E (Ions score 76)
12 - 29	1013.05	2024.08	2024.08	0	1	K.KLEALEDAVLTGYSLFQK.E (Ions score 89)
12 - 29	675.70	2024.08	2024.08	0	1	K.KLEALEDAVLTGYSLFQK.E (Ions score 88)
12 - 29	1013.05	2024.08	2024.08	0	1	K.KLEALEDAVLTGYSLFQK.E (Ions score 71)
12 - 29	1013.05	2024.09	2024.08	1	1	K.KLEALEDAVLTGYSLFQK.E (Ions score 90)
32 - 51	977.47	1952.93	1952.94	-2	0	K.MVLNEGTSGTAVTTSTPGSK.G + Oxidation (M) (Ions score 147)
32 - 51	977.47	1952.93	1952.94	-2	0	K.MVLNEGTSGTAVTTSTPGSK.G + Oxidation (M) (Ions score 166)
32 - 51	977.47	1952.93	1952.94	-1	0	K.MVLNEGTSGTAVTTSTPGSK.G + Oxidation (M) (Ions score 167)
52 - 90	1023.81	3068.40	3068.40	-1	0	K.GSVASGGSGGSVASGGSVASGGSVASGGSVASGGSGNSR.R (Ions score 133)
52 - 90	1023.81	3068.40	3068.40	-1	0	K.GSVASGGSGGSVASGGSVASGGSVASGGSVASGGSGNSR.R (Ions score 100)
52 - 90	1023.81	3068.40	3068.40	0	0	K.GSVASGGSGGSVASGGSVASGGSVASGGSVASGGSGNSR.R (Ions score 153)
52 - 91	1075.84	3224.50	3224.50	-1	1	K.GSVASGGSGGSVASGGSVASGGSVASGGSVASGGSGNSRR.T (Ions score 47)
52 - 91	1075.84	3224.50	3224.50	1	1	K.GSVASGGSGGSVASGGSVASGGSVASGGSVASGGSGNSRR.T (Ions score 43)
115 - 121	432.76	863.51	863.51	-4	0	R.NYLLTIK.E (Ions score 19)
147 - 161	937.97	1873.93	1873.93	-1	0	K.YLIDGYEEINELLYK.L (Ions score 78)
147 - 161	937.98	1873.94	1873.93	1	0	K.YLIDGYEEINELLYK.L (Ions score 90)
147 - 161	937.98	1873.94	1873.93	1	0	K.YLIDGYEEINELLYK.L (Ions score 63)
162 - 170	600.82	1199.63	1199.63	-1	0	K.LNFYFDLLR.A (Ions score 58)
162 - 170	600.82	1199.63	1199.63	1	0	K.LNFYFDLLR.A (Ions score 58)
192 - 199	451.25	900.49	900.49	-3	0	R.ANELDVLK.K (Ions score 7)
192 - 199	451.25	900.49	900.49	-2	0	R.ANELDVLK.K (Ions score 10)
192 - 200	515.30	1028.58	1028.59	-6	1	R.ANELDVLKK.L (Ions score 22)
192 - 200	515.30	1028.58	1028.59	-5	1	R.ANELDVLKK.L (Ions score 14)
200 - 206	441.76	881.51	881.51	-2	1	K.KLVFGYR.K (Ions score 26)
200 - 206	441.76	881.51	881.51	-1	1	K.KLVFGYR.K (Ions score 31)
229 - 242	553.96	1658.87	1658.87	-3	1	K.TIENINELIEESKK.T (Ions score 63)
229 - 242	553.96	1658.87	1658.87	-2	1	K.TIENINELIEESKK.T (Ions score 61)
229 - 242	553.96	1658.87	1658.87	-1	1	K.TIENINELIEESKK.T (Ions score 63)
229 - 242	830.44	1658.87	1658.87	-1	1	K.TIENINELIEESKK.T (Ions score 67)
229 - 242	830.44	1658.87	1658.87	-1	1	K.TIENINELIEESKK.T (Ions score 76)
259 - 271	809.91	1617.80	1617.80	-2	0	K.LYQAQYDLSIYNK.Q (Ions score 73)
259 - 271	809.91	1617.80	1617.80	-1	0	K.LYQAQYDLSIYNK.Q (Ions score 83)
259 - 271	809.91	1617.80	1617.80	0	0	K.LYQAQYDLSIYNK.Q (Ions score 77)
272 - 285	541.63	1621.86	1621.87	-2	0	K.QLEEAHNLISVLEK.R (Ions score 10)
272 - 285	811.94	1621.87	1621.87	-1	0	K.QLEEAHNLISVLEK.R (Ions score 98)
272 - 285	541.63	1621.87	1621.87	-1	0	K.QLEEAHNLISVLEK.R (Ions score 8)
272 - 285	811.94	1621.87	1621.87	-1	0	K.QLEEAHNLISVLEK.R (Ions score 93)
272 - 286	593.66	1777.96	1777.97	-2	1	K.QLEEAHNLISVLEKR.I (Ions score 64)
272 - 286	593.66	1777.97	1777.97	-2	1	K.QLEEAHNLISVLEKR.I (Ions score 61)
272 - 286	593.66	1777.97	1777.97	-1	1	K.QLEEAHNLISVLEKR.I (Ions score 59)
423 - 430	512.74	1023.46	1023.46	0	0	K.NIYTDNER.K (Ions score 40)
433 - 440	510.79	1019.56	1019.57	-4	1	K.FINEIKEK.I (Ions score 22)
433 - 440	510.79	1019.56	1019.57	-3	1	K.FINEIKEK.I (Ions score 17)
433 - 440	510.79	1019.56	1019.57	-2	1	K.FINEIKEK.I (Ions score 15)
461 - 471	670.34	1338.66	1338.67	-1	1	K.SLNDITKEYEK.L (Ions score 51)
472 - 480	547.79	1093.56	1093.57	-3	0	K.LLNEIYDSK.F (Ions score 36)
472 - 480	547.79	1093.56	1093.57	-3	0	K.LLNEIYDSK.F (Ions score 35)
505 - 518	550.26	1647.76	1647.76	-3	0	K.LTHHNTFASYENSK.H (Ions score 5)
530 - 537	538.74	1075.46	1075.46	-3	0	K.YMEDYSLR.N (Ions score 35)
530 - 537	538.74	1075.46	1075.46	-1	0	K.YMEDYSLR.N (Ions score 37)
530 - 537	546.74	1091.46	1091.46	-1	0	K.YMEDYSLR.N + Oxidation (M) (Ions score 31)
530 - 537	546.74	1091.46	1091.46	0	0	K.YMEDYSLR.N + Oxidation (M) (Ions score 46)
538 - 546	536.32	1070.63	1070.63	-3	1	R.NIVVEKELK.Y (Ions score 14)
538 - 546	536.32	1070.63	1070.63	-1	1	R.NIVVEKELK.Y (Ions score 17)
538 - 546	536.32	1070.63	1070.63	-1	1	R.NIVVEKELK.Y (Ions score 21)
589 - 597	508.30	1014.59	1014.60	-3	0	K.ILEVSDIVK.V (Ions score 30)

589 - 597	508.30	1014.60	1014.60	-1	0	K.ILEVSDIVK.V (Ions score 33)
589 - 597	508.31	1014.60	1014.60	-1	0	K.ILEVSDIVK.V (Ions score 26)

Sample name: 6

Protein Information

Protein name:	Glyceraldehyde-3-phosphate dehydrogenase OS=Plasmodium falciparum Vietnam Oak-Knoll (FVO) GN=PFFVO_05270 PE=3 SV=1
Entry name:	A0A024V013_PLAFA
Calculated MW:	37068
Calculated pI:	7.59
Mascot score:	826
Sequence coverage:	43%
Bioinformatic tools:	1: UniProt Entry 2: Conserved Domains in NCBI

Analysis Information

- Enzyme: Trypsin
- Variable modifications: Oxidation (M)
- Fixed modifications: Carbamidomethyl (C)
- Database search program: Mascot version 2.4
- Peptide Tolerance: 10 ppm
- Database: UniprotTREMBL (50011027 protein sequences)

Protein sequence

Matched peptides shown in bold underline

```
  1 MAVTKLGING FGRIGRLVFR AAFGRKDIEV VAINDPFMDL NHLCYLLKYD
 51 SVHGQFPCEV THADGFLLIG EKKVSVFAEK DPSQIPWGKC QVDVVCESTG
101 VFLTKELASS HLKGGAKKVI MSAPPKDDTP IYVMGINHHQ YDTKQLIVSN
151 ASCTTNCLAP LAKVINDRFG IVEGLMTTVH ASTANQLVVD GPSKGGKDWR
201 AGRCALSNII PASTGAAKAV GKVLPELNGK LTGVAFRVPI GTVSVVDLVC
251 RLQKPAKYEE VALEIKKAAE GPLKGILGYT EDEVVSQDFV HDNRSSIFDM
301 KAGLALNDNF FKLVSWYDNE WGYSNRVLDL AVHITNN
```

Peptides used for identification

Peptides shown in bold have been analysed by MS/MS sequencing

Start - End	Observed	Mr(expt)	Mr(calc)	Delta	Miss	Sequence
6 - 13	417.23	832.45	832.46	-2	0	**K.LGINGFGR.I (Ions score 40)**
6 - 13	417.23	832.45	832.46	-2	0	**K.LGINGFGR.I (Ions score 34)**
6 - 13	417.23	832.45	832.46	-1	0	**K.LGINGFGR.I (Ions score 41)**
74 - 89	596.65	1786.92	1786.93	-2	1	**K.VSVFAEKDPSQIPWGK.C (Ions score 50)**
74 - 89	596.65	1786.92	1786.93	-2	1	**K.VSVFAEKDPSQIPWGK.C (Ions score 69)**
90 - 105	921.44	1840.87	1840.87	-1	0	**K.CQVDVVCESTGVFLTK.E (Ions score 64)**
90 - 105	921.44	1840.87	1840.87	0	0	**K.CQVDVVCESTGVFLTK.E (Ions score 79)**
106 - 113	442.75	883.48	883.48	-1	0	**K.ELASSHLK.G (Ions score 21)**
145 - 163	1031.03	2060.04	2060.04	-1	0	**K.QLIVSNASCTTNCLAPLAK.V (Ions score 136)**
145 - 163	1031.03	2060.04	2060.04	-1	0	**K.QLIVSNASCTTNCLAPLAK.V (Ions score 140)**

Alphalyse A/S ■ Unsbjergvej 4 ■ 5220 Odense ■ Denmark
Tel: +45 6310 6500 ■ e-mail info@alphalyse.com
www.alphalyse.com

145 - 163	1031.03	2060.04	2060.04	0	0	K.QLIVSNASCTTNCLAPLAK.V (Ions score 133)
204 - 218	737.39	1472.76	1472.77	-1	0	R.CALSNIIPASTGAAK.A (Ions score 70)
204 - 218	737.39	1472.76	1472.77	-1	0	R.CALSNIIPASTGAAK.A (Ions score 72)
204 - 218	737.39	1472.77	1472.77	0	0	R.CALSNIIPASTGAAK.A (Ions score 75)
258 - 267	611.34	1220.66	1220.67	-4	1	K.YEEVALEIKK.A (Ions score 53)
258 - 267	611.34	1220.66	1220.67	-2	1	K.YEEVALEIKK.A (Ions score 61)
275 - 294	765.03	2292.06	2292.07	0	0	K.GILGYTEDEVVSQDFVHDNR.S (Ions score 156)
275 - 294	1147.04	2292.07	2292.07	0	0	K.GILGYTEDEVVSQDFVHDNR.S (Ions score 145)
275 - 294	765.03	2292.07	2292.07	0	0	K.GILGYTEDEVVSQDFVHDNR.S (Ions score 142)
275 - 294	1147.04	2292.07	2292.07	0	0	K.GILGYTEDEVVSQDFVHDNR.S (Ions score 163)
302 - 312	605.32	1208.62	1208.62	-1	0	K.AGLALNDNFFK.L (Ions score 43)
302 - 312	605.32	1208.62	1208.62	-1	0	K.AGLALNDNFFK.L (Ions score 40)
302 - 312	605.32	1208.62	1208.62	1	0	K.AGLALNDNFFK.L (Ions score 54)
313 - 326	894.90	1787.78	1787.79	-3	0	K.LVSWYDNEWGYSNR.V (Ions score 81)
313 - 326	894.90	1787.79	1787.79	0	0	K.LVSWYDNEWGYSNR.V (Ions score 94)
327 - 337	604.83	1207.65	1207.66	-2	0	R.VLDLAVHITNN.- (Ions score 25)
327 - 337	604.83	1207.65	1207.66	-2	0	R.VLDLAVHITNN.- (Ions score 22)
327 - 337	604.84	1207.66	1207.66	0	0	R.VLDLAVHITNN.- (Ions score 28)

LC-MSMS Protein Identification Report

Order 16372_Plasmodium falciparum

Sample name: 6

Protein Information

Protein name:	**Elongation factor 1-alpha OS=Plasmodium falciparum Vietnam Oak-Knoll (FVO) GN=PFFVO_04465 PE=3 SV=1**
Entry name:	A0A024V296_PLAFA
Calculated MW:	49156
Calculated pI:	9.12
Mascot score:	654
Sequence coverage:	37%
Bioinformatic tools:	1: UniProt Entry 2: Conserved Domains in NCBI

Analysis Information

- Enzyme: Trypsin
- Variable modifications: Oxidation (M)
- Fixed modifications: Carbamidomethyl (C)
- Database search program: Mascot version 2.4
- Peptide Tolerance: 10 ppm
- Database: UniprotTREMBL (50011027 protein sequences)

Protein sequence

Matched peptides shown in bold underline

1 MGKEKTHINL VVIGHVDSGK **STTTGHIIYK** LGGIDRRTIE KFEKESAEMG
51 KGSFKYAWVL DKLKAERERG ITIDIALWKF ETPR**YFFTVI** **DAPGHKDFIK**
101 NMITGTSQAD VALLVVPAEV GGFEGAFSKE GQTKEHALLA FTLGVK**QIVV**
151 **GVNKMDTVK**Y SEDRYEEIKK EVKDYLKK**VG** **YQADKVDFIP** **ISGFEGDNLI**
201 **EK**SDKTPWYK GRTLIEALDT MEPPKRPYDK PLR**IPLQGVY** **KIGGIGTVPV**
251 **GR**VETGILK**A** **GMVLNFAPSA** **VVSECK**SVEM HK**EVLEEARP** **GDNIGFNVK**N
301 VSVKEIKRGY VASDTKNEPA KGCSK**FTAQV** **IILNHPGEIK** NGYTPVLDCH
351 TSHISCKFLN IDSKIDKRSG KVVEENPKAI K**SGDSALVSL** **EPKKPMVVET**
401 **FTEYPPLGR**F AIRDMR**QTIA** **VGIIK**SVEKK EPGAVTAKAP AKK

Peptides used for identification

Peptides shown in bold have been analysed by MS/MS sequencing

Start - End	Observed	Mr(expt)	Mr(calc)	Delta	Miss	Sequence
21 - 30	560.80	1119.59	1119.59	0	0	**K.STTTGHIIYK.L (Ions score 17)**
85 - 100	633.33	1896.97	1896.98	-2	1	**R.YFFTVIDAPGHKDFIK.N (Ions score 70)**
85 - 100	633.33	1896.98	1896.98	0	1	**R.YFFTVIDAPGHKDFIK.N (Ions score 68)**
147 - 159	723.90	1445.79	1445.79	-4	1	**K.QIVVGVNKMDTVK.Y + Oxidation (M) (Ions score 27)**
147 - 159	723.90	1445.79	1445.79	0	1	**K.QIVVGVNKMDTVK.Y + Oxidation (M) (Ions score 22)**
179 - 202	1327.67	2653.32	2653.33	-1	1	**K.VGYQADKVDFIPISGFEGDNLIEK.S (Ions score 13)**
179 - 202	885.45	2653.32	2653.33	-1	1	**K.VGYQADKVDFIPISGFEGDNLIEK.S (Ions score 87)**

Alphalyse A/S ■ Unsbjergvej 4 ■ 5220 Odense ■ Denmark
Tel: +45 6310 6500 ■ e-mail info@alphalyse.com
www.alphalyse.com

179 - 202	885.45	2653.32	2653.33	-1	1	K.VGYQADKVDFIPISGFEGDNLIEK.S (Ions score 93)
179 - 202	885.45	2653.33	2653.33	0	1	K.VGYQADKVDFIPISGFEGDNLIEK.S (Ions score 104)
179 - 202	1327.67	2653.33	2653.33	1	1	K.VGYQADKVDFIPISGFEGDNLIEK.S (Ions score 8)
234 - 241	459.28	916.54	916.54	-3	0	R.IPLQGVYK.I (Ions score 25)
234 - 241	459.28	916.54	916.54	-3	0	R.IPLQGVYK.I (Ions score 14)
242 - 252	513.31	1024.60	1024.60	-3	0	K.IGGIGTVPVGR.V (Ions score 64)
242 - 252	513.31	1024.60	1024.60	-2	0	K.IGGIGTVPVGR.V (Ions score 64)
242 - 252	513.31	1024.60	1024.60	-1	0	K.IGGIGTVPVGR.V (Ions score 64)
260 - 276	898.44	1794.86	1794.86	-2	0	K.AGMVLNFAPSAVVSECK.S + Oxidation (M) (Ions score 88)
260 - 276	898.44	1794.86	1794.86	-1	0	K.AGMVLNFAPSAVVSECK.S + Oxidation (M) (Ions score 95)
283 - 299	629.66	1885.95	1885.95	-2	0	K.EVLEEARPGDNIGFNVK.N (Ions score 34)
283 - 299	629.66	1885.95	1885.95	-1	0	K.EVLEEARPGDNIGFNVK.N (Ions score 17)
283 - 299	629.66	1885.95	1885.95	-1	0	K.EVLEEARPGDNIGFNVK.N (Ions score 6)
326 - 340	560.65	1678.94	1678.94	-2	0	K.FTAQVIILNHPGEIK.N (Ions score 53)
326 - 340	560.65	1678.94	1678.94	-2	0	K.FTAQVIILNHPGEIK.N (Ions score 75)
326 - 340	560.65	1678.94	1678.94	-2	0	K.FTAQVIILNHPGEIK.N (Ions score 70)
326 - 340	840.48	1678.94	1678.94	-1	0	K.FTAQVIILNHPGEIK.N (Ions score 84)
326 - 340	840.48	1678.94	1678.94	-1	0	K.FTAQVIILNHPGEIK.N (Ions score 76)
382 - 393	601.81	1201.62	1201.62	-3	0	K.SGDSALVSLEPK.K (Ions score 49)
382 - 393	601.82	1201.62	1201.62	-2	0	K.SGDSALVSLEPK.K (Ions score 48)
382 - 393	601.82	1201.62	1201.62	-1	0	K.SGDSALVSLEPK.K (Ions score 74)
394 - 409	627.32	1878.95	1878.96	-2	0	K.KPMVVETFTEYPPLGR.F + Oxidation (M) (Ions score 53)
417 - 425	471.80	941.59	941.59	-2	0	R.QTIAVGIIK.S (Ions score 8)
417 - 425	471.80	941.59	941.59	-1	0	R.QTIAVGIIK.S (Ions score 5)
417 - 425	471.80	941.59	941.59	-1	0	R.QTIAVGIIK.S (Ions score 7)

Alphalyse A/S ■ Unsbjergvej 4 ■ 5220 Odense ■ Denmark
Tel: +45 6310 6500 ■ e-mail info@alphalyse.com
www.alphalyse.com

LC-MSMS Protein Identification Report

Order 16372_Plasmodium falciparum

Sample name: 6

Protein Information

**Protein name:**	**Phosphoglycerate kinase OS=Plasmodium falciparum Vietnam Oak-Knoll (FVO) GN=PFFVO_02527 PE=3 SV=1**
Entry name:	A0A024V742_PLAFA
Calculated MW:	45569
Calculated pI:	7.63
Mascot score:	648
Sequence coverage:	34%
Bioinformatic tools:	1: UniProt Entry 2: Conserved Domains in NCBI

Analysis Information

- Enzyme: Trypsin
- Variable modifications: Oxidation (M)
- Fixed modifications: Carbamidomethyl (C)
- Database search program: Mascot version 2.4
- Peptide Tolerance: 10 ppm
- Database: UniprotTREMBL (50011027 protein sequences)

Protein sequence

Matched peptides shown in bold underline

```
  1 MLGNKLSISD LKDIKNKKVL VRVDFNVPIE NGIIKDTNRI TATLPTINHL
 51 KKEGASKIIL ISHCGRPDGL RNEKYTLKPV AETLKGLLGE EVLFLNDCVG
101 KEVEDKINAA KENSVILLEN LRFHIEEEGK GVDANGNKVK ANKEDVEKFQ
151 NDLTKLADVF INDAFGTAHR AHSSMVGVKL NVKASGFLMK KELEYFSKAL
201 ENPQRPLLAI LGGAKVSDKI QLIKNLLDKV DRMIIGGGMA YTFKKVLNNM
251 KIGTSLFDEA GSKIVGEIME KAKAKNVQIF LPVDFKIADN FDNNANTKFV
301 TDEEGIPDNW MGLDAGPKSI ENYKDVILTS KTVIWNGPQG VFEMPNFAKG
351 SIECLNLVVE VTKKGAITIV GGGDTASLVE QQNKKNEISH VSTGGGASLE
401 LLEGKELPGV LALSNK
```

Matched (bold underlined) stretches: LSISD LKDIK (6–15); VDFNVPIE NGIIKDTNRI TATLPTINHL KK (23–52); ENSVILLEN LR (112–122); LADVF INDAFGTAHR (156–170); AL ENPQRPLLAI LGGAK (199–215); NLLDKV DR (225–232); IGTSLFDEA GSK (252–263); NVQIF LPVDFKIADN FDNNANTK (276–298); TVIWNGPQG VFEMPNFAK (332–349).

Peptides used for identification

Peptides shown in bold have been analysed by MS/MS sequencing

Start - End	Observed	Mr(expt)	Mr(calc)	Delta	Miss	Sequence
6 - 15	566.33	1130.65	1130.65	-2	1	**K.LSISDLKDIK.N (Ions score 21)**
23 - 35	729.40	1456.79	1456.79	-3	0	**R.VDFNVPIENGIIK.D (Ions score 28)**
23 - 35	729.40	1456.79	1456.79	-1	0	**R.VDFNVPIENGIIK.D (Ions score 28)**
23 - 39	648.68	1943.01	1943.01	-1	1	**R.VDFNVPIENGIIKDTNR.I (Ions score 93)**
40 - 52	483.96	1448.86	1448.87	-9	1	**R.ITATLPTINHLKK.E (Ions score 17)**
112 - 122	650.37	1298.72	1298.72	-2	0	**K.ENSVILLENLR.F (Ions score 51)**
112 - 122	650.37	1298.72	1298.72	0	0	**K.ENSVILLENLR.F (Ions score 47)**

Alphalyse A/S ■ Unsbjergvej 4 ■ 5220 Odense ■ Denmark
Tel: +45 6310 6500 ■ e-mail info@alphalyse.com
www.alphalyse.com

156 - 170	549.61	1645.82	1645.82	-3	0	K.LADVFINDAFGTAHR.A (Ions score 64)
156 - 170	549.61	1645.82	1645.82	-1	0	K.LADVFINDAFGTAHR.A (Ions score 72)
199 - 215	587.68	1760.03	1760.03	-2	0	K.ALENPQRPLLAILGGAK.V (Ions score 72)
199 - 215	587.68	1760.03	1760.03	-1	0	K.ALENPQRPLLAILGGAK.V (Ions score 42)
225 - 232	486.78	971.54	971.54	-4	1	K.NLLDKVDR.M (Ions score 37)
252 - 263	612.81	1223.60	1223.60	-1	0	K.IGTSLFDEAGSK.I (Ions score 68)
252 - 263	612.81	1223.60	1223.60	0	0	K.IGTSLFDEAGSK.I (Ions score 68)
276 - 286	660.37	1318.73	1318.73	-2	0	K.NVQIFLPVDFK.I (Ions score 40)
276 - 286	660.37	1318.73	1318.73	-1	0	K.NVQIFLPVDFK.I (Ions score 56)
276 - 286	660.37	1318.73	1318.73	-1	0	K.NVQIFLPVDFK.I (Ions score 52)
287 - 298	668.81	1335.60	1335.61	-1	0	K.IADNFDNNANTK.F (Ions score 38)
332 - 349	1018.01	2034.00	2034.00	-1	0	K.TVIWNGPQGVFEMPNFAK.G (Ions score 84)
332 - 349	1018.01	2034.00	2034.00	-1	0	K.TVIWNGPQGVFEMPNFAK.G (Ions score 62)
332 - 349	1026.01	2050.00	2050.00	-1	0	K.TVIWNGPQGVFEMPNFAK.G + Oxidation (M) (Ions score 93)

Alphalyse A/S ■ Unsbjergvej 4 ■ 5220 Odense ■ Denmark
Tel: +45 6310 6500 ■ e-mail info@alphalyse.com
www.alphalyse.com

Sample name: 6

Protein Information

**Protein name:**	**Enolase OS=Plasmodium falciparum Vietnam Oak-Knoll (FVO) GN=PFFVO_02844 PE=3 SV=1**
Entry name:	A0A024V5N5_PLAFA
Calculated MW:	48989
Calculated pI:	6.21
Mascot score:	633
Sequence coverage:	24%
Bioinformatic tools:	1: UniProt Entry 2: Conserved Domains in NCBI

Analysis Information

- Enzyme: Trypsin
- Variable modifications: Oxidation (M)
- Fixed modifications: Carbamidomethyl (C)
- Database search program: Mascot version 2.4
- Peptide Tolerance: 10 ppm
- Database: UniprotTREMBL (50011027 protein sequences)

Protein sequence

Matched peptides shown in bold underline

```
  1 MAHVITRINA REILDSRGNP TVEVDLETNL GIFRAAVPSG ASTGIYEALE
 51 LRDNDKSRYL GKGVQKAIKN INEIIAPKLI GMNCTEQKKI DNLMVEELDG
101 SKNEWGWSKS KLGANAILAI SMAVCRAGAA ANKVSLYKYL AQLAGKKSDQ
151 MVLPVPCLNV INGGSHAGNK LSFQEFMIVP VGAPSFKEAL RYGAEVYHTL
201 KSEIKKKYGI DATNVGDEGG FAPNILNANE ALDLLVTAIK SAGYEGKVKI
251 AMDVAASEFY NSENKTYDLD FKTPNNDKSL VKTGAQLVDL YIDLVKKYPI
301 VSIEDPFDQD DWENYAKLTA AIGKDVQIVG DDLLVTNPTR ITKALEKNAC
351 NALLLKVNQI GSITEAIEAC LLSQKNNWGV MVSHRSGETE DVFIADLVVA
401 LRTGQIKTGA PCRSERNAKY NQLLRIEESL GNNAVFAGEK FRLQLN
```

Peptides used for identification

Peptides shown in bold have been analysed by MS/MS sequencing

Start - End	Observed	Mr(expt)	Mr(calc)	Delta	Miss	Sequence
171 - 187	949.00	1895.98	1895.99	-3	0	**K.LSFQEFMIVPVGAPSFK.E (Ions score 44)**
171 - 187	949.00	1895.98	1895.99	-1	0	**K.LSFQEFMIVPVGAPSFK.E (Ions score 53)**
171 - 187	949.00	1895.98	1895.99	-1	0	**K.LSFQEFMIVPVGAPSFK.E (Ions score 86)**
171 - 187	957.00	1911.98	1911.98	-2	0	**K.LSFQEFMIVPVGAPSFK.E + Oxidation (M) (Ions score 88)**
171 - 187	957.00	1911.98	1911.98	-1	0	**K.LSFQEFMIVPVGAPSFK.E + Oxidation (M) (Ions score 96)**
171 - 187	957.00	1911.98	1911.98	0	0	**K.LSFQEFMIVPVGAPSFK.E + Oxidation (M) (Ions score 79)**
283 - 296	774.44	1546.86	1546.86	-3	0	**K.TGAQLVDLYIDLVK.K (Ions score 48)**

Alphalyse A/S ■ Unsbjergvej 4 ■ 5220 Odense ■ Denmark
Tel: +45 6310 6500 ■ e-mail info@alphalyse.com
www.alphalyse.com

283 - 296	774.44	1546.86	1546.86	0	0	K.TGAQLVDLYIDLVK.K (Ions score 49)
283 - 296	774.44	1546.86	1546.86	0	0	K.TGAQLVDLYIDLVK.K (Ions score 48)
297 - 317	858.07	2571.18	2571.18	-1	1	K.KYPIVSIEDPFDQDDWENYAK.L (Ions score 104)
297 - 317	858.07	2571.18	2571.18	-1	1	K.KYPIVSIEDPFDQDDWENYAK.L (Ions score 92)
297 - 317	858.07	2571.18	2571.18	0	1	K.KYPIVSIEDPFDQDDWENYAK.L (Ions score 87)
318 - 340	803.78	2408.33	2408.33	-1	1	K.LTAAIGKDVQIVGDDLLVTNPTR.I (Ions score 125)
318 - 340	803.78	2408.33	2408.33	0	1	K.LTAAIGKDVQIVGDDLLVTNPTR.I (Ions score 106)
318 - 340	803.78	2408.33	2408.33	1	1	K.LTAAIGKDVQIVGDDLLVTNPTR.I (Ions score 126)
325 - 340	877.97	1753.92	1753.92	-2	0	K.DVQIVGDDLLVTNPTR.I (Ions score 95)
325 - 340	877.97	1753.92	1753.92	-1	0	K.DVQIVGDDLLVTNPTR.I (Ions score 98)
357 - 375	1037.55	2073.08	2073.08	-1	0	K.VNQIGSITEAIEACLLSQK.N (Ions score 71)
357 - 375	1037.55	2073.08	2073.08	0	0	K.VNQIGSITEAIEACLLSQK.N (Ions score 44)
357 - 375	1037.55	2073.08	2073.08	1	0	K.VNQIGSITEAIEACLLSQK.N (Ions score 64)
426 - 440	789.39	1576.77	1576.77	1	0	R.IEESLGNNAVFAGEK.F (Ions score 90)
426 - 440	789.39	1576.77	1576.77	1	0	R.IEESLGNNAVFAGEK.F (Ions score 85)

Sample name: 6

Protein Information

**Protein name:**	**Hsp70-like protein OS=Plasmodium falciparum Vietnam Oak-Knoll (FVO) GN=PFFVO_02131 PE=3 SV=1**
Entry name:	A0A024V7B6_PLAFA
Calculated MW:	74724
Calculated pI:	5.51
Mascot score:	471
Sequence coverage:	13%
Bioinformatic tools:	1: UniProt Entry 2: Conserved Domains in NCBI

Analysis Information

- Enzyme: Trypsin
- Variable modifications: Oxidation (M)
- Fixed modifications: Carbamidomethyl (C)
- Database search program: Mascot version 2.4
- Peptide Tolerance: 10 ppm
- Database: UniprotTREMBL (50011027 protein sequences)

Protein sequence

Matched peptides shown in bold underline

```
  1 MASAKGSKPN LPESNIAIGI DLGTTYSCVG VWRNENVDII ANDQGNRTTP
 51 SYVAFTDTER LIGDAAKNQV ARNPENTVFD AKRLIGRKFT ESSVQSDMKH
101 WPFTVKSGVD EKPMIEVTYQ GEKKLFHPEE ISSMVLQKMK ENAEAFLGKS
151 IKNAVITVPA YFNDSQRQAT KDAGTIAGLN VMRIINEPTA AAIAYGLHKK
201 GKGEKNILIF DLGGGTFDVS LLTIEDGIFE VKATAGDTHL GGEDFDNRLV
251 NFCVEDFKRK NRGKDLSKNS RALRRLRTQC ERAKRTLSSS TQATIEIDSL
301 FEGIDYSVTV SRARFEELCI DYFRDTLIPV EKVLKDAMMD KKSVHEVVLV
351 GGSTRIPKIQ TLIKEFFNGK EACRSINPDE AVAYGAAVQA AILSGDQSNA
401 VQDLLLLDVC SLSLGLETAG GVMTKLIERN TTIPAKKSQI FTTYADNQPG
451 VLIQVYEGER ALTKDNNLLG KFHLDGIPPA PRKVPQIEVT FDIDANGILN
501 VTAVEKSTGK QNHITITNDK GRLSQDEIDR MVNDAEKYKA EDEENRKRIE
551 ARNSLENYCY GVKSSLEDQK IKEKLQPAEI ETCMKTITTI LEWLEKNQLA
601 GKDEYEAKQK EAESVCAPIM SKIYQDAAGA AGGMPGGMPG GMPGGMPGGM
651 PGGMNFPGGM PGAGMPGNAP AGSGPTVEEV D
```

Matched peptides (bold underline in the original): NENVDII ANDQGNRTTP SYVAFTDTER (34–60); NPENTVFD AKR (73–83); NAVITVPA YFNDSQR (153–167); DAGTIAGLN VMRIINEPTA AAIAYGLHK (172–199); SVHEVVLV GGSTR (343–355).

Peptides used for identification

Peptides shown in bold have been analysed by MS/MS sequencing

Start - End Observed Mr(expt) Mr(calc) Delta Miss Sequence

Alphalyse A/S ■ Unsbjergvej 4 ■ 5220 Odense ■ Denmark
Tel: +45 6310 6500 ■ e-mail info@alphalyse.com
www.alphalyse.com

34 - 47	786.37	1570.73	1570.73	0	0	R.NENVDIIANDQGNR.T (Ions score 65)
34 - 47	786.37	1570.73	1570.73	0	0	R.NENVDIIANDQGNR.T (Ions score 83)
48 - 60	744.35	1486.69	1486.69	-1	0	R.TTPSYVAFTDTER.L (Ions score 69)
73 - 83	645.83	1289.64	1289.64	0	1	R.NPENTVFDAKR.L (Ions score 58)
153 - 167	847.93	1693.84	1693.84	-1	0	K.NAVITVPAYFNDSQR.Q (Ions score 82)
153 - 167	847.93	1693.84	1693.84	1	0	K.NAVITVPAYFNDSQR.Q (Ions score 72)
172 - 183	617.32	1232.62	1232.62	-2	0	K.DAGTIAGLNVMR.I + Oxidation (M) (Ions score 52)
172 - 183	617.32	1232.62	1232.62	-1	0	K.DAGTIAGLNVMR.I + Oxidation (M) (Ions score 58)
184 - 199	561.31	1680.92	1680.92	-2	0	R.IINEPTAAAIAYGLHK.K (Ions score 67)
343 - 355	670.37	1338.72	1338.73	-2	0	K.SVHEVVLVGGSTR.I (Ions score 43)
343 - 355	670.37	1338.72	1338.73	-1	0	K.SVHEVVLVGGSTR.I (Ions score 54)

Sample name: 6

Protein Information

**Protein name:**	**Hsp90-like protein OS=Plasmodium falciparum Vietnam Oak-Knoll (FVO) GN=PFFVO_01664 PE=3 SV=1**
Entry name:	A0A024V8X4_PLAFA
Calculated MW:	86512
Calculated pI:	4.94
Mascot score:	468
Sequence coverage:	12%
Bioinformatic tools:	1: UniProt Entry 2: Conserved Domains in NCBI

Analysis Information

- Enzyme: Trypsin
- Variable modifications: Oxidation (M)
- Fixed modifications: Carbamidomethyl (C)
- Database search program: Mascot version 2.4
- Peptide Tolerance: 10 ppm
- Database: UniprotTREMBL (50011027 protein sequences)

Protein sequence

Matched peptides shown in bold underline

```
  1 MSTETFAFNA DIRQLMSLII NTFYSNKEIF LRELISNASD ALDKIRYESI
 51 TDTQKLSAEP EFFIRIIPDK TNNTLTIEDS GIGMTKNDLI NNLGTIARSG
101 TKAFMEAIQA SGDISMIGQF GVGFYSAYLV ADHVVVISKN NDDEQYVWES
151 AAGGSFTVTK DETNEKLGRG TKIILHLKED QLEYLEEKRI KDLVKKHSEF
201 ISFPIKLYCE RQNEKEITAS EEEEGEGEGE REGEEEEEKK KKTGEDKNAD
251 ESKEENEDEE KKEDNEEDDN KTDHPKVEDV TEELENAEKK KKEKRKKKIH
301 TVEHEWEELN KQKPLWMRKP EEVTNEEYAS FYKSLTNDWE DHLAVKHFSV
351 EGQLEFKALL FIPKRAPFDM FENRKKRNNI KLYVRRVFIM DDCEEIIPEW
401 LNFVKGVVDS EDLPLNISRE SLQQNKILKV IKKNLIKKCL DMFSELAENK
451 ENYKKFYEQF SKNLKLGIHE DNANRTKITE LLRFQTSKSG DEMIGLKEYV
501 DRMKENQKDI YYITGESINA VSNSPFLEAL TKKGFEVIYM VDPIDEYAVQ
551 QLKDFDGKKL KCCTKEGLDI DDSEEAKKDF ETLKAEYEGL CKVIKDVLHE
601 KVEKVVVGQR ITDSPCVLVT SEFGWSANME RIMKAQALRD NSMTSYMLSK
651 KIMEINARHP IISALKQKAD ADKSDKTVKD LIWLLFDTSL LTSGFALEEP
701 TTFSKRIHRM IKLGLSIDEE ENNDIDLPPL EETVDATDSK MEEVD
```

Matched (bold underline): LSAEP EFFIR (56–65); NDLI NNLGTIAR (87–98); HSEF ISFPIK (197–206); KP EEVTNEEYAS FYKSLTNDWE DHLAVK (319–346); GVVDS EDLPLNISR (406–419); KGFEVIYM VDPIDEYAVQ QLK (533–553).

Peptides used for identification

Peptides shown in bold have been analysed by MS/MS sequencing

Alphalyse A/S ■ Unsbjergvej 4 ■ 5220 Odense ■ Denmark
Tel: +45 6310 6500 ■ e-mail info@alphalyse.com
www.alphalyse.com

Start - End	Observed	Mr(expt)	Mr(calc)	Delta	Miss	Sequence
56 - 65	604.82	1207.62	1207.62	-1	0	K.LSAEPEFFIR.I (Ions score 54)
56 - 65	604.82	1207.62	1207.62	-1	0	K.LSAEPEFFIR.I (Ions score 65)
56 - 65	604.82	1207.62	1207.62	-1	0	K.LSAEPEFFIR.I (Ions score 64)
87 - 98	657.36	1312.71	1312.71	-2	0	K.NDLINNLGTIAR.S (Ions score 37)
197 - 206	602.82	1203.62	1203.63	-4	0	K.HSEFISFPIK.L (Ions score 39)
197 - 206	602.82	1203.63	1203.63	-3	0	K.HSEFISFPIK.L (Ions score 27)
319 - 333	917.43	1832.84	1832.85	-2	0	R.KPEEVTNEEYASFYK.S (Ions score 103)
319 - 333	917.43	1832.85	1832.85	2	0	R.KPEEVTNEEYASFYK.S (Ions score 109)
334 - 346	764.37	1526.73	1526.74	-3	0	K.SLTNDWEDHLAVK.H (Ions score 54)
406 - 419	757.39	1512.77	1512.78	-3	0	K.GVVDSEDLPLNISR.E (Ions score 85)
406 - 419	757.40	1512.78	1512.78	-2	0	K.GVVDSEDLPLNISR.E (Ions score 79)
406 - 419	757.40	1512.78	1512.78	-1	0	K.GVVDSEDLPLNISR.E (Ions score 92)
533 - 553	834.42	2500.25	2500.26	-2	1	K.KGFEVIYMVDPIDEYAVQQLK.D + Oxidation (M) (Ions score 72)
533 - 553	834.42	2500.25	2500.26	-2	1	K.KGFEVIYMVDPIDEYAVQQLK.D + Oxidation (M) (Ions score 70)
533 - 553	834.43	2500.26	2500.26	1	1	K.KGFEVIYMVDPIDEYAVQQLK.D + Oxidation (M) (Ions score 65)

Sample name: 6

Protein Information

**Protein name:**	**Uncharacterized protein (Fragment) OS=Plasmodium falciparum Vietnam Oak-Knoll (FVO) GN=PFFVO_03207 PE=4 SV=1**
Entry name:	A0A024V5X8_PLAFA
Calculated MW:	68379
Calculated pI:	6.63
Mascot score:	432
Sequence coverage:	13%
Bioinformatic tools:	1: UniProt Entry 2: Conserved Domains in NCBI

Analysis Information

- Enzyme: Trypsin
- Variable modifications: Oxidation (M)
- Fixed modifications: Carbamidomethyl (C)
- Database search program: Mascot version 2.4
- Peptide Tolerance: 10 ppm
- Database: UniprotTREMBL (50011027 protein sequences)

Protein sequence

Matched peptides shown in bold underline

1 ENATTSTNEQ HSHDQNTEVH PNDK**LALVPF QGIK**NPIPSN ESQPIISFPN
51 EDDNHAQNEG SINAPSEGEH NNTDNKEGPI ITPLEGEQAG TAHKEDVTHK
101 HMVGEHVPPQ KTHHGPIITP VGGNHVPPQT HHAHIITPVG GEHAHGQGNN
151 DTTYVTMNTD ESSSSDTKGE HSNLRSYNKN MNNNHAQRDQ YDSDTLNSEG
201 SDDAYSSMQQ NFEKNGIDSF KGKGLHVSLR ERIIIEIMES AKNGIDGLLK
251 LKDSKDSGKL FMEALEKLNI NMKDLKKDKN LISLEVYDKI LSTMFKILTE
301 MSFYEDSKFY ETLGIKK**DIL NQSLKDIK**IK MLRKLGVSYS RLPPIIKHTE
351 GK**CAIKDIII SISSK**ELAQR MAIMFTKWLA PDEYGAVVDY ENNVELNVLC
401 SGAPILIQQW KYYQNMLGFE EDKDHAYLGL IDELLVMNKR **YSQNKDYVET**
451 **LEK**IKKSKVF KHCTKIMRIG GK**VSSVPFNY ENVKKPSSSI IGSLGNLIK**A
501 NISTYYKATA QRINSYFHYT EKKSKKSSPL KIISVCTLLH LTDMLYKCSD
551 ENSNGVMDLY NLQLNTLNMK GK**MVLQYLVH LK**FLTQEKKN QLKEICEPQN
601 GLI

Peptides used for identification

Peptides shown in bold have been analysed by MS/MS sequencing

Start - End	Observed	Mr(expt)	Mr(calc)	Delta	Miss	Sequence
25 - 34	543.34	1084.66	1084.66	-2	0	**K.LALVPFQGIK.N (Ions score 28)**
318 - 328	643.87	1285.72	1285.72	-4	1	**K.DILNQSLKDIK.I (Ions score 36)**

Alphalyse A/S ■ Unsbjergvej 4 ■ 5220 Odense ■ Denmark
Tel: +45 6310 6500 ■ e-mail info@alphalyse.com
www.alphalyse.com

318 - 328	643.87	1285.72	1285.72	-2	1	K.DILNQSLKDIK.I (Ions score 40)
353 - 365	724.41	1446.81	1446.81	-2	1	K.CAIKDIIISISSK.E (Ions score 88)
353 - 365	724.41	1446.81	1446.81	-1	1	K.CAIKDIIISISSK.E (Ions score 88)
441 - 453	808.89	1615.77	1615.77	-2	1	R.YSQNKDYVETLEK.I (Ions score 61)
441 - 453	539.60	1615.77	1615.77	-2	1	R.YSQNKDYVETLEK.I (Ions score 20)
441 - 453	808.89	1615.77	1615.77	0	1	R.YSQNKDYVETLEK.I (Ions score 62)
441 - 453	539.60	1615.77	1615.77	1	1	R.YSQNKDYVETLEK.I (Ions score 19)
473 - 484	691.85	1381.69	1381.69	-1	0	K.VSSVPFNYENVK.K (Ions score 73)
473 - 484	691.85	1381.69	1381.69	-1	0	K.VSSVPFNYENVK.K (Ions score 54)
473 - 484	691.85	1381.69	1381.69	1	0	K.VSSVPFNYENVK.K (Ions score 58)
485 - 499	757.45	1512.88	1512.89	-2	0	K.KPSSSIIGSLGNLIK.A (Ions score 98)
485 - 499	757.45	1512.89	1512.89	-1	0	K.KPSSSIIGSLGNLIK.A (Ions score 98)
485 - 499	757.45	1512.89	1512.89	-1	0	K.KPSSSIIGSLGNLIK.A (Ions score 107)
573 - 582	630.36	1258.71	1258.71	-2	0	K.MVLQYLVHLK.F + Oxidation (M) (Ions score 29)
573 - 582	630.36	1258.71	1258.71	-1	0	K.MVLQYLVHLK.F + Oxidation (M) (Ions score 33)

Sample name: 6

Protein Information

Protein name:	**S-adenosylmethionine synthase OS=Plasmodium falciparum Vietnam Oak-Knoll (FVO) GN=PFFVO_02524 PE=3 SV=1**
Entry name:	A0A024V6I4_PLAFA
Calculated MW:	45272
Calculated pI:	6.28
Mascot score:	379
Sequence coverage:	15%
Bioinformatic tools:	1: UniProt Entry 2: Conserved Domains in NCBI

Analysis Information

- Enzyme: Trypsin
- Variable modifications: Oxidation (M)
- Fixed modifications: Carbamidomethyl (C)
- Database search program: Mascot version 2.4
- Peptide Tolerance: 10 ppm
- Database: UniprotTREMBL (50011027 protein sequences)

Protein sequence

Matched peptides shown in bold underline

1 MSQLKIKRGN FLFTSESVNE GHPDKICDQI SDAILDSCLR EDPYSKVACE
51 VCAKKNYIFI FGEITTKAKV NYDKVTRDVL KHIGYDDESK GLDYKTAEIK
101 VSIDEQSPDI AQCVHENRSP ELIGAGDQGI MFGYATDETE NYMPLTHHYA
151 TLLGKRLTEV RK**LGILPYLG** **PDGK**TQITIE YKNKGSCGGH LEPLRVHTVL
201 ISTQHAEDIK YEQLKTDLME NVIK**YVIPEK** **LLDNETLYYL** **NPSGKFVLGG**
251 **PAADAGLTGR** KIICDTYGGW GAHGGGAFSG KDASKVDRSA AYYLRFIAKS
301 LVANKFCRRV LVQASYSIGI ANPISLNVNS YGTVSTGYTD YDLEQIILR**N**
351 **FDLRPGFIIQ** **ELK**LTEPVFS KTSAYGHFGR EGDTFTWEKI KDLSHEKNAL
401 KN

Peptides used for identification

Peptides shown in bold have been analysed by MS/MS sequencing

Start - End	Observed	Mr(expt)	Mr(calc)	Delta	Miss	Sequence
163 - 174	621.86	1241.70	1241.70	-1	0	**K.LGILPYLGPDGK.T** (Ions score 56)
163 - 174	621.86	1241.70	1241.70	-1	0	**K.LGILPYLGPDGK.T** (Ions score 56)
225 - 245	823.77	2468.28	2468.28	-1	1	**K.YVIPEKLLDNETLYYLNPSGK.F** (Ions score 51)
225 - 245	823.77	2468.29	2468.28	1	1	**K.YVIPEKLLDNETLYYLNPSGK.F** (Ions score 60)
231 - 245	870.45	1738.88	1738.88	-1	0	**K.LLDNETLYYLNPSGK.F** (Ions score 96)
246 - 260	701.38	1400.74	1400.74	-2	0	**K.FVLGGPAADAGLTGR.K** (Ions score 116)
350 - 363	845.47	1688.92	1688.93	-4	0	**R.NFDLRPGFIIQELK.L** (Ions score 51)

Alphalyse A/S ■ Unsbjergvej 4 ■ 5220 Odense ■ Denmark
Tel: +45 6310 6500 ■ e-mail info@alphalyse.com
www.alphalyse.com

350 - 363	563.98	1688.92	1688.93	-4	0	R.NFDLRPGFIIQELK.L (Ions score 10)
350 - 363	845.47	1688.92	1688.93	-3	0	R.NFDLRPGFIIQELK.L (Ions score 31)
350 - 363	563.98	1688.92	1688.93	-1	0	R.NFDLRPGFIIQELK.L (Ions score 11)
350 - 363	563.98	1688.92	1688.93	-1	0	R.NFDLRPGFIIQELK.L (Ions score 8)
350 - 363	845.47	1688.92	1688.93	0	0	R.NFDLRPGFIIQELK.L (Ions score 42)

Sample name: 6

Protein Information

Protein name:	Uncharacterized protein OS=Plasmodium falciparum Vietnam Oak-Knoll (FVO) GN=PFFVO_03482 PE=3 SV=1
Entry name:	A0A024V542_PLAFA
Calculated MW:	95301
Calculated pI:	5.28
Mascot score:	365
Sequence coverage:	9%
Bioinformatic tools:	1: UniProt Entry 2: Conserved Domains in NCBI

Analysis Information

- Enzyme: Trypsin
- Variable modifications: Oxidation (M)
- Fixed modifications: Carbamidomethyl (C)
- Database search program: Mascot version 2.4
- Peptide Tolerance: 10 ppm
- Database: UniprotTREMBL (50011027 protein sequences)

Protein sequence

Matched peptides shown in bold underline

1 MKLNNIYSFF FLFFVLCVIQ ENVRRVLCDS SVEGDKGPSD DVSDSSGEKK
51 EVKRDRDTLE EIEEGEKPTE SMESHQYQTE VTRLMDIIVN SLYTQKEVFL
101 RELISNAADA LEKIRFLSLS DESVLGEEKK LEIRISANKE KNILSITDTG
151 IGMTK**VDLIN NLGTIAK**SGT SNFLEAISKS GGDMSLIGQF GVGFYSAFLV
201 ADKVIVYTKN NDDEQYIWES TADAKFTIYK DPRGATLKRG TRISLHLKED
251 ATNLLNDKKL MDLISKYSQF IQFPIYLLHE NVYTEEVLAD IAKDMVNDPN
301 YDSVKVEETD DPNKKTRTVE KKVKKWTLMN EQRPIWLRSP KELKDEDYKQ
351 FFSVLSGYND QPLYHIHFFA EGEIEFKCLI YIPSKAPSMN DQLYSKQNSL
401 KLYVRR**VLVA DEFVEFLPR**Y MSFVK**GVVDS DDLPLNVSR**E QLQQNKILKA
451 VSKRIVRKIL DTFHKLYKEG KKNKETLRSE LENETDEEKK KEITKKLSEP
501 STYKLIYKEY RKFLKSGCYE DDINRNKIAK LLLFKTMQYP KSISLDTYIE
551 HMKPDQKFIY YASGDSYEYL AKIPQLQIFK KKNIDVLFLT ESVDESCIQR
601 VQEYEGKKFK SIQKGEISFE LTEEEKKKEQ QMQKMYK**ALI DVISDTLKNQ**
651 **IFK**VEISRRL VDAPCAVVST EWGLSGQMEK LMKMNVSNSD QIKAMSGQKI
701 LEINPNHPIM IDLLKRSVTN PKDLELTNSI KIMYQSAK**LA SGFDLEDTAD**
751 **LAQIVYDHIN QK**LGVDNNLK IDDLDPSIFE TKKIEDENDS SKFEEEINID
801 DEIQKKDNNV DNESNDKSDE L

Alphalyse A/S ■ Unsbjergvej 4 ■ 5220 Odense ■ Denmark
Tel: +45 6310 6500 ■ e-mail info@alphalyse.com
www.alphalyse.com

Peptides used for identification

Peptides shown in bold have been analysed by MS/MS sequencing

Start - End	Observed	Mr(expt)	Mr(calc)	Delta	Miss	Sequence
156 - 167	635.87	1269.73	1269.73	-1	0	K.**VDLINNLGTIAK**.S (Ions score 54)
407 - 419	767.42	1532.82	1532.82	-2	0	R.**VLVADEFVEFLPR**.Y (Ions score 93)
407 - 419	767.42	1532.82	1532.82	0	0	R.**VLVADEFVEFLPR**.Y (Ions score 79)
426 - 439	743.38	1484.75	1484.75	-1	0	K.**GVVDSDDLPLNVSR**.E (Ions score 83)
638 - 648	594.35	1186.68	1186.68	-3	0	K.**ALIDVISDTLK**.N (Ions score 63)
638 - 648	594.35	1186.68	1186.68	-2	0	K.**ALIDVISDTLK**.N (Ions score 50)
638 - 653	909.52	1817.03	1817.03	-1	1	K.**ALIDVISDTLKNQIFK**.V (Ions score 24)
638 - 653	909.52	1817.03	1817.03	0	1	K.**ALIDVISDTLKNQIFK**.V (Ions score 26)
638 - 653	606.68	1817.03	1817.03	0	1	K.**ALIDVISDTLKNQIFK**.V (Ions score 47)
638 - 653	606.68	1817.03	1817.03	2	1	K.**ALIDVISDTLKNQIFK**.V (Ions score 36)
739 - 762	892.77	2675.30	2675.31	-3	0	K.**LASGFDLEDTADLAQIVYDHINQK**.L (Ions score 15)
739 - 762	892.78	2675.32	2675.31	3	0	K.**LASGFDLEDTADLAQIVYDHINQK**.L (Ions score 26)

Alphalyse A/S ■ Unsbjergvej 4 ■ 5220 Odense ■ Denmark
Tel: +45 6310 6500 ■ e-mail info@alphalyse.com
www.alphalyse.com

Sample name: 6

Protein Information

**Protein name:**	**Diphosphate-fructose-6-phosphate 1-phosphotransferase OS=Plasmodium falciparum Vietnam Oak-Knoll (FVO) GN=PFFVO_02450 PE=3 SV=1**
Entry name:	A0A024V6T3_PLAFA
Calculated MW:	160663
Calculated pI:	6.32
Mascot score:	360
Sequence coverage:	8%
Bioinformatic tools:	1: UniProt Entry 2: Conserved Domains in NCBI

Analysis Information

- Enzyme: Trypsin
- Variable modifications: Oxidation (M)
- Fixed modifications: Carbamidomethyl (C)
- Database search program: Mascot version 2.4
- Peptide Tolerance: 10 ppm
- Database: UniprotTREMBL (50011027 protein sequences)

Protein sequence

Matched peptides shown in bold underline

1 MDTKSGDKNA ANKGGADGLV KTVSVLLRDN KCQFNIDENY DHNDKEKLEC
51 EVGKRDSGMI NCLMEKLTSK KFLEEKESKN SFYLVDNENM KIKKLKEHGH
101 SASLNDDLSP LQYERTKYIP SLPKALASEY QILDENYGDE FINKNDYEDV
151 KRFLKNLHNL PMLNVKDSNN NESFKGGNIL KIGIILSGGP APGGHNVISG
201 IYDYAKRYNE QSQVIGFLGG IDGLYSKNYV TITDSMMNRF RNLGGFNMLW
251 SGRGKVKNKD DLIAIENIVA KLKLNGLVII GGDGSNSNAA LMAEYFAERQ
301 IPISIIGVPK TIDGDLKSEA IEISFGFDTA TRTYSEIIGN LCTDVKTGHN
351 VYHVVRVMGR SASHVVLECA LQTRPNVVLI GEEVEQLNLS LKDIVKNIVN
401 IILKRKSLNK **NYGVILLPEG** **LIEFVPEMK**I LISELNVILK DGPFDASKLQ
451 KSKEVWDFLP PIIRDQLLMD RESTGYIQVG KIATERLIIV LVESELAKLN
501 DKNLNIQFMS HYLGYEGRCA IPSNFDCNYC YALGYNAALL IDHKKTGYMS
551 IIR**NLEDSYT** **NWIPAAIPFL** **R**IMHVIKDNT GNEFPAVKR**Y** **LVDLNSPLFN**
601 **VLK**EVR**SLWS** **LYDLYR**SPGP IQFNGHLGNA RCYTVKTPTK DNLLCQNADD
651 LELIINLTNK NMYENNGDNN HNISDDKARD GGSSPTSSAK KTKYNLSDDN
701 NNNNNNINNV STNYNNTSDG STFGNTTLLN TAYNVNGDGM NTLNCQKSNT
751 SDVLSSEPVN QGFYEQHASS YKSLGCMSEL QTSRLYNKLE LPELCSDLKA
801 KVRAGKQYIS NDPYTQKQIL SNYPHMSYEN KFQIQEIFHD KYASPISFEI
851 RIGIVFLSRQ APGAMNVLCG LYRRLKLLKG VCIAFYGLYG LLHNKYIIID
901 DDNIAKYVNQ GGLELTGNSP EHSLFDKENR NKVCETVTKL QLNGLVMPGS

951 NITITEAALL SEYFLEKKIP TSVVGIPLTG SNNLIHELIE TCVGFDSSTK
1001 **VYASLIGNVL** **TDAVSMPK**YW HFIRLMGRSP SHEVLECALQ THPNVVIISE
1051 EYGAADKTLW RVVQDIADVV CARADVGKNY GTVLIPDALL MHLPHMK**ILL**
1101 **SEISDILNEA** **SEK**GQLLEAR NDLVNLSGVD HGHLTSEWVS K**LTPWSLALL**
1151 **K**TFPQFIIKE LLQVDLRSMR FEQLETEQLL LQMVKEELQD RKQKGKYSGS
1201 FMGLTHFFGY QGRSSLPSEF DCKLAYAYGH AASIVIESGL TGYIVSIRGL
1251 CGNVKDWK**LF** **AIPFISLMK**I LPKGQGSKYL KSASKGDLPV IPSAPVDLNG
1301 KAYRSLKIAL QKWQMEDRFC NPGPIQFEGN ASNYYNRILF EEQSEYFEML
1351 RYVECYANIL KDTCRFGVSA DYLKNVFVQL CGMLVLAYKP NDILSNMPYI
1401 GSIEDYYDWE NQRKRMN

Peptides used for identification

Peptides shown in bold have been analysed by MS/MS sequencing

Start - End	Observed	Mr(expt)	Mr(calc)	Delta	Miss	Sequence
411 - 429	1089.08	2176.15	2176.15	-1	0	**K.NYGVILLPEGLIEFVPEMK.I** + Oxidation (M) (Ions score 35)
411 - 429	1089.08	2176.15	2176.15	-1	0	**K.NYGVILLPEGLIEFVPEMK.I** + Oxidation (M) (Ions score 37)
411 - 429	1089.08	2176.15	2176.15	0	0	**K.NYGVILLPEGLIEFVPEMK.I** + Oxidation (M) (Ions score 34)
554 - 571	1060.54	2119.07	2119.07	-2	0	**R.NLEDSYTNWIPAAIPFLR.I** (Ions score 35)
554 - 571	1060.54	2119.07	2119.07	0	0	**R.NLEDSYTNWIPAAIPFLR.I** (Ions score 41)
554 - 571	1060.55	2119.08	2119.07	1	0	**R.NLEDSYTNWIPAAIPFLR.I** (Ions score 31)
590 - 603	817.96	1633.91	1633.91	-2	0	**R.YLVDLNSPLFNVLK.E** (Ions score 32)
590 - 603	817.96	1633.91	1633.91	0	0	**R.YLVDLNSPLFNVLK.E** (Ions score 49)
590 - 603	817.96	1633.91	1633.91	1	0	**R.YLVDLNSPLFNVLK.E** (Ions score 61)
607 - 616	658.34	1314.66	1314.66	-1	0	**R.SLWSLYDLYR.S** (Ions score 57)
1001 - 1018	947.50	1892.99	1892.99	-2	0	**K.VYASLIGNVLTDAVSMPK.Y** + Oxidation (M) (Ions score 62)
1001 - 1018	947.50	1892.99	1892.99	-1	0	**K.VYASLIGNVLTDAVSMPK.Y** + Oxidation (M) (Ions score 80)
1098 - 1113	887.48	1772.94	1772.94	1	0	**K.ILLSEISDILNEASEK.G** (Ions score 34)
1142 - 1151	571.35	1140.69	1140.69	-2	0	**K.LTPWSLALLK.T** (Ions score 19)
1259 - 1269	648.37	1294.73	1294.74	-4	0	**K.LFAIPFISLMK.I** + Oxidation (M) (Ions score 28)
1259 - 1269	648.37	1294.73	1294.74	-1	0	**K.LFAIPFISLMK.I** + Oxidation (M) (Ions score 24)
1259 - 1269	648.38	1294.74	1294.74	0	0	**K.LFAIPFISLMK.I** + Oxidation (M) (Ions score 31)

Alphalyse A/S ■ Unsbjergvej 4 ■ 5220 Odense ■ Denmark
Tel: +45 6310 6500 ■ e-mail info@alphalyse.com
www.alphalyse.com

LC-MSMS Protein Identification Report

Order 16372_Plasmodium falciparum

Sample name: 6

Protein Information

**Protein name:**	**Ornithine aminotransferase OS=Plasmodium falciparum Vietnam Oak-Knoll (FVO) GN=PFFVO_06225 PE=3 SV=1**
Entry name:	A0A024UX71_PLAFA
Calculated MW:	46938
Calculated pI:	6.47
Mascot score:	349
Sequence coverage:	14%
Bioinformatic tools:	1: UniProt Entry 2: Conserved Domains in NCBI

Analysis Information

- Enzyme: Trypsin
- Variable modifications: Oxidation (M)
- Fixed modifications: Carbamidomethyl (C)
- Database search program: Mascot version 2.4
- Peptide Tolerance: 10 ppm
- Database: UniprotTREMBL (50011027 protein sequences)

Protein sequence

Matched peptides shown in bold underline

```
  1 MDFVKELKSS QDYMNNELTY GAHNYDPIPV VLKRGKGVFV YDIEDRRYYD
 51 FLSAYSSVNQ GHCHPDILNA MINQAKKLTI CSRAFFSDSL GVCERYLTNL
101 FGYDKVLMMN TGAEASETAY KLCRKWGYEV KKIPENSAKI IVCNNNFSGR
151 TLGCVSASTD KKCKNNFGPF VPNFLKVPYD DLEALEKELQ DPNVCAFIVE
201 PVQGEAGVIV PSDSYFPGVA SLCKKYNVLF VADEVQTGLG RTGKLLCTHH
251 YGVKPDVILL GKALSGGHYP ISAILANDDV MLVLKPGEHG STYGGNPLAA
301 AICVEALKVL INEKLCENAD KLGAPFLQNL KEQLKDSKVV REVRGKGLLC
351 AIEFKNDLVN VWDICLKFKE NGLITRSVHD KTVRLTPPLC ITKEQLDECT
401 EIIVKTVKFF DDNL
```

Peptides used for identification

Peptides shown in bold have been analysed by MS/MS sequencing

Start - End	Observed	Mr(expt)	Mr(calc)	Delta	Miss	Sequence
84 - 95	694.32	1386.62	1386.62	1	0	**R.AFFSDSLGVCER.Y** (Ions score 65)
96 - 105	617.31	1232.60	1232.61	-3	0	**R.YLTNLFGYDK.V** (Ions score 44)
96 - 105	617.31	1232.61	1232.61	-1	0	**R.YLTNLFGYDK.V** (Ions score 58)
140 - 150	647.32	1292.63	1292.63	-1	0	**K.IIVCNNNFSGR.T** (Ions score 64)
140 - 150	647.32	1292.63	1292.63	-1	0	**K.IIVCNNNFSGR.T** (Ions score 42)
165 - 176	697.37	1392.72	1392.72	-2	0	**K.NNFGPFVPNFLK.V** (Ions score 67)
165 - 176	697.37	1392.72	1392.72	-1	0	**K.NNFGPFVPNFLK.V** (Ions score 18)

Alphalyse A/S ■ Unsbjergvej 4 ■ 5220 Odense ■ Denmark
Tel: +45 6310 6500 ■ e-mail info@alphalyse.com
www.alphalyse.com

226 - 241	890.96	1779.91	1779.92	-1	0	K.YNVLFVADEVQTGLGR.T (Ions score 95)
226 - 241	890.96	1779.92	1779.92	0	0	K.YNVLFVADEVQTGLGR.T (Ions score 93)

LC-MSMS Protein Identification Report

Order 16372_Plasmodium falciparum

Sample name: 6

Protein Information

Protein name:	**Hypoxanthine-guanine-xanthine phosphoribosyltransferase OS=Plasmodium falciparum Vietnam Oak-Knoll (FVO) GN=PFFVO_02805 PE=4 SV=1**
Entry name:	A0A024V748_PLAFA
Calculated MW:	24632
Calculated pI:	9.05
Mascot score:	328
Sequence coverage:	44%
Bioinformatic tools:	1: UniProt Entry 2: Conserved Domains in NCBI

Analysis Information

- Enzyme: Trypsin
- Variable modifications: Oxidation (M)
- Fixed modifications: Carbamidomethyl (C)
- Database search program: Mascot version 2.4
- Peptide Tolerance: 10 ppm
- Database: UniprotTREMBL (50011027 protein sequences)

Protein sequence

Matched peptides shown in bold underline

1 MCIKQYTYKN ISKLGHKYLT K**VLVPNGVIK** NRIEKLAYDI KK**VYNNEEFH**
51 **ILCLLK**GSR**G** **FFTALLK**HLS R**IHNYSAVET** **SKPLFGEHYV** **R**VK**SYCNDQS**
101 **TGTLEIVSED** **LSCLK**GK**HVL** **IVEDIIDTGK** TLVKFCEYLK KFEIKTVAIA
151 CLFIKR**TPLW** **NGFK**ADFVGF SIPDHFVVGY SLDYNEIFRD LDHCCLVNDE
201 GKKKYKATSL

Peptides used for identification

Peptides shown in bold have been analysed by MS/MS sequencing

Start - End	Observed	Mr(expt)	Mr(calc)	Delta	Miss	Sequence
22 - 30	469.80	937.59	937.60	-3	0	**K.VLVPNGVIK.N (Ions score 23)**
43 - 56	597.97	1790.90	1790.90	-1	0	**K.VYNNEEFHILCLLK.G (Ions score 44)**
60 - 67	448.76	895.51	895.52	-2	0	**R.GFFTALLK.H (Ions score 21)**
60 - 67	448.77	895.52	895.52	-1	0	**R.GFFTALLK.H (Ions score 16)**
72 - 91	587.55	2346.17	2346.18	-4	0	**R.IHNYSAVETSKPLFGEHYVR.V (Ions score 21)**
72 - 91	587.55	2346.17	2346.18	-2	0	**R.IHNYSAVETSKPLFGEHYVR.V (Ions score 25)**
94 - 115	1260.07	2518.12	2518.12	1	0	**K.SYCNDQSTGTLEIVSEDLSCLK.G (Ions score 104)**
118 - 130	726.41	1450.80	1450.80	-1	0	**K.HVLIVEDIIDTGK.T (Ions score 73)**
118 - 130	726.41	1450.80	1450.80	0	0	**K.HVLIVEDIIDTGK.T (Ions score 77)**
157 - 164	481.76	961.50	961.50	-4	0	**R.TPLWNGFK.A (Ions score 34)**
157 - 164	481.76	961.50	961.50	-3	0	**R.TPLWNGFK.A (Ions score 27)**

Alphalyse A/S ■ Unsbjergvej 4 ■ 5220 Odense ■ Denmark
Tel: +45 6310 6500 ■ e-mail info@alphalyse.com
www.alphalyse.com

LC-MSMS Protein Identification Report

Order 16372_Plasmodium falciparum

Sample name: 6

Protein Information

Protein name:	L-lactate dehydrogenase OS=Plasmodium falciparum Vietnam Oak-Knoll (FVO) GN=PFFVO_04104 PE=3 SV=1
Entry name:	A0A024V3C8_PLAFA
Calculated MW:	34314
Calculated pI:	7.12
Mascot score:	322
Sequence coverage:	24%
Bioinformatic tools:	1: UniProt Entry 2: Conserved Domains in NCBI

Analysis Information

- Enzyme: Trypsin
- Variable modifications: Oxidation (M)
- Fixed modifications: Carbamidomethyl (C)
- Database search program: Mascot version 2.4
- Peptide Tolerance: 10 ppm
- Database: UniprotTREMBL (50011027 protein sequences)

Protein sequence

Matched peptides shown in bold underline

```
  1 MAPKAKIVLV GSGMIGGVMA TLIVQKNLGD VVLFDIVKNM PHGKALDTSH
 51 TNVMAYSNCK VSGSNTYDDL AGADVVIVTA GFTKAPGKSD KEWNRDDLLP
101 LNNKIMIEIG GHIKKNCPNA FIIVVTNPVD VMVQLLHQHS GVPKNKIIGL
151 GGVLDTSRLK YYISQKLNVC PRDVNAHIVG AHGNKMVLLK RYITVGGIPL
201 QEFINNKLIS DAELEAIFDR TVNTALEIVN LHASPYVAPA AAIIEMAESY
251 LKDLKKVLIC STLLEGQYGH SDIFGGTPVV LGANGVEQVI ELQLNSEEKA
301 KFDEAIAETK RMKALA
```

Peptides used for identification

Peptides shown in bold have been analysed by MS/MS sequencing

Start - End	Observed	Mr(expt)	Mr(calc)	Delta	Miss	Sequence
27 - 38	666.38	1330.75	1330.75	-2	0	**K.NLGDVVLFDIVK.N** (Ions score 34)
27 - 38	666.38	1330.75	1330.75	-1	0	**K.NLGDVVLFDIVK.N** (Ions score 34)
27 - 38	666.38	1330.75	1330.75	-1	0	**K.NLGDVVLFDIVK.N** (Ions score 38)
61 - 84	1200.60	2399.18	2399.19	-3	0	**K.VSGSNTYDDLAGADVVIVTAGFTK.A** (Ions score 82)
61 - 84	1200.60	2399.19	2399.19	1	0	**K.VSGSNTYDDLAGADVVIVTAGFTK.A** (Ions score 95)
61 - 84	1200.60	2399.19	2399.19	1	0	**K.VSGSNTYDDLAGADVVIVTAGFTK.A** (Ions score 77)
147 - 158	600.85	1199.68	1199.69	-3	0	**K.IIGLGGVLDTSR.L** (Ions score 90)
147 - 158	600.85	1199.69	1199.69	-1	0	**K.IIGLGGVLDTSR.L** (Ions score 90)
147 - 158	600.85	1199.69	1199.69	-1	0	**K.IIGLGGVLDTSR.L** (Ions score 89)
173 - 185	666.34	1330.67	1330.67	-1	0	**R.DVNAHIVGAHGNK.M** (Ions score 12)

Alphalyse A/S ■ Unsbjergvej 4 ■ 5220 Odense ■ Denmark
Tel: +45 6310 6500 ■ e-mail info@alphalyse.com
www.alphalyse.com

192 - 207	903.49	1804.97	1804.97	-2	0	R.YITVGGIPLQEFINNK.L (Ions score 83)
192 - 207	903.49	1804.97	1804.97	-1	0	R.YITVGGIPLQEFINNK.L (Ions score 87)
192 - 207	903.49	1804.97	1804.97	-1	0	R.YITVGGIPLQEFINNK.L (Ions score 85)

LC-MSMS Protein Identification Report

Order 16372_Plasmodium falciparum

Sample name: 6

Protein Information

Protein name:	Uncharacterized protein OS=Plasmodium falciparum UGT5.1 GN=C923_03132 PE=4 SV=1
Entry name:	W7JXD3_PLAFA
Calculated MW:	124344
Calculated pI:	4.28
Mascot score:	254
Sequence coverage:	6%
Bioinformatic tools:	1: UniProt Entry 2: Conserved Domains in NCBI

Analysis Information

- Enzyme: Trypsin
- Variable modifications: Oxidation (M)
- Fixed modifications: Carbamidomethyl (C)
- Database search program: Mascot version 2.4
- Peptide Tolerance: 10 ppm
- Database: UniprotTREMBL (50011027 protein sequences)

Protein sequence

Matched peptides shown in bold underline

1 MRNLFHITIC LVTLNLFILE INAKTNTSEN RNKRIGGPKL RGNVTSNIKF
51 PSDNKGKIIR GSNDQLNKNS EDVLEQSEKS LVSENVPSGL DIDDIPKESI
101 FIQEDQEGQT HSELNPETSE HSKDLNNNGS KNESSDIISE NNKSNKVQNH
151 FESLSDLELL ENSSQDNLDK DTISTEPFPN QKHKDLQQDL NDEPLEPFPT
201 QIHKDYKEKN LINEEDSEPF PRQKHKKVDN HNEEKNVFHE NGSANGNQGS
251 LKLKSFDEHL KDEKIENEPL VHENLSIPND PIEQILNQPE QETNIQEQLY
301 NEKQNVEEKQ NSQIPSLDLK EPTNEDILPN HNPLENIKQS ESEINHVQDH
351 ALPKENIIDK LDNQKEHIDQ SQHNINVLQE NNINNHQLEP QEKPNIESFE
401 PK**NIDSEIIL** **PENVETEEII** **DDVPSPK**HSN HETFEEETSE SEHEEAVSEK
451 NAHETVEHEE TVSQESNPEK ADNDGNVSQN SNNELNENEF VESEKSEHEP
501 AENEESSLEE GHHEEIVPEQ NNEESGESKL VDNDEGGFEE AHHENFSSEV
551 SNSELNENEF VESDKSVTEP AEHEEVVSEE SNPEPAENEE SSIEEGHQEE
601 IVPEQNDEES GESGLVDNEE GDFEEPNHEE FEPDQNDSEL SENELVESEK
651 SVSEPAEHVE IVSEK**SASEP** **AEHVEIVSEK** SVSEPAEHVE SVSEQSNNEP
701 SEKKDGPVPS KPFEEIEKVD VQPK**IVDLQI** **IEPNFVDSQP** **NPQEPVEPSF**
751 **VKIEK**VPSEE NKHASVDPEV KEKENVSEVV EEKQNSQESV EEIPVNEDEF
801 EDVHTEQLDL DHKTVDPEIV EVEEIPSELH ENEVAHPEIV EIEEVFPEPN
851 QNNEFQEINE DDKSAHIQHE IVEVEEILPE DDKNEKVEEI LPEEDKNEKG
901 QHEIVEVEEI LPEIVEIEEV PSQTNNNENI ETIKPEEKKN EFSVEEKAIP

Alphalyse A/S ■ Unsbjergvej 4 ■ 5220 Odense ■ Denmark
Tel: +45 6310 6500 ■ e-mail info@alphalyse.com
www.alphalyse.com

951 QEPVVPTLNE NENVPPKPSE GESTKPDIVQ IKIVQENKPN KKETPVVDGP
1001 KHVEQNIQED DNDEEDDDDI DFEGLSRKDD EKDSSNKNKK KSSFITYIST
1051 KKFKKVSQTI VSVMINAYDG VIQVVSTIKG IAKDIVIFFQ NI

Peptides used for identification

Peptides shown in bold have been analysed by MS/MS sequencing

Start - End	Observed	Mr(expt)	Mr(calc)	Delta	Miss	Sequence
403 - 427	936.80	2807.39	2807.40	-1	0	K.**NIDSEIILPENVETEEIIDDVPSPK**.H (Ions score 87)
403 - 427	936.81	2807.40	2807.40	0	0	K.**NIDSEIILPENVETEEIIDDVPSPK**.H (Ions score 73)
403 - 427	936.81	2807.40	2807.40	2	0	K.**NIDSEIILPENVETEEIIDDVPSPK**.H (Ions score 76)
666 - 680	806.40	1610.78	1610.78	0	0	K.**SASEPAEHVEIVSEK**.S (Ions score 42)
666 - 680	806.40	1610.78	1610.78	2	0	K.**SASEPAEHVEIVSEK**.S (Ions score 37)
725 - 752	1060.21	3177.62	3177.62	0	0	K.**IVDLQIIEPNFVDSQPNPQEPVEPSFVK**.I (Ions score 68)
725 - 752	1060.22	3177.62	3177.62	0	0	K.**IVDLQIIEPNFVDSQPNPQEPVEPSFVK**.I (Ions score 85)
725 - 752	1060.22	3177.63	3177.62	1	0	K.**IVDLQIIEPNFVDSQPNPQEPVEPSFVK**.I (Ions score 85)
725 - 755	1183.62	3547.85	3547.85	0	1	K.**IVDLQIIEPNFVDSQPNPQEPVEPSFVKIEK**.V (Ions score 39)

Alphalyse A/S ■ Unsbjergvej 4 ■ 5220 Odense ■ Denmark
Tel: +45 6310 6500 ■ e-mail info@alphalyse.com
www.alphalyse.com

Sample name: 6

Protein Information

**Protein name:**	**Uncharacterized protein OS=Plasmodium falciparum Vietnam Oak-Knoll (FVO) GN=PFFVO_01453 PE=3 SV=1**
Entry name:	A0A024VAR9_PLAFA
Calculated MW:	93184
Calculated pI:	4.95
Mascot score:	193
Sequence coverage:	7%
Bioinformatic tools:	1: UniProt Entry 2: Conserved Domains in NCBI

Analysis Information

- Enzyme: Trypsin
- Variable modifications: Oxidation (M)
- Fixed modifications: Carbamidomethyl (C)
- Database search program: Mascot version 2.4
- Peptide Tolerance: 10 ppm
- Database: UniprotTREMBL (50011027 protein sequences)

Protein sequence

Matched peptides shown in bold underline

1 MEDNTDKKAL VDENNGENKV PKKKNLSRLI VEEATNDDNS VVALNTKRME
51 ELNFFRGDTI IIKGKKRHST ICIILNDNDL DEGKIRINKV ARKNLRVCLG
101 DVVYVKSCPE IPYGKKIQVL PIDDTIEGLA KDTLFEIFLK PYFNESYRPV
151 KKGDLFLVRG GFMSVEFKVV EVDPDDFCIV SPDTVIYYEG DPIKRDDEEK
201 LDEIGYDDIG GCKKQLAQIR EMIELPLRHP GLFKTLGVKP PRGVLLYGPP
251 GSGKTCIARA VANETGAFFF LINGPEVMSK MAGEAEANLR R**AFEEAEKNS**
301 **PAIIFIDEID** **SIAPK**REKTN GEVERRVVSQ LLTLMDGIKS RGQVVVIAAT
351 NRQNSIDPAL RRFGRFDREI DIGVPDDNGR FEILRIHTKN MKLSPDVKLE
401 ELASNTHGFV GADLAQLCTE AALTCIREKM DVIDLEDEII DKEVLESMCV
451 TQDHFNMALG TCNPSSLRET VVEVPNVKWD DIGGLDEVKS TLREMILYPI
501 DHPDKFEKFG MSPSRGVLFY GPPGCGKTLL AKAVASECSA NFVSIKGPEL
551 LTMWFGESEA NVREVFDKAR **AAAPCVLFFD** **ELDSIGTQR**G SSLGDGSGAG
601 DRVMNQLLTE IDGVGPKKNL FFIGATNRPE LLDEALLRPG R**LDQLIYIPL**
651 **PDLGAR**ISIL TAILRKCPVA ENVPIDFLAQ KTAGFSGADL AELCQRAARA
701 AIRDAIDAEE MNKKSKLELS NKKENEQNET NENDVHNKTE QQANDQQKND
751 DDNIKYEITR HHFKEGLAGA RRSVSQADLI KYDNFRIKFD PLYKTKTGGT
801 GDDFIIDWPD EDNNDDTPAY VVDEDLYS

Alphalyse A/S ■ Unsbjergvej 4 ■ 5220 Odense ■ Denmark
Tel: +45 6310 6500 ■ e-mail info@alphalyse.com
www.alphalyse.com

Peptides used for identification

Peptides shown in bold have been analysed by MS/MS sequencing

Start - End	Observed	Mr(expt)	Mr(calc)	Delta	Miss	Sequence
292 - 315	883.12	2646.34	2646.34	-1	1	R.**AFEEAEKNSPAIIFIDEIDSIAPK**.R (Ions score 56)
571 - 589	1055.52	2109.02	2109.02	-1	0	R.**AAAPCVLFFDELDSIGTQR**.G (Ions score 93)
571 - 589	1055.52	2109.02	2109.02	-1	0	R.**AAAPCVLFFDELDSIGTQR**.G (Ions score 54)
571 - 589	704.01	2109.02	2109.02	-1	0	R.**AAAPCVLFFDELDSIGTQR**.G (Ions score 31)
571 - 589	704.01	2109.02	2109.02	0	0	R.**AAAPCVLFFDELDSIGTQR**.G (Ions score 60)
571 - 589	1055.52	2109.02	2109.02	1	0	R.**AAAPCVLFFDELDSIGTQR**.G (Ions score 58)
571 - 589	704.02	2109.02	2109.02	1	0	R.**AAAPCVLFFDELDSIGTQR**.G (Ions score 50)
642 - 656	848.99	1695.96	1695.96	0	0	R.**LDQLIYIPLPDLGAR**.I (Ions score 44)

Alphalyse A/S ■ Unsbjergvej 4 ■ 5220 Odense ■ Denmark
Tel: +45 6310 6500 ■ e-mail info@alphalyse.com
www.alphalyse.com

Sample name: 6

Protein Information

**Protein name:**	**M1 family aminopeptidase OS=Plasmodium falciparum Vietnam Oak-Knoll (FVO) GN=PFFVO_03958 PE=4 SV=1**
Entry name:	A0A024V3R7_PLAFA
Calculated MW:	126553
Calculated pI:	7.3
Mascot score:	179
Sequence coverage:	4%
Bioinformatic tools:	1: UniProt Entry 2: Conserved Domains in NCBI

Analysis Information

- Enzyme: Trypsin
- Variable modifications: Oxidation (M)
- Fixed modifications: Carbamidomethyl (C)
- Database search program: Mascot version 2.4
- Peptide Tolerance: 10 ppm
- Database: UniprotTREMBL (50011027 protein sequences)

Protein sequence

Matched peptides shown in bold underline

```
  1 MKLTKGCAYK YIIFTVLILA NILYDNKKRC MIKKNLRISS CGIISRLLKS
 51 NSNYNSFNKN YNFTSAISEL QFSNFWNLDI LQKDIFSNIH NNKNKPQSYI
101 IHKRLMSEKG DNNNNNHQNN NGNDNKKRLG SVVNNEENTC SDKRMKPFEE
151 GHGITQVDKM NNNSDHLQQN GVMNLNSNNV ENNNNNNSVV VKKNEPKIHY
201 RKDYKPSGFI INNVTLNINI HDNETIVRSV LDMDISKHNV GEDLVFDGVG
251 LKINEISINN KKLVEGEEYT YDNEFLTIFS KFVPKSKFAF SSEVIIHPET
301 NYALTGLYKS KNIIVSQCEA TGFRRITFFI DRPDMMAKYD VTVTADKEKY
351 PVLLSNGDKV NEFEIPGGRH GARFNDPHLK PCYLFAVVAG DLKHLSATYI
401 TKYTKKKVEL YVFSEEKYVS KLQWALECLK KSMAFDEDYF GLEYDLSRLN
451 LVAVSDFNVG AMENKGLNIF NANSLLASKK NSIDFSYARI LTVVGHEYFH
501 NYTGNRVTLR DWFQLTLKEG LTVHRENLFS EEMTKTVTTR LSHVDLLRSV
551 QFLEDSSPLS HPIRPESYVS MENFYTTTVY DKGSEVMRMY LTILGEEYYK
601 KGFDIYIKKN DGNTATCEDF NYAMEQAYKM KKADNSANLN QYLLWFSQSG
651 TPHVSFKYNY DAEKKQYSIH VNQYTKPDEN QKEKKPLFIP ISVGLINPEN
701 GKEMISQTTL ELTKESDTFV FNNIAVKPIP SLFRGFSAPV YIEDNLTDEE
751 RILLLKYDSD AFVRYNSCTN IYMKQILMNY NEFLKAKNEK LESFNLTPVN
801 AQFIDAIKYL LEDPHADAGF KSYIVSLPQD RYIINFVSNL DTDVLADTKE
851 YIYKQIGDKL NDVYYKMFKS LEAKADDLTY FNDESHVDFD QMNMRTLRNT
901 LLSLLSKAQY PNILNEIIEH SKSPYPSNWL TSLSVSAYFD KYFELYDKTY
```

Matched peptides (bold underline): GLNIF NANSLLASK (466–479); ESDTFV FNNIAVKPIP SLFR (715–734); LESFNLTPVN AQFIDAIK (791–808)

Alphalyse A/S ■ Unsbjergvej 4 ■ 5220 Odense ■ Denmark
Tel: +45 6310 6500 ■ e-mail info@alphalyse.com
www.alphalyse.com

951 KLSKDDELLL QEWLKTVSRS DRKDIYEILK KLENEVLKDS KNPNDIRAVY
1001 LPFTNNLRRF HDISGKGYKL IAEVITKTDK FNPMVATQLC EPFKLWNKLD
1051 TKRQELMLNE MNTMLQEPNI SNNLKEYLLR LTNKL

Peptides used for identification

Peptides shown in bold have been analysed by MS/MS sequencing

Start - End	Observed	Mr(expt)	Mr(calc)	Delta	Miss	Sequence
466 - 479	731.40	1460.79	1460.80	-3	0	K.**GLNIFNANSLLASK**.K (Ions score 26)
466 - 479	731.41	1460.80	1460.80	-2	0	K.**GLNIFNANSLLASK**.K (Ions score 48)
715 - 734	765.41	2293.21	2293.21	0	0	K.**ESDTFVFNNIAVKPIPSLFR**.G (Ions score 93)
715 - 734	765.41	2293.21	2293.21	1	0	K.**ESDTFVFNNIAVKPIPSLFR**.G (Ions score 106)
715 - 734	765.41	2293.22	2293.21	3	0	K.**ESDTFVFNNIAVKPIPSLFR**.G (Ions score 104)
791 - 808	1010.54	2019.07	2019.07	-1	0	K.**LESFNLTPVNAQFIDAIK**.Y (Ions score 26)
791 - 808	1010.54	2019.07	2019.07	0	0	K.**LESFNLTPVNAQFIDAIK**.Y (Ions score 13)
791 - 808	1010.54	2019.07	2019.07	1	0	K.**LESFNLTPVNAQFIDAIK**.Y (Ions score 22)

Alphalyse A/S ■ Unsbjergvej 4 ■ 5220 Odense ■ Denmark
Tel: +45 6310 6500 ■ e-mail info@alphalyse.com
www.alphalyse.com

LC-MSMS Protein Identification Report

Order 16372_Plasmodium falciparum

Sample name: 6

Protein Information

Protein name:	Uncharacterized protein OS=Plasmodium falciparum Vietnam Oak-Knoll (FVO) GN=PFFVO_01035 PE=4 SV=1
Entry name:	A0A024VCE7_PLAFA
Calculated MW:	27525
Calculated pI:	6.07
Mascot score:	167
Sequence coverage:	17%
Bioinformatic tools:	1: UniProt Entry 2: Conserved Domains in NCBI

Analysis Information

- Enzyme: Trypsin
- Variable modifications: Oxidation (M)
- Fixed modifications: Carbamidomethyl (C)
- Database search program: Mascot version 2.4
- Peptide Tolerance: 10 ppm
- Database: UniprotTREMBL (50011027 protein sequences)

Protein sequence

Matched peptides shown in bold underline

```
  1 MDNLLRHLKI SKEQITPVVL VVGDPGRVDK IKVVCDSYVD LAYNREYKSV
 51 ECHYKGQKFL CVSHGVGSAG CAVCFEELCQ NGAKVIIRAG SCGSLQPDLI
101 KRGDICICNA AVREDRVSHL LIHGDFPAVG DFDVYDTLNK CAQELNVPVF
151 NGISVSSDMY YPNKIIPSRL EDYSKANAAV VEMELATLMV IGTLRKVKTG
201 GILIVDGCPF KWDEGDFDNN LVPHQLENMI KIALGACAKL ATKYA
```

Peptides used for identification

Peptides shown in bold have been analysed by MS/MS sequencing

Start - End	Observed	Mr(expt)	Mr(calc)	Delta	Miss	Sequence
13 - 30	641.03	1920.06	1920.07	-3	1	K.**EQITPVVLVVGDPGRVDK**.I (Ions score 69)
141 - 164	916.76	2747.26	2747.26	0	0	K.**CAQELNVPVFNGISVSSDMYYPNK**.I + Oxidation (M) (Ions score 72)
141 - 164	1374.64	2747.26	2747.26	1	0	K.**CAQELNVPVFNGISVSSDMYYPNK**.I + Oxidation (M) (Ions score 31)
141 - 164	916.76	2747.26	2747.26	1	0	K.**CAQELNVPVFNGISVSSDMYYPNK**.I + Oxidation (M) (Ions score 97)

Alphalyse A/S ■ Unsbjergvej 4 ■ 5220 Odense ■ Denmark
Tel: +45 6310 6500 ■ e-mail info@alphalyse.com
www.alphalyse.com

LC-MSMS Protein Identification Report

Order 16372_Plasmodium falciparum

Sample name: 6

Protein Information

**Protein name:**	**Elongation factor 2 OS=Plasmodium falciparum Vietnam Oak-Knoll (FVO) GN=PFFVO_05133 PE=4 SV=1**
Entry name:	A0A024UZJ8_PLAFA
Calculated MW:	94546
Calculated pI:	6.36
Mascot score:	160
Sequence coverage:	3%
Bioinformatic tools:	1: UniProt Entry 2: Conserved Domains in NCBI

Analysis Information

- Enzyme: Trypsin
- Variable modifications: Oxidation (M)
- Fixed modifications: Carbamidomethyl (C)
- Database search program: Mascot version 2.4
- Peptide Tolerance: 10 ppm
- Database: UniprotTREMBL (50011027 protein sequences)

Protein sequence

Matched peptides shown in bold underline

1 MVNFTVDQVR EIMNKTKQIR NMSVIAHVDH GKSTLTDSLV SKAGIISSKN
51 AGDARFTDTR QDEQERCITI KSTGISMYFE HDLEDGEGKK PFLINLIDSP
101 GHVDFSSEVT AALRVTDGAL VVVDTIEGVC VQTETVLYQA LGERIKPVLH
151 VNKVDRALLE LQMEVEDIYQ TFARTIESVN VIISTYTDKL MGDIQVYPEK
201 GTVSFGSGLQ GWAFTLETFS RIYSKKFGIE KKKMMQRLWG NSFYDAKTKK
251 WSKNQQEGYK RGFCQFIMEP ILNLCQSIMN DDKEKYTKML TNIGVELKGD
301 DKLLTGKQLL KKAMQLWLPA GDTLLEMIVT HLPSPADAQK YRVENLYEGP
351 MDDEAANAIR NCDPNGPLMM YISKMVPTSD KGRFYAFGRV FSGTVATGQK
401 VRIQGPHYVP GEKTDLYEKN IQRTVLMMGR YTEQVQDVPC GNTCCLVGVD
451 QYIVKSGTIT TFKEAHNIAD MKYSVSPVVR VAVKPKDSKQ LPKLVDGLKK
501 LAKSDPLVLC TTDESGEHII SGCGELHIEI CLKDLKDEYA QIDFIVSDPV
551 VSYR**<u>ETVTEE STITCLGK</u>**SP NKHNRLFMKA YPLAEGLPEA IDKNKVSDKD
601 DPKTRANYLH SNFQWDKNLA LKIWAFGPET IGPNLLTDNT SGIQYMNEIK
651 VHCVAAFQWA SKEGVLCEEN MRGIEFRMLD VHMHADAIHR GAGQIMPACK
701 KCIYACELTA FPRLVEPIYL VDISCPQDVV SGVYGVLNKR RGIVISEEQK
751 LGTPLLK**<u>IQS HLPVSESFGF TSALR</u>**AATSG QAFPQCVFDH WSVLYDDPFD
801 SNKNSYKIIM NIRERKGIKV EMPQLDQYLD KL

Alphalyse A/S ■ Unsbjergvej 4 ■ 5220 Odense ■ Denmark
Tel: +45 6310 6500 ■ e-mail info@alphalyse.com
www.alphalyse.com

Peptides used for identification

Peptides shown in bold have been analysed by MS/MS sequencing

Start - End	Observed	Mr(expt)	Mr(calc)	Delta	Miss	Sequence
555 - 568	784.38	1566.74	1566.74	-1	0	**R.ETVTEESTITCLGK.S (Ions score 84)**
555 - 568	784.38	1566.75	1566.74	0	0	**R.ETVTEESTITCLGK.S (Ions score 71)**
758 - 775	659.35	1975.01	1975.02	-1	0	**K.IQSHLPVSESFGFTSALR.A (Ions score 76)**

Alphalyse A/S ■ Unsbjergvej 4 ■ 5220 Odense ■ Denmark
Tel: +45 6310 6500 ■ e-mail info@alphalyse.com
www.alphalyse.com

Sample name: 6

Protein Information

**Protein name:**	**Uncharacterized protein OS=Plasmodium falciparum Vietnam Oak-Knoll (FVO) GN=PFFVO_05312 PE=4 SV=1**
Entry name:	A0A024UY79_PLAFA
Calculated MW:	164287
Calculated pI:	4.74
Mascot score:	154
Sequence coverage:	2%
Bioinformatic tools:	1: UniProt Entry 2: Conserved Domains in NCBI

Analysis Information

- Enzyme: Trypsin
- Variable modifications: Oxidation (M)
- Fixed modifications: Carbamidomethyl (C)
- Database search program: Mascot version 2.4
- Peptide Tolerance: 10 ppm
- Database: UniprotTREMBL (50011027 protein sequences)

Protein sequence

Matched peptides shown in bold underline

1 MDRIDKEHRR KSNVELDFSD NVSDDDINID KENIWDTDIN IIDDDLNIID
51 NDDVLIQNNS NNKNTDYQND KIFNFQPKQS EWFDEEEIFD LPNNLEDITS
101 KNNSLENNEI NKYDYQNEYN KYNFVDQQNE CDQQNICDQQ NIYDQQNICD
151 QQNICDQQII SEEQNISEEQ NISEEQNIFE EQNISEEQNI CDQKEDIKIH
201 VDNKNVHEFC DTDNLFNNFS TNKPDEQNNE NVPMCLISKS SYSEVNDEFN
251 INNEEHHNKH MNMLLQIKK**I** **LNINEDEDVI** **KYIEK**LNNMN TNHIENVGDD
301 YYGDKNFNEK DSLLNEENEH KVNELKEREL YYLGMIEELR NEIKTKEENE
351 GNNIEKLENK IHEYEKQNEE LRNEKEKLQS TINEYSHNFN NLNDHNKITN
401 KECEELKNNY NTIKEKYERL KEEQEIYIKQ EEEYKSLLDE LENENNEWKE
451 KNQKMHEENM KTCEELKKFE EIRNVEDVKK IEELENKIND LLNINDDYKV
501 KLNQSNDVLL QFEKKLNNSL NDNKKNEHIY SNKIQTLENT IMLLEEEMNC
551 IKKENQEIIN NYNELENDYN LLLNTNKQLN EEYGNLKICK EKNEQNIQHL
601 KSNIQINEQE YSLNSKKLHE QNLKIENLII ENNKNSKMLE LLKNEKLKIE
651 SENDILKKDL TNMEEDLKKL EKHSYDIYEI KNHLEEVVDK NKKLIEDIEL
701 EKNEKEHLNN QLKAYNIEMN NSYLVLKTYK MKCSFFLNII KNYEENIHIL
751 ENKLKKYEYK NDKINDFTHN LYLSSRQNNK ESYGEEKEHL NNSYLKRSDN
801 SKGKYFDERK EEEYDEEEKF AYNILLNNQS DCLMKAQLLL KEELDKEIQK
851 KDEILIKLQK LISTLKEKNL IINEKNYKIK KYQLINENLQ DCIAGYQKDI
901 QILKENIHNL EKQIELKQIK NIVLPPKVHV HVSKLSSLEN NIKNELK**GII**

951 **GNSSNFLENT FK**YINENPLK KNLQNKAFFA TSGEKKIQDD ISKQGEQNKE
1001 EVSNFDSVVD VTGMAKNFLY NNMNILQNFK YDMKDNVVLK NDSFNENEPK
1051 YDDVKVNDQY VTVDNEKEVI YDERKNNIYD EGDNLYDNKD PYISINEEKE
1101 YIHSDNVSYD DDIKNVNVVS NEQFKNLNIS QNDEVSENKN IYFNLFFGKK
1151 SKKNNNNNNN NISSSSRNNS NNLNSKNEIL GSQNSTNSVV KADCDIKNLF
1201 DKNMKNENKK AKEETTAFFM KKFFPTNEND TSNNNKQNNK NNTESVFPTT
1251 YETPYHLNKQ HEHIYDELKG YRNEKILKNE NFCDNNMNTY NDLTYNSELY
1301 ISEEDTNKYD ANIEAQNVMD NSNNEEQIKK NLTHKSSEQY PQPNLSDEDP
1351 PISDVWNDKI DIDNFELENV

Peptides used for identification

Peptides shown in bold have been analysed by MS/MS sequencing

Start - End	Observed	Mr(expt)	Mr(calc)	Delta	Miss	Sequence
270 - 285	650.01	1947.01	1947.02	-3	1	K.**ILNINEDEDVIKYIEK**.L (Ions score 50)
948 - 962	820.92	1639.82	1639.82	0	0	K.**GIIGNSSNFLENTFK**.Y (Ions score 63)
948 - 962	820.92	1639.82	1639.82	1	0	K.**GIIGNSSNFLENTFK**.Y (Ions score 104)

Alphalyse A/S ■ Unsbjergvej 4 ■ 5220 Odense ■ Denmark
Tel: +45 6310 6500 ■ e-mail info@alphalyse.com
www.alphalyse.com

LC-MSMS Protein Identification Report

Order 16372_Plasmodium falciparum

Sample name: 6

Protein Information

**Protein name:**	**Pyruvate kinase OS=Plasmodium falciparum Vietnam Oak-Knoll (FVO) GN=PFFVO_01529 PE=3 SV=1**
Entry name:	A0A024VAV7_PLAFA
Calculated MW:	56480
Calculated pI:	7.5
Mascot score:	146
Sequence coverage:	4%
Bioinformatic tools:	1: UniProt Entry 2: Conserved Domains in NCBI

Analysis Information

- Enzyme: Trypsin
- Variable modifications: Oxidation (M)
- Fixed modifications: Carbamidomethyl (C)
- Database search program: Mascot version 2.4
- Peptide Tolerance: 10 ppm
- Database: UniprotTREMBL (50011027 protein sequences)

Protein sequence

Matched peptides shown in bold underline

1 MSSFKYKNSA AGASMQSAAN ITLR**QILEPN NVNLR**SKKTH IVCTLGPACK
51 SVETLVKLID AGMDICRFNF SHGSHEDHKE MFNNVLKAQE LRPNCLLGML
101 LDTKGPEIRT GFLKNKEVHL KEGSKLKLVT DYEFLGDETC IACSYKKLPQ
151 SVKPGNIILI ADGSVSCKVL ETHEDHVITE VLNSAVIGER KNMNLPNVKV
201 DLPIISEKDK NDILNFAIPM GCNFIAASFI QSADDVRLIR NLLGPRGRHI
251 KIIPKIENIE GIIHFDKILA ESDGIMIARG DLGMEISPEK VFLAQKLMIS
301 KCNLQGKPII TATQMLESMT KNPRPTRAEV TDVANAVLDG TDCVMLSGET
351 AGGKFPVEAV TIMSKICLEA EACIDYKLLY QSLVNAIETP ISVQEAVARS
401 AVETAESIQA SLIIALTETG YTARLIAKYK PSCTILALSA SDSTVKCLNV
451 HRGVTCIK**VG SFQGTDIVIR** NAIEIAKQRN MAKVGDSVIA IHGIKEEVSG
501 GTNLMKVVQI E

Peptides used for identification

Peptides shown in bold have been analysed by MS/MS sequencing

Start - End	Observed	Mr(expt)	Mr(calc)	Delta	Miss	Sequence
25 - 35	655.36	1308.71	1308.72	-4	0	**R.QILEPNNVNLR.S (Ions score 63)**
25 - 35	655.36	1308.71	1308.72	-4	0	**R.QILEPNNVNLR.S (Ions score 64)**
25 - 35	655.36	1308.71	1308.72	-3	0	**R.QILEPNNVNLR.S (Ions score 57)**
459 - 470	646.35	1290.69	1290.69	-3	0	**K.VGSFQGTDIVIR.N (Ions score 82)**

Alphalyse A/S ■ Unsbjergvej 4 ■ 5220 Odense ■ Denmark
Tel: +45 6310 6500 ■ e-mail info@alphalyse.com
www.alphalyse.com

459 - 470	646.35	1290.69	1290.69	-3	0	K.VGSFQGTDIVIR.N (Ions score 82)
459 - 470	646.35	1290.69	1290.69	-2	0	K.VGSFQGTDIVIR.N (Ions score 82)

Alphalyse A/S ■ Unsbjergvej 4 ■ 5220 Odense ■ Denmark
Tel: +45 6310 6500 ■ e-mail info@alphalyse.com
www.alphalyse.com

LC-MSMS Protein Identification Report

Order 16372_Plasmodium falciparum

Sample name: 6

Protein Information

**Protein name:**	**Eukaryotic initiation factor 4A OS=Plasmodium falciparum Vietnam Oak-Knoll (FVO) GN=PFFVO_05337 PE=3 SV=1**
Entry name:	A0A024UY98_PLAFA
Calculated MW:	46306
Calculated pI:	5.67
Mascot score:	126
Sequence coverage:	5%
Bioinformatic tools:	1: UniProt Entry 2: Conserved Domains in NCBI

Analysis Information

- Enzyme: Trypsin
- Variable modifications: Oxidation (M)
- Fixed modifications: Carbamidomethyl (C)
- Database search program: Mascot version 2.4
- Peptide Tolerance: 10 ppm
- Database: UniprotTREMBL (50011027 protein sequences)

Protein sequence

Matched peptides shown in bold underline

```
  1 MSTKEETFNN ENDIEGNTEE IVDTFDALGL NEKLLRGIYS YGFEKPSAIQ
 51 QRGIKPILNG YDTIGQAQSG TGKTATFVIS SLQLINYDYV ACQALILAPT
101 RELAQQIQKV VLALGDYLKV KCHACVGGTV VREDIDKLKQ GVHMVVGTPG
151 RVYDMIDKRH LGVDRLKLFI LDEADEMLSR GFKAQIYEVF KKLVPDIQVA
201 LFSATMPQEI LELTTRFMRD PKTILVKKDE LTLEGIRQFY VAVEKEEWKL
251 DTLCDLYETL TITQSIIYCN TRKKVDILTQ EMHNRLFTVS CMHGDMDQKD
301 RDLIMREFRS GSTRVLVTTD LLARGIDVQQ VSLVINYDLP ASPDTYIHRI
351 GRSGRFGRKG VAINFVTNDD KEKDKLKKIE SYYSTQIEEM PLEQNKVDHV
401 LYF
```

Peptides used for identification

Peptides shown in bold have been analysed by MS/MS sequencing

Start - End	Observed	Mr(expt)	Mr(calc)	Delta	Miss	Sequence
168 - 180	776.39	1550.77	1550.77	0	0	K.**LFILDEADEMLSR**.G (Ions score 68)
315 - 324	550.84	1099.66	1099.66	-2	0	R.**VLVTTDLLAR**.G (Ions score 59)
315 - 324	550.84	1099.66	1099.66	-1	0	R.**VLVTTDLLAR**.G (Ions score 54)

Alphalyse A/S ■ Unsbjergvej 4 ■ 5220 Odense ■ Denmark
Tel: +45 6310 6500 ■ e-mail info@alphalyse.com
www.alphalyse.com

Sample name: 6

Protein Information

**Protein name:**	**Polyadenylate-binding protein OS=Plasmodium falciparum Vietnam Oak-Knoll (FVO) GN=PFFVO_03505 PE=3 SV=1**
Entry name:	A0A024V3P9_PLAFA
Calculated MW:	97439
Calculated pI:	8.96
Mascot score:	122
Sequence coverage:	4%
Bioinformatic tools:	1: UniProt Entry 2: Conserved Domains in NCBI

Analysis Information

- Enzyme: Trypsin
- Variable modifications: Oxidation (M)
- Fixed modifications: Carbamidomethyl (C)
- Database search program: Mascot version 2.4
- Peptide Tolerance: 10 ppm
- Database: UniprotTREMBL (50011027 protein sequences)

Protein sequence

Matched peptides shown in bold underline

1 MIATGTNMMH PSFSTASLYV GDLNEDVTEA VLYEIFNTVG HVSSIRVCRD
51 SVTRKSLGYA YVNYHNLADA ERALDTLNYT NIKGQPARLM WSHRDPSLRK
101 SGTGNIFVKN LDKSIDNK**AL** **FDTFSMFGNI** **LSCK**VATDEF GKSKSYGFVH
151 YEDEESAKEA IEKVNGVQLG SKNVYVGPFI KKSERATNDT KFTNLYVKNF
201 PDSVTETHLR QLFNPYGEIT SMIVKMDNKN RKFCFINYAD AESAKNAMDN
251 LNGKKITDDG QIDETYDPKK EEATASTSGA ANQTTGTDAD KTDKNDKNNK
301 ADKNEKGDSS NANNNATATG ATTTDTTTTP GETTTTTANA DSTGANNNNG
351 LSPNTNTSNT TTGSSNNSIN LNENNNTAGN NNSTNNNNNS GSSMNNAGSA
401 KKDETAASDC ADTPNILYVG PHQSRARRHA ILKAKFDNLN VENKNKHQGV
451 NLYIKNLDDG IDDIMLRELF EPFGTITSAK VMRDEKEQSK GFGFVCFASQ
501 EEANKAVTEM HLKIINGKPL YVGLAEKREQ RLSRLQQRFR MHPIRHHMNN
551 PLNTPMQYAA PQSPQLQFSQ NTLSYGRPVI TAFNQNNLIS WRHQQAAQQQ
601 AVHQQAVHQQ AAQQQLNFNT NLRGQINQMR LYTQNNMMNN NLNQNKPNAQ
651 LHHNQQYVPN ALAQNGQQQP NLNAAGQHNA QQLQQQGNNQ LLNNNMRNMN
701 NRANRNMGNL GNMNNQKQLP LNINNKQQNA ASQANQMNHQ AQPQGAQAQQ
751 KNPQQMQQVP QGNNFKFTAQ ARNRMELPNK NANKVNTMNN NMNVNFNNNS
801 TLTAAALASA PPSMQK**QVLG** **ENLFPLVANY** **HPTLAGK**ITG MMLEMDNSEL
851 LILLENEEQL KKKIDEALVV LQKAK

Alphalyse A/S ■ Unsbjergvej 4 ■ 5220 Odense ■ Denmark
Tel: +45 6310 6500 ■ e-mail info@alphalyse.com
www.alphalyse.com

Peptides used for identification

Peptides shown in bold have been analysed by MS/MS sequencing

Start - End	Observed	Mr(expt)	Mr(calc)	Delta	Miss	Sequence
119 - 134	933.94	1865.87	1865.87	0	0	K.ALFDTFSMFGNILSCK.V + Oxidation (M) (Ions score 56)
119 - 134	933.94	1865.87	1865.87	1	0	K.ALFDTFSMFGNILSCK.V + Oxidation (M) (Ions score 30)
817 - 837	761.08	2280.22	2280.23	-2	0	K.QVLGENLFPLVANYHPTLAGK.I (Ions score 54)
817 - 837	761.08	2280.22	2280.23	-2	0	K.QVLGENLFPLVANYHPTLAGK.I (Ions score 65)

LC-MSMS Protein Identification Report

Order 16372_Plasmodium falciparum

Sample name: 6

Protein Information

Protein name:	Chaperone DnaK OS=Plasmodium falciparum Vietnam Oak-Knoll (FVO) GN=PFFVO_02477 PE=3 SV=1
Entry name:	A0A024V6C5_PLAFA
Calculated MW:	72457
Calculated pI:	5.18
Mascot score:	117
Sequence coverage:	5%
Bioinformatic tools:	1: UniProt Entry 2: Conserved Domains in NCBI

Analysis Information

- Enzyme: Trypsin
- Variable modifications: Oxidation (M)
- Fixed modifications: Carbamidomethyl (C)
- Database search program: Mascot version 2.4
- Peptide Tolerance: 10 ppm
- Database: UniprotTREMBL (50011027 protein sequences)

Protein sequence

Matched peptides shown in bold underline

1 MKQIRPYILL LIVSLLKFIS AVDSNIEGPV IGIDLGTTYS CVGVFKNGRV
51 EILNNELGNR ITPSYVSFVD GERKVGEAAK LEATLHPTQT VFDVKRLIGR
101 KFDDQEVVKD RSLLPYEIVN NQGKPNIKVQ IKDKDTTFAP EQISAMVLEK
151 MKEIAQSFLG KPVKNAVVTV PAYFNDAQRQ ATK**DAGTIAG LNIVR**IINEP
201 TAAALAYGLD KKEETSILVY DLGGGTFDVS ILVIDNGVFE VYATAGNTHL
251 GGEDFDQRVM DYFIKMFKKK NNIDLRTDKR AIQKLRKEVE IAKRNLSVVH
301 STQIEIEDIV EGHNFSETLT RAKFEELNDD LFRETLEPVK KVLDDAKYEK
351 SKIDEIVLVG GSTRIPKIQQ IIKEFFNGKE PNRGINPDEA VAYGAAIQAG
401 IILGEELQDV VLLDVTPLTL GIETVGGIMT QLIKRNTVIP TKK**SQTFSTY**
451 **QDNQPAVLIQ VFEGER**ALTK DNHLLGKFEL SGIPPAQRGV PKIEVTFTVD
501 KNGILHVEAE DKGTGKSRGI TITNDKGRLS KEQIEKMIND AEKFADEDKN
551 LREKVEAKNN LDNYIQSMKA TVEDKDKLAD KIEKEDKNTI LSAVKDAEDW
601 LNNNSNADSE ALKQKLKDLE AVCQPIIVKL YGQPGGPSPQ PSGDEDVDSD
651 EL

Peptides used for identification

Peptides shown in bold have been analysed by MS/MS sequencing

Start - End Observed Mr(expt) Mr(calc) Delta Miss Sequence

Alphalyse A/S ■ Unsbjergvej 4 ■ 5220 Odense ■ Denmark
Tel: +45 6310 6500 ■ e-mail info@alphalyse.com
www.alphalyse.com

184 - 195	600.34	1198.66	1198.67	-5	0	K.DAGTIAGLNIVR.I (Ions score 48)
444 - 466	1329.14	2656.27	2656.28	-2	0	K.SQTFSTYQDNQPAVLIQVFEGER.A (Ions score 31)
444 - 466	886.43	2656.28	2656.28	-1	0	K.SQTFSTYQDNQPAVLIQVFEGER.A (Ions score 69)
444 - 466	1329.15	2656.28	2656.28	0	0	K.SQTFSTYQDNQPAVLIQVFEGER.A (Ions score 28)
444 - 466	886.43	2656.28	2656.28	1	0	K.SQTFSTYQDNQPAVLIQVFEGER.A (Ions score 63)
444 - 466	886.43	2656.28	2656.28	1	0	K.SQTFSTYQDNQPAVLIQVFEGER.A (Ions score 59)

Alphalyse A/S ■ Unsbjergvej 4 ■ 5220 Odense ■ Denmark
Tel: +45 6310 6500 ■ e-mail info@alphalyse.com
www.alphalyse.com

Sample name: 6

Protein Information

**Protein name:**	**Uncharacterized protein OS=Plasmodium falciparum FCH/4 GN=PFFCH_00586 PE=4 SV=1**
Entry name:	A0A024VT85_PLAFA
Calculated MW:	13960
Calculated pI:	7.66
Mascot score:	88
Sequence coverage:	18%
Bioinformatic tools:	1: UniProt Entry 2: Conserved Domains in NCBI

Analysis Information

- Enzyme: Trypsin
- Variable modifications: Oxidation (M)
- Fixed modifications: Carbamidomethyl (C)
- Database search program: Mascot version 2.4
- Peptide Tolerance: 10 ppm
- Database: UniprotTREMBL (50011027 protein sequences)

Protein sequence

Matched peptides shown in bold underline

```
  1 MISGIRVNDN CVTEFNNMKI RKTCGWIIFV IQNCEIIIHS KGASTTLTEL
 51 VQSIDKNNEI QCAYVVFDAV SKIHFFMYAR ESSNSRDRMT YASSKQAILK
101 KIEGVNVLTS VIESAQDVAD LK
```

Peptides used for identification

Peptides shown in bold have been analysed by MS/MS sequencing

Start - End	Observed	Mr(expt)	Mr(calc)	Delta	Miss	Sequence
101 - 122	776.76	2327.25	2327.26	-2	1	**K.KIEGVNVLTSVIESAQDVADLK.-** (Ions score 88)
101 - 122	776.76	2327.26	2327.26	1	1	**K.KIEGVNVLTSVIESAQDVADLK.-** (Ions score 74)
101 - 122	776.76	2327.26	2327.26	2	1	**K.KIEGVNVLTSVIESAQDVADLK.-** (Ions score 46)

Alphalyse A/S ■ Unsbjergvej 4 ■ 5220 Odense ■ Denmark
Tel: +45 6310 6500 ■ e-mail info@alphalyse.com
www.alphalyse.com

LC-MSMS Protein Identification Report

Order 16372_Plasmodium falciparum

Sample name: 6

Protein Information

Protein name:	Uncharacterized protein OS=Plasmodium falciparum Vietnam Oak-Knoll (FVO) GN=PFFVO_02339 PE=4 SV=1
Entry name:	A0A024V7I0_PLAFA
Calculated MW:	99081
Calculated pI:	5.96
Mascot score:	70
Sequence coverage:	3%
Bioinformatic tools:	1: UniProt Entry 2: Conserved Domains in NCBI

Analysis Information

- Enzyme: Trypsin
- Variable modifications: Oxidation (M)
- Fixed modifications: Carbamidomethyl (C)
- Database search program: Mascot version 2.4
- Peptide Tolerance: 10 ppm
- Database: UniprotTREMBL (50011027 protein sequences)

Protein sequence

Matched peptides shown in bold underline

1 MYIYFVDEPE QFYWFVEHFL SVKFRVPKHL KDKNIHNFTP CLNRSWVSEF
51 LKEYEEPFVN PVMKFLDKEQ R**LFFTYNFGD** **VEPQGK**YTYF PVKEFHKYCI
101 LPPLIKTNIK DGESGEFLKY QLNKEEYKVF LSSVGSQMTA IKNLYSTVED
151 EQRKQLLKVI IENESTNDIS VQCPTYNIKL HYTKECANSN NILKCIDEFL
201 RKTCEKKTES KHPSADLCEH LQFLFESLKN PYLDNFKKFM TNSDFTLIKP
251 QSVWNVPIFD IYKPKNYLDS VQNLDTECFK KLNSKNLIFL SFHDDIPNNP
301 YYNVELQEIV KLSTYTYSIF DKLYNFFFVF KKSGAPISPV SVKELSHNIT
351 DFSFKEDNSE IQCQNVRKSL DLEVDVETMK GIAAEKLCKI IEKFILTKDD
401 AGKPEKSDIH RGFRILCILI STHVEAYNIV RQLLNMESMI SLTRYTSLYI
451 HKFFKSVTLL KGNFLYKNNK AIRYSRACSK ASLHVPSVLY RRNIYIPETF
501 LSLYLGLSNL VSSNPSSPFF EYAIIEFLVT YYNKGSEKFV LYFISIISVL
551 YINEYYYEQL SCFYPKEFEL IKSRMIHPNI VDRILKGIDN LMKSTRYDKM
601 RTMYLDFESS DIFSREK**VFT** **ALYNFDSFIK** TNEQLKKKNL EEISEIPVQL
651 ETSNDGIGYR KQDVLYETDK PQTMDEASYE ETVDEDAHHV NEKQHSAHFL
701 DAIAEKDILE EKTKDQDLEI ELYKYMGPLK EQSKSTSAAS TSDELSGSEG
751 PSTESTSTGN QGEDKTTDNT YKEMEELEEA EGTSNLKKGL EFYKSSLKLD
801 QLDKEKPKKK KSKRKKKRDS SSDRILLEES KTFTSENEL

Alphalyse A/S ■ Unsbjergvej 4 ■ 5220 Odense ■ Denmark
Tel: +45 6310 6500 ■ e-mail info@alphalyse.com
www.alphalyse.com

Peptides used for identification

Peptides shown in bold have been analysed by MS/MS sequencing

Start - End	Observed	Mr(expt)	Mr(calc)	Delta	Miss	Sequence
72 - 86	881.43	1760.84	1760.84	-1	0	**R.LFFTYNFGDVEPQGK.Y** (Ions score 36)
72 - 86	881.43	1760.84	1760.84	0	0	**R.LFFTYNFGDVEPQGK.Y** (Ions score 6)
618 - 630	782.91	1563.80	1563.80	0	0	**K.VFTALYNFDSFIK.T** (Ions score 35)

Alphalyse A/S ■ Unsbjergvej 4 ■ 5220 Odense ■ Denmark
Tel: +45 6310 6500 ■ e-mail info@alphalyse.com
www.alphalyse.com

Sample name: 6

Protein Information

Protein name:	Uncharacterized protein OS=Plasmodium falciparum Vietnam Oak-Knoll (FVO) GN=PFFVO_00066 PE=4 SV=1
Entry name:	A0A024VEQ1_PLAFA
Calculated MW:	18479
Calculated pI:	5.1
Mascot score:	53
Sequence coverage:	8%
Bioinformatic tools:	1: UniProt Entry 2: Conserved Domains in NCBI

Analysis Information

- Enzyme: Trypsin
- Variable modifications: Oxidation (M)
- Fixed modifications: Carbamidomethyl (C)
- Database search program: Mascot version 2.4
- Peptide Tolerance: 10 ppm
- Database: UniprotTREMBL (50011027 protein sequences)

Protein sequence

Matched peptides shown in bold underline

```
  1 MSGSNCVAIA CDLRLGANTF TTVSTKFSKI FKMNNNVYVG LSGLATDIQT
 51 LYEILRYRVN LYEVRQDAEM DVECFANMLS SILYSNRFSP YFVNPIVVGF
101 KLKHYVDEEG EKKVNYEPYL TAYDLIGAKC ETRDFVVNGV TSEQLFGMCE
151 SLYVKDQVKE
```

Peptides used for identification

Peptides shown in bold have been analysed by MS/MS sequencing

Start - End	Observed	Mr(expt)	Mr(calc)	Delta	Miss	Sequence
88 - 101	807.44	1612.86	1612.87	-1	0	**R.FSPYFVNPIVVGFK.L** (Ions score 53)
88 - 101	807.44	1612.87	1612.87	0	0	**R.FSPYFVNPIVVGFK.L** (Ions score 41)

Alphalyse A/S ■ Unsbjergvej 4 ■ 5220 Odense ■ Denmark
Tel: +45 6310 6500 ■ e-mail info@alphalyse.com
www.alphalyse.com

Sample name: 8

Protein Information

Protein name:	No identification
Entry name:	
Calculated MW:	
Calculated pI:	
Mascot score:	
Sequence coverage:	

Analysis Information

- Enzyme:
- Variable modifications:
- Database search program: Mascot version 2.4
- Peptide Tolerance:
- Database:

Alphalyse A/S ■ Unsbjergvej 4 ■ 5220 Odense ■ Denmark
Tel: +45 6310 6500 ■ e-mail info@alphalyse.com
www.alphalyse.com

LC-MSMS Protein Identification Report

Order 16372_Plasmodium falciparum

Sample name: 9

Protein Information

**Protein name:**	**Merozoite surface protein 1 OS=Plasmodium falciparum Vietnam Oak-Knoll (FVO) GN=PFFVO_02613 PE=4 SV=1**
Entry name:	A0A024V850_PLAFA
Calculated MW:	189101
Calculated pI:	5.98
Mascot score:	1031
Sequence coverage:	17%
Bioinformatic tools:	1: UniProt Entry 2: Conserved Domains in NCBI

Analysis Information

- Enzyme: Trypsin
- Variable modifications: Oxidation (M)
- Fixed modifications: Carbamidomethyl (C)
- Database search program: Mascot version 2.4
- Peptide Tolerance: 10 ppm
- Database: UniprotTREMBL (50011027 protein sequences)

Protein sequence

Matched peptides shown in bold underline

1 MKIIFFLCSF LFFIINTQCV THESYQELVK KLEALEDAVL TGYSLFQKEK
51 **MVLNEGTSGT** **AVTTSTPGSK** GSVASGGSGG SVASGGSVAS GGSVASGGSV
101 ASGGSVASGG SGNSRRTNPS DNSSDSDAKS YADLKHRVRN YLLTIKELKY
151 PQLFDLTNHM LTLCDNIHGF KYLIDGYEEI NELLYKLNFY FDLLRAKLND
201 VCANDYCQIP FNLKIRANEL DVLKKLVFGY RKPLDNIKDN VGKMEDYIKK
251 NKK**TIENINE** **LIEESKK**TID KNKNATKEEE KKK**LYQAQYD** **LSIYNK**QLEE
301 AHNLISVLEK RIDTLKKNEN IKELLDKINE IKNPPPANSG NTPNTLLDKN
351 KKIEEHEKEI KEIAKTIKFN IDSLFTDPLE LEYYLREKNK NIDISAKVET
401 KESTEPNEYP NGVTYPLSYN DINNALNELN SFGDLINPFD YTKEPSKNIY
451 TDNERKKFIN EIKEKIKIEK KKIESDKKSY EDRSKSLNDI TKEYEKLLNE
501 IYDSK**FNNNI** **DLTNFEK**MMG KRYSYKVEKL THHNTFASYE NSKHNLEKLT
551 KALKYMEDYS LRNIVVEKEL KYYKNLISKI ENEIETLVEN IKKDEEQLFE
601 KKITKDENKP DEKILEVSDI VKVQVQKVLL MNKIDELKKT QLILKNVELK
651 HNIHVPNSYK QENKQEPYYL IVLKKEIDKL KVFMPK**VESL** **INEEKK**NIKT
701 EGQSDNSEPS TEGEITGQAT TKPGQQAGSA LEGDSVQAQA QEQK**QAQPPV**
751 **PVPVPEAK**AQ VPTPPAPVNN KTENVSKLDY LEKLYEFLNT SYICHKYILV
801 SHSTMNEKIL KQYKITKEEE SKLSSCDPLD LLFNIQNNIP VMYSMFDSLN
851 NSLSQLFMEI YEKEMVCNLY KLKDNDKIKN LLEEAKKVST SVK**TLSSSSM**
901 **QPLSLTPQDK** **PEVSANDDTS** **HSTNLNNSLK** **LFENILSLGK** **NKNIYQELIG**
951 **QK**SSENFYEK **ILKDSDTFYN** **ESFTNFVK**SK ADDINSLNDE SKRKKLEEDI

1001 NKLKKTLQLS FDLYNKYKLK LERLFDKKKT VGKYKMQIKK LTLLKEQLES
1051 KLNSLNNPK**H** **VLQNFSVFFN** **K**KK**EAEIAET** **ENTLENTK**IL LKHYKGLVKY
1101 YNGESSPLK**T** **LSEESIQTED** **NYASLENFK**V LSKLEGKLKD NLNLEKKKLS
1151 YLSSGLHHLI AELKEVIKNK NYTGNSPSEN NTDVNNALES YKK**FLPEGTD**
1201 **VATVVSESGS** **DTLEQSQPK**K PASTHVGAES NTITTSQNVD DEVDDVIIVP
1251 IFGESEEDYD DLGQVVTGEA VTPSVIDNIL SKIENEYEVL YLKPLAGVYR
1301 SLKK**QLENNV** **MTFNVNVK**DI LNSRFNKREN FKNVLESDLI PYKDLTSSNY
1351 VVKDPYKFLN KEKRDKFLSS YNYIKDSIDT DINFANDVLG YYKILSEKYK
1401 SDLDSIKKYI NDKQGENEKY LPFLNNIETL YKTVNDKIDL FVIHLEAKVL
1451 NYTYEKSNVE VKIKELNYLK TIQDKLADFK K**NNNFVGIAD** **LSTDYNHNNL**
1501 **LTKFLSTGMV** **FENLAK**TVLS NLLDGNLQGM LNISQHQCVK KQCPQNSGCF
1551 RHLDEREECK CLLNYKQEGD KCVENPNPTC NENNGGCDAD AKCTEEDSGS
1601 NGKKITCECT KPDSYPLFDG IFCSSSNFLG ISFLLILMLI LYSFI

Peptides used for identification

Peptides shown in bold have been analysed by MS/MS sequencing

Start - End	Observed	Mr(expt)	Mr(calc)	Delta	Miss	Sequence
51 - 70	977.47	1952.93	1952.94	-1	0	K.MVLNEGTSGTAVTTSTPGSK.G + Oxidation (M) (Ions score 74)
51 - 70	977.48	1952.94	1952.94	0	0	K.MVLNEGTSGTAVTTSTPGSK.G + Oxidation (M) (Ions score 76)
254 - 267	830.44	1658.87	1658.87	-3	1	K.TIENINELIEESKK.T (Ions score 43)
254 - 267	553.96	1658.87	1658.87	-1	1	K.TIENINELIEESKK.T (Ions score 56)
254 - 267	830.44	1658.87	1658.87	-1	1	K.TIENINELIEESKK.T (Ions score 84)
254 - 267	553.96	1658.87	1658.87	-1	1	K.TIENINELIEESKK.T (Ions score 57)
254 - 267	830.44	1658.87	1658.87	0	1	K.TIENINELIEESKK.T (Ions score 65)
254 - 267	553.96	1658.87	1658.87	0	1	K.TIENINELIEESKK.T (Ions score 84)
254 - 267	830.44	1658.87	1658.87	0	1	K.TIENINELIEESKK.T (Ions score 87)
254 - 267	553.97	1658.87	1658.87	1	1	K.TIENINELIEESKK.T (Ions score 62)
284 - 296	809.91	1617.80	1617.80	-2	0	K.LYQAQYDLSIYNK.Q (Ions score 58)
284 - 296	809.91	1617.80	1617.80	0	0	K.LYQAQYDLSIYNK.Q (Ions score 77)
284 - 296	809.91	1617.81	1617.80	1	0	K.LYQAQYDLSIYNK.Q (Ions score 84)
506 - 517	734.86	1467.70	1467.70	-1	0	K.FNNNIDLTNFEK.M (Ions score 33)
506 - 517	734.86	1467.70	1467.70	2	0	K.FNNNIDLTNFEK.M (Ions score 48)
687 - 696	594.82	1187.63	1187.64	-5	1	K.VESLINEEKK.N (Ions score 18)
687 - 696	594.83	1187.64	1187.64	-1	1	K.VESLINEEKK.N (Ions score 12)
687 - 696	594.83	1187.64	1187.64	0	1	K.VESLINEEKK.N (Ions score 29)
745 - 758	728.91	1455.80	1455.81	-3	0	K.QAQPPVPVPVPEAK.A (Ions score 54)
745 - 758	728.91	1455.81	1455.81	-2	0	K.QAQPPVPVPVPEAK.A (Ions score 35)
745 - 758	728.91	1455.81	1455.81	-1	0	K.QAQPPVPVPVPEAK.A (Ions score 32)
745 - 758	728.91	1455.81	1455.81	-1	0	K.QAQPPVPVPVPEAK.A (Ions score 49)
745 - 758	728.91	1455.81	1455.81	0	0	K.QAQPPVPVPVPEAK.A (Ions score 66)
894 - 930	990.72	3958.87	3958.87	-1	0	K.TLSSSSMQPLSLTPQDKPEVSANDDTSHSTNLNNSLK.L + Oxidation (M) (Ions score 20)
894 - 930	990.73	3958.87	3958.87	0	0	K.TLSSSSMQPLSLTPQDKPEVSANDDTSHSTNLNNSLK.L + Oxidation (M) (Ions score 9)
931 - 940	567.33	1132.65	1132.65	-2	0	K.LFENILSLGK.N (Ions score 63)
931 - 940	567.33	1132.65	1132.65	-2	0	K.LFENILSLGK.N (Ions score 20)
931 - 940	567.33	1132.65	1132.65	-1	0	K.LFENILSLGK.N (Ions score 15)
931 - 940	567.33	1132.65	1132.65	1	0	K.LFENILSLGK.N (Ions score 22)
941 - 952	724.40	1446.78	1446.78	-1	1	K.NKNIYQELIGQK.S (Ions score 60)
941 - 952	724.40	1446.78	1446.78	0	1	K.NKNIYQELIGQK.S (Ions score 32)
941 - 952	724.40	1446.78	1446.78	1	1	K.NKNIYQELIGQK.S (Ions score 45)
961 - 978	723.36	2167.05	2167.05	1	1	K.ILKDSDTFYNESFTNFVK.S (Ions score 32)
961 - 978	723.36	2167.05	2167.05	2	1	K.ILKDSDTFYNESFTNFVK.S (Ions score 78)
961 - 978	723.36	2167.06	2167.05	4	1	K.ILKDSDTFYNESFTNFVK.S (Ions score 37)

Alphalyse A/S ■ Unsbjergvej 4 ■ 5220 Odense ■ Denmark
Tel: +45 6310 6500 ■ e-mail info@alphalyse.com
www.alphalyse.com

964 - 978	907.40	1812.78	1812.78	0	0	K.DSDTFYNESFTNFVK.S (Ions score 37)
964 - 978	907.40	1812.79	1812.78	1	0	K.DSDTFYNESFTNFVK.S (Ions score 34)
964 - 978	907.40	1812.79	1812.78	2	0	K.DSDTFYNESFTNFVK.S (Ions score 46)
1060 - 1071	740.39	1478.76	1478.77	-3	0	K.HVLQNFSVFFNK.K (Ions score 28)
1060 - 1071	740.39	1478.77	1478.77	-1	0	K.HVLQNFSVFFNK.K (Ions score 51)
1060 - 1071	740.39	1478.77	1478.77	1	0	K.HVLQNFSVFFNK.K (Ions score 20)
1074 - 1088	846.40	1690.79	1690.79	-2	0	K.EAEIAETENTLENTK.I (Ions score 82)
1074 - 1088	846.40	1690.79	1690.79	-1	0	K.EAEIAETENTLENTK.I (Ions score 89)
1074 - 1088	846.40	1690.79	1690.79	1	0	K.EAEIAETENTLENTK.I (Ions score 91)
1110 - 1129	1159.54	2317.06	2317.06	0	0	K.TLSEESIQTEDNYASLENFK.V (Ions score 88)
1110 - 1129	1159.54	2317.06	2317.06	0	0	K.TLSEESIQTEDNYASLENFK.V (Ions score 59)
1110 - 1129	1159.54	2317.06	2317.06	0	0	K.TLSEESIQTEDNYASLENFK.V (Ions score 82)
1194 - 1219	907.77	2720.30	2720.30	-2	0	K.FLPEGTDVATVVSESGSDTLEQSQPK.K (Ions score 49)
1194 - 1219	907.77	2720.30	2720.30	-1	0	K.FLPEGTDVATVVSESGSDTLEQSQPK.K (Ions score 57)
1194 - 1219	907.78	2720.31	2720.30	1	0	K.FLPEGTDVATVVSESGSDTLEQSQPK.K (Ions score 45)
1305 - 1318	833.42	1664.82	1664.82	1	0	K.QLENNVMTFNVNVK.D + Oxidation (M) (Ions score 9)
1482 - 1503	826.41	2476.20	2476.20	0	0	K.NNNFVGIADLSTDYNHNNLLTK.F (Ions score 9)
1482 - 1503	826.41	2476.20	2476.20	0	0	K.NNNFVGIADLSTDYNHNNLLTK.F (Ions score 21)
1504 - 1516	728.88	1455.74	1455.74	-2	0	K.FLSTGMVFENLAK.T (Ions score 9)
1504 - 1516	728.88	1455.75	1455.74	2	0	K.FLSTGMVFENLAK.T (Ions score 49)
1504 - 1516	736.88	1471.74	1471.74	-2	0	K.FLSTGMVFENLAK.T + Oxidation (M) (Ions score 60)
1504 - 1516	736.88	1471.74	1471.74	1	0	K.FLSTGMVFENLAK.T + Oxidation (M) (Ions score 37)
1504 - 1516	736.88	1471.74	1471.74	2	0	K.FLSTGMVFENLAK.T + Oxidation (M) (Ions score 28)

Alphalyse A/S ■ Unsbjergvej 4 ■ 5220 Odense ■ Denmark
Tel: +45 6310 6500 ■ e-mail info@alphalyse.com
www.alphalyse.com

LC-MSMS Protein Identification Report

Order 16372_Plasmodium falciparum

Sample name: 9

Protein Information

**Protein name:**	**Uncharacterized protein OS=Plasmodium falciparum UGT5.1 GN=C923_03132 PE=4 SV=1**
Entry name:	W7JXD3_PLAFA
Calculated MW:	124344
Calculated pI:	4.28
Mascot score:	440
Sequence coverage:	12%
Bioinformatic tools:	1: UniProt Entry 2: Conserved Domains in NCBI

Analysis Information

- Enzyme: Trypsin
- Variable modifications: Oxidation (M)
- Fixed modifications: Carbamidomethyl (C)
- Database search program: Mascot version 2.4
- Peptide Tolerance: 10 ppm
- Database: UniprotTREMBL (50011027 protein sequences)

Protein sequence

Matched peptides shown in bold underline

1 MRNLFHITIC LVTLNLFILE INAKTNTSEN RNKRIGGPKL RGNVTSNIKF
51 PSDNKGKIIR GSNDQLNKNS EDVLEQSEKS LVSENVPSGL DIDDIPKESI
101 FIQEDQEGQT HSELNPETSE HSKDLNNNGS KNESSDIISE NNKSNKVQNH
151 FESLSDLELL ENSSQDNLDK DTISTEPFPN QKHKDLQQDL NDEPLEPFPT
201 QIHKDYKEK**N** **LINEEDSEPF** **PR**QKHKKVDN HNEEKNVFHE NGSANGNQGS
251 LKLKSFDEHL KDEKIENEPL VHENLSIPND PIEQILNQPE QETNIQEQLY
301 NEKQNVEEKQ NSQIPSLDLK EPTNEDILPN HNPLENIKQS ESEINHVQDH
351 ALPKENIIDK LDNQKEHIDQ SQHNINVLQE NNINNHQLEP QEKPNIESFE
401 PKNIDSEIIL PENVETEEII DDVPSPKHSN HETFEEETSE SEHEEAVSEK
451 NAHETVEHEE TVSQESNPEK ADNDGNVSQN SNNELNENEF VESEKSEHEP
501 AENEESSLEE GHHEEIVPEQ NNEESGESKL VDNDEGGFEE AHHENFSSEV
551 SNSELNENEF VESDKSVTEP AEHEEVVSEE SNPEPAENEE SSIEEGHQEE
601 IVPEQNDEES GESGLVDNEE GDFEEPNHEE FEPDQNDSEL SENELVESEK
651 **SVSEPAEHVE** **IVSEKSASEP** **AEHVEIVSEK** SVSEPAEHVE SVSEQSNNEP
701 SEKK**DGPVPS** **KPFEEIEKVD** **VQPK**IVDLQI IEPNFVDSQP NPQEPVEPSF
751 VKIEKVPSEE NKHASVDPEV KEKENVSEVV EEKQNSQESV EEIPVNEDEF
801 EDVHTEQLDL DHKTVDPEIV EVEEIPSELH ENEVAHPEIV EIEEVFPEPN
851 QNNEFQEINE DDKSAHIQHE IVEVEEILPE DDKNEKVEEI LPEEDKNEKG
901 QHEIVEVEEI LPEIVEIEEV PSQTNNNENI ETIKPEEKKN EFSVEEK**AIP**

Alphalyse A/S ■ Unsbjergvej 4 ■ 5220 Odense ■ Denmark
Tel: +45 6310 6500 ■ e-mail info@alphalyse.com
www.alphalyse.com

951 **QEPVVPTLNE NENVPPKPSE GESTKPDIVQ IK**IVQENKPN KKETPVVDGP
1001 K**HVEQNIQED DNDEEDDDDI DFEGLSR**KDD EKDSSNKNKK K**SSFITYIST**
1051 **K**KFKKVSQTI VSVMINAYDG VIQVVSTIKG IAKDIVIFFQ NI

Peptides used for identification

Peptides shown in bold have been analysed by MS/MS sequencing

Start - End	Observed	Mr(expt)	Mr(calc)	Delta	Miss	Sequence
210 - 222	780.37	1558.73	1558.73	-1	0	**K.NLINEEDSEPFPR.Q (Ions score 74)**
210 - 222	780.37	1558.73	1558.73	-1	0	**K.NLINEEDSEPFPR.Q (Ions score 67)**
210 - 222	780.37	1558.73	1558.73	0	0	**K.NLINEEDSEPFPR.Q (Ions score 74)**
651 - 665	547.28	1638.81	1638.81	-2	0	**K.SVSEPAEHVEIVSEK.S (Ions score 34)**
651 - 665	820.41	1638.81	1638.81	-1	0	**K.SVSEPAEHVEIVSEK.S (Ions score 62)**
651 - 665	820.41	1638.81	1638.81	-1	0	**K.SVSEPAEHVEIVSEK.S (Ions score 39)**
651 - 680	808.90	3231.56	3231.58	-6	1	**K.SVSEPAEHVEIVSEKSASEPAEHVEIVSEK.S (Ions score 27)**
651 - 680	808.90	3231.57	3231.58	-3	1	**K.SVSEPAEHVEIVSEKSASEPAEHVEIVSEK.S (Ions score 21)**
651 - 680	808.90	3231.58	3231.58	1	1	**K.SVSEPAEHVEIVSEKSASEPAEHVEIVSEK.S (Ions score 19)**
666 - 680	806.40	1610.78	1610.78	-1	0	**K.SASEPAEHVEIVSEK.S (Ions score 40)**
666 - 680	806.40	1610.78	1610.78	-1	0	**K.SASEPAEHVEIVSEK.S (Ions score 15)**
705 - 724	746.73	2237.15	2237.16	-1	1	**K.DGPVPSKPFEEIEKVDVQPK.I (Ions score 40)**
705 - 724	746.73	2237.16	2237.16	-1	1	**K.DGPVPSKPFEEIEKVDVQPK.I (Ions score 41)**
705 - 724	746.73	2237.16	2237.16	0	1	**K.DGPVPSKPFEEIEKVDVQPK.I (Ions score 19)**
705 - 724	746.73	2237.16	2237.16	1	1	**K.DGPVPSKPFEEIEKVDVQPK.I (Ions score 65)**
948 - 982	949.25	3792.97	3792.98	-3	0	**K.AIPQEPVVPTLNENENVPPKPSEGESTKPDIVQIK.I (Ions score 22)**
948 - 982	949.25	3792.98	3792.98	0	0	**K.AIPQEPVVPTLNENENVPPKPSEGESTKPDIVQIK.I (Ions score 22)**
948 - 982	949.25	3792.98	3792.98	0	0	**K.AIPQEPVVPTLNENENVPPKPSEGESTKPDIVQIK.I (Ions score 11)**
1002 - 1027	1031.09	3090.24	3090.24	0	0	**K.HVEQNIQEDDNDEEDDDDIDFEGLSR.K (Ions score 122)**
1002 - 1027	1031.09	3090.24	3090.24	0	0	**K.HVEQNIQEDDNDEEDDDDIDFEGLSR.K (Ions score 113)**
1042 - 1051	573.80	1145.59	1145.60	-2	0	**K.SSFITYISTK.K (Ions score 29)**
1042 - 1051	573.81	1145.60	1145.60	0	0	**K.SSFITYISTK.K (Ions score 27)**

Alphalyse A/S ■ Unsbjergvej 4 ■ 5220 Odense ■ Denmark
Tel: +45 6310 6500 ■ e-mail info@alphalyse.com
www.alphalyse.com

Sample name: 9

Protein Information

**Protein name:**	**Glutamate-rich protein OS=Plasmodium falciparum (isolate 3D7) GN=PF10_0344 PE=4 SV=1**
Entry name:	Q8IJ56_PLAF7
Calculated MW:	141024
Calculated pI:	4.28
Mascot score:	406
Sequence coverage:	8%
Bioinformatic tools:	1: UniProt Entry 2: Conserved Domains in NCBI

Analysis Information

- Enzyme: Trypsin
- Variable modifications: Oxidation (M)
- Fixed modifications: Carbamidomethyl (C)
- Database search program: Mascot version 2.4
- Peptide Tolerance: 10 ppm
- Database: UniprotTREMBL (50011027 protein sequences)

Protein sequence

Matched peptides shown in bold underline

**1**	MRNLFHITIC	LVTLNLFILE	INAKTNTSEN	RNKRIGGPKL	RGNVTSNIKF
**51**	PSDNKGKIIR	GSNDKLNKNS	EDVLEQSEKS	LVSENVPSGL	DIDDIPKESI
**101**	FIQEDQEGQT	HSELNPETSE	HSKDLNNNDS	KNESSDIISV	NNKSNKVQNH
**151**	FESLSDLELL	ENSSQDNLDK	DTISTEPFPN	QKHKDLQQDL	NDEPLEPFPT
**201**	QIHKDYKEK**N**	**LINEEDSEPF**	**PR**QKHKKVDN	HNEEKNVFHE	NGSANGNQGS
**251**	LKLKSFDEHL	KDEKIENEPL	VHENLSIPND	PIEQILNQPE	QETNIQEQLY
**301**	NEKQNVEEKQ	NSQIPSLDLK	EPTNEDILPN	HNPLENIKQS	ESEINHVQDH
**351**	ALPKENIIDK	LDNQKEHIDQ	SQHNINVLQE	NNINNHQLEP	QEKPNIESFE
**401**	PKNIDSEIIL	PENVETEEII	DDVPSPKHSN	HETFEEETSE	SEHEEAVSEK
**451**	NAHETVEHEE	TVSQESNPEK	ADNDGNVSQN	SNNELNENEF	VESEKSEHEP
**501**	AENEESSLEE	GHHEEIVPEQ	NNEESGESKL	VDNDEGGFEE	AHHENFSSEV
**551**	SNSELNENEF	VESDKSVTEP	AEHEEVVSEE	SNPEPAENEE	SSIEEAHQEE
**601**	IVPEQNDEES	GESGLVDNEE	GDFEEPNHEE	FEPDQNDSEL	SENELVESEK
**651**	**SVSEPAEHVE**	**IVSEK**SVSEP	AEHVEIVSEK	STSEPAEHVE	SVSEQSNNEP
**701**	SEKK**DGPVPS**	**KPFEEIEKVD**	**VQPK**IVDLQI	IEPNFVDSQP	NPQEPVEPSF
**751**	VKIEKVPSEE	NKHASVDPEV	KEKENVSEVV	EEKQNSQESV	EEIPVNEDEF
**801**	EDVHTEQLDL	DHKTVDPEIV	EVEEIPSELH	ENEVAHPEIV	EIEEVFPEPN
**851**	QNNEFQEINE	DDKSAHIQHE	IVEVEEILPE	DDKNEKVEHE	IVEVEEILPE
**901**	DKNEKVQHEI	VEVEEILPED	KNEKVEHEIV	EVEEILPEDK	NEK**GQHEIVE**

Alphalyse A/S ■ Unsbjergvej 4 ■ 5220 Odense ■ Denmark
Tel: +45 6310 6500 ■ e-mail info@alphalyse.com
www.alphalyse.com

951 **VEEILPEDKN** **EK**VQHEIVEV EEILPEDKNE KGQHEIVEVE EILPEDKNEK
1001 VEHEIVEVEE ILPEDKNEKG QHEIVEVEEI LPEDDKNEKG QHEIVEVEEI
1051 LPEIVEIEEV PSQTNNNENI ETIKPEEKKN EFSVVEEKAI PQEPVVPTLN
1101 ENENVTPKPS EGESTKPDIV QIKIVQENKP NKKETPVVDG PK**HVEQNIQE**
1151 **DDNDEEDDDD** **IDFEGLSR**KD DEKDSSNKNK KK**SSFITYIS** **TK**KFKKVSQT
1201 IVSVMINAYD GVIQVVSTIK GIAKDIVIFF QNI

Peptides used for identification

Peptides shown in bold have been analysed by MS/MS sequencing

Start - End	Observed	Mr(expt)	Mr(calc)	Delta	Miss	Sequence
210 - 222	780.37	1558.73	1558.73	-1	0	K.**NLINEEDSEPFPR**.Q (Ions score 74)
210 - 222	780.37	1558.73	1558.73	-1	0	K.**NLINEEDSEPFPR**.Q (Ions score 67)
210 - 222	780.37	1558.73	1558.73	0	0	K.**NLINEEDSEPFPR**.Q (Ions score 74)
651 - 665	547.28	1638.81	1638.81	-2	0	K.**SVSEPAEHVEIVSEK**.S (Ions score 34)
651 - 665	820.41	1638.81	1638.81	-1	0	K.**SVSEPAEHVEIVSEK**.S (Ions score 62)
651 - 665	820.41	1638.81	1638.81	-1	0	K.**SVSEPAEHVEIVSEK**.S (Ions score 39)
705 - 724	746.73	2237.15	2237.16	-1	1	K.**DGPVPSKPFEEIEKVDVQPK**.I (Ions score 40)
705 - 724	746.73	2237.16	2237.16	-1	1	K.**DGPVPSKPFEEIEKVDVQPK**.I (Ions score 41)
705 - 724	746.73	2237.16	2237.16	0	1	K.**DGPVPSKPFEEIEKVDVQPK**.I (Ions score 19)
705 - 724	746.73	2237.16	2237.16	1	1	K.**DGPVPSKPFEEIEKVDVQPK**.I (Ions score 65)
944 - 962	745.71	2234.11	2234.11	1	1	K.**GQHEIVEVEEILPEDKNEK**.V (Ions score 56)
944 - 962	745.71	2234.11	2234.11	2	1	K.**GQHEIVEVEEILPEDKNEK**.V (Ions score 54)
1143 - 1168	1031.09	3090.24	3090.24	0	0	K.**HVEQNIQEDDNDEEDDDDIDFEGLSR**.K (Ions score 122)
1143 - 1168	1031.09	3090.24	3090.24	0	0	K.**HVEQNIQEDDNDEEDDDDIDFEGLSR**.K (Ions score 113)
1183 - 1192	573.80	1145.59	1145.60	-2	0	K.**SSFITYISTK**.K (Ions score 29)
1183 - 1192	573.81	1145.60	1145.60	0	0	K.**SSFITYISTK**.K (Ions score 27)

Alphalyse A/S ■ Unsbjergvej 4 ■ 5220 Odense ■ Denmark
Tel: +45 6310 6500 ■ e-mail info@alphalyse.com
www.alphalyse.com

LC-MSMS Protein Identification Report

Order 16372_Plasmodium falciparum

Sample name: 9

Protein Information

**Protein name:**	**Merozoite surface protein 1 (Fragment) OS=Plasmodium falciparum GN=MSP-1 PE=2 SV=1**
Entry name:	Q8T6A9_PLAFA
Calculated MW:	75823
Calculated pI:	7.26
Mascot score:	317
Sequence coverage:	10%
Bioinformatic tools:	1: UniProt Entry 2: Conserved Domains in NCBI

Analysis Information

- Enzyme: Trypsin
- Variable modifications: Oxidation (M)
- Fixed modifications: Carbamidomethyl (C)
- Database search program: Mascot version 2.4
- Peptide Tolerance: 10 ppm
- Database: UniprotTREMBL (50011027 protein sequences)

Protein sequence

Matched peptides shown in bold underline

```
  1 VTHESYQELV KKLEALEDAV LTGYSLFQKE KMVLNEGTSG TAVTTSTPGS
 51 KGSVASGGSG GSVASGGSVA SGGSVASGGS VASGGSGNSR RTNPSDNSSD
101 SDAKSYADLK HRVRNYLLTI KELKYPQLFD LTNHMLTLCD NIHGFKYLID
151 GYEEINELLY KLNFYFDLLR AKLNDVCAND YCQIPFNLKI RANELDVLKK
201 LVFGYRKPLD NIKDNVGKME DYIKKNKKTI ENINELIEES KKTIDKNKNA
251 TKEEEKKKLY QAQYDLSIYN KQLEEAHNLI SVLEKRIDTL KKNENIKELL
301 DKINEIKNPP PANSGNTPNT LLDKNKKIEE HEKEIKEIAK TIKFNIDSLF
351 TDPLELEYYL REKNKNIDIS AKVETKESTE PNEYPNGVTY PLSYNDINNA
401 LNELNSFGDL INPFDYTKEP SKNIYTDNER KKFINEIKEK IKIEKKKIES
451 DKKSYEDRSK SLNDITKEYE KLLNEIYDSK FNNNIDLTNF EKMMGKRYSY
501 KVEKLTHHNT FASYENSKHN LEKLTKALKY MEDYSLRNIV VEKELKYYKN
551 LISKIENEIE TLVENIKKDE EQLFEKKITK DENKPDEKIL EVSDIVKVQV
601 QKVLLMNKID ELKKTQLILK NVELKHNIHV PNSYKQENKQ EPYYLIVLKK
651 EIDKLK
```

Matched peptides (bold underline): 1 VTHESYQELV, K (11), MVLNEGTSG TAVTTSTPGS, K (51); 229 TI ENINELIEES KK; 259 LY QAQYDLSIYN K; 481 FNNNIDLTNF EK

Peptides used for identification

Peptides shown in bold have been analysed by MS/MS sequencing

Start - End Observed Mr(expt) Mr(calc) Delta Miss Sequence

Alphalyse A/S ■ Unsbjergvej 4 ■ 5220 Odense ■ Denmark
Tel: +45 6310 6500 ■ e-mail info@alphalyse.com
www.alphalyse.com

1 - 11	666.84	1331.67	1331.67	-3	0	-.VTHESYQELVK.K (Ions score 12)
1 - 11	666.84	1331.67	1331.67	-1	0	-.VTHESYQELVK.K (Ions score 14)
1 - 11	666.84	1331.67	1331.67	-1	0	-.VTHESYQELVK.K (Ions score 24)
1 - 11	666.85	1331.68	1331.67	3	0	-.VTHESYQELVK.K (Ions score 16)
32 - 51	977.47	1952.93	1952.94	-1	0	K.MVLNEGTSGTAVTTSTPGSK.G + Oxidation (M) (Ions score 74)
32 - 51	977.48	1952.94	1952.94	0	0	K.MVLNEGTSGTAVTTSTPGSK.G + Oxidation (M) (Ions score 76)
229 - 242	830.44	1658.87	1658.87	-3	1	K.TIENINELIEESKK.T (Ions score 43)
229 - 242	553.96	1658.87	1658.87	-1	1	K.TIENINELIEESKK.T (Ions score 56)
229 - 242	830.44	1658.87	1658.87	-1	1	K.TIENINELIEESKK.T (Ions score 84)
229 - 242	553.96	1658.87	1658.87	-1	1	K.TIENINELIEESKK.T (Ions score 57)
229 - 242	830.44	1658.87	1658.87	0	1	K.TIENINELIEESKK.T (Ions score 65)
229 - 242	553.96	1658.87	1658.87	0	1	K.TIENINELIEESKK.T (Ions score 84)
229 - 242	830.44	1658.87	1658.87	0	1	K.TIENINELIEESKK.T (Ions score 87)
229 - 242	553.97	1658.87	1658.87	1	1	K.TIENINELIEESKK.T (Ions score 62)
259 - 271	809.91	1617.80	1617.80	-2	0	K.LYQAQYDLSIYNK.Q (Ions score 58)
259 - 271	809.91	1617.80	1617.80	0	0	K.LYQAQYDLSIYNK.Q (Ions score 77)
259 - 271	809.91	1617.81	1617.80	1	0	K.LYQAQYDLSIYNK.Q (Ions score 84)
481 - 492	734.86	1467.70	1467.70	-1	0	K.FNNNIDLTNFEK.M (Ions score 33)
481 - 492	734.86	1467.70	1467.70	2	0	K.FNNNIDLTNFEK.M (Ions score 48)

LC-MSMS Protein Identification Report

Order 16372_Plasmodium falciparum

Sample name: 9

Protein Information

**Protein name:**	**Uncharacterized protein OS=Plasmodium falciparum Vietnam Oak-Knoll (FVO) GN=PFFVO_03053 PE=4 SV=1**
Entry name:	A0A024V579_PLAFA
Calculated MW:	94874
Calculated pI:	4.34
Mascot score:	305
Sequence coverage:	12%
Bioinformatic tools:	1: UniProt Entry 2: Conserved Domains in NCBI

Analysis Information

- Enzyme: Trypsin
- Variable modifications: Oxidation (M)
- Fixed modifications: Carbamidomethyl (C)
- Database search program: Mascot version 2.4
- Peptide Tolerance: 10 ppm
- Database: UniprotTREMBL (50011027 protein sequences)

Protein sequence

Matched peptides shown in bold underline

1 MRNLFHITIC LVTLNLFILE ISAKTNTSEN RNKRIGGPKL RGNVTSNIKL
51 PSNNKGKIIR **GSNDELNKNS** **EDVLEQSEK**S LVSENVPSGL DIDDIPKESI
101 FIQEDQEGQT HSELNPETSE HSKDLNNNDS KNESSDIISE NNKSNKVQNH
151 FESLSDLELL ENSSQDNLDK DTISTEPFPN QKHKDLQQDL NDEPLEPFPT
201 QIHKDYKEK**N** **LINEEDSEPF** **PR**QEHKKVDN HNEEKNVFHE NGSANGNQGS
251 LKLKSFDEHL KDEKIENEPL VHENLSIPND PIEQILNQPE QETNIQEQLY
301 NEKQNVEEKQ NSQIPSLDLK EPTNEDILPN HNPLENIKQS ESEINHVQDH
351 ALPKENIIDK LDNQKEHIDQ SQHNINVLQE NNINNHQLEP QEKPNIESFE
401 PKNIDSEIIL PENVETEEII DDVPSPKHSN HETFEEETSE SEHEEAVSEK
451 NAHETVEHEE TVSQESNPEK ADNDGNVSQN SNNELNENEF VESEKSEHEA
501 AENEESSLEE GHHEEIVPEQ NNEESGESKL VDNDEGGFEE AHHENFSSEV
551 SNSELNENEF VESDKSVTEP AEHEEVVSEE SNPEPAENEE SSIEEGHQEE
601 IVPEQNDEES GESGLVDNEE GDFEEPNHEE FEPDQNDSEL SENELVESEK
651 SVSEPAEHVE IVPSQTNNNE NIETIKPEEK KNEFSVEEK**A** **IPQEPVVPTL**
701 **NENENVPPKP** **SEGESTKPDI** **VQIK**IVQENK PNKKETPVVD GPK**HVEQNIQ**
751 **EDDNDEEDDD** **DIDFEGLSR**K DDEKDSSNKN KKK**SSFITYI** **STK**KFKKVSQ
801 TIVSVMINAY DGVIQVVSTI KGIAKDIVIF FQNI

Peptides used for identification

Peptides shown in bold have been analysed by MS/MS sequencing

Start - End	Observed	Mr(expt)	Mr(calc)	Delta	Miss	Sequence
61 - 79	712.33	2133.96	2133.97	-2	1	R.GSNDELNKNSEDVLEQSEK.S (Ions score 58)
61 - 79	712.33	2133.97	2133.97	0	1	R.GSNDELNKNSEDVLEQSEK.S (Ions score 49)
61 - 79	712.33	2133.97	2133.97	1	1	R.GSNDELNKNSEDVLEQSEK.S (Ions score 29)
210 - 222	780.37	1558.73	1558.73	-1	0	K.NLINEEDSEPFPR.Q (Ions score 74)
210 - 222	780.37	1558.73	1558.73	-1	0	K.NLINEEDSEPFPR.Q (Ions score 67)
210 - 222	780.37	1558.73	1558.73	0	0	K.NLINEEDSEPFPR.Q (Ions score 74)
690 - 724	949.25	3792.97	3792.98	-3	0	K.AIPQEPVVPTLNENENVPPKPSEGESTKPDIVQIK.I (Ions score 22)
690 - 724	949.25	3792.98	3792.98	0	0	K.AIPQEPVVPTLNENENVPPKPSEGESTKPDIVQIK.I (Ions score 22)
690 - 724	949.25	3792.98	3792.98	0	0	K.AIPQEPVVPTLNENENVPPKPSEGESTKPDIVQIK.I (Ions score 11)
744 - 769	1031.09	3090.24	3090.24	0	0	K.HVEQNIQEDDNDEEDDDDIDFEGLSR.K (Ions score 122)
744 - 769	1031.09	3090.24	3090.24	0	0	K.HVEQNIQEDDNDEEDDDDIDFEGLSR.K (Ions score 113)
784 - 793	573.80	1145.59	1145.60	-2	0	K.SSFITYISTK.K (Ions score 29)
784 - 793	573.81	1145.60	1145.60	0	0	K.SSFITYISTK.K (Ions score 27)

Alphalyse A/S ■ Unsbjergvej 4 ■ 5220 Odense ■ Denmark
Tel: +45 6310 6500 ■ e-mail info@alphalyse.com
www.alphalyse.com

Sample name: 9

Protein Information

**Protein name:**	**Uncharacterized protein OS=Plasmodium falciparum FCH/4 GN=PFFCH_03287 PE=4 SV=1**
Entry name:	A0A024VN30_PLAFA
Calculated MW:	108332
Calculated pI:	5.4
Mascot score:	231
Sequence coverage:	6%
Bioinformatic tools:	1: UniProt Entry 2: Conserved Domains in NCBI

Analysis Information

- Enzyme: Trypsin
- Variable modifications: Oxidation (M)
- Fixed modifications: Carbamidomethyl (C)
- Database search program: Mascot version 2.4
- Peptide Tolerance: 10 ppm
- Database: UniprotTREMBL (50011027 protein sequences)

Protein sequence

Matched peptides shown in bold underline

```
  1 MYVRNYGEAN FSFFQATPAS HIEEPQKTES TDAQSNITQN EEINNTKPLQ
 51 ENITNNQQNS NEQQNNNEQQ NNIEQQNNNI QQNNIDTSIS HVPNDTIKNP
101 IDNSNISNLD KSNDTNNVIK HEENNQTKEN NLETIKHTEP LTNQNTNEVK
151 INDHNEHEKN TEENKIEEQY NNINHHSHDI NKNQSINNNM NNENADKNVP
201 HLDQSAMSNE KHINEHTTNH PIESHTGENN HDKINEPIPI EHATTPTNEP
251 IPIEHAATPT NEPIPIEHAA TPTNEPIHIE HAATPTNEPI HIEHVATPAN
301 EPTHIHPNDK LALVPFQGIK NPIPSNESQP IISFPNEDDN HAQNEGSINA
351 PSEGEHNNTD NKEGPIITPL EGEQAGTAHK EDVTHKHMVG EHVPPQKTHH
401 GPIITPVGGN HVPPQTHHAH IITPVGGEHA HGQGNNDTTY VTMNTDESSS
451 SDTKGEHSNL RSYNKNMNNN HAQRDQYDSD TLNSEGSDDA YSSMQQNFEK
501 NGIDSFKGKG LHVSLRERII IEIMESAKNG IDGLLKLKDS KDSGKLFMEA
551 LEKLNINMKD LKKDKNLISL EVYDKILSTM FKILTEMSFY EDSKFYETLG
```

601 IKK**DILNQSL** **KDIK**IKMLRK LGVSYSRLPP IIKHTEGK**CA** **IKDIIISISS**
651 **K**ELAQRMAIM FTKWLAPDEY GAVVDYENNV ELNVLCSGAP ILIQQWKYYQ
701 NMLGFEEDKD HAYLGLIDEL LVMNKRYSQN KDYVETLEKI KKSKVFKHCT
751 KIMRIGGK**VS** **SVPFNYENVK** KPSSSIIGSL GNLIKANIST YYKATAQRIN
801 SYFHYTEKKS KKSSPLKIIS VCTLLHLTDM LYKCSDENSN GVMDLYNLQL
851 NTLNMKGK**MV** **LQYLVHLK**FL TQEKKNQLK**E** **ICEPQNGLID** **ETLTK**MLILL
901 STDSHELLSH ELENKGFDED YIQDEIKNIN ESDNNIRDKE EDDAEKMIFD

Alphalyse A/S ■ Unsbjergvej 4 ■ 5220 Odense ■ Denmark
Tel: +45 6310 6500 ■ e-mail info@alphalyse.com
www.alphalyse.com

951 DL

Peptides used for identification

Peptides shown in bold have been analysed by MS/MS sequencing

Start - End	Observed	Mr(expt)	Mr(calc)	Delta	Miss	Sequence
604 - 614	643.87	1285.72	1285.72	-4	1	K.DILNQSLKDIK.I (Ions score 22)
604 - 614	643.87	1285.72	1285.72	-2	1	K.DILNQSLKDIK.I (Ions score 50)
604 - 614	643.87	1285.72	1285.72	-1	1	K.DILNQSLKDIK.I (Ions score 24)
604 - 614	643.87	1285.72	1285.72	-1	1	K.DILNQSLKDIK.I (Ions score 12)
639 - 651	724.41	1446.81	1446.81	-3	1	K.CAIKDIIISISSK.E (Ions score 56)
639 - 651	724.41	1446.81	1446.81	-1	1	K.CAIKDIIISISSK.E (Ions score 74)
639 - 651	724.41	1446.81	1446.81	2	1	K.CAIKDIIISISSK.E (Ions score 53)
759 - 770	691.85	1381.68	1381.69	-3	0	K.VSSVPFNYENVK.K (Ions score 32)
759 - 770	691.85	1381.69	1381.69	-1	0	K.VSSVPFNYENVK.K (Ions score 33)
759 - 770	691.85	1381.69	1381.69	0	0	K.VSSVPFNYENVK.K (Ions score 16)
759 - 770	691.85	1381.69	1381.69	1	0	K.VSSVPFNYENVK.K (Ions score 27)
859 - 868	630.36	1258.71	1258.71	-2	0	K.MVLQYLVHLK.F + Oxidation (M) (Ions score 17)
859 - 868	630.36	1258.71	1258.71	-1	0	K.MVLQYLVHLK.F + Oxidation (M) (Ions score 12)
880 - 895	930.46	1858.90	1858.90	-1	0	K.EICEPQNGLIDETLTK.M (Ions score 45)
880 - 895	930.46	1858.90	1858.90	2	0	K.EICEPQNGLIDETLTK.M (Ions score 57)

Alphalyse A/S ■ Unsbjergvej 4 ■ 5220 Odense ■ Denmark
Tel: +45 6310 6500 ■ e-mail info@alphalyse.com
www.alphalyse.com

Sample name: 9

Protein Information

Protein name:	Glyceraldehyde-3-phosphate dehydrogenase OS=Plasmodium falciparum Vietnam Oak-Knoll (FVO) GN=PFFVO_05270 PE=3 SV=1
Entry name:	A0A024V013_PLAFA
Calculated MW:	37068
Calculated pI:	7.59
Mascot score:	54
Sequence coverage:	4%
Bioinformatic tools:	1: UniProt Entry 2: Conserved Domains in NCBI

Analysis Information

- Enzyme: Trypsin
- Variable modifications: Oxidation (M)
- Fixed modifications: Carbamidomethyl (C)
- Database search program: Mascot version 2.4
- Peptide Tolerance: 10 ppm
- Database: UniprotTREMBL (50011027 protein sequences)

Protein sequence

Matched peptides shown in bold underline

1 MAVTKLGING FGRIGRLVFR AAFGRKDIEV VAINDPFMDL NHLCYLLKYD
51 SVHGQFPCEV THADGFLLIG EKKVSVFAEK DPSQIPWGKC QVDVVCESTG
101 VFLTKELASS HLKGGAKKVI MSAPPKDDTP IYVMGINHHQ YDTKQLIVSN
151 ASCTTNCLAP LAKVINDRFG IVEGLMTTVH ASTANQLVVD GPSKGGKDWR
201 AGRCALSNII PASTGAAKAV GKVLPELNGK LTGVAFR<u>**VPI**</u> <u>**GTVSVVDLVC**</u>
251 <u>**R**</u>LQKPAKYEE VALEIKKAAE GPLKGILGYT EDEVVSQDFV HDNRSSIFDM
301 KAGLALNDNF FKLVSWYDNE WGYSNRVLDL AVHITNN

Peptides used for identification

Peptides shown in bold have been analysed by MS/MS sequencing

Start - End	Observed	Mr(expt)	Mr(calc)	Delta	Miss	Sequence
238 - 251	757.42	1512.83	1512.83	1	0	**R.VPIGTVSVVDLVCR.L** (Ions score 54)
238 - 251	757.43	1512.84	1512.83	4	0	**R.VPIGTVSVVDLVCR.L** (Ions score 49)

Alphalyse A/S ■ Unsbjergvej 4 ■ 5220 Odense ■ Denmark
Tel: +45 6310 6500 ■ e-mail info@alphalyse.com
www.alphalyse.com

LC-MSMS Protein Identification Report

Order 16372_Plasmodium falciparum

Sample name: 9

Protein Information

**Protein name:**	**Phosphoenolpyruvate carboxykinase (ATP) OS=Plasmodium falciparum Vietnam Oak-Knoll (FVO) GN=PFFVO_04304 PE=3 SV=1**
Entry name:	A0A024V3K4_PLAFA
Calculated MW:	66794
Calculated pI:	6.01
Mascot score:	37
Sequence coverage:	2%
Bioinformatic tools:	1: UniProt Entry 2: Conserved Domains in NCBI

Analysis Information

- Enzyme: Trypsin
- Variable modifications: Oxidation (M)
- Fixed modifications: Carbamidomethyl (C)
- Database search program: Mascot version 2.4
- Peptide Tolerance: 10 ppm
- Database: UniprotTREMBL (50011027 protein sequences)

Protein sequence

Matched peptides shown in bold underline

1 MVEKSSDYDL AEKMEDMKKI VIEEVRRNLI YKKPIGPIMS SKDILTLSQE
51 QESKFNEEVH ELGLHVNSIH HNSTPAFLYE MALKYEGNSF ITSTGALCCI
101 SGEKTGRSPS DKRIVQEKSS EDDIWWGNVN IPIKEKSYEI NKSRAIDYLN
151 LQPNLYVIDA YAGWDERCRI KVRVITSRAY HALYMLNMLI PPKNAEEIQN
201 FVPDFIIYNA GEFPSNRLTD GMSSKTSVIL NFGSMNMVIL GTQYAGEMKK
251 GILTLFMYKM PKEGKLPLHS SCNIGKKNDV TLFFGLSGTG KTTLSADANR
301 YLIGDDEHVW TDDGIFNIEG GCYAKCKGLS KRQEPEIYKA IK**FGAILENV**
351 **VMDPVTR**EVD YNNCTITENT RCAYPLSYIE NAKIPAYIHT HPQNIILLTC
401 DAFGVIPPLC KLDVYQMMYH FVSGYTSKMA GTEDNILKPT ATFSSCYAAP
451 FLALHPMIYA QMLADKYQKH KPNVWLLNTG WIYGSYGSDN GIRIPLKYTR
501 LLVDYIHENK LNNIQYKKTP IFNFNIPEHL EGIPDEVIDP LIGWKDKEDY
551 LTNLQILAKE FINNFSLYLD KAGPEILSGG PNL

Peptides used for identification

Peptides shown in bold have been analysed by MS/MS sequencing

Start - End	Observed	Mr(expt)	Mr(calc)	Delta	Miss	Sequence
343 - 357	830.94	1659.86	1659.87	-6	0	K.FGAILENVVMDPVTR.E (Ions score 37)
343 - 357	830.94	1659.86	1659.87	-5	0	K.FGAILENVVMDPVTR.E (Ions score 35)

Alphalyse A/S ■ Unsbjergvej 4 ■ 5220 Odense ■ Denmark
Tel: +45 6310 6500 ■ e-mail info@alphalyse.com
www.alphalyse.com
